# Supplementary material for: Deciphering complexity in Pd–catalyzed cross-couplings
Source: Nat Commun. 2024 May 10;15:3968. doi: 10.1038/s41467-024-47939-5 (PMC11087562; doi:10.1038/s41467-024-47939-5)
Supplement: Supplementary file 1 — Supplementary Information [file 41467_2024_47939_MOESM1_ESM.pdf]

Supplementary Information

Deciphering Complexity in Pd–Catalyzed Cross-Couplings

George E. Clarke,[1,⁋] James D. Firth,[1,⁋] Lyndsay A. Ledingham,[1,⁋] Chris S. Horbaczewskyj,[1] Richard Bourne,[2] Joshua T. W. Bray,[1] Poppy L. Martin,[1] Jonathan B. Eastwood,[1] Rebecca Campbell,[1] Alex Pagett,[1] Duncan J. MacQuarrie,[1] John M. Slattery,[1] Jason M. Lynam,[1] Adrian C. Whitwood,[1] Jessica Milani,[1] Sam Hart,[1] Julie Wilson,*[,3] and Ian J. S. Fairlamb*[,1]

[1] Department of Chemistry, University of York, Heslington, York, YO10 5DD, UK
[2] School of Chemistry, University of Leeds, Woodhouse Lane, Leeds, UK.
[3] Department of Mathematics, University of York, Heslington, York, YO10 5DD, UK

⁋ These authors contributed equally to this work. * Joint corresponding authors.

Email for correspondence: ian.fairlamb@york.ac.uk; julie.wilson@york.ac.uk

Table of Contents

1. Supplementary Notes 4

1.1 General Experimental Details 4

1.2 High-Throughput Experimentation Setup 7

1.3 Analytical Methods and Data Processing 8

1.4 Statistical Analysis 9

1.4.1 Principal Component Analysis (PCA) 9

1.4.2 Correspondence Analysis (CA) 9

1.4.3 Heatmaps and Hierarchical Clustering (HCA) 10

2. Supplementary Methods 11

2.1 Procedure for High Throughput Experimentation Reaction Screening 11

2.2 Initial Reaction Condition Screening 16

2.3 Synthesis and Characterization of Compounds in Figure 3 17

2.4 *In Operando* Monitoring of the Reaction of **1a** in DMF at 80 °C using IR Spectroscopic Analysis 24

2.5 Exemplar LC-MS traces and Retention times of Compounds of Interest 25

2.6 Identification of By-Products and Side-Products 28

2.6.1 Isolation and Characterization of Side-Products from Large Scale Reactions of **1a**, **1a-*d*$_5$** and **1b** 28

2.6.2 Observation by HRMS From Large Scale Reactions of **1a**, **1a-*d*$_5$** and **1b** 36

2.6.3 Confirmation of Additional Side-Products Structure by Chromatographic Methods 37

2.6.4 Confirmation of Side-Product Structure by LC-MS 37

2.6.5 Confirmation of Additional Side-Product Structures Not Observed by HTE 38

2.6.6 Compounds Shown to Not Be Present in Reaction Mixtures 39

2.6.7 Origin of the ‘new’ phenyl ring **10a** and **12a** 40

2.6.8 Synthesis of Analytical Standards 42

2.7 Mechanistic Studies by ^{31}P NMR and Mass Spectrometry Experiments 69

2.7.1 Identification of the active Pd^0 catalytic Species Pd(dppe) **I** 69

2.7.2 Identification of Oxidative Addition products by ^{31}P NMR and HRMS 72

3. Supplementary Discussion 76

3.1 PCA plot of all Times and Temperature 76

3.2 Principal component analysis of all Times and Temperature 77

3.3 X-Ray Crystallographic Data for Single Crystals 78

3.4 Representative NMR Spectra of Compounds 81

6. Supplementary References 123

1. Supplementary Notes

1.1. General Experimental Details

Commercially sourced solvents and reagents were purchased from Acros Organics, Alfa Aesar, Fisher Scientific, Fluorochem, Sigma-Aldrich or VWR and used as received unless otherwise noted. Petrol refers to the fraction of petroleum ether boiling in the range of 40–60 °C. Room temperature (RT) refers to reactions where no thermostatic control was applied and was recorded as 16–23 °C (typically 21 °C).

Thin layer chromatography (TLC) analysis was performed using Merck 5554 aluminum backed silica plates. Spots were visualized by the quenching of ultraviolet light (λ_{max} = 254 nm) or through staining with $KMnO_4$. Retention factors (R_f) are quoted to two decimal places and reported along with the solvent system used in parentheses. All flash column chromatography was performed using either Merck 60 or Fluorochem 60 Å silica gel (particle size 40–63 μm) and the solvent system used is reported in parentheses.

Melting points were recorded using a Stuart digital SMP3 machine using a temperature ramp of 5 °C min^{-1} and are quoted to the nearest whole number. Where applicable, decomposition (dec.) is noted.

All NMR spectra were recorded on Jeol ECS400, Jeol ECX400 or Bruker AV500 spectrometers (typically at 298 K). ^1H Chemical shifts are reported in parts per million (ppm), typically using a tetramethyl silane reference standard. Coupling constants (J) are reported in Hz and quoted to ±0.5 Hz. Multiplicities are described as singlet (s), doublet (d), triplet (t), quartet (q), quintet (quin), sextet, (sext), heptet (hept), multiplet (m), apparent (app) and broad (br). Spectra were processed using MestReNova. NMR spectra are representative of the compounds prepared.

Proton (^1H) spectra were typically recorded at 400 MHz. Compound chemical shifts were internally referenced to residual non-deuterated solvent ($CHCl_3$ δH = 7.26 ppm), given to two decimal places.

Carbon-13 (^{13}C) spectra were typically recorded at 101 MHz. Compound chemical shifts were internally referenced to residual solvent ($CDCl_3$ δC = 77.16 ppm) and given to one decimal place.

Boron-11 (^{11}B) spectra were recorded at 128 MHz and obtained with ^1H decoupling. Chemical shifts were externally referenced to $BF_3 \cdot OEt_2$ and given to one decimal place.

Phosphorus-31 (^{31}P) NMR spectra were recorded at 162 or 203 MHz and carried out with ^1H decoupling. ^{31}P NMR spectra were typically recorded using 128 scans and a spectral window of 300 ppm (δ 250 to –50 ppm). Chemical shifts are externally referenced to 85% H_3PO_4 in H_2O (w/w) and given to one decimal

place. Referencing was practically carried out by inserting a sealed, vacuum dried capillary tube containing 85% H_3PO_4 in H_2O (w/w) into an NMR tube containing the sample of interest, collecting a ^{31}P NMR spectrum and setting the H_3PO_4 resonance to 0 ppm.

Electrospray ionisation (ESI) mass spectrometry was performed using a Bruker Daltronics micrOTOF spectrometer. Electron impact (EI) mass spectrometry was performed using a Waters GCT Premier mass spectrometer. Mass to charge ratios (m/z) are reported in Daltons with percentage abundance in parentheses along with the corresponding fragment ion, where known. Where complex isotope patterns were observed, the most abundant ion is reported. High resolution mass spectra (HRMS) are reported with less than 5 ppm error.

Infrared spectra were recorded on a Bruker Vertex 70, a Bruker Alpha or Perkin Elmer UATR Two FT-IR spectrometer. Absorption maxima (ν_{max}) are reported in wavenumbers (cm^{-1}) to the nearest whole number.

Preparative HPLC was performed using a Varian Pro Star Preparative HPLC using a SunFireTM Prep C18 OBMTM 10 µm (19 × 250 mm). Method conditions: LC run time: 20 mins. Total flow rate: 17.1 mL/min. Solvent System Method: 20:80 MeCN:water to 90:10 MeCN:water over 15 mins then decrease to 20:80 MeCN:water at constant rate over 5 mins 5 secs. Hold for 4 mins (equilibrium time). UV detector at 254 nm. All solvents included 0.1% formic acid.

GC-MS was performed either on a GCT premier TOF mass spectrometer using Agilent 7890A GC or JEOL AccuTOF GCx-plus using Agilent 7890B GC. A Zebra ZB1 MS (30 m length, 250 µm internal diameter, and 0.25 µm film thickness) column was used. Method conditions: initial temperature of 50 °C, hold for 1 min. Ramp rate: 21 °C / min until 300 °C, hold for 7 mins. Total run time: 20 mins.

Diffraction data were collected at 110 K on an Oxford Diffraction SuperNova diffractometer with either Mo-K_α radiation (λ = 0.71073 Å CCDC 2063167), or Cu-K_α radiation (λ = 1.54184 Å, CCDC 2063164-6 and 2081723-4), using a EOS CCD camera. The crystal was cooled with an Oxford Instruments Cryojet. Diffractometer control, data collection, initial unit cell determination, frame integration and unit-cell refinement was carried out with "Crysalis".[1] Face-indexed absorption corrections were applied using spherical harmonics, implemented in SCALE3 ABSPACK scaling algorithm.[2] OLEX2[3] was used for overall structure solution, refinement and preparation of computer graphics and publication data. Within OLEX2, the algorithm used for structure solution was "Superflip charge-flipping"[4] or "ShelXT dual-space"[5] (CCDC 2081723-4). Refinement by full-matrix least-squares used the SHELXL-97[6] algorithm within OLEX2.[3] All non-hydrogen atoms were refined anisotropically. Hydrogen atoms were placed

using a “riding model” and included in the refinement at calculated positions. For CCDC 2063165, the molecule adopted two different orientations and was modelled by placing the amide in two positions with the refine occupancy 0.708: 0.292(3). The C-C distances, near the amide, and the C-N distances in the major and minor components were restrained to be similar. Due to the close proximity, the corresponding atoms on the major and minor components were constrained to have the same ADP (*e.g.* N1 and N1').

1.2 High-Throughput Experimentation Setup

Screening reactions were carried out on a Chemspeed Swing robot equipped with a four needle (0.8 mm ID) liquid dispensing tool and 2 × 1 mL and 2 × 10 mL syringes, a SDU solid dispensing tool, a Huber Unistat Tango (heating) and Huber UC015 Advanced (reflux cooling). Reactions were performed under air in a 48 × 8 mL ISYNTH reaction block (Supplementary Figure 1). Solvent was removed under vacuum. Line priming solvent was MeCN. Software used is Chemspeed AutoSuite version 2.2.33.3. Reactions were performed in triplicate and well positions were randomized.

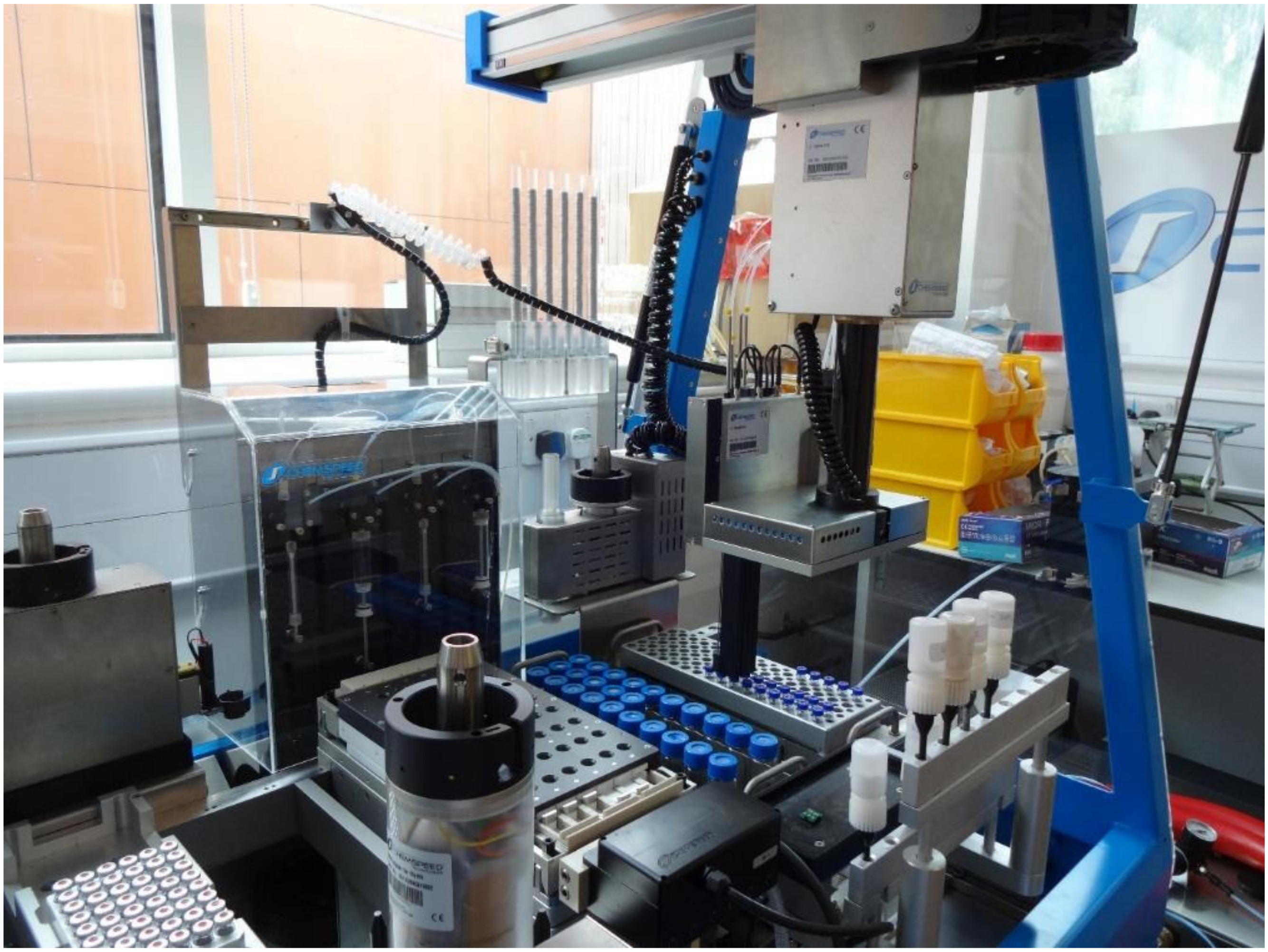

Supplementary Figure 1. Chemspeed robot deck during HTE screening experiments.

1.3 Analytical Methods and Data Processing

LC-MS was performed on Bruker HCTultra ETD II spectrometer and a Dionex Ultimate 3000 LC. *Column*: Ascentis® Express C18 2.7 μm, 50 × 4.6 mm;

Volume injected: 10 μL.

Solvent: MeCN and H_2O, both including 0.1% formic acid

Flow rate: 500 μL/min.

Column oven: 25 °C.

Mobile phase program: 0.0-0.50 min 10% MeCN, 0.50-10.0 min gradient to 90% MeCN, 10.0-14.0 min hold at 90% MeCN, 14.0-14.2 min gradient to 10% MeCN, 14.2-17.0 min hold at 10% MeCN. *Electrospray ionization (ESI) mass spectrometry conditions*: Nebulizer pressure: 40 psi, Dry Gas: 8 L/min, Dry Temperature: 350 °C.

Column Preparation: At the start of LC-MS data collection, a blank water or MeCN sample was run first, followed by 20 Quality Control (QC) sample injections in order to equilibrate the column to the type of compounds present in the experimental samples. The QC samples contained caffeine (0.80 mM), hexamethylbenzene (0.28 mM), 2-bromo-*N*-phenylbenzamide (starting material **1a** - 0.50 mM), and phenanthridinone **2a** – 0.23 mM). Further QC samples were injected after every five reaction samples.

LC-MS samples were screened in batches of 180 (two solvents, six temperatures and five reaction times performed in triplicate) and were run in a random order within each batch. Internal standard (hexamethylbenzene) and external standard (caffeine - 0.80 mM) were used.

Peak picking and alignment in each batch of the LC-MS data was carried out using Progenesis QI software (nonlinear.com)[7] and the peak areas for each observation exported as Excel spreadsheet files. Section 2.5 shows an exemplar chromatogram and details the peaks of interest. Code was written in-house to extract the peaks corresponding to the chosen reaction products and a data matrix produced with rows corresponding to observations (for each reaction time, temperature and solvent) and columns corresponding to the integrated peaks of the different reaction products. The data were then normalised by dividing the integrated value for each peak by the total for all chosen peaks and multiplying by 100 so that the chosen peaks are represented as percentages. Exploratory analyses showed reduced variability between replicate analyses after this normalization step.

All data from LC-MS is available in raw form, see:

https://pure.york.ac.uk/portal/en/datasets/deciphering-complexity-in-pdcatalyzed-cross-couplings

1.4 Statistical Analysis

All data analyses were carried out in the R programming environment (R Core Team, 2020).[8]

1.4.1 Principal Component Analysis (PCA)

Principal component analysis (PCA) allows patterns in data with multiple variables to be visualized. This is achieved by a rotation of the multi-dimensional axes, where each axis corresponds to a different variable (here, integrated peak intensity for a particular species), in such a way that a small subset of the new axes, or principal components, provides most of the information in the data. The rotation preserves the orthogonality of the axes but the first principal component is chosen as the direction of maximum variance in the data, the second principal component corresponds to the next most variance (orthogonal to the first) and so on. In this way, pairwise scatter plots showing the coordinates of the observations in relation to the first few components can show most of the variance, or information, in the data. The coordinates along the principal components are known as scores and often the scores plot for just the first two components is sufficient to reveal patterns in the data. As the principal components are obtained by rotating the original axes, each component is a linear combination of the original variables. This means that the contribution of any variable to a particular component can be determined from its coefficient, or loading, in the linear combination; a loading that is large in absolute value shows the corresponding variable to be important for any trends or patterns seen for that component.

As their variance is likely to be greater than for small peaks, large peaks can dominate the analysis and differences due to small peaks can be masked unless the variables are rescaled. There are several methods for scaling variables, but the most common is UV-scaling, also known as Pareto scaling or scaling to unit variance. In this method, each variable is rescaled by subtracting the mean and dividing by the standard deviation (over all observations for that variable). This results in mean-centered data in which every variable has a variance of one, giving all variables equal influence on the analysis. Mean-centering is performed even when the data is not scaled.

1.4.2 Correspondence Analysis (CA)

Correspondence analysis is used to assess the level of association between categorical variables. A table of count data is produced with rows corresponding to the different categories of one variable and columns corresponding to the categories of another in which the number in the jth column of row i is the number of observations having both the ith attribute of the first (row) variable and the jth attribute of the second (column) variable. For example, the first variable could be hair colour with categories dark, blond and red and the second variable could be eye colour with categories brown, blue and green. If the two variables are related, we might expect to see more observations of red hair and green eyes than red hair

with either blue or brown eyes. Associations are tested by comparing with the numbers that would be expected if there were no associations and all possibilities were equally likely. The differences between these expected values and the observed counts, known as residuals, are then plotted so that similar residuals are close together. The closer two row labels are together, the more similar their residuals. Similarly, for the column labels. However, when interpreting a correspondence plot, it is important to consider how far from zero the labels are plotted as small residuals do not provide evidence of any association. When comparing row and column labels with each other, the angle between them needs to be considered as well as the distance from the origin. Small angles indicate strong association whereas an angle of $\pi/2$ shows no association and an angle close to π indicates a negative association.

1.4.3 Heatmaps and Hierarchical Clustering (HCA)

The values in a matrix to be visualized in a heatmap by coloring the pixel in row *i* and column *j* to reflect the value of the corresponding element in the matrix, according to a given color code. For example, a heatmap can be used to represent a correlation matrix. The rows and columns of the matrix are often rearranged first using hierarchical cluster analysis so that groups of similar observations form blocks of similar color in the heatmap. Agglomerative hierarchical clustering begins with every observation forming a separate cluster and the distance measure between observations converted to a distance measure between clusters (*e.g.* the average over all pairwise distances between observations with one from each cluster). Pairs of clusters are then combined in a hierarchical algorithm reflecting their similarity until finally all observations belong to the same cluster. A dendrogram provides a graphical representation of the clustering in which the lengths of the vertical lines indicate the distances at which clusters are joined and is often shown in the margins of a heatmap.

2. Supplementary Methods

2.1. Procedure for High Throughput Experimentation Reaction Screening

Reactions were performed in triplicate and well positions were randomized. Each run consisted of using a single solvent at six different temperatures sequentially.

Solvents used: DMF, propylene carbonate, MeCN, BuCN, methyl ethyl ketone (MEK), Bu_2O, toluene and a 9:1 Bu_2O:DMF mixture.
Temperatures: 80, 90, 100, 110, 120, 130 °C.

Reaction setup: A solution of each of the following were added to the 8 mL reaction vials in the ISYNTH reactor using the 4-needle head and then concentrated under reduced pressure sequentially: 2-bromo-*N*-phenylbenzamide **1a** (1.0 mL of a 0.2 M solution in CH_2Cl_2, 0.20 mmol, 1.0 eq.), dppe (1.0 mL of a 10 mM solution in CH_2Cl_2, 0.010 mmol, 5 mol%), $Pd(OAc)_2$ ((1.0 mL of a 10 mM solution in CH_2Cl_2, 0.010 mmol, 5 mol%). Then, K_2CO_3 (55 mg, 0.40 mmol, 2.0 eq.) was added to each reaction vial via the SDU. Then, a solution of hexamethylbenzene solution (0.0425 M) in solvent (1 mL) was added to the reaction vials. The reaction vials were shaken at 500 rpm and heated at the desired temperature for 24 h.

Reaction sampling: After 1 h, 2 h, 4 h, 8 h and 24 h, a 10 µL sample was taken from each reaction vial and transferred into a 2 mL HPLC vial containing a solution of caffeine (1.5 mL of a 0.8 mM solution in MeCN). Analysis was performed using off-line LC-MS (see section 1.3).

Supplementary Figure 2 shows a simplified workflow of the HTE Chemspeed protocol for a single solvent. Supplementary Figures 3, 4 and 5 show the Chemspeed deck layout and tasks.

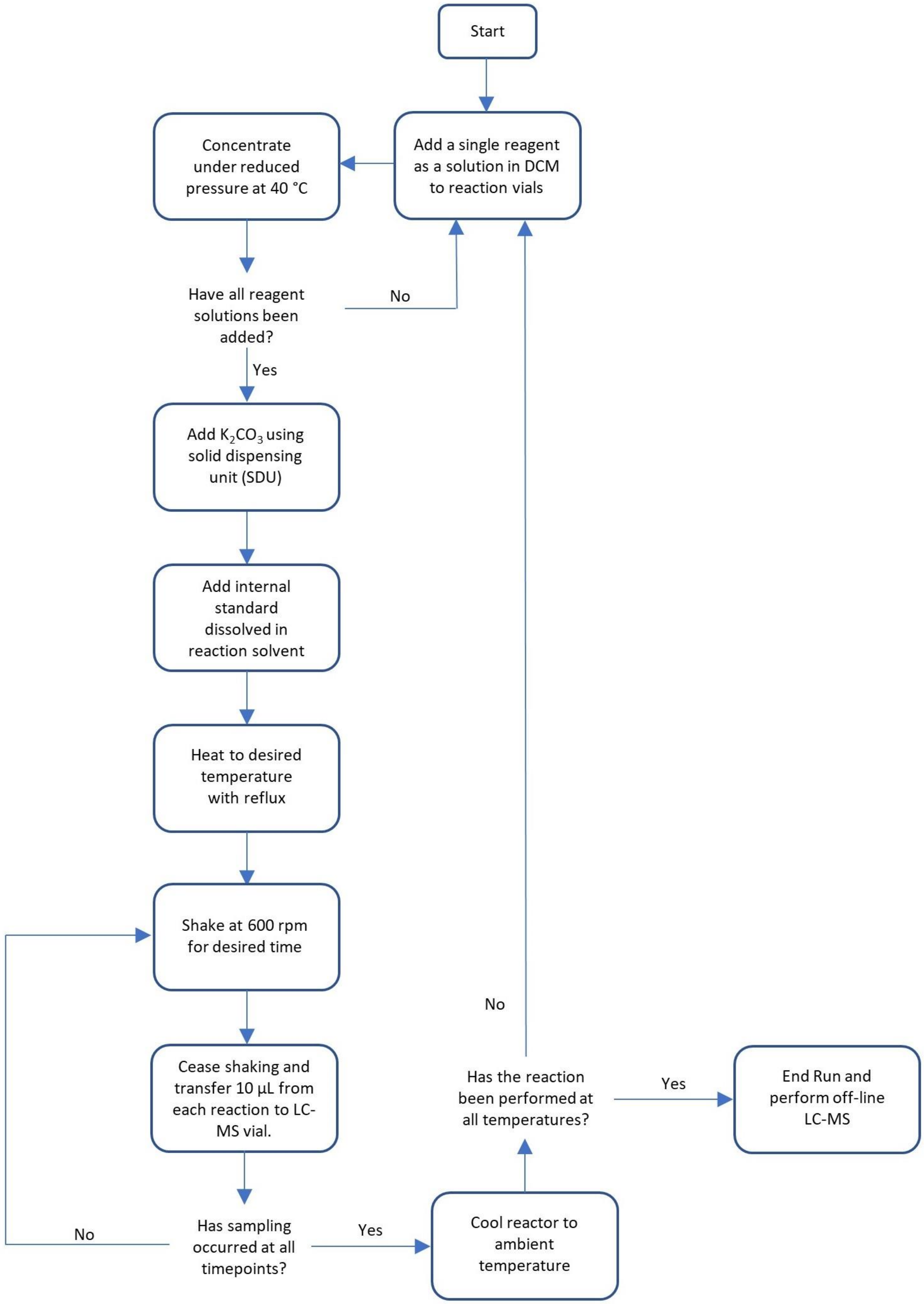

Supplementary Figure 2. Simplified workflow of the Chemspeed ISYNTH HTE protocol for a single solvent.

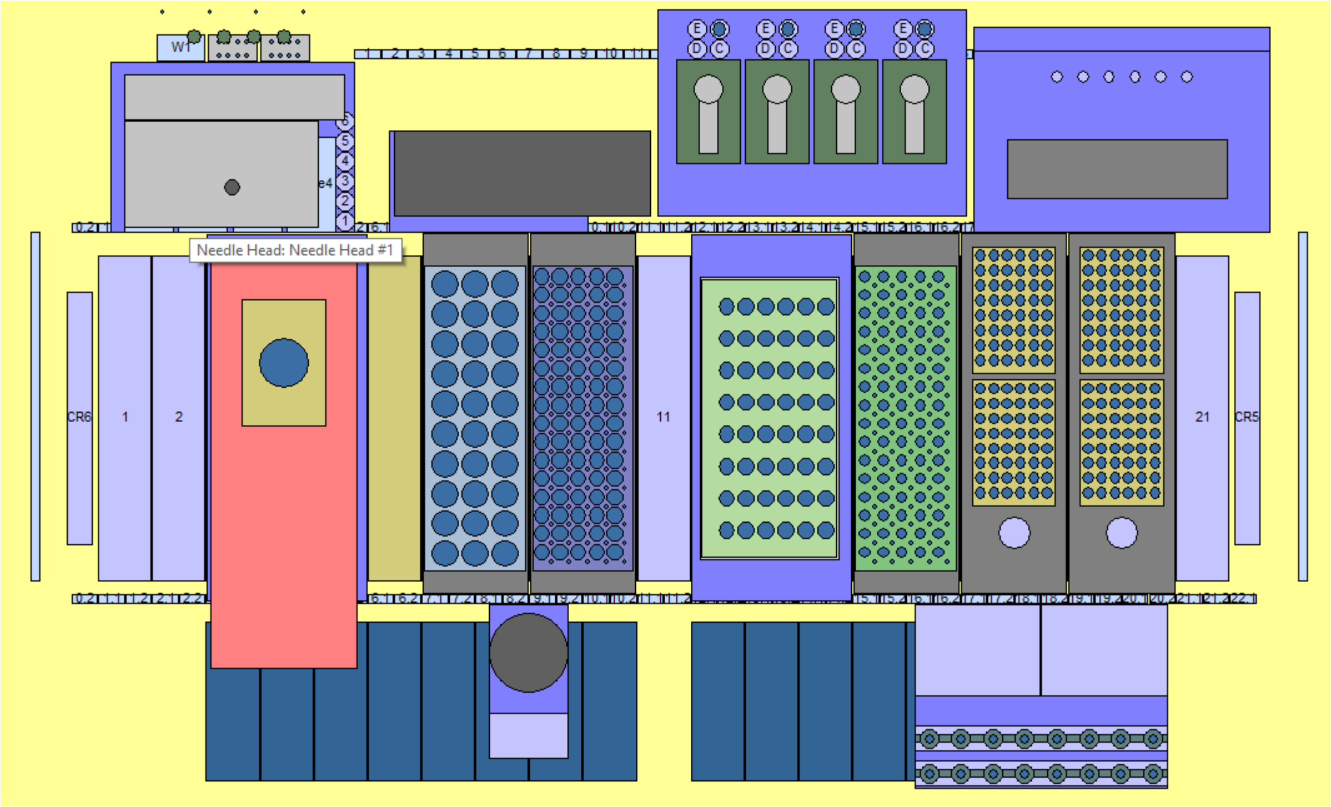

Supplementary Figure 3. Chemspeed ISYNTH Deck Layout.

Task	Name	Parameter	Description	Estimated Time	Task Number
1	Transfer Volumetrically	Transfer liquid from valve ports B to Waste 1 with Needle Head #1		06 min 13 sec	1
2	80 C reactions	Execute Once		25 hours 31 min 11 sec	2
3	Transfer Volumetrically	Transfer liquid from valve ports B to Waste 1 with Needle Head #1		06 min 13 sec	3
4	90 C reactions	Execute Once		25 hours 31 min 11 sec	4
5	Transfer Volumetrically	Transfer liquid from valve ports B to Waste 1 with Needle Head #1		06 min 13 sec	5
6	100 C reactions	Execute Once		25 hours 31 min 11 sec	6
7	Transfer Volumetrically	Transfer liquid from valve ports B to Waste 1 with Needle Head #1		06 min 13 sec	7
8	110 C reactions	Execute Once		25 hours 31 min 11 sec	8
9	Transfer Volumetrically	Transfer liquid from valve ports B to Waste 1 with Needle Head #1		06 min 13 sec	9
10	120 C reactions	Execute Once		25 hours 31 min 11 sec	10
11	Transfer Volumetrically	Transfer liquid from valve ports B to Waste 1 with Needle Head #1		06 min 13 sec	11
12	130 C reactions	Execute Once		25 hours 31 min 11 sec	12

Supplementary Figure 4. Simple overview of Chemspeed ISYNTH tasks.

Task	Name	Parameter	Description	Estimated Time	Task Number
1	Transfer Volumetrically	Transfer liquid from valve ports B to Waste 1 with Needle Head #1		06 min 13 sec	1
2	80 C reactions	Execute Once		25 hours 31 min 11 sec	2
1	Liquid Addition -Substrate	Execute Once		00 sec	2.1
1	Set Drawer Valve	Set Reactionblock state to Open Without Inert Gas on zone ISYNTH		00 sec	2.1.1
2	Macro Task	Execute Once		00 sec	2.1.2
1	Set Variable	Row := Row + 1		00 sec	2.1.2.1
2	Import CSV	import row 'Row' from file 'Substrate_dist_310519.csv''.		00 sec	2.1.2.2
3	Transfer Volumetrically	Transfer liquid from Substrate in DCM to variable 'Seq_Reactors' wi		00 sec	2.1.2.3
<insert sub tasks here>					
<insert sub tasks here>					
2	Reagent Solution Conc	Execute Once		30 min 15 sec	2.2
1	Set Drawer Valve	Set Reactionblock state to Closed Under Vacuum on zone ISYNTH a		00 sec	2.2.1
2	Set Vacuum	Vacuum On on zone ISYNTH all		02 sec	2.2.2
3	Heat / Cool	Thermostat ON on zone ISYNTH all		02 sec	2.2.3
4	Stir	Agitation ON on zone ISYNTH all		02 sec	2.2.4
5	Wait	Waiting for 30:00 minutes		30 min 00 sec	2.2.5
6	Set Vacuum	Vacuum Off on zone ISYNTH all		02 sec	2.2.6
7	Heat / Cool	Thermostat OFF on zone ISYNTH all		00 sec	2.2.7
8	Stir	Agitation OFF on zone ISYNTH all		02 sec	2.2.8
9	Set Drawer Valve	Set Reactionblock state to Closed Under Inert Gas on zone ISYNTH		00 sec	2.2.9
10	Wait	Waiting for 5 seconds		05 sec	2.2.10
11	Set Drawer Valve	Set Reactionblock state to Open Without Inert Gas on zone ISYNTH		00 sec	2.2.11
<insert sub tasks here>					
3	Liquid Addition - Ligand	Execute Once		00 sec	2.3
1	Macro Task	Execute Once		00 sec	2.3.1
1	Set Variable	Row := Row + 1		00 sec	2.3.1.1
2	Import CSV	import row 'Row' from file 'Ligand_dist_310519.csv''.		00 sec	2.3.1.2
3	Transfer Volumetrically	Transfer liquid from DPPE in DCM to variable 'Seq_Reactors' with N		00 sec	2.3.1.3
<insert sub tasks here>					
<insert sub tasks here>					
4	Reagent Solutions Conc	Execute Once		30 min 15 sec	2.4
1	Set Drawer Valve	Set Reactionblock state to Closed Under Vacuum on zone ISYNTH a		00 sec	2.4.1
2	Set Vacuum	Vacuum On on zone ISYNTH all		02 sec	2.4.2
3	Heat / Cool	Thermostat ON on zone ISYNTH all		02 sec	2.4.3
4	Stir	Agitation ON on zone ISYNTH all		02 sec	2.4.4
5	Wait	Waiting for 30:00 minutes		30 min 00 sec	2.4.5
6	Set Vacuum	Vacuum Off on zone ISYNTH all		02 sec	2.4.6
7	Heat / Cool	Thermostat OFF on zone ISYNTH all		00 sec	2.4.7
8	Stir	Agitation OFF on zone ISYNTH all		02 sec	2.4.8
9	Set Drawer Valve	Set Reactionblock state to Closed Under Inert Gas on zone ISYNTH		00 sec	2.4.9
10	Wait	Waiting for 5 seconds		05 sec	2.4.10
11	Set Drawer Valve	Set Reactionblock state to Open Without Inert Gas on zone ISYNTH		00 sec	2.4.11
<insert sub tasks here>					
5	Liquid Addition - Catalyst	Execute Once		00 sec	2.5
1	Macro Task	Execute Once		00 sec	2.5.1
1	Set Variable	Row := Row + 1		00 sec	2.5.1.1
2	Import CSV	import row 'Row' from file 'Pd_dist_310519.csv''.		00 sec	2.5.1.2
3	Transfer Volumetrically	Transfer liquid from PdOAc in DCM to variable 'Seq_Reactors' with		00 sec	2.5.1.3
<insert sub tasks here>					
<insert sub tasks here>					
6	Reagent Solutions Conc	Execute Once		30 min 15 sec	2.6
1	Set Drawer Valve	Set Reactionblock state to Closed Under Vacuum on zone ISYNTH a		00 sec	2.6.1
2	Set Vacuum	Vacuum On on zone ISYNTH all		02 sec	2.6.2
3	Heat / Cool	Thermostat ON on zone ISYNTH all		02 sec	2.6.3
4	Stir	Agitation ON on zone ISYNTH all		02 sec	2.6.4
5	Wait	Waiting for 30:00 minutes		30 min 00 sec	2.6.5
6	Set Vacuum	Vacuum Off on zone ISYNTH all		02 sec	2.6.6
7	Heat / Cool	Thermostat OFF on zone ISYNTH all		00 sec	2.6.7
8	Stir	Agitation OFF on zone ISYNTH all		02 sec	2.6.8
9	Set Drawer Valve	Set Reactionblock state to Closed Under Inert Gas on zone ISYNTH		00 sec	2.6.9
10	Wait	Waiting for 5 seconds		05 sec	2.6.10
11	Set Drawer Valve	Set Reactionblock state to Open Without Inert Gas on zone ISYNTH		00 sec	2.6.11
<insert sub tasks here>					
7	Solid Import Export	Execute Once		00 sec	2.7
1	Macro Task	Execute Once		00 sec	2.7.1
1	Set Variable	Row := Row + 1		00 sec	2.7.1.1
2	Import CSV	import row 'Row' from file 'Base_dist_310519.csv''.		00 sec	2.7.1.2
3	Transfer Gravimetrically	Gravimetric Transfer with SDU #1 from K2CO3 to variable 'Seq_Rea		00 sec	2.7.1.3
4	Get Property	Read property 'Last Gravimetric Dosage' from variable zone 'Seq_R		00 sec	2.7.1.4
5	Export CSV	'Export in file 'Base export solid.txt''.		00 sec	2.7.1.5
6	Get Property	Read property 'Enabled' from the well in zone 'Last_K2CO3' and stc		00 sec	2.7.1.6
7	Macro Task	Execute If 'not Empty'		00 sec	2.7.1.7
<insert sub tasks here>					
2	Set Drawer Valve	Set Reactionblock state to Open Without Inert Gas on zone ISYNTH		00 sec	2.7.2
<insert sub tasks here>					

#	Task	Details	Duration	Step
8	Liquid Addition - Solvent	Execute Once	06 sec	2.8
1	Macro Task	Execute Once	00 sec	2.8.1
1	Set Variable	Row := Row + 1	00 sec	2.8.1.1
2	Import CSV	import row 'Row' from file 'Solvent_dist_DMF_310519.csv''.	00 sec	2.8.1.2
3	Transfer Volumetrically	Transfer liquid from DMF with IS1 to variable 'Seq_Reactors' with N	00 sec	2.8.1.3
<insert sub tasks here>				
2	Macro Task	Execute Once	00 sec	2.8.2
1	Set Variable	Row := Row + 1	00 sec	2.8.2.1
2	Import CSV	import row 'Row' from file 'Solvent_dist_Toluene_310519.csv''.	00 sec	2.8.2.2
3	Transfer Volumetrically	Transfer liquid from Toluene with IS1 to variable 'Seq_Reactors' witł	00 sec	2.8.2.3
<insert sub tasks here>				
3	Set Drawer Valve	Set Reactionblock state to Closed Without Inert Gas on zone ISYNT	00 sec	2.8.3
4	Stir	Agitation ON on zone ISYNTH all	02 sec	2.8.4
5	Heat / Cool	Thermostat ON on zone ISYNTH all	02 sec	2.8.5
6	Reflux	Reflux On on zone ISYNTH all	02 sec	2.8.6
<insert sub tasks here>				
9	Wait	Waiting for 1:00:00 hours	01 hours 00 min 00 sec	2.9
10	Sampling	Execute Once	04 sec	2.10
1	Stir	Agitation OFF on zone ISYNTH all	02 sec	2.10.1
2	Set Drawer Valve	Set Reactionblock state to Open Without Inert Gas on zone ISYNTH	00 sec	2.10.2
3	Macro Task	Execute Once	00 sec	2.10.3
1	Set Variable	Row := Row + 1	00 sec	2.10.3.1
2	Import CSV	import row 'Row' from file 'Sampling_order_310519.csv''.	00 sec	2.10.3.2
3	Transfer Volumetrically	Transfer liquid from variable 'Seq_Reactors' to variable 'Seq_Sample	00 sec	2.10.3.3
<insert sub tasks here>				
4	Set Drawer Valve	Set Reactionblock state to Closed Without Inert Gas on zone ISYNT	00 sec	2.10.4
5	Stir	Agitation ON on zone ISYNTH all	02 sec	2.10.5
<insert sub tasks here>				
11	Wait	Waiting for 1:00:00 hours	01 hours 00 min 00 sec	2.11
12	Sampling	Execute Once	04 sec	2.12
1	Stir	Agitation OFF on zone ISYNTH all	02 sec	2.12.1
2	Set Drawer Valve	Set Reactionblock state to Open Without Inert Gas on zone ISYNTH	00 sec	2.12.2
3	Macro Task	Execute Once	00 sec	2.12.3
1	Set Variable	Row := Row + 1	00 sec	2.12.3.1
2	Import CSV	import row 'Row' from file 'Sampling_order_310519.csv''.	00 sec	2.12.3.2
3	Transfer Volumetrically	Transfer liquid from variable 'Seq_Reactors' to variable 'Seq_Sample	00 sec	2.12.3.3
<insert sub tasks here>				
4	Set Drawer Valve	Set Reactionblock state to Closed Without Inert Gas on zone ISYNT	00 sec	2.12.4
5	Stir	Agitation ON on zone ISYNTH all	02 sec	2.12.5
<insert sub tasks here>				
13	Wait	Waiting for 2:00:00 hours	02 hours 00 min 00 sec	2.13
14	Sampling	Execute Once	04 sec	2.14
1	Stir	Agitation OFF on zone ISYNTH all	02 sec	2.14.1
2	Set Drawer Valve	Set Reactionblock state to Open Without Inert Gas on zone ISYNTH	00 sec	2.14.2
3	Macro Task	Execute Once	00 sec	2.14.3
1	Set Variable	Row := Row + 1	00 sec	2.14.3.1
2	Import CSV	import row 'Row' from file 'Sampling_order_310519.csv''.	00 sec	2.14.3.2
3	Transfer Volumetrically	Transfer liquid from variable 'Seq_Reactors' to variable 'Seq_Sample	00 sec	2.14.3.3
<insert sub tasks here>				
4	Set Drawer Valve	Set Reactionblock state to Closed Without Inert Gas on zone ISYNT	00 sec	2.14.4
5	Stir	Agitation ON on zone ISYNTH all	02 sec	2.14.5
<insert sub tasks here>				
15	Wait	Waiting for 4:00:00 hours	04 hours 00 min 00 sec	2.15
16	Sampling	Execute Once	04 sec	2.16
1	Stir	Agitation OFF on zone ISYNTH all	02 sec	2.16.1
2	Set Drawer Valve	Set Reactionblock state to Open Without Inert Gas on zone ISYNTH	00 sec	2.16.2
3	Macro Task	Execute Once	00 sec	2.16.3
1	Set Variable	Row := Row + 1	00 sec	2.16.3.1
2	Import CSV	import row 'Row' from file 'Sampling_order_310519.csv''.	00 sec	2.16.3.2
3	Transfer Volumetrically	Transfer liquid from variable 'Seq_Reactors' to variable 'Seq_Sample	00 sec	2.16.3.3
<insert sub tasks here>				
4	Set Drawer Valve	Set Reactionblock state to Closed Without Inert Gas on zone ISYNT	00 sec	2.16.4
5	Stir	Agitation ON on zone ISYNTH all	02 sec	2.16.5
<insert sub tasks here>				
17	Wait	Waiting for 16:00:00 hours	16 hours 00 min 00 sec	2.17
18	Sampling	Execute Once	04 sec	2.18
1	Stir	Agitation OFF on zone ISYNTH all	02 sec	2.18.1
2	Set Drawer Valve	Set Reactionblock state to Open Without Inert Gas on zone ISYNTH	00 sec	2.18.2
3	Macro Task	Execute Once	00 sec	2.18.3
1	Set Variable	Row := Row + 1	00 sec	2.18.3.1
2	Import CSV	import row 'Row' from file 'Sampling_order_310519.csv''.	00 sec	2.18.3.2
3	Transfer Volumetrically	Transfer liquid from variable 'Seq_Reactors' to variable 'Seq_Sample	00 sec	2.18.3.3
<insert sub tasks here>				
4	Set Drawer Valve	Set Reactionblock state to Closed Without Inert Gas on zone ISYNT	00 sec	2.18.4
5	Reflux	Reflux Off on zone ISYNTH all	02 sec	2.18.5
6	Heat / Cool	Thermostat OFF on zone ISYNTH all	00 sec	2.18.6
<insert sub tasks here>				
<insert sub tasks here>				

Supplementary Figure 5. Detailed Chemspeed tasks carried out for reactions run at 80 °C.

2.2 Initial Reaction Condition Screening

Reaction screening was performed using the following procedure and results are detailed in Supplementary Table 1.

$Pd(OAc)_2$ (5.6 mg, 0.025 mmol, 5 mol%), ligand (0.025 mmol, 5 mol%) and K_2CO_3 (138 mg, 1.0 mmol, 2.0 eq.) were placed in a Schlenk tube under N_2. DMF (1.5 mL) was added and the mixture was stirred at the desired temperature for 2 min. Then, a solution of 2-bromo-*N*-phenylbenzamide **1a** (138 mg, 0.5 mmol, 1.0 eq.) in DMF (1 mL) was added *via* cannula. The reaction mixture was stirred at the desired temperature for 2 h then allowed to cool to rt. Then, EtOAc (10 mL) was added and the resulting mixture was filtered through Celite and washed with EtOAc. The filtrate was washed with 1M $HCl_{(aq)}$ (10 mL) and brine (10 mL), dried over $MgSO_4$, filtered and the concentrated under reduced pressure to give a crude product which was subsequently purified by flash column chromatography (SiO_2, 9:1 to 8:2 petrol:EtOAc), to afford the *title compound* **2a** as a white solid.

Supplementary Table 1. Reaction condition screening. [a] Isolated yield after chromatography; [b] introduced to the reaction as the preformed catalyst $Pd_2dbaphos_2$; [c] 5 mL of air added.

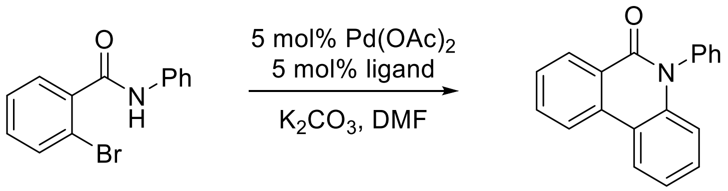

1a **2a**

Entry	Ligand	Temperature / °C	Time / h	Yield / %[a]
**1**	dppm	130	6	52
**2**	dppe	130	0.5	64
**3**	dppp	130	6	83
**4**	dppb	130	6	71
**5**	dppf	130	6	58
**6**	dbaphos[9]	130	5	0
**7**	dppe	80	6	54
**8**	dppe[c]	80	6	58

2.3 Synthesis and Characterization of Compounds

2-Bromo-*N*-phenylbenzamide 1a

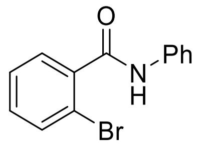

1a

Oxalyl chloride (6.3 mL, 74.6 mmol, 1.0 eq.) was added to a solution of 2-bromobenzoic acid (15.0 g, 74.6 mmol, 1.0 eq.) and DMF (2 drops) in CH_2Cl_2 (100 mL) at 0 °C. The solution was warmed to rt and stirred for 30 mins then concentrated under reduced pressure to provide the 2-bromobenzoyl chloride intermediate. A solution of 2-bromobenzoyl chloride (max. 74.6 mmol) in CH_2Cl_2 (50 mL) was added dropwise to a stirred solution of aniline **16a** (6.8 mL, 74.6 mmol, 1.0 eq.) and Et_3N (10.4 mL, 74.6 mmol, 1.0 eq.) in CH_2Cl_2 (60 mL) at 0 °C. The mixture was warmed to rt and stirred at rt for 18 h. Water (60 mL) was added, and the layers were separated. The aqueous layer was extracted with CH_2Cl_2 (2 × 60 mL). The combined organic layers were washed with water (60 mL) and brine (60 mL), dried ($MgSO_4$), filtered and concentrated under reduced pressure. The crude product was recrystallised from CH_2Cl_2 and hexane to give the title compound **1a** as a white solid (15.1 g, 73%).

MP: 119-120 °C (Lit. 122-123 °C[10]);

^{1}H NMR (400 MHz, $CDCl_3$): δ 7.84 (br s, 1H, NH), 7.66–7.57 (m, 4H, Ar), 7.37 (m, 3H, Ar), 7.30 (td, *J* = 7.5, 2.0 Hz, 1H, Ar), 7.20–7.14 (m, 1H, Ar);

^{13}C NMR (101 MHz, $CDCl_3$): δ 165.7 (C=O), 137.9 (*ipso*-Ar), 137.6 (*ipso*-Ar), 133.6 (Ar), 131.7 (Ar), 129.8 (Ar), 129.2 (Ar), 127.8 (Ar), 125.0 (Ar), 119.4 (*ipso*-Ar);

IR (ATR): 3250, 3191, 3131, 3057, 3019, 1654 (C=O), 1597, 1587, 1536, 1499, 1487, 1441, 1424, 1322, 1280, 1253, 1021, 745, 718, 687, 587, 511, 505, 452 cm^{-1};

HRMS (ESI): calc. for $C_{13}H_{11}{}^{79}BrNO$ $[M + H]^+$: 276.0019; found: 276.0018 (+0.1 ppm).

The analytical data obtained was in accordance with the literature.[11]

2-Bromo-*N*-[(2,3,4,5,6-$^2H_5$)phenyl]benzamide 1a-*d*$_5$

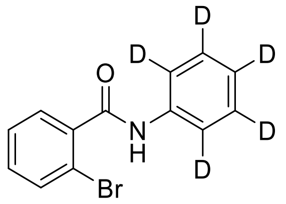

1a-*d*$_5$

Oxalyl chloride (0.38 mL, 3.0 mmol, 1.0 eq.) was added to a solution of 2-bromobenzoic acid (0.6 g, 3 mmol, 1.0 eq.) and DMF (1 drop) in CH_2Cl_2 (5 mL) at 0 °C. The solution was warmed to rt and stirred for 30 mins then concentrated under reduced pressure to provide the 2-bromobenzoyl chloride intermediate. A solution of 2-bromobenzoyl chloride (max. 3.0 mmol) in CH_2Cl_2 (5 mL) was added dropwise to a stirred solution of *d*$_5$-aniline (0.29 mL, 3.0 mmol, 1.0 eq.) and Et_3N (0.42 mL, 3.0 mmol, 1.0 eq.) in CH_2Cl_2 (5 mL) at 0 °C. The mixture was warmed to rt and stirred at rt for 2 h. Water (10 mL) was added, and the layers were separated. The aqueous layer was extracted with CH_2Cl_2 (2 × 20 mL). The combined organic layers were washed with water (20 mL) and brine (20 mL), dried ($MgSO_4$), filtered and concentrated under reduced pressure. The crude product was recrystallised from CH_2Cl_2 and hexane to give the title compound **1a-*d*$_5$** as a white solid (0.6 g, 72%).

MP: 118-120 °C;

^{1}H NMR (400 MHz, CDCl$_3$): δ 7.73 (s, 1H, NH), 7.65–7.61 (m, 2H, Ar), 7.40 (app. td, *J* = 7.5, 1.0 Hz, 1H, Ar), 7.32 (app. td, *J* = 7.5, 2.0 Hz, 1H, Ar);

^{13}C NMR (101 MHz, CDCl$_3$): δ 165.6 (C=O), 137.9 (*ipso*-Ar), 133.7 (*ipso*-Ar), 133.6 (Ar) 131.8 (Ar), 129.9 (Ar), 128.7 (t, *J* = 24.8 Hz, CD), 127.9 (Ar), 119.7 (t, *J* = 24.9 Hz, CD), 119.4 (*ipso*-Ar), one CD signal not resolved;

IR (ATR): 3260, 3160, 3088, 1639 (C=O), 1590, 1467, 1385, 1332, 1315, 1261, 1025, 889, 690, 555, 447cm^{-1};

HRMS (ESI): calc. for $C_{13}H_6D_5{}^{79}BrNO$ $[M + H]^+$: 281.1332; found: 281.0320 (+4.1 ppm).

2-Bromo-4-methyl-*N*-phenylbenzamide 1b

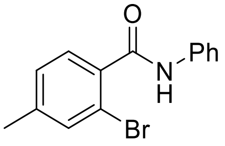

1b

Oxalyl chloride (78 µL, 0.92 mmol, 1.0 eq.) was added to a solution of 2-bromo-4-methylbenzoic acid (200 mg, 0.92 mmol, 1.0 eq.) and DMF (1 drop) in CH_2Cl_2 (5 mL) at 0 °C. The solution was warmed to rt and stirred for 30 mins then concentrated under reduced pressure to provide the 2-bromo-4-methylbenzoyl chloride intermediate. A solution of 2-bromo-4-methylbenzoyl chloride (max. 0.92 mmol) in CH_2Cl_2 (5 mL) was added dropwise to a stirred solution of aniline **16a** (84 µL, 0.92 mmol, 1.0 eq.) and Et_3N (128 µL, 0.92 mmol, 1.0 eq.) in CH_2Cl_2 (6 mL) at 0 °C. The mixture was warmed to rt and stirred at rt for 2 h. Water (10 mL) was added, and the layers were separated. The aqueous layer was extracted with CH_2Cl_2 (2 × 10 mL). The combined organic layers were washed with water (10 mL) and brine (10 mL), dried ($MgSO_4$), filtered and concentrated under reduced pressure. The crude product was recrystallised from CH_2Cl_2 and hexane to give the title compound **1b** as a white solid (203 mg, 76%).

MP: 145-146 °C;

^{1}H NMR (400 MHz, CDCl$_3$): δ 7.86 (br s, 1H, NH), 7.63 (d, *J* = 8.0 Hz, 2H, Ar), 7.54–7.50 (m, 1H, Ar), 7.44 (s, 1H, Ar), 7.36 (app. t, *J* = 8.0 Hz, 2H, Ar), 7.20–7.12 (m, 2H, Ar), 2.36 (s, 3H, Me);

^{13}C NMR (101 MHz, CDCl$_3$): δ 165.7 (C=O), 142.5 (*ipso*-Ar), 137.8 (*ipso*-Ar), 134.8 (*ipso*-Ar), 134.0 (Ar), 129.9 (Ar), 129.2 (Ar), 128.6 (Ar), 124.8 (Ar), 120.1 (Ar), 119.2 (*ipso*-Ar), 21.1 (Me);

IR (ATR): 3238, 1645 (C=O), 1596, 1541, 1490, 1442, 1327, 1281, 1259, 1143, 1039, 926, 827, 755, 693, 586, 512, 420 cm^{-1};

HRMS (ESI): calc. for $C_{14}H_{12}{}^{79}BrNNaO$ $[M + Na]^+$: 311.9994; found 311.9977 (+4.9 ppm);

CHN: calc. for $C_{14}H_{12}BrNO$: C 57.95, H 4.17, N 4.83; found C 57.38, H 4.14, N 4.74.

5-Phenyl-5,6-dihydrophenanthridin-6-one 2a

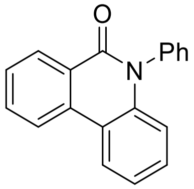

2a

$Pd(OAc)_2$ (5.6 mg, 0.025 mmol, 5 mol%), dppe (10.0 mg, 0.025 mmol, 5 mol%) and K_2CO_3 (138 mg, 1.0 mmol, 2.0 eq.) were placed in a Schlenk tube under N_2. DMF (1.5 mL) was added, and the mixture was stirred at 80 °C for 2 min. Then, a solution of 2-bromo-N-phenylbenzamide **1a** (138 mg, 0.5 mmol, 1.0 eq.) in DMF (1 mL) was added via cannula. [Air was injected into the reaction mixture via syringe (5 mL) – to assess the impact of trace air in the reaction systems]. The reaction mixture was then stirred at 80 °C for 6 h then allowed to cool to room temperature (~22 ˚C). Then, EtOAc (10 mL) was added, and the resulting mixture was filtered through Celite and washed with EtOAc (3x5 mL). The combined organic extracts were was washed with 1M HCl(aq) (10 mL) and brine (10 mL), dried over $MgSO_4$, filtered and the concentrated under reduced pressure to give a crude product which was subsequently purified by flash column chromatography (SiO_2, 9:1 to 8:2 petrol:EtOAc), to afford compound **2a** as a white solid (39 mg, 58%).

***R*f:** 0.33 (petrol/EtOAc, 7:3);

MP: 228-230 °C (lit. 227-229 °C[12]);

^{1}H NMR (400 MHz, $CDCl_3$): δ 8.58 (dd, *J* = 8.0, 1.5 Hz, 1H, Ar), 8.35 (d, *J* = 8.5 Hz, 1H, Ar), 8.33–8.29 (m, 1H, Ar), 7.82 (ddd, *J* = 8.0, 7.5, 1.5 Hz, 1H, Ar), 7.66–7.60 (m, 3H, Ar), 7.55 (tt, *J* = 8.5, 1.5 Hz, 1H, Ar), 7.36–7.27 (m, 4H, Ar), 6.74–6.67 (m, 1H, Ar);

^{13}C NMR (101 MHz, $CDCl_3$): δ 161.7 (C=O), 139.1 (*ipso*-Ar), 138.3 (*ipso*-Ar), 134.0 (*ipso*-Ar), 132.8 (Ar), 130.2 (Ar), 129.1 (Ar), 129.0 (Ar), 128.7 (Ar), 128.1 (Ar), 125.8 (*ipso*-Ar), 123.0 (Ar), 122.6 (Ar), 121.8 (Ar), 119.0 (*ipso*-Ar), 117.0 (Ar);

IR (ATR): 3059, 1651 (C=O), 1603, 1485, 1430, 1320, 1290, 1040, 801, 745, 686, 644, 512 cm^{-1};

HRMS (ESI): calc. for $C_{19}H_{14}NO$ $[M + H]^+$: 272.1070; found 272.1074 (–1.0 ppm);
The analytical data obtained was in accordance with the literature.[12]

An X-ray diffraction crystal structure of this compound was obtained (CCDC 2063167), see below:

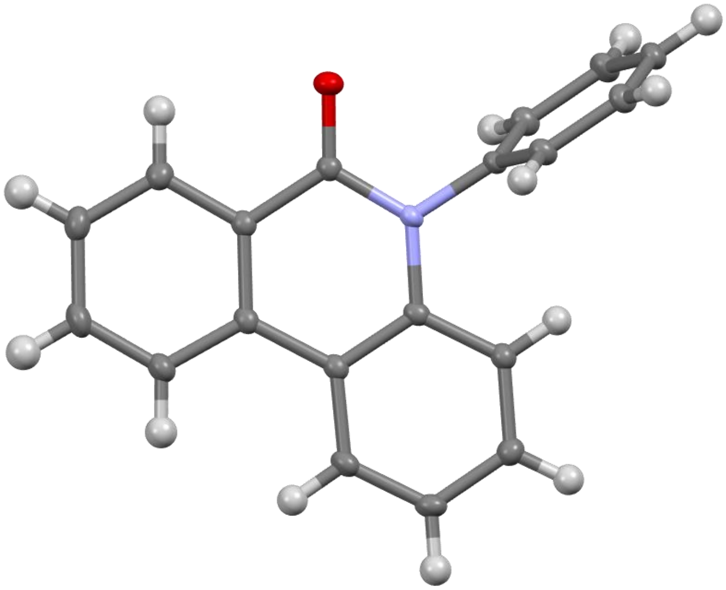

5-[(2,3,4,5,6-$^2H_5$)Phenyl]-5,6-dihydrophenanthridin-6-one 2a-*d_5*

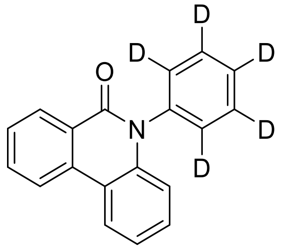

2a-*d_5*

$Pd(OAc)_2$ (7 mg, 0.031 mmol, 5 mol%), dppe (12.5 mg, 0.031 mmol, 5 mol%) and K_2CO_3 (173 mg, 1.25 mmol, 2.0 eq.) were placed in a Schlenk tube under N_2. DMF (7.5 mL) was added, and the mixture was stirred at 130 °C for 2 min. Then, 2-bromo-*N*-[(2,3,4,5,6-$^2H_5$)phenyl]benzamide **1a-*d_5*** (173 mg, 0.63 mmol, 1.0 eq.) was added. The reaction mixture was stirred at 130 °C for 2 h then allowed to cool to rt. Then, EtOAc (20 mL) was added, and the resulting mixture was filtered through Celite and washed with EtOAc. The filtrate was washed with 1M $HCl_{(aq)}$ (20 mL) and brine (20 mL), dried over $MgSO_4$, filtered and the concentrated under reduced pressure to give a crude product which was subsequently purified by flash column chromatography (SiO_2, 9:1 to 8:2 petrol:EtOAc), to afford the *title compound* **2a-*d_5*** as a white solid (52 mg, 61%).

***R_f*:** 0.33 (petrol/EtOAc, 7:3);

^{1}H NMR (400 MHz, $CDCl_3$): δ 8.58 (dd, *J* =8.0, 1.5 Hz, 1H, Ar), 8.35 (d, *J* = 8.5 Hz, 1H, Ar), 8.33–8.28 (m, 1H, Ar), 7.82 (ddd, *J* = 8.0, 7.0, 1.5 Hz, 1H, Ar), 7.62 (ddd, *J* = 8.0, 7.5, 1.0 Hz, 1H, Ar), 7.32–7.25 (m, 2H, Ar), 6.73–6.68 (m, 1H, Ar);

^{13}C NMR (101 MHz, $CDCl_3$): δ 161.7 (C=O), 139.1 (*ipso*-Ar), 138.1 (*ipso*-Ar), 134.0 (*ipso*-Ar), 132.8 (Ar), 129.7 (t, *J* = 24.4 Hz, CD), 129.1 (Ar), 129.0 (Ar), 128.6 (t, *J* = 24.4 Hz), 128.2 (t, *J* = 24.4 Hz), 128.1 (Ar), 125.8, 123.0 (Ar), 122.6 (Ar), 121.8 (Ar), 119.0 (*ipso*-Ar), 117.0 (Ar);

IR (thin film): 3072, 1655 (C=O), 1608, 1588, 1486, 1435, 1391, 1331, 1316, 1267, 747, 724, 549 cm^{-1};

HRMS (ESI): calc. for $C_{19}H_8D_5NO$ $[M + H]^+$: 277.1384; found 277.1377 (+2.4 ppm).

2,9-Dimethyl-5-phenyl-5,6-dihydrophenanthridin-6-one 2b

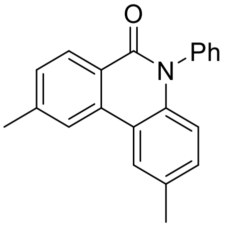

2b

$Pd(OAc)_2$ (14 mg, 0.063 mmol, 5 mol%), dppe (25 mg, 0.063 mmol, 5 mol%) and K_2CO_3 (345 mg, 2.5 mmol, 2.0 eq.) were placed in a Schlenk tube under N_2. DMF (15 mL) was added, and the mixture was stirred at 80 °C for 2 min. Then, 2-bromo-4-methyl-*N*-phenyl benzamide **1b** (363 mg, 1.25 mmol, 1.0 eq.) was added. The reaction mixture was stirred at 80 °C for 2 h then allowed to cool to rt. Then, EtOAc (40 mL) was added, and the resulting mixture was filtered through Celite and washed with EtOAc. The filtrate was washed with 1M $HCl_{(aq)}$ (40 mL) and brine (40 mL), dried over $MgSO_4$, filtered and the concentrated under reduced pressure to give a crude product which was subsequently purified by flash column chromatography (SiO_2, 9:1 to 8:2 petrol:EtOAc), to afford the *title compound* **2b** as a white solid (73 mg, 39%).

***R*f:** 0.47 (petrol/EtOAc, 4:1);

MP: 170-171 °C;

^{1}H NMR (500 MHz, $CDCl_3$): δ 8.44 (d, *J* = 8.0 Hz, 1H, Ar), 8.12 (d, *J* = 0.5 Hz, 1H, Ar), 8.09 (d, *J* = 1.5 Hz, 1H, Ar), 7.62–7.58 (m, 2H, Ar), 7.52 (ddt, *J* = 8.5, 7.0, 1.5 Hz, 1H, Ar), 7.42 (ddd, *J* = 8.0, 1.5, 0.5 Hz, 1H, Ar), 7.34–7.31 (m, 2H, Ar), 7.10 (ddd, *J* = 8.5, 2.0, 0.5 Hz, 1H, Ar), 6.58 (d, *J* = 8.5 Hz, 1H, Ar), 2.60 (s, 3H, Me), 2.46 (s, 3H, Me);

^{13}C NMR (101 MHz, $CDCl_3$): δ 161.7 (C=O), 143.3 (Ar), 138.6 (Ar), 137.4 (Ar), 134.0 (Ar), 132.1 (Ar), 130.2 (Ar), 130.1 (Ar), 129.5 (Ar), 129.3 (Ar), 129.1 (Ar), 128.7 (Ar), 123.8 (Ar), 123.1 (Ar), 121.9 (Ar), 118.9 (Ar), 117.0, 22.3 (Me), 21.1 (Me);

IR (thin film): 3061, 2920, 2241, 1651 (C=O), 1616, 1582, 1508, 1492, 1453, 1339, 1308, 732, 694 cm^{-1};

HRMS (ESI): calc. for $C_{21}H_{18}NO$ $[M + H]^+$: 300.1383; found 300.1379 (+0.7 ppm);

CHN: calc. for $C_{21}H_{17}NO$: C 84.25, H 5.72, N 4.68; found C 84.13, H 5.80, N 4.69.

An X-ray diffraction crystal structure of this compound was obtained (CCDC 2063164), see below:

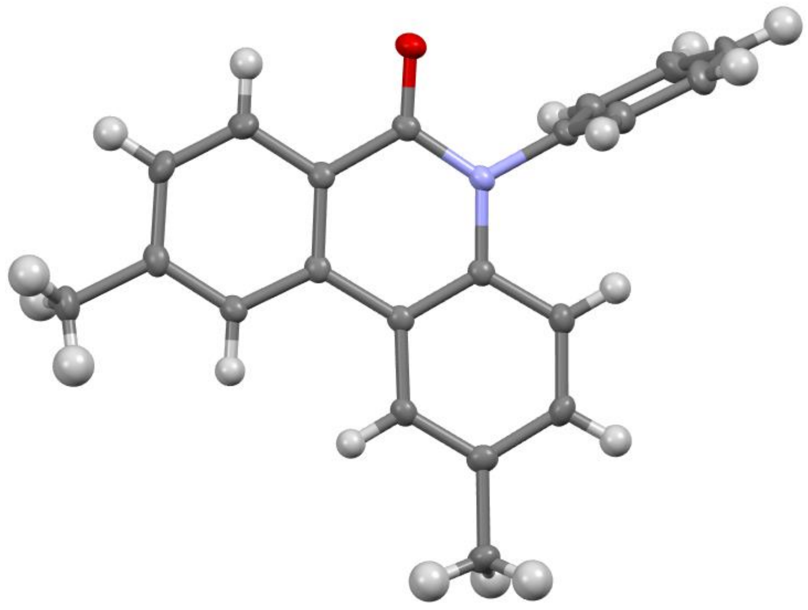

2.4. *In Operando* Monitoring of the Reaction of 1a in DMF at 80 °C using IR Spectroscopic Analysis

Reaction profiles shown in Figure 2 were monitored by *in operando* IR analysis, monitoring a clear stretching band for **1a** at 1324 cm^{-1}. *In situ* IR spectroscopic measurements were made on a Mettler Toledo ReactIR ic10 with a K6 conduit SiComp (silicon) probe and MCT detector. An oven dried three-necked round-bottom flask equipped with a magnetic stirrer bar was attached to the ReactIR ic10 probe. One neck was sealed with a septum, and the other connected to a Schlenk line. The system was evacuated and subsequently backfilled with N_2 five times. Following this a background spectrum was recorded under an N_2 atmosphere. An internal thermocouple and dry deoxygenated solvent were introduced *via* the septum. The system was heated using a stirrer hotplate until thermocouple was giving steady readings at the desired temperature. Sample measurements were then started at 30 second intervals, and reagents added in a sequential order. Liquids were injected through a rubber septum, whereas solids required rapid removal of the rubber septum under a slight positive flow of N_2, addition, and replacement of the septum. After each addition, a comment was added to the experimental run; 5 minutes were given for the IR signal/ reaction temperature to stabilize.

Cold System Experimental Procedure:

K_2CO_3 (414 mg, 3.0 mmol, 2.0 eq.) and 2-bromo-*N*-phenylbenzamide **1a** (414 mg, 1.5 mmol, 1.0 eq.) were added to dry, degassed DMF (14 mL) at 80 °C under N_2, in a three-neck round bottom flask attached to Metler Mettler Toledo ReactIR ic10 with a K6 conduit and silicon probe. After the addition of each material, the solution was allowed to equilibrate for 5 minutes. A solution of $Pd(OAc)_2$ (16.8 mg, 0.075 mmol, 5 mol%) and dppe (30 mg, 0.075 mmol, 5 mol%) in DMF (3.0 mL) – *done at 20 °C and stirred for 2 minutes* – was added to the reaction. The reaction was stirred at 80 °C for 180 minutes. An aliquot (0.2 mL) was removed at 180 minutes, which was filtered through Celite™ with EtOAc (1 mL), and washed with 2M $HCl_{(aq)}$ (1 mL) and brine (1 mL), before being analyzed by ^1H NMR.

Pre-Heated System Experimental Procedure:

K_2CO_3 (414 mg, 3.0 mmol, 2.0 eq.) and 2-bromo-*N*-phenylbenzamide **1a** (414 mg, 1.5 mmol, 1.0 eq.) were added to dry, degassed DMF (14 mL) at 80 °C under N_2, in a three-neck round bottom flask attached to Metler Mettler Toledo ReactIR ic10 with a K6 conduit and silicon probe. After the addition of each material, the solution was allowed to equilibrate for 5 minutes. A solution of $Pd(OAc)_2$ (16.8 mg, 0.075 mmol, 5 mol%) and dppe (30 mg, 0.075 mmol, 5 mol%) in DMF (3.0 mL) – *done at 80 °C and stirred for 2 minutes* – was added to the reaction. The reaction was stirred at 80 °C for 180 minutes. An aliquot (0.2 mL) was removed 180 minutes. The aliquot was filtered through Celite™ with EtOAc (1 mL), and washed with 2M $HCl_{(aq)}$ (1 mL) and brine (1 mL), before being analysed by ^1H NMR.

2.5 Exemplar LC-MS traces and Retention times of Compounds of Interest

Supplementary Figure 6 shows an exemplar chromatogram, with some compounds of interest labelled (not all compounds of interest were observed in every chromatogram). Supplementary Table 2 details the peaks of interest, ordered by compound number. Section 2.6 below details the characterization of these species.

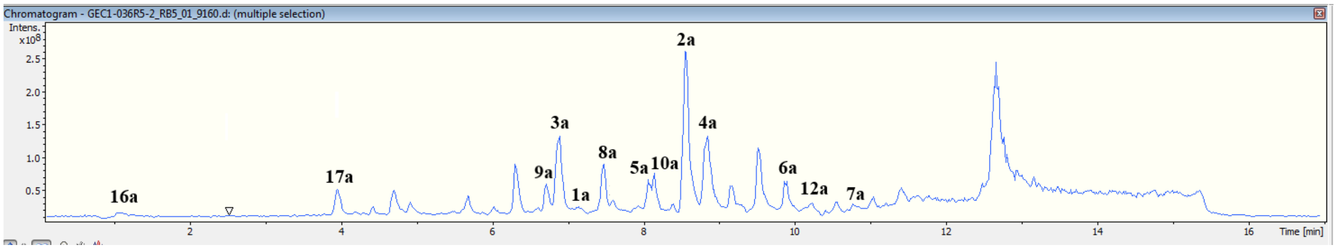

Supplementary Figure 6. Exemplar chromatogram. Reaction conditions: **1a** with 5 mol% $Pd(OAc)_2$, 5 mol% dppe and 2.0 eq. K_2CO_3 in MeCN at 130 °C for 2 h.

Supplementary Table 2. Compounds of interest.

Structure	Compound Number	Molecular Formula	Monoisotopic Mass	LC-MS Retention Time / min
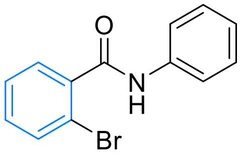	**1a**	$C_{13}H_{10}BrNO$	274.99 / 276.99	7.1-7.2
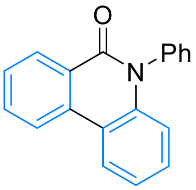	**2a**	$C_{19}H_{13}NO$	271	8.5-8.7
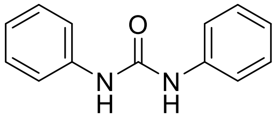	**3a**	$C_{13}H_{12}N_2O$	212	6.9-7.0
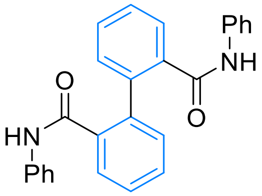	**4a**	$C_{26}H_{20}N_2O_2$	392	8.8-9.1

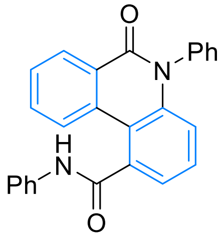	**5a**	$C_{26}H_{18}N_2O_2$	390	8.0-8.2
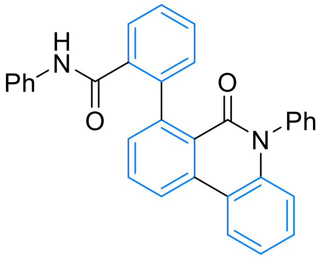	**6a**	$C_{32}H_{22}N_2O_2$	466	9.9-10.1
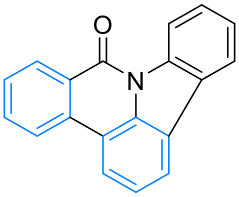	**7a**	$C_{19}H_{11}NO$	269	10.8-10.9
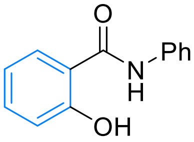	**8a**	$C_{13}H_{11}NO_2$	213	7.4
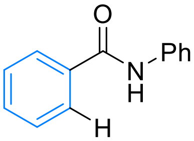	**9a**	$C_{13}H_{11}NO$	197	6.7-6.8
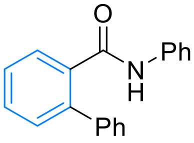	**10a**	$C_{19}H_{15}NO$	273	8.1-8.2
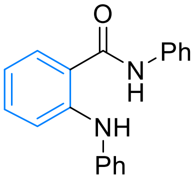	**11a**	$C_{19}H_{16}N_2O$	288	9.4
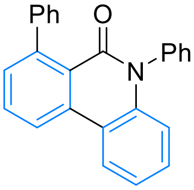	**12a**	$C_{25}H_{17}NO$	347	10.0-10.5

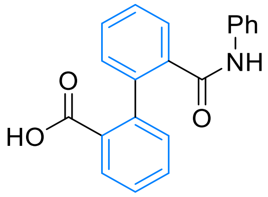	**13a**	$C_{20}H_{15}NO_3$	317	6.5
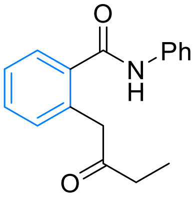	**14a**	$C_{17}H_{17}NO_2$	267	7.2
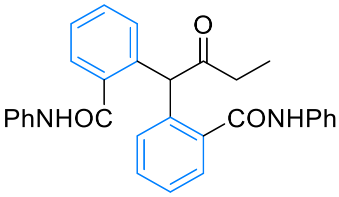	**15a**	$C_{30}H_{26}N_2O_3$	462	9.1
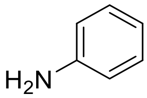	**16a**	C_6H_7N	93	1.0-1.3
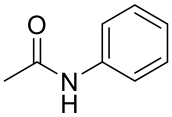	**17a**	C_8H_9NO	135	3.8-4.1
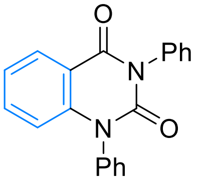	**18a**	$C_{20}H_{14}N_2O_2$	314	9.7-10.0
Not assigned *(treated as an unknown material)*	**19a**		468	9.3-9.4

2.6 Identification of By-Products and Side-Products

Characterization of the above by-products and side-products was achieved through performing LC-MS, GC-MS, flash column chromatography and preparative HPLC on the crude reaction mixtures and comparing the chromatographic, MS and/or ^{1}H and ^{13}C NMR data with authentic samples.

2.6.1 Isolation and Characterization of Side-Products from Large Scale Reactions of 1a, 1a-d_5 and 1b

Two larger scale reactions of **1a** were performed (*vide infra*) and some side products were isolated by flash column chromatography. Analogous reactions were performed with deuterated and methylated bromo benzamides **1a-d_5** and **1b** (see section 2.3). The following side-products were isolated from these three reactions by flash column chromatography (Supplementary Figure 7).

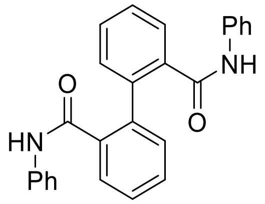

4a

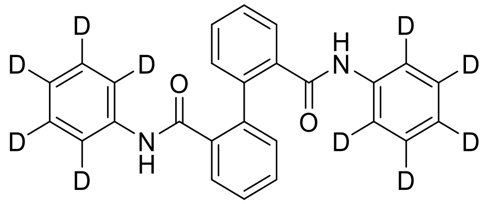

4a-d_{10}

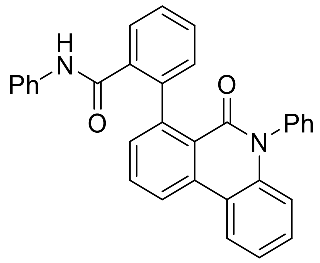

6a

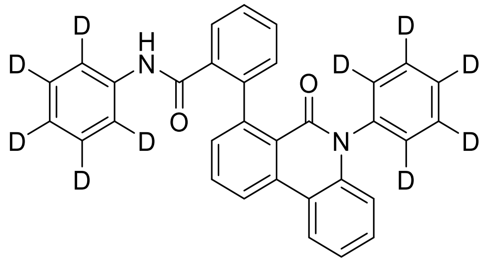

6a-d_{10}

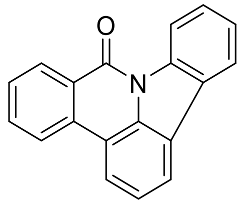

7a

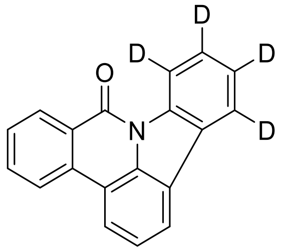

7a-d_4

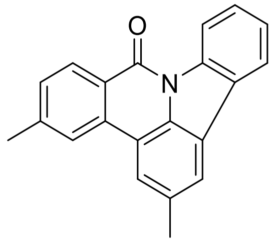

7b

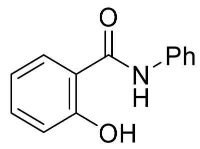

8a

Supplementary Figure 7. Side-products isolated from large scale reactions of **1a**, **1a-d_5** and **1b**.

Experimental Procedure for larger-scale reaction of 1a using dppe ligand in DMF:

A Schlenk tube was charged with $Pd(OAc)_2$ (14 mg, 0.063 mmol, 5 mol%), dppe (25 mg, 0.063 mmol, 5 mol%) and K_2CO_3 (345 mg, 2.5 mmol, 2.0 eq.) under N_2. DMF (15 mL) was added, and the mixture was stirred at 80 °C for 2 min. Then, 2-bromo-*N*-phenylbenzamide **1a** (345 mg, 1.25 mmol, 1.0 eq.) was added. The reaction mixture was stirred at 80 °C for 2 h then allowed to cool to rt. Then, EtOAc (40 mL) was added, and the resulting mixture was filtered through Celite and washed with EtOAc. The filtrate was washed with 1M $HCl_{(aq)}$ (20 mL) and brine (20 mL), dried over $MgSO_4$, filtered and the concentrated under reduced pressure to give a crude product which was subsequently purified by flash column chromatography (SiO_2, 9:1 to 55:45 petrol:EtOAc), to afford the *title compound* **2a** as a white solid (17 mg, 28%) and **4a** (31 mg isolated as a mixture), **7a** as a white solid (3.7 mg, 2.2%) and **8a** (5 mg as a mixture).

Experimental Procedure for larger-scale reaction of 1a using dppp ligand in DMA:

A Schlenk tube was charged with $Pd(OAc)_2$ (30 mg, 0.136 mmol, 5 mol%), dppp (56 mg, 0.136 mmol, 5 mol%) and K_2CO_3 (750 mg, 5.42 mmol, 2.0 eq.) under N_2. A second Schlenk tube was charged with 2-bromo-*N*-phenylbenzamide **1a** (750 mg, 2.72 mmol, 1.0 eq.). DMA (8 mL) was added to the first tube and DMA (3.5 mL) was added to the second tube. The catalyst mixture was heated to 130 °C for 2 min before the solution of **1a** at 130 °C was transferred into the catalyst mixture via cannula. The resulting mixture was stirred at 130 °C for 2 h then allowed to cool to rt. Then, EtOAc (50 mL) was added, and the resulting mixture was filtered through Celite and washed with EtOAc. The filtrate was washed with 1M $HCl_{(aq)}$ (20 mL) and brine (20 mL), dried over $MgSO_4$, filtered and the concentrated under reduced pressure to give a crude product as a yellow solid. Purification by flash column chromatography (SiO_2, 9:1 to 55:45 petrol:EtOAc), to afford the *title compounds* **2a** as a white solid (117 mg, 32%), **6a** as a yellow solid (5.3 mg, 1.8%) and **7a** as a white solid (76 mg, 21%).

N2,N2'-Diphenyl-[1,1'-biphenyl]-2,2'-dicarboxamide 4a

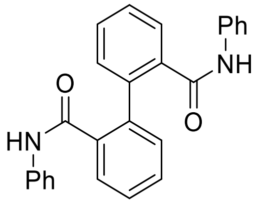

4a

31 mg isolated as a mixture with unknown compounds. Identity confirmed through comparison with literature spectral data,[13] through an X-ray diffraction crystal structure and through independent synthesis and chromatographic studies (see section 2.8).

***R*f:** 0.16 (petrol/EtOAc, 7:3);

^{1}H NMR (400 MHz, $CDCl_3$): δ 9.22 (s, 2H, NH), 7.67–7.61 (m, 2H, Ar), 7.42 (app. d, J = 8.5 Hz, 4H, Ar), 7.40–7.27 (m, 4H, Ar), 7.24–7.21 (m, 4H, Ar), 7.09–7.03 (m, 4H, Ar);

^{13}C NMR (101 MHz, $CDCl_3$): δ 168.5 (C=O), 139.2 (Ar), 138.1 (Ar), 136.2 (Ar), 130.2 (Ar), 129.8 (Ar), 129.0 (Ar), 128.1 (Ar), 127.4 (Ar), 124.5 (Ar), 120.1 (Ar);

HRMS (ESI): calc. for $C_{26}H_{21}N_2O_2$ $[M + H]^+$: 393.1598; found 393.1594 (+1.1 ppm).

An X-ray diffraction crystal structure of this compound was obtained (CCDC 2063166), see below:

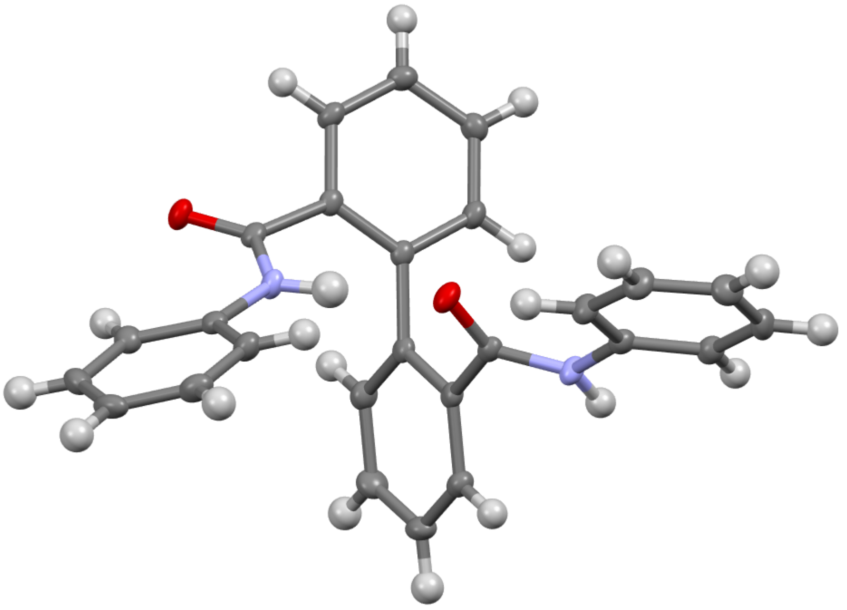

N2,N2'-bis[(2,3,4,5,6-$^2H_5$)Phenyl]-[1,1'-biphenyl]-2,2'-dicarboxamide 4a-d_{10}

16 mg isolated as a mixture with unknown compounds.

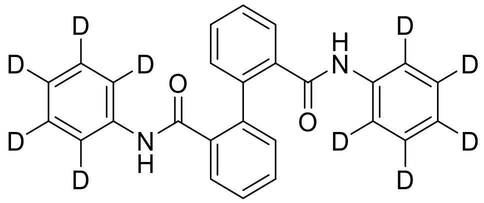

4a-d_{10}

Selected Data:

***R*f:** 0.14 (petrol/EtOAc, 7:3);

^{1}H NMR (400 MHz, CDCl$_3$): δ 9.14 (br s, 2H, NH), 7.71–7.62 (m, 2H, Ar), 7.40–7.33 (m, 4H, Ar), 7.14–7.10 (m, 2H);

HRMS (ESI): calc. for $C_{26}H_{11}D_{10}N_2O_2$ [M + H]$^+$: 403.2225; found 403.2225 (+0.5 ppm).

2-(6-Oxo-5-phenyl-5,6-dihydrophenanthridin-7-yl)-*N*-phenylbenzamide 6a

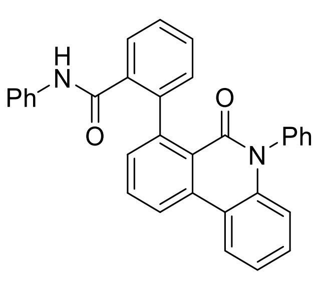

6a

Stucture confirmed by independent synthesis (see section 2.8) and by analogy with related compounds.[14]

Selected Data:

***R*f:** 0.29 (petrol/EtOAc, 7:3);

^{1}H NMR (400 MHz, CDCl$_3$): δ 8.97 (s, 1H, NH), 8.38–8.31 (m, 2H, Ar), 7.78–7.74 (m, 1H, Ar), 7.67–7.49 (m, 4H, Ar), 7.41–7.30 (m, 6H, Ar), 7.26–7.23 (m, 2H, Ar), 7.18–7.14 (m, 2H, Ar), 7.11–7.09 (m, 1H, Ar), 7.06–7.03 (m, 1H, Ar), 6.97–6.93 (m, 1H, Ar), 6.63–6.67 (1H, m, Ar);

MS (ESI): [M + H]$^+$: 467.2; [M + Na]$^+$: 489.2.

2-{6-Oxo-5-[(2,3,4,5,6-$^2H_5$)phenyl]-5,6-dihydrophenanthridin-7-yl}-*N*-[(2,3,4,5,6-$^2H_5$)phenyl]benzamide 6a-*d*$_{10}$

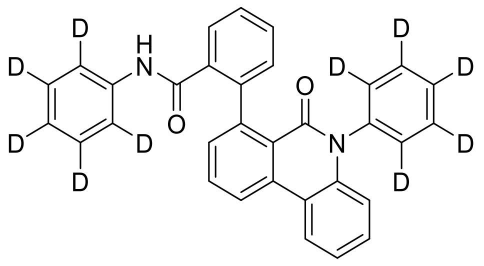

6a-*d*$_{10}$

8 mg isolated as a mixture. Stucture confirmed by analogy with related compounds.[14]

Selected Data:

***R*f:** 0.29 (petrol/EtOAc, 7:3);

^{1}H NMR (400 MHz, CDCl3): δ 8.39–8.32 (m, 1H, NH), 7.75 (dd, *J* = 8.0, 7.5 Hz, 1H, Ar), 7.71–7.68 (m, 1H, Ar), 7.65–7.61 (m, 1H, Ar), 7.42–7.35 (m, 3H), 7.34–7.31 (m, 2H, Ar), 7.22–7.18 (m, 1H, Ar), 7.08–7.04 (m, 1H, Ar), 6.67–6.61 (m, 1H, Ar);

HRMS (ESI): calc. for $C_{32}H_{13}D_{10}N_2O_2$ [M + H]$^+$: 477.2387; found 477.2396 (+1.9 ppm).

1-Azapentacyclo[10.7.1.0^{2,7}.0^{8,20}.0^{13,18}]icosa-2(7),3,5,8,10,12(20),13,15,17-nonaen-19-one 7a

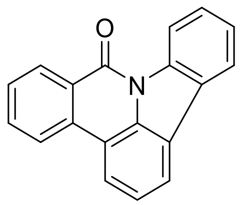

7a

Isolated as a white solid. Identity confirmed through comparison with literature spectral data,[15] through independent synthesis (see section 2.8), and through an X-ray diffraction crystal structure.

***R*f:** 0.58 (petrol/EtOAc, 7:3);

^{1}H NMR (400 MHz, CDCl3): δ 8.86 (dt, *J* = 8.0, 1.0 Hz, 1H, Ar), 8.71 (ddd, *J* = 8.0, 1.5, 0.5 Hz, 1H, Ar), 8.39–8.33 (m, 1H, Ar), 8.21 (dd, *J* = 8.0, 1.0 Hz, 1H, Ar), 8.14–8.07 (m, 2H, Ar), 7.89–7.81 (m, 1H, Ar), 7.68 (ddd, *J* = 8.0, 7.0, 1.0 Hz, 1H, Ar), 7.65–7.59 (m, 2H, Ar), 7.51 (td, *J* = 7.5, 1.0 Hz, 1H, Ar);

^{13}C NMR (101 MHz, CDCl3): δ 160.0 (C=O), 138.6 (Ar), 133.9 (Ar), 133.0 (Ar), 129.4 (Ar), 128.4 (Ar), 128.1 (Ar), 127.8 (Ar), 126.4 (Ar), 125.0 (Ar), 124.5 (Ar), 124.1 (Ar), 122.5 (Ar), 121.1 (Ar), 120.8 (Ar), 120.3 (Ar), 117.3 (Ar), 117.2 (Ar), one signal not resolved;

IR (thin film): 3303, 2923, 2853, 1664 (C=O), 1597, 1516, 1444, 1421, 1351, 1339, 1305, 1272, 760, 733, 688, 674, 479 cm^{-1};

HRMS (ESI): calc. for $C_{19}H_{12}NO$ [M + H]$^+$: 270.0913; found 270.0925 (–4.8 ppm).

An X-ray diffraction crystal structure of this compound was obtained (CCDC 2063165), see below:

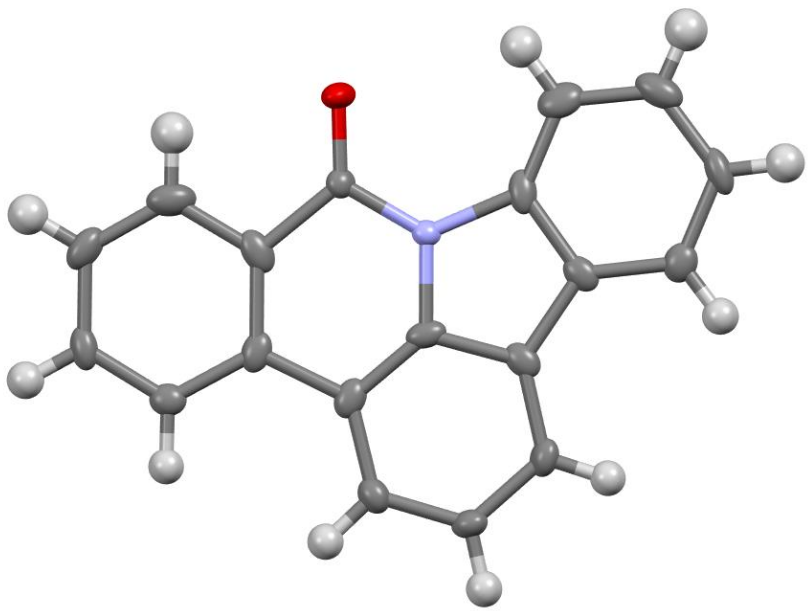

(3,4,5,6-$^{2}H_4$)-1-Azapentacyclo[10.7.1.$0^{2,7}$.$0^{8,20}$.$0^{13,18}$]icosa-2(7),3,5,8,10,12(20),13,15,17-nonaen-19-one 7a-d$_4$

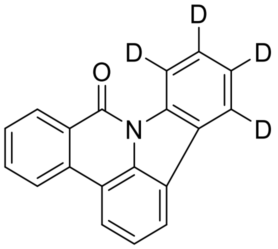

7a-d$_4$

1.8 mg (2% yield), isolated as a white solid.

Selected Data:

***R*$_f$:** 0.46 (petrol/EtOAc, 7:3);

^{1}H NMR (400 MHz, CDCl$_3$): δ 8.72 (dd, *J* = 8.0, 1.5 Hz, 1H, Ar), 8.38 (d, *J* = 7.5 Hz, 1H, Ar), 8.23 (d, *J* = 8.0 Hz, 1H, Ar), 8.13 (dd, *J* = 7.5, 1.0 Hz, 1H, Ar), 7.89–7.83 (m, 1H, Ar), 7.69 (ddd, *J* = 8.0, 7.0, 1.5 Hz, 1H), 7.63 (t, J = 7.5 Hz, 1H, Ar);

IR (thin film): 2958, 2923, 2854, 1667 (C=O), 1461, 1384, 1305, 1265, 769 cm^{-1};

HRMS (ESI): calc. for $C_{19}H_7D_4NNaO$ [M + Na]$^+$: requires 296.0984; found 296.0990 (–1.4 ppm).

10,15-Dimethyl-1-azapentacyclo[10.7.1.$0^{2,7}$.$0^{8,20}$.$0^{13,18}$]icosa-2(7),3,5,8,10,12(20),13,15,17-nonaen-19-one 7b

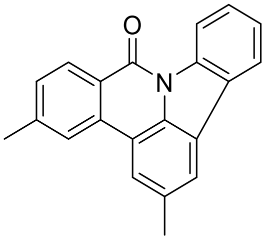

7b

2.3 mg (1.2% yield), isolated as a white solid.

Selected Data:

***R*f:** 0.67 (petrol/EtOAc, 4:1);

^{1}H NMR (400 MHz, CDCl$_3$): δ 8.81 (dd, *J* = 8.0, 1.0 Hz, 1H, Ar), 8.56 (d, *J* = 8.0 Hz, 1H, Ar), 8.10 (s, 1H, Ar), 8.04 (dd, *J* = 7.5, 1.0 Hz, 1H, Ar), 7.99 (s, 1H, Ar), 7.89 (s, 1H, Ar), 7.62–7.55 (m, 1H, Ar), 7.50–7.43 (m, 1H, Ar), 2.67 (s, 3H, Me), 2.60 (s, 3H, Me);

IR (thin film): 2961, 2924, 2854, 1678 (C=O), 1617, 1351, 1133, 782, 754 cm^{-1};

HRMS (ESI): calc. for $C_{21}H_{15}NNaO$ [M + Na]$^+$: requires 320.1046; found 230.1032 (+3.8 ppm).

2-Hydroxy-*N*-phenylbenzamide 8a

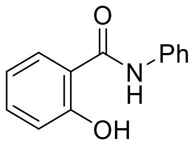

8a

5 mg isolated as a mixture. Identity confirmed through comparison with literature spectral data,[16] and through chromatographic studies (see section 2.6.2).

Selected Data:

***R*f:** 0.45 (petrol/EtOAc, 7:3);

^{1}H NMR (400 MHz, CDCl$_3$): δ 11.99 (s, 1H, OH), 7.95 (br s, 1H, NH), 7.58 (d, *J* = 8.3 Hz, 2H, Ar), 7.58 (d, *J* = 8.5 Hz, 2H, Ar), 7.53 (d, *J* = 8.5 Hz, 1H, Ar), 7.51–7.43 (m, 1H, Ar), 7.40 (app. t, *J* = 7.5 Hz, 2H, Ar), 7.24–7.18 (m, 1H, Ar), 7.04 (d, *J* = 8.5 Hz, 1H, Ar), 6.98–6.89 (m, 1H, Ar);

HRMS (ESI): calc. for $C_{19}H_{15}NNaO$ [M + Na]$^+$: 296.1046; found 296.1044 (–0.6 ppm).

2.6.2 Observation by HRMS From Large Scale Reactions of 1a, 1a-*d*5 and 1b

Column fractions from the reactions of **1a**, **1a-*d*5** and **1b** detailed in section 2.6.1 contained the following species by HRMS (Supplementary Figure 8). The presence of compounds **8a**, **9a** and **10a** were confirmed through separate chromatographic studies in comparison with authetic samples (see section 2.6.4). Furthermore, **8a** was also identified by ^{1}H NMR of the mixed fraction (see see section 2.6.1).

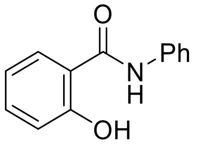

8a
MNa$^+$ 236

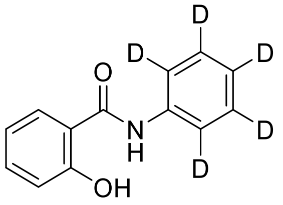

8a-*d*5
Not observed

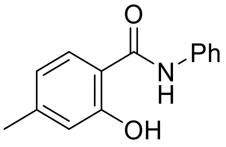

8b
MNa$^+$ 250

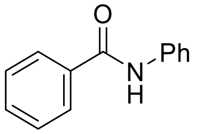

9a
MNa$^+$ 220

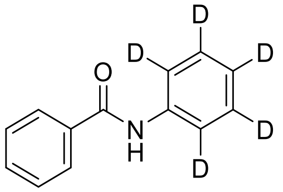

9a-*d*5
MNa$^+$ 225

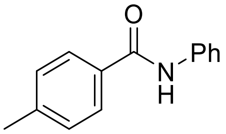

9b
MNa$^+$ 234

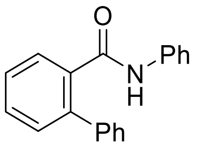

10a
MNa$^+$ 296

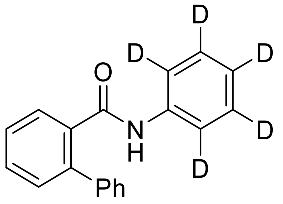

10a-*d*5
MNa$^+$ 301

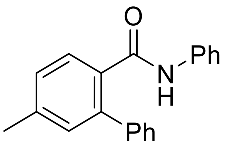

10b
MNa$^+$ 310

Supplementary Figure 8. By-products observed by MS of mixed column fractions.

2.6.3 Confirmation of Additional Side-Product Structure by Chromatographic Methods

Preparative HPLC (Supplementary Figure 9) was performed on a reaction of **1a** performed with 5 mol% $Pd(OAc)_2$, 5 mol% dppp and 2.0 eq. K_2CO_3 in DMF at 130 °C for 2 h (see section 2.6.1). LC-MS and GC-MS was performed on the fractions and comparison with authentic samples was used to confirm structure.

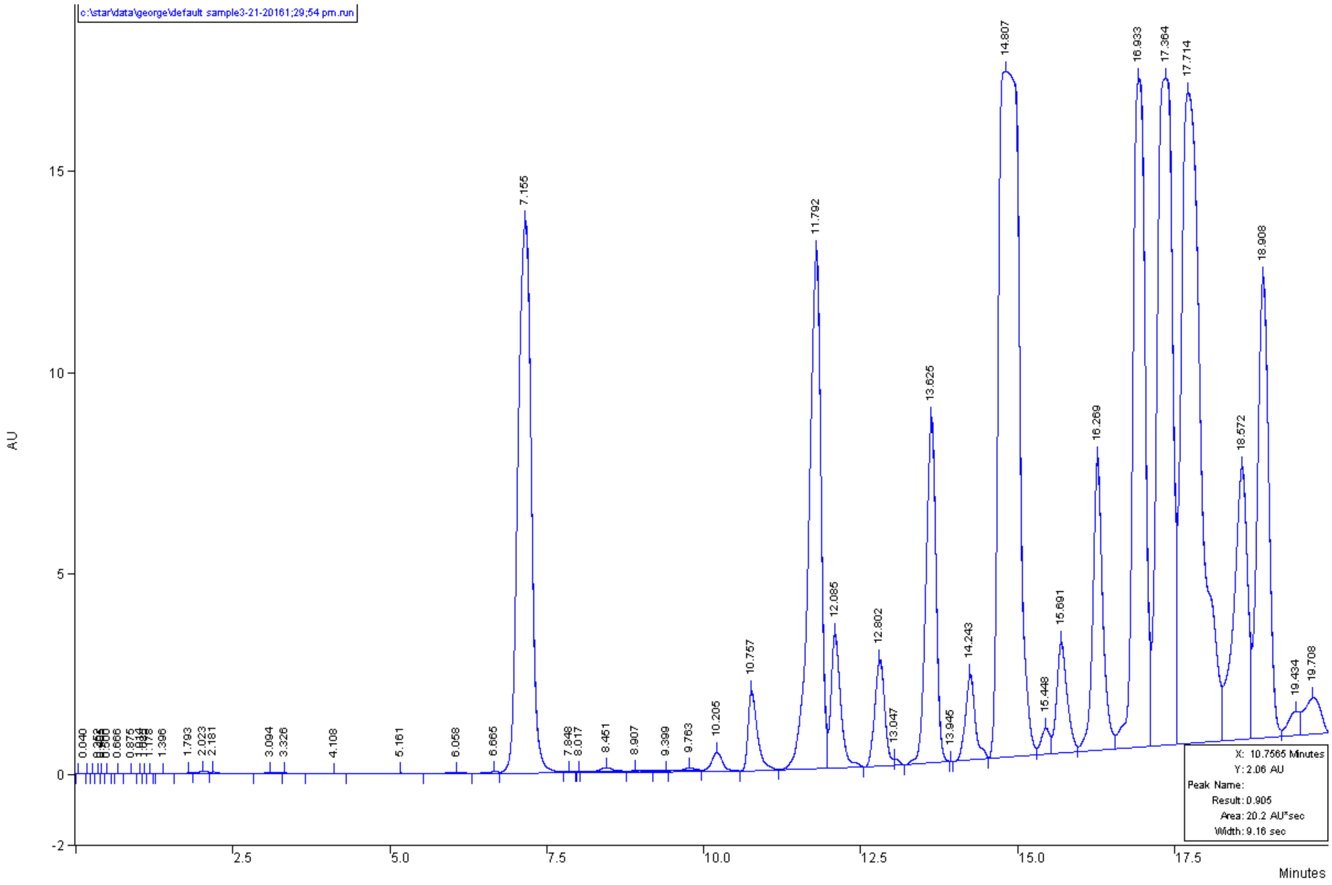

Supplementary Figure 9. Prep-HPLC chromatogram. Reaction conditions: **1a** with 5 mol% $Pd(OAc)_2$, 5 mol% dppp and 2.0 eq. K_2CO_3 in DMF at 130 °C for 2 h.

2.6.4 Confirmation of Side-Product Structure by LC-MS

Confirmation of the structure of compounds shown in Figure 1 and detailed in Supplementary Table 2 was achieved through comparison of the LC-MS traces of the reaction mixtures, compounds isolated from prep-HPLC and those of commercially available materials (**3a**, **8a**, **9a, 16a**, **17a**, and **22a**) or independently synthesized analytical standards (see Section 2.8). See Supplementary Table 2 for compound retention times.

The structures of **14a** and **15a** were not independently verified. Given they are only observed when using MEK as the solvent, their structures are assumed to be products of the mono- and di-α-arylation of MEK respectively and may be mixtures of regioisomeric products.[17–19]

Quinazolinedione **18a** was identified by comparison of its ESI +ve mode mass spectrum and reported diagnostic ^{1}H NMR spectral data. Where the regiochemistry was ambiguous (**12a**), synthesis of multiple possible isomers was undertaken, and LC-MS and GC-MS used to confirm the correct regiochemistry. Supplementary Figure 10 shows the regioisomers of **12a** synthesized.

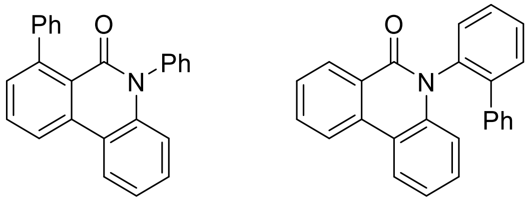

12a **12a'**

Supplementary Figure 10. Potential regioisomers of **12a**.

2.6.5 Confirmation of Additional Side-Product Structures Not Observed by HTE

Additional compounds **20a**, **21a**, **22a**, and **23-25** (Supplementary Figure 11, Supplementary Table 3) were identified from LC-MS and GC-MS of some prep-HPLC fractions. Confirmation of the structure of these compounds, shown in Figure 3 (in the manuscript), was achieved through comparison of the LC-MS and GC-MS traces of the prep-HPLC fractions with commercially available materials.

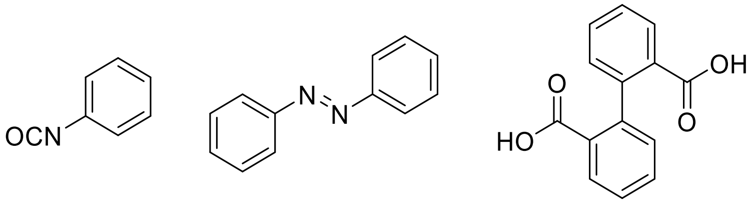

20a **21a** **22a**

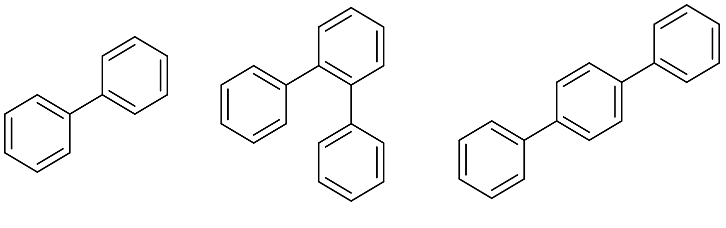

23 **24** **25**

Supplementary Figure 11. Further compounds identified by LC-MS and GC-MS.

Supplementary Table 3. Additional compounds identified by GC-MS and retention times.

Structure	Compound Number	Molecular Formula	Monoisotopic Mass	GC-MS Retention Time / min	LC-MS Retention Time / min
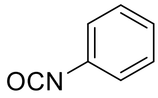	**20a**	C_7H_5NO	119	4.42	-
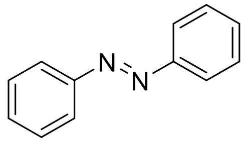	**21a**	$C_{12}H_{10}N_2$	182	9.13	-
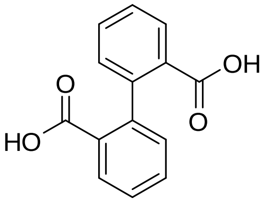	**22a**	$C_{14}H_{10}O_4$	242	11.62	5.2
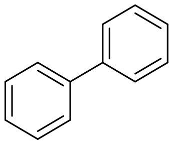	**23**	$C_{12}H_{10}$	154	7.64	-
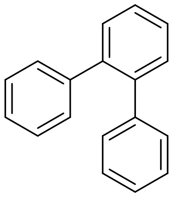	**24**	$C_{18}H_{14}$	230	10.50	-
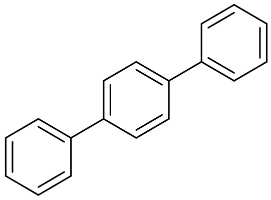	**25**	$C_{18}H_{14}$	230	11.76	-

2.6.6 Compounds Shown to Not Be Present in Reaction Mixtures

The following compounds (Supplementary Figure 12) were suspected to be present in the reaction mixtures by HRMS analysis but were not observed through comparison of the LC-MS and GC-MS traces of the prep-HPLC fractions with commercially available materials (**S1** and **S2**) or independently synthesized analytical standards (**S3**, **S4**, **S5** and **S6**). Compounds **S4**, **S5** and **S6** were speculated to be the structure of unidentified species **19a** (see Figure 3 in manuscript).

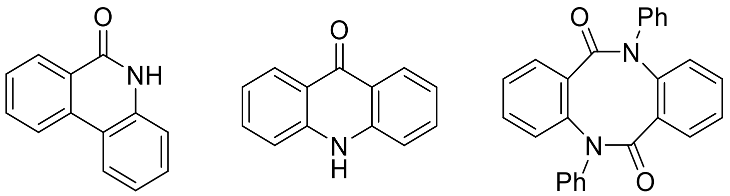

S1 **S2** **S3**

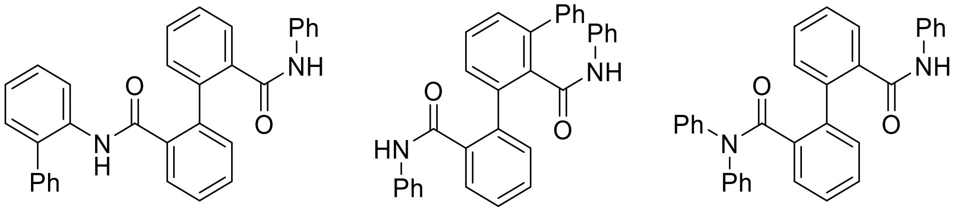

S4 **S5** **S6**

Supplementary Figure 12. Further compounds suspected to be present in the reaction mixtures.

2.6.7 Origin of the 'new' phenyl ring 10a and 12a

The studies detailed above (Supplementary Figure 13; section 2.6.2) indicate that **10a-d$_5$** and **10b** are formed when using **1a-d$_5$** and **1b** respectively. These experiments show that the 'new' ring of **10a** does not originate from the starting material **1**. Experiments using PPh_3 and PPh_3-d_{15} as ligands indicate that the 'new' phenyl ring of **10a** and **12a** originate from the ligand. Toluene was used as the solvent as this leads to the highest conversion to **10a**.

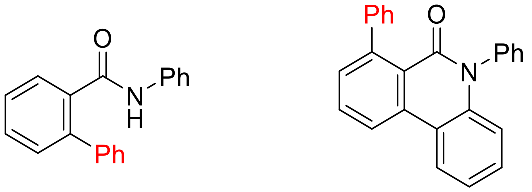

10a **12a**

Supplementary Figure 13. Sturucures of **10a** and **12a** with the 'new' ring coloured red.

***N*-phenyl-[1,1'-biphenyl]-2-carboxamide 10a & 5,7-diphenyl-5,6-dihydrophenanthridin-6-one 12a**

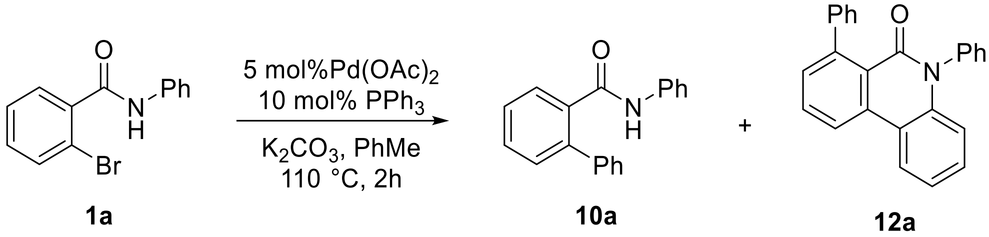

A Schlenk tube was charged with $Pd(OAc)_2$ (14 mg, 0.063 mmol, 5 mol%), PPh_3 (33 mg, 0.125 mmol, 10 mol%) and K_2CO_3 (345 mg, 2.5 mmol, 2.0 eq.) under N_2. A second Schlenk tube was charged with 2-bromo-*N*-phenylbenzamide **1a** (350 mg, 1.25 mmol, 1.0 eq.). Toluene (5 mL) was added to the first tube and toluene (3 mL) was added to the second tube. The catalyst mixture was heated to 110 °C for 2 min before the solution of **1a** at 110 °C was transferred into the catalyst mixture via cannula. The resulting mixture was stirred at 110 °C for 2 h then allowed to cool to rt. Then, EtOAc (20 mL) was added, and the resulting mixture was filtered through Celite and washed with EtOAc. The filtrate was washed with 1M $HCl_{(aq)}$ (20 mL) and brine (20 mL), dried over $MgSO_4$, filtered and the concentrated under reduced pressure to give a crude product as an orange solid (101 mg). The crude mixture contained **10a** by LC-MS ([M + H^+] 274, 8.1 min) and **12a** by LC-MS ([M + H^+] 348, 10.5 min).

***N*-Phenyl(2',3',4',5',6'-$^2H_5$)-[1,1'-biphenyl]-2-carboxamide 10a-*d*$_5$B & 5-phenyl-7-[(2,3,4,5,6-$^2H_5$)phenyl]-5,6-dihydrophenanthridin-6-one 12a-*d*$_5$**

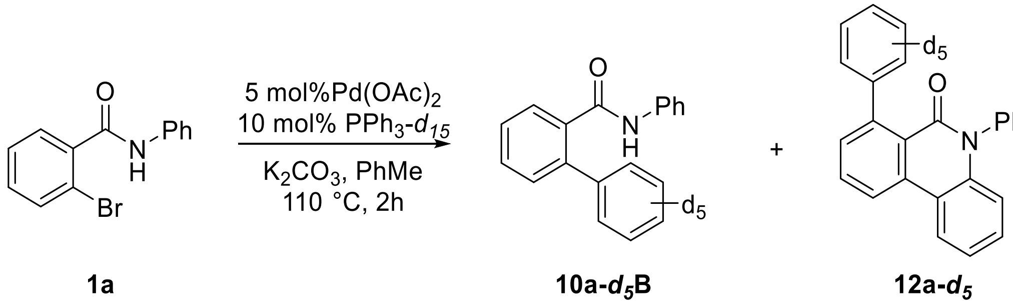

A Schlenk tube was charged with $Pd(OAc)_2$ (14 mg, 0.063 mmol, 5 mol%), PPh_3-d_{15} (35 mg, 0.125 mmol, 10 mol%) and K_2CO_3 (345 mg, 2.5 mmol, 2.0 eq.) under N_2. A second Schlenk tube was charged with 2-bromo-*N*-phenylbenzamide **1a** (350 mg, 1.25 mmol, 1.0 eq.). Toluene (5 mL) was added to the first tube and toluene (3 mL) was added to the second tube. The catalyst mixture was heated to 110 °C for 2 min before the solution of **1a** at 110 °C was transferred into the catalyst mixture via cannula. The resulting mixture was stirred at 110 °C for 2 h then allowed to cool to rt. Then, EtOAc (20 mL) was added, and the resulting mixture was filtered through Celite and washed with EtOAc. The filtrate was

washed with 1M $HCl_{(aq)}$ (20 mL) and brine (20 mL), dried over $MgSO_4$, filtered and the concentrated under reduced pressure to give a crude product as an orange solid (101 mg). The crude mixture contained **10a-*d*$_5$B** by LC-MS ([M + H^+] 279, 8.1 min) and **12a-*d*$_5$** by LC-MS ([M + H^+] 353, 10.5 min). Purification by flash column chromatography (SiO_2, 9:1 to 55:45 petrol:EtOAc), afforded the *title compound* **10a-*d*$_5$B** as a white solid (2.6 mg, 0.3%).

***R*f:** 0.51 (petrol/EtOAc, 7:3).

^{1}H NMR (400 MHz, $CDCl_3$): δ 7.92–7.89 (m, 1H, Ar), δ 7.55–7.47 (m, 2H, Ar), 7.45–7.42 (m, 1H, Ar), 7.24–7.21 (m, 2H, Ar), 7.11–7.03 (m, 3H, Ar), 6.87 (br s, 1H, NH);

MS (ESI): $[M + H]^+$: 279.1, $[M + Na]^+$: 301.1.

2.6.8 Synthesis of Analytical Standards

*N*2,*N*2'-Diphenyl-[1,1'-biphenyl]-2,2'-dicarboxamide 4a

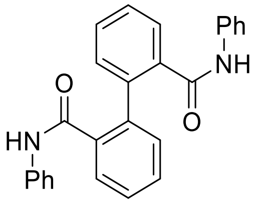

4a

A mixture of aryl bromide **1a** (552 mg, 2.0 mmol, 1.0 eq.), B_2pin_2 (559 mg, 2.2 mmol, 1.1 eq.), $PdCl_2dppf$ (73 mg, 0.1 mmol, 5 mol%), KOAc (589 mg, 6.0 mmol, 3.0 eq.) and dioxane (20 mL) was degassed with N_2 for 10 min. Then, the resulting mixture was heated to 90 °C and stirred at 90 °C for 16 h. Upon cooling, EtOAc (20 mL) and water (30 mL) were added, and the layers separated. The aqueous layer was extracted with EtOAc (2 × 20 mL). The combined organic layers were dried ($MgSO_4$), filtered and concentrated under reduced pressure. The crude product was purified by flash column chromatography (SiO_2, 97:3 CH_2Cl_2:Et_2O), to afford the *title compound* **4a** as a white solid (124 mg, 32%).

MP: 231–232 °C (Lit: 230-232 °C[20]);

***R*f:** (97:3 CH_2Cl_2:Et_2O) 0.23;

^{1}H NMR (400 MHz, $CDCl_3$): δ 8.99 (br s, 2H, NH), 7.71–7.67 (m, 2H, Ar), 7.47–7.36 (m, 8H, Ar), 7.30–7.24 (m, 6H, Ar), 7.20–7.15 (m, 2H, Ar), 7.11–7.04 (m, 2H, Ar);

^{13}C NMR (101 MHz, $CDCl_3$): δ 168.3 (C=O), 139.1 (*ipso*-Ar), 137.9 (*ipso*-Ar), 135.8 (*ipso*-Ar) 130.2 (Ar), 129.7 (Ar), 128.9 (Ar), 128.1 (Ar), 127.2 (Ar), 124.5 (Ar), 119.9 (Ar);

IR (ATR): 3270, 3012, 1654, 1639 (C=O), 1620, 1598, 1589, 1545, 1499, 1488, 1439, 1328, 753, 709, 691, 507 cm^{-1};

HRMS (ESI): calc. for $C_{26}H_{21}N_2O_2$ $[M + H]^+$: 393.1598; found 393.1599 (+0.3 ppm).

The analytical data obtained was in accordance with the literature.[13]

2-Bromo-3-nitro-*N*-phenylbenzamide S7

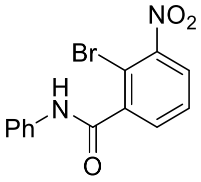

S7

Oxalyl chloride (339 µL, 4.0 mmol, 1.0 eq.) was added to a solution of 2-bromo-3-nitro benzoic acid (984 mg, 4.0 mmol, 1.0 eq.) and DMF (1 drop) in CH_2Cl_2 (10 mL) at 0 °C. The solution was warmed to rt and stirred for 1 h then concentrated under reduced pressure to provide the benzoyl chloride intermediate. A solution of 2-bromo-3-nitro benzoyl chloride (max. 4.0 mmol) in CH_2Cl_2 (5 mL) was added dropwise to a stirred solution of aniline (364 µL, 4.0 mmol, 1.0 eq.) and Et_3N (697 µL, 5.0 mmol, 1.2 eq.) in CH_2Cl_2 (5 mL) at 0 °C. The mixture was warmed to rt and stirred at rt for 16 h. Water (10 mL) was added, and the layers were separated. The aqueous layer was extracted with CH_2Cl_2 (2 × 10 mL). The combined organic layers were washed with water (10 mL) and brine (10 mL), dried ($MgSO_4$), filtered and concentrated under reduced pressure. The crude product was purified by flash column chromatography (SiO_2, 4:1 to 1:1 hexane:EtOAc), to afford the *title compound* **S7** as a yellow solid (1.05 g, 82%).

MP: 161–163 °C (Lit. 162.5–163.5 °C[21]);

***R*f:** (4:1 hexane:EtOAc) 0.20;

^{1}H NMR (400 MHz, $CDCl_3$): δ 7.80 (dd, *J* = 8.0, 1.5 Hz, 1H, Ar), 7.75 (dd, *J* = 8.0, 1.5 Hz, 1H, Ar), 7.65–7.52 (m, 4H, Ar and NH), 7.45–7.36 (m, 2H, Ar), 7.25–7.18 (m, 1H, Ar);

^{13}C NMR (101 MHz, $CDCl_3$): δ 164.1 (C=O), 151.1 (*ipso*-Ar), 141.2 (*ipso*-Ar), 136.9 (Ar), 131.8 (Ar), 129.3 (Ar), 128.8 (Ar), 126.1 (Ar), 125.5 (Ar), 120.2 (Ar), 111.4 (*ipso*-Ar);

IR (ATR): 1658 (C=O), 1600, 1523, 1500, 1439, 1366, 1323, 1265, 753, 702, 686, 506 cm^{-1};

HRMS (ESI): calc. for $C_{13}H_{10}{}^{79}BrNO_3$ $[M + H]^+$: 320.9869; found: 320.9867 (+0.8 ppm).

The analytical data obtained was in accordance with the literature.[22]

Methyl 2'-nitro-6'-(phenylcarbamoyl)-[1,1'-biphenyl]-2-carboxylate S8

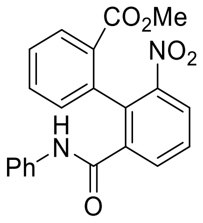

S8

A mixture of aryl bromide **S7** (875 mg, 2.72 mmol, 1.0 eq.), methyl 2-iodobenzoate (1.60 mL, 10.9 mmol, 4.0 eq.) and copper powder (1.50 g, 23.6 mmol, 8.7 eq.) were heated to 150 °C and stirred at 150 °C for 16 h. Upon cooling, EtOAc (30 mL) was added and the suspension filtered through Celite and washed with EtOAc (100 mL). The filtrate was concentrated under reduced pressure to give a crude product which was subsequently purified by flash column chromatography (SiO_2, 6:4 to 0:10 hexane:Et_2O), to afford the *title compound* **S8** as a yellow solid (150 mg, 15%).

MP: 165–167 °C;

$\boldsymbol{R_f}$**:** (7:3 hexane:Et_2O) 0.27;

^{1}H NMR (400 MHz, $CDCl_3$): δ 8.62 (br s, 1H, NH), 7.98 (dd, *J* = 12.0, 1.5 Hz, 1H, Ar), 7.96 (dd, *J* = 12.0, 1.5 Hz, 1H, Ar), 7.93–7.85 (m, 1H, Ar), 7.64 (app. t, *J* = 8.0 Hz, 1H, Ar), 7.52 (td, *J* = 7.5, 1.5 Hz, 1H, Ar), 7.43 (td, *J* = 7.5, 1.5 Hz, 1H, Ar), 7.28–7.11 (m, 5H, Ar), 7.09–6.98 (m, 1H, Ar), 3.90 (s, 3H, Me);

^{13}C NMR (101 MHz, $CDCl_3$): δ 169.8 (C=O), 165.4 (C=O), 148.4 (*ipso*-Ar), 139.7 (*ipso*-Ar), 137.3 (*ipso*-Ar), 134.9 (*ipso*-Ar), 132.7 (*ipso*-Ar), 132.5 (Ar), 132. (Ar)2, 130.8 (*ipso*-Ar), 130.5 (Ar), 129.2 (Ar), 129.1 (Ar), 129.0 (Ar), 128.9 (Ar), 125.1 (Ar), 124.6 (Ar), 119.7 (Ar), 53.0 (Me);

IR (ATR): 2951, 1718 (C=O, ester), 1666, 1646 (C=O, amide), 1523, 1446, 1321, 1262, 1132, 1084, 771, 758, 739, 692 cm^{-1};

HRMS (ESI): calc. for $C_{21}H_{17}N_2O_5$ $[M + H]^+$: 377.1132; found: 377.1133 (–0.2 ppm).

6-Oxo-*N*-phenyl-5,6-dihydrophenanthridine-1-carboxamide S9

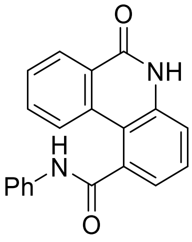
S9

5% Pd/C (25 mg) was added to a stirred solution of **S8** (126 mg, 0.33 mmol, 1.0 eq.) in MeOH (30 mL). The reaction flask was evacuated under reduced pressure and back-filled with N_2 three times. After the final evacuation, H_2 was charged, and the reaction mixture was stirred vigorously under a balloon of H_2 for 16 h. A white precipitate formed. Then, MeOH (70 mL) and *p*-toluenesulfonic acid monohydrate (64 mg, 0.33 mmol, 1.0 eq.) were added. The resulting mixture heated to 70 °C and stirred at 70 °C for 16 h. Upon cooling, the mixture was concentrated under reduced pressure. DMSO (5 mL) was added and heated to dissolve the white precipitate and the suspension was filtered through celite and washed with DMSO (1 mL). [NOTE: the white precipitate is insoluble in MeOH, EtOH, CH_2Cl_2, MeCN, toluene, EtOAc and AcOH]. EtOAc (50 mL) and $NaHCO_3$ (20 mL) were added, and the layers separated. The organic layer containing a white precipitate was concentrated under reduced pressure. Then, Et_2O (10 mL) was added to the residue and the solid collected by filtration, washed with Et_2O (10 mL) and dried under reduced pressure to afford the crude *title compound* **S9** as a white solid (165 mg).

^{1}H NMR (400 MHz, DMSO-*d₆*): δ ^{1}H NMR (400 MHz, DMSO-d_6) δ 11.87 (br s, 1H, NH), 10.71 (br s, 1H, NH), 8.35 (dd, *J* = 8.0, 1.5 Hz, 1H, Ar), 8.14 (d, *J* = 8.0 Hz, 1H, Ar), 7.76–7.63 (m, 3H, Ar), 7.61–7.49 (m, 2H, Ar), 7.46 (dd, *J* = 8.0, 1.5 Hz, 1H, Ar), 7.40–7.30 (m, 2H, Ar), 7.25 (dd, *J* = 7.5, 1.5 Hz, 1H, Ar), 7.16–7.05 (m, 1H, Ar);

^{13}C NMR (101 MHz, DMSO-d_6): δ 169.0 (C=O), 160.5 (C=O), 139.1 (*ipso*-Ar), 137.4 (*ipso*-Ar), 135.7 (*ipso*-Ar), 133.1 (*ipso*-Ar), 132.4 (Ar), 129.1 (Ar), 129.0 (Ar), 128.2 (Ar), 127.7 (Ar), 126.5 (*ipso*-Ar), 125.0 (Ar), 124.0 (Ar), 122.4 (Ar), 119.8 (Ar), 117.4 (Ar), 114.0 (*ipso*-Ar);

HRMS (ESI): calc. for $C_{20}H_{14}N_2NaO_2$ $[M + Na]^+$: 337.0947; found: 337.0936 (+3.5 ppm).

6-Oxo-*N*,5-diphenyl-5,6-dihydrophenanthridine-1-carboxamide 5a

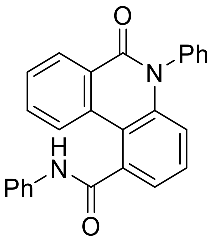

5a

A screw cap 7 mL vial was charged with lactam **S9** (max. 0.33 mmol, 1.0 eq.), iodobenzene (37 μL, 0.33 mmol, 1.0 eq.), K_2CO_3 (91 mg, 0.66 mmol, 2.0 eq.), CuI (6.3 mg, 0.033 mmol, 10 mol%), 1-(2-pyridyl)-3-(2-pyridyl)-1,3-propanedione (7.5 mg, 0.033 mmol, 10 mol%) and DMSO (2 mL). The vial was capped with a lid with a Teflon seal and the headspace was purged with N_2. The resulting mixture was heated to 140 °C and stirred at 140 °C for 16 h. Upon cooling, EtOAc (20 mL) and sat. $NaHCO_{3(aq)}$ (20 mL) were added, and the layers separated. The aqueous layer was extracted with EtOAc (2 × 10 mL). The combined organic layers were dried ($MgSO_4$), filtered and concentrated under reduced pressure. The crude product was purified by flash column chromatography (SiO_2, 7:3 hexane:EtOAc), to afford the *title compound* **5a** as a white solid (35 mg, 27%).

MP: 251–253 °C;

***R*f:** (7:3 petrol:EtOAc) 0.20;

^{1}H NMR (400 MHz, $CDCl_3$): δ 8.63 (br s, 1H, NH), 8.34 (d, *J* = 8.5 Hz, 1H, Ar), 8.06 (dd, *J* = 8.0, 1.5 Hz, 1H, Ar), 7.65 (ddd, *J* = 8.5, 7.0, 1.5 Hz, 1H, Ar), 7.62–7.58 (m, 2H, Ar), 7.47–7.38 (m, 5H, Ar), 7.37–7.28 (m, 3H, Ar), 7.19–7.11 (m, 1H, Ar), 6.81 (br s, 2H, Ar), 6.67 (dd, *J* = 8.5, 1.5 Hz, 1H, Ar);

^{13}C NMR (101 MHz, $CDCl_3$): δ 169.3 (C=O), 161.2 (C=O), 139.3 (*ipso*-Ar), 138.2 (*ipso*-Ar), 137.7 (*ipso*-Ar), 135.8 (*ipso*-Ar), 132.6 (Ar), 132.5 (*ipso*-Ar), 130.1 (Ar), 129.0 (Ar), 128.9 (Ar), 128.7 (Ar), 128.6 (Ar), 128.6 (Ar), 128.5 (Ar), 126.2 (Ar), 125.9 (Ar), 124.6 (Ar), 120.2 (Ar), 118.4 (Ar), 116.0 (*ipso*-Ar);

IR (ATR): 3061, 2923, 1645 (C=O), 1585, 1491, 1438, 1341, 1326, 754, 728, 692, 664, 644, 513 cm^{-1};

HRMS (ESI): calc. for $C_{26}H_{19}N_2O_2$ $[M + H]^+$: 391.1441; found: 391.1447 (–1.5 ppm).

An X-ray diffraction crystal structure of this compound was obtained (CCDC 2081723), see below:

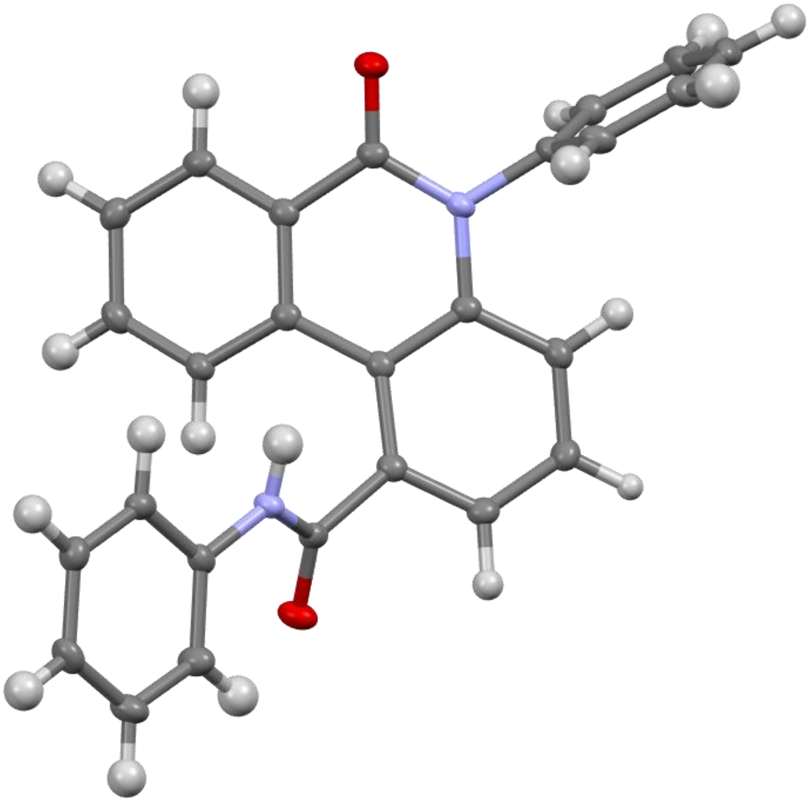

5-Hydroxy-2,2-dimethyl-2,4-dihydro-1,3-benzodioxin-4-one S10

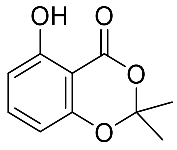

S10

Thionyl chloride (2.84 ml, 39.0 mmol, 1.3 eq.) was added to a stirred solution of 2,6-dihydroxybenzoic acid (4.62 g, 30.0 mmol, 1.0 eq.), DMAP (183 mg, 1.5 mmol, 5 mol%) and acetone (2.89 mL, 39.0 mmol, 1.3 eq.) in DME (20 mL) at 0 °C under N_2. The resulting solution was warmed to rt and stirred at rt for 16 h. Then, Et_2O (50 mL) and sat. $NaHCO_{3(aq)}$ (50 mL) were added, and the layers separated. The aqueous layer was extracted with Et_2O (2 × 50 mL). The combined organic layers were dried ($MgSO_4$), filtered and concentrated under reduced pressure. The crude product was purified by flash column chromatography (SiO_2, 9:1 petrol:EtOAc), to afford the *title compound* **S10** as an off white solid (4.72 g, 81%).

MP: 64–65 °C (Lit. 59–61 °C[23]);

***R*f:** (9:1 petrol:EtOAc) 0.28;

^{1}H NMR (400 MHz, CDCl$_3$): δ 10.3 (s, 1H, OH), 7.41 (t, *J* = 8.5 Hz, 1H, Ar), 6.63 (dd, *J* = 8.5, 1.0 Hz, 1H, Ar), 6.44 (dd, *J* = 8.5, 1.0 Hz, 1H, Ar), 1.75 (s, 6H, Me);

^{13}C NMR (101 MHz, $CDCl_3$): δ 165.6 (C=O or *ipso*-Ar), 161.5 (C=O or *ipso*-Ar), 155.7 (*ipso*-Ar), 138.1 (Ar), 107.4 (Ar), 107.2 (*ipso*-Ar), 99.4 (*C*Me_2), 25.8 (C*Me_2*);

IR (ATR): 3187, 1687 (C=O), 1631, 1385, 1204, 1151, 1079, 920, 803, 688, 668, 631, 620, 590 cm^{-1};

HRMS (APCI): calc. for $C_{10}H_{11}O_4$ $[M + H]^+$: 195.0652; found: 195.0649 (–1.4 ppm).

The analytical data obtained was in accordance with the literature.[23]

2,2-Dimethyl-4-oxo-2,4-dihydro-1,3-benzodioxin-5-yl trifluoromethanesulfonate S11

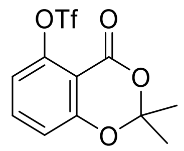

S11

Trifluoromethanesulfonic anhydride (2.02 ml, 12.0 mmol, 1.2 eq.) was added to a stirred solution of phenol **S10** (1.94 g, 10.0 mmol, 1.0 eq.) and pyridine (2.91 mL, 36.0 mmol, 3.6 eq.) in CH_2Cl_2 (10 mL) at 0 °C under N_2. The resulting solution was stirred at 0 °C for 2 h. Then, CH_2Cl_2 (20 mL) and water (30 mL) were added, and the layers separated. The aqueous layer was extracted with CH_2Cl_2 (2 × 20 mL). The combined organic layers were washed with sat. $CuSO_{4(aq)}$, dried ($MgSO_4$), filtered and concentrated under reduced pressure. The crude product was purified by flash column chromatography (SiO_2, 8:2 hexane:EtOAc), to afford the *title compound* **S11** as a white solid (3.06 g, 94%).

MP: 117–119 °C (Lit. 115–117 °C[23]);

***R_f*:** (8:2 hexane:EtOAc) 0.22;

^{1}H NMR (400 MHz, $CDCl_3$): δ 7.60 (t, *J* = 8.5 Hz, 1H, Ar), 7.06 (dd, *J* = 8.5, 1.0 Hz, 1H, Ar), 7.00 (d, *J* = 8.5 Hz, 1H, Ar), 1.76 (s, 6H, Me);

^{13}C NMR (101 MHz, $CDCl_3$): δ 157.4 (C=O or *ipso*-Ar), 157.1 (C=O or *ipso*-Ar), 148.6 (*ipso*-Ar), 136.3 (Ar), 118.7 (q, *J* = 321.0 Hz, CF_3), 117.9 (Ar), 116.5 (br, Ar), 108.3 (*ipso*-Ar or *C*Me_2), 106.9 (*ipso*-Ar or *C*Me_2), 25.4 (C*Me_2*);

IR (ATR): 2925, 1745 (C=O), 1621, 1437, 1206, 1137, 1021, 852, 836, 813, 750, 686, 629, 601, 590 cm^{-1};

HRMS (ESI): calc. for $C_{11}H_{10}F_3O_6S$ $[M + H]^+$: 327.0140; found: 327.0145 (+1.4 ppm).

The analytical data obtained was in accordance with the literature.[23]

2,2-Dimethyl-5-phenyl-2,4-dihydro-1,3-benzodioxin-4-one S12

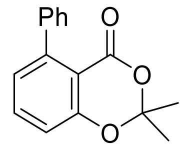

S12

A mixture of triflate **S11** (978 mg, 3.0 mmol, 1.0 eq.), phenylboronic acid (366 mg, 3.0 mmol, 1.0 eq.), $Pd(PPh_3)_4$ (35 mg, 0.03 mmol, 1 mol%), $NaHCO_3$ (504 mg, 6.0 mmol, 2.0 eq.), DME (9 mL) and water (1.8 mL) was degassed with N_2 for 10 min. Then, the resulting mixture was heated to 100 °C and stirred at 100 °C for 16 h. Upon cooling, EtOAc (20 mL) and water (30 mL) were added, and the layers separated. The aqueous layer was extracted with EtOAc (2 × 20 mL). The combined organic layers were dried ($MgSO_4$), filtered and concentrated under reduced pressure. The crude product was purified by flash column chromatography (SiO_2, 9:1 hexane:EtOAc, then 9:1 hexane:acetone), to afford the *title compound* **S12** as a white solid (643 mg, 84%).

MP: 162–163°C (Lit. 164–165 °C[24]);

***R*f:** (9:1 hexane:acetone) 0.16;

^{1}H NMR (400 MHz, $CDCl_3$): δ 7.52 (t, *J* = 8.0 Hz, 1H, Ar), 7.47–7.29 (m, 5H, Ar), 7.01 (d, *J* = 7.5 Hz, 1H, Ar), 6.98 (d, *J* = 8.0 Hz, 1H, Ar), 1.80 (s, 6H, Me);

^{13}C NMR (101 MHz, $CDCl_3$): δ 159.6 (C=O or *ipso*-Ar), 157.3 (C=O or *ipso*-Ar), 146.2 (*ipso*-Ar), 140.3 (*ipso*-Ar), 135.1 (Ar), 128.7 (Ar), 128.1 (Ar), 127.8 (Ar), 125.9 (Ar), 116.5 (Ar), 112.1 (*ipso*-Ar or *C*Me$_2$), 105.4 (*ipso*-Ar or *C*Me$_2$), 25.8 (C*Me*$_2$);

IR (ATR): 2996, 1727 (C=O), 1649, 1585, 1470, 1319, 1273, 1259, 1206, 1107, 1044, 813, 762, 696 cm^{-1};

HRMS (ESI): calc. for $C_{16}H_{15}O_3$ $[M + H]^+$: 255.1017; found: 255.1016 (–0.4 ppm).

The analytical data obtained was in accordance with the literature.[24]

3-Hydroxy-*N*-phenyl-[1,1'-biphenyl]-2-carboxamide S14

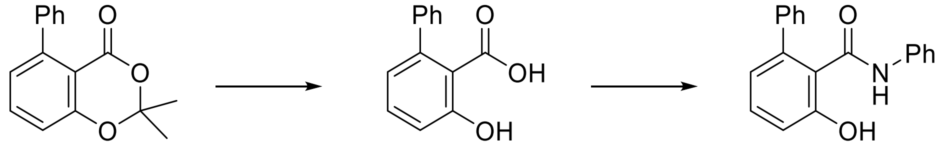

S12 **S13** **S14**

A solution of KOH (695 mg, 12.4 mmol, 5.0 eq.) in water (20 ml) was added to a stirred solution of ester **S12** (630 mg 2.48 mmol, 1.0 eq.) in THF (20 mL) at rt. Then, the resulting mixture was heated to 70 °C and stirred at 70 °C for 16 h. Upon cooling, EtOAc (30 mL) and 1 M $HCl_{(aq)}$ (30 mL) were added, and the layers separated. The aqueous layer was extracted with EtOAc (2 × 20 mL). The combined organic layers were dried ($MgSO_4$), filtered and concentrated under reduced pressure to give crude acid **S13** (522 mg) as an orange solid that was use without further purification.

^{1}H NMR (400 MHz, $CDCl_3$): δ 10.8 (br s, 1H, COOH or OH), 7.45 (dd, *J* = 8.5, 7.5 Hz, 1H, Ar), 7.45–7.33 (m, 3H, Ar), 7.33–7.25 (m, 2H, Ar), 7.01 (dd, *J* = 8.5, 1.5 Hz, 1H, Ar), 6.80 (dd, *J* = 7.5, 1.5 Hz, 1H, Ar).

Aniline (339 µL, 3.72 mmol, 1.5 eq.) was added to a stirred solution of crude carboxylic acid **S13** (522 mg, 2.48 mmol max, 1.0 eq.), EDC·HCl (713 mg, 3.72 mmol, 1.5 eq.), and DIPEA (1.30 mL, 7.44 mmol, 3.0 eq.) in CH_2Cl_2 (30 mL) under N_2. The resulting solution was stirred at rt for 16 h. Then, CH_2Cl_2 (10 mL) and sat. $NaHCO_{3(aq)}$ (20 mL) were added, and the layers separated. The aqueous layer was extracted with CH_2Cl_2 (2 × 30 mL). The combined organic layers were washed with 1M $HCl_{(aq)}$ (30 mL), dried ($MgSO_4$), filtered and concentrated under reduced pressure. The crude product was purified by flash column chromatography (SiO_2, 8:2 hexane:EtOAc), to afford the *title compound* **S14** as a white solid (375 mg, 54%).

MP: 79–80°C;

***R_f*:** (8:2 hexane:EtOAc) 0.31;

^{1}H NMR (400 MHz, $CDCl_3$): δ 11.9 (br s, 1H, OH), 7.56–7.46 (m, 5H, NH + Ar), 7.41 (dd, *J* = 8.5, 7.5 Hz, 1H, Ar), 7.25–7.19 (m, 2H, Ar), 7.11–7.04 (m, 3H, Ar), 6.97–6.93 (m, 2H, Ar), 6.84 (dd, *J* = 7.5, 1.5 Hz, 1H, Ar);

^{13}C NMR (101 MHz, $CDCl_3$): δ 168.2 (C=O), 161.6 (*ipso*-Ar), 141.0 (*ipso*-Ar), 140.3 (*ipso*-Ar), 136.4 (*ipso*-Ar), 132.7 (Ar), 129.4 (Ar), 129.2 (Ar), 128.8 (Ar), 128.7 (Ar), 124.9 (Ar), 122.0 (Ar), 120.2 (Ar), 117.7 (Ar), 114.7 (*ipso*-Ar);

IR (ATR): 3392, 3057, 1641 (C=O), 1534, 1490, 1436, 1209, 808, 756, 743, 705, 693, 565, 512, 482 cm^{-1};

HRMS (ESI): calc. for $C_{19}H_{16}NO_2$ $[M + H]^+$: 290.1179; found: 290.1176 (–1.3 ppm).

2-(Phenylcarbamoyl)-[1,1'-biphenyl]-3-yl trifluoromethanesulfonate S15

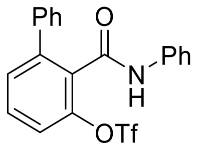

S15

Trifluoromethanesulfonic anhydride (91 µl, 0.54 mmol, 1.2 eq.) was added to a stirred solution of phenol **S14** (130 mg, 0.45 mmol, 1.0 eq.) and pyridine (131 µl, 36.0 mmol, 3.6 eq.) in CH_2Cl_2 (5 mL) at 0 °C under N_2. The resulting solution was stirred at 0 °C for 1 h. Then, CH_2Cl_2 (10 mL) and water (20 mL) were added, and the layers separated. The aqueous layer was extracted with CH_2Cl_2 (2 × 10 mL). The combined organic layers were washed with sat. $CuSO_{4(aq)}$, dried ($MgSO_4$), filtered and concentrated under reduced pressure. The crude product was purified by flash column chromatography (SiO_2, 75:25 hexane:EtOAc), to afford the *title compound* **S15** as a white solid (156 mg, 82%).

MP: 160–162 °C;

***R*f:** (7:3 hexane:EtOAc) 0.35;

^{1}H NMR (400 MHz, $CDCl_3$): δ 7.59–7.54 (m, 1H, Ar), 7.50–7.45 (m, 3H, Ar), 7.42–7.35 (m, 4H, Ar), 7.26–7.14 (m, 5H, Ar), 7.12–7.06 (m, 1H, Ar);

^{13}C NMR (101 MHz, $CDCl_3$): δ 162.2 (C=O), 146.0 (*ipso*-Ar), 142.3 (*ipso*-Ar), 137.9 (*ipso*-Ar), 136.6 (*ipso*-Ar), 131.0 (Ar), 130.2 (*ipso*-Ar), 130.1 (Ar), 128.8 (Ar), 128.6 (Ar), 128.4 (Ar), 120.8 (Ar), 120.7 (Ar), 118.4 (q, J = 320.3 Hz, CF_3);

IR (ATR): 3254, 1656 (C=O), 1600, 1415, 1217, 1135, 927, 809, 764, 754, 741, 698, 607, 497 cm^{-1};

HRMS (ESI): calc. for $C_{20}H_{15}F_3NO_4S$ $[M + H]^+$: 422.0668; found: 422.0675 (–1.6 ppm).

6-Oxo-5-phenyl-5,6-dihydrophenanthridin-7-yl trifluoromethanesulfonate S16

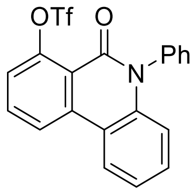

S16

A screw cap 7 mL vial was charged with amide **S15** (105 mg, 0.25 mmol, 1.0 eq.), *N*-iodo succinamide (169 mg, 0.75 mmol, 3.0 eq.), and 1,2-dichloroethane (2 mL). The vial was capped with a lid with a Teflon seal and the headspace was purged with N_2. The resulting mixture was heated to 110 °C and stirred at 110 °C for 16 h. Upon cooling, CH_2Cl_2 (10 mL) and sat. $Na_2S_2O_{3(aq)}$ (10 mL) were added, and the layers separated. The aqueous layer was extracted with CH_2Cl_2 (2 × 10 mL). The combined organic layers were dried ($MgSO_4$), filtered and concentrated under reduced pressure. The crude product was purified by flash column chromatography (SiO_2, 75:25 hexane:EtOAc), to afford the *title compound* **S16** as a white solid (49 mg, 47%).

MP: 235–236 °C;

***R*f:** (7:3 hexane:EtOAc) 0.31;

^{1}H NMR (400 MHz, CDCl$_3$): δ 8.42 (d, *J* = 8.5 Hz, 1H), 8.27 (dd, *J* = 8.0, 1.5 Hz, 1H), 7.84 (t, *J* = 8.0 Hz, 1H), 7.63–7.57 (m, 2H), 7.55–7.50 (m, 1H), 7.41 (d, *J* = 8.0 Hz, 1H), 7.37–7.27 (m, 4H), 6.65 (dd, *J* = 8.0, 1.5 Hz, 1H);

^{13}C NMR (101 MHz, CDCl$_3$): δ 158.7 (C=O), 149.8 (*ipso*-Ar), 139.6 (*ipso*-Ar), 137.6 (*ipso*-Ar), 137.4 (*ipso*-Ar), 133.1 (Ar), 130.4 (Ar), 130.3 (Ar), 129.1 (Ar), 128.9 (Ar), 123.6 (Ar), 123.0 (Ar), 122.6 (Ar), 122.0 (Ar), 120.5 (q, *J* = 294.8 Hz, CF_3) 120.4 (*ipso*-Ar), 119.1 (*ipso*-Ar), 117.5 (*ipso*-Ar), 117.1 (Ar);

IR (ATR): 3060, 2925, 1711, 1659 (C=O), 1607, 1424, 1319, 1226, 1217, 1202, 1142, 931, 826, 756, 597 cm^{-1};

HRMS (ESI): calc. for $C_{20}H_{13}F_3NO_4S$ $[M + H]^+$: 420.0512; found: 420.0508 (+0.8 ppm).

***N*-Phenyl-2-(4,4,5,5-tetramethyl-1,3,2-dioxaborolan-2-yl)benzamide S17**

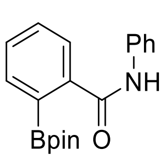

S17

HATU (1.4 g, 3.0 mmol, 1.5 eq.) was added to a stirred solution of 2-(4,4,5,5-tetramethyl-1,3,2-dioxaborolan-2-yl)benzoic acid (496 mg, 2.0 mmol, 1.0 eq.) and DIPEA (522 µL, 3.0 mmol, 1.5 eq.) in CH_2Cl_2 (20 mL) under N_2. The resulting solution was stirred at rt for 30 min, then aniline (273 µL, 3.0 mmol, 1.5 eq.) was added and the resulting mixture was stirred at rt for 16 h. Then, CH_2Cl_2 (20 mL) and sat. $NaHCO_{3(aq)}$ (30 mL) were added, and the layers separated. The aqueous layer was extracted with CH_2Cl_2 (2 × 20 mL). The combined organic layers were dried ($MgSO_4$), filtered and concentrated under reduced pressure. The crude product

was purified by flash column chromatography (SiO_2, 97:3 to 9:1 $CH_2Cl_2:Et_2O$), to afford the *title compound* **S17** as a white solid (444 mg, 69%).

MP: 208–210°C;

***R*f:** (19:1 $CH_2Cl_2:Et_2O$) 0.35;

^{1}H NMR (400 MHz, CDCl$_3$): δ 8.94 (br s, 1H, NH), 7.75 (d, J = 8.0 Hz, 1H, Ar), 7.63 (d, J = 7.0 Hz, 1H, Ar), 7.56 (d, J = 7.5 Hz, 2H, Ar), 7.41 (td, J = 7.5, 1.0 Hz, 1H, Ar), 7.30–7.22 (m, 3H, Ar), 7.15–7.09 (m, 1H, Ar), 1.33 (s, 12H, Me);

^{13}C NMR (101 MHz, CDCl$_3$): δ 168.0 (C=O), 138.2 (*ipso*-Ar), 137.7 (*ipso*-Ar), 133.3 (Ar), 131.2 (Ar), 129.4 (Ar), 129.0 (Ar), 125.5 (Ar), 124.9 (Ar), 120.8 (Ar), 83.7 (*C*Me$_2$), 25.0 (C*Me*$_2$), the carbon next to boron was not observed, presumably due to quadrupole relaxation;

^{11}B NMR (128 MHz, CDCl$_3$): δ 27.2;

IR (ATR): 3059, 2974, 1718, 1606 (C=O), 1552, 1492, 1444, 1325, 1142, 1102, 1029, 753, 694, 676, 616, 516 cm^{-1};

HRMS (ESI): calc. for $C_{19}H_{22}BNNaO_3$ $[M + Na]^+$: 346.1585; found: 346.1599 (–3.0 ppm).

2-(6-Oxo-5-phenyl-5,6-dihydrophenanthridin-7-yl)-*N*-phenylbenzamide 6a

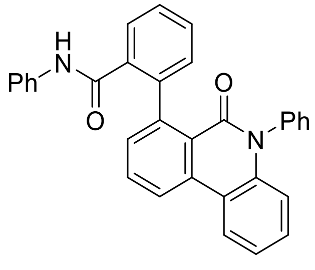

6a

A screw cap 7 mL vial was charged with pinacol boronate **S17** (35 mg, 0.11 mmol, 1.0 eq.), aryl triflate **S16** (46 mg, 0.11 mmol, 1.0 eq.), $Pd_2dba_3{\cdot}CHCl_3$ (5.7 mg, 0.005 mmol, 5 mol%), Sphos (4.5 mg, 0.011 mmol, 10 mol%), K_2CO_3 (46 mg, 0.33 mmol, 3.0 eq.), water (0.4 mL) and toluene (2 mL). The vial was capped with a lid with a Teflon seal and the mixture was degassed with N_2 for 10 min. Then, the resulting mixture was heated to 100 °C and stirred at 100 °C for 16 h. Upon cooling, EtOAc (20 mL) and water (20 mL) were added, and the layers separated. The aqueous layer was extracted with EtOAc (2 × 10 mL). The combined organic layers were dried ($MgSO_4$), filtered and concentrated under reduced pressure. The crude product was purified by flash column chromatography (SiO_2, 75:25 hexane:EtOAc), to afford the *title compound* **6a** as a white solid (22 mg, 43%).

MP: 221–223°C;

***R*f:** (7:3 hexane:EtOAc) 0.15;

^{1}H NMR (400 MHz, $CDCl_3$): δ 8.92 (br s, 1H, NH), 8.41–8.29 (m, 2H, Ar), 7.76 (dd, *J* = 8.0, 7.5 Hz, 1H, Ar), 7.66–7.63 (m, 1H, Ar), 7.61–7.55 (m, 2H, Ar), 7.54–7.48 (m, 1H, Ar), 7.41–7.30 (m, 6H, Ar), 7.27–7.23 (m, 2H, Ar), 7.19–7.13 (m, 2H, Ar), 7.13–7.09 (m, 1H, Ar), 7.07–7.04 (m, 1H, Ar), 6.98–6.93 (m, 1H, Ar), 6.71–6.60 (m, 1H, Ar);

^{13}C NMR (101 MHz, $CDCl_3$): δ 168.3 (C=O), 162.6 (C=O), 143.2 (*ipso*-Ar), 141.1 (*ipso*-Ar), 138.9 (*ipso*-Ar), 138.4 (*ipso*-Ar), 138.1 (*ipso*-Ar), 135.6 (*ipso*-Ar), 135.4 (*ipso*-Ar), 132.2 (Ar), 131.9 (Ar), 130.4 (Ar), 130.3 (Ar), 129.4 (Ar), 129.3 (Ar), 129.2 (Ar), 129.0 (Ar), 128.7 (Ar), 128.7 (Ar), 128.6 (Ar), 127.4 (Ar), 127.0 (Ar), 123.6 (Ar), 123.5 (Ar), 123.1 (*ipso*-Ar), 123.1 (Ar), 122.0 (Ar), 119.3 (Ar), 119.2 (*ipso*-Ar), 117.1 (Ar);

IR (ATR): 3259, 3062, 2247, 1671 (C=O), 1635 (C=O), 1599, 1540, 1499, 1490, 1444, 1318, 908, 752, 728, 694 cm^{-1};

HRMS (ESI): calc. for $C_{32}H_{23}N_2O_2$ $[M + H]^+$: 467.1754; found: 469.1762 (–1.6 ppm).

1-Azapentacyclo[10.7.1.0²,⁷.0⁸,²⁰.0¹³,¹⁸]icosa-2(7),3,5,8(20),9,11,13,15,17-nonaen-19-one 7a

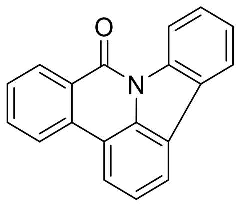

7a

A screw cap 7 mL vial was charged with 1-bromo-9H-carbazole (123 mg, 0.5 mmol, 1.0 eq.), 2-methoxycarbonylphenylboronic acid (90 mg, 0.5 mmol, 1.0 eq.), Na_2CO_3 (159 mg, 1.5 mmol, 3.0 eq.), $Pd(PPh_3)_4$ (57 mg, 0.05 mmol, 10 mol%), DME (1.2 mL), EtOH (0.8 mL) and H_2O (0.4 mL). The vial was capped with a lid with a Teflon seal and the mixture was degassed with N_2 for 10 min. The resulting mixture was heated to 100 °C and stirred at 100 °C for 16 h. Upon cooling, EtOAc (20 mL) and sat. $NaHCO_{3(aq)}$ (20 mL) were added, and the layers separated. The aqueous layer was extracted with EtOAc (2 × 10 mL). The combined organic layers were dried ($MgSO_4$), filtered and concentrated under reduced pressure. The crude product was purified by flash column chromatography (SiO_2, 6:4 to 3:7 hexane:CH_2Cl_2), to afford the *title compound* **7a** as a white solid (20 mg, 15%).

MP: 226–228 °C (Lit. 224–226 °C[15]);

***R*f:** (1:1 hexane:CH_2Cl_2) 0.21;

^{1}H NMR (400 MHz, $CDCl_3$): δ 8.83 (dt, *J* = 8.0, 1.0 Hz, 1H, Ar), 8.67 (dd, *J* = 8.0, 1.5 Hz, 1H, Ar), 8.30 (d, *J* = 8.0 Hz, 1H), 8.15 (d, *J* = 8.0 Hz, 1H, Ar), 8.08–8.03 (m, 2H, Ar), 7.82 (ddd, *J* = 8.0, 7.0, 1.5 Hz, 1H, Ar), 7.70–7.56 (m, 2H, Ar), 7.57 (t, *J* = 7.5 Hz, 1H, Ar), 7.49 (td, *J* = 7.5, 1.0 Hz, 1H, Ar);

^{13}C NMR (101 MHz, CDCl$_3$): δ 160.0 (C=O), 138.5 (*ipso*-Ar), 134.2 (*ipso*-Ar), 133.8 (*ipso*-Ar), 132.9 (Ar), 129.3 (Ar), 128.3 (Ar), 128.1 (Ar), 127.7 (*ipso*-Ar), 126.4 (*ipso*-Ar), 124.9 (Ar), 124.4 (*ipso*-Ar), 124.1 (Ar), 122.4 (Ar), 121.0 (Ar), 120.8 (Ar), 120.2 (Ar), 117.3 (Ar), 117.1 (*ipso*-Ar);

IR (ATR): 3039, 2922, 2852, 1694, 1662 (C=O), 1601, 1445, 1421, 1351, 1339, 1304, 1271, 757, 732, 688, 655 cm^{-1};

HRMS (ESI): calc. for $C_{19}H_{12}NO$ [M + H]$^+$: 270.0913; found: 270.0915 (–0.7 ppm).

The analytical data obtained was in accordance with the literature.[15]

***N*-Phenyl-[1,1'-biphenyl]-2-carboxamide 10a**

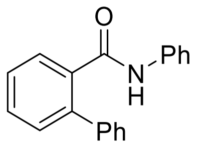

10a

Aniline (91 µL, 1.0 mmol, 1.0 eq.) was added to a stirred solution of biphenyl-2-carboxylic acid (198 mg, 1.0 mmol, 1.0 eq.), EDC·HCl (249 mg, 1.3 mmol, 1.3 eq.), DMAP (183 mg, 1.5 mmol, 1.5 eq.) and DIPEA (348 µL, 2.0 mmol, 2.0 eq.) in CH_2Cl_2 (5 mL) under N_2. The resulting solution was stirred at rt for 16 h. Then, CH_2Cl_2 (10 mL) and sat. $NaHCO_{3(aq)}$ (20 mL) were added, and the layers separated. The aqueous layer was extracted with CH_2Cl_2 (2 × 30 mL). The combined organic layers were washed with 1M $HCl_{(aq)}$ (30 mL), dried ($MgSO_4$), filtered and concentrated under reduced pressure. The crude product was purified by flash column chromatography (SiO_2, 7:3 hexane:EtOAc), to afford the *title compound* **10a** as a white solid (158 mg, 57%).

MP: 110–111 °C (lit. 111–113 °C[25]);

***R*$_f$:** (7:3 hexane:EtOAc) 0.40;

^{1}H NMR (400 MHz, CDCl$_3$): δ 7.88 (dd, *J* = 7.5, 1.5 Hz, 1H, Ar), 7.61–7.36 (m, 8H, Ar), 7.24–7.17 (m, 2H, Ar), 7.11–7.07 (m, 2H, Ar), 7.07–7.00 (m, 1H, Ar), 6.88 (br s, 1H, NH);

^{13}C NMR (101 MHz, CDCl$_3$): δ 167.2 (C=O), 140.0 (*ipso*-Ar), 139.3 (*ipso*-Ar), 137.6 (*ipso*-Ar), 135.3 (*ipso*-Ar), 130.8 (Ar), 130.5 (Ar), 129.7 (Ar), 129.1 (Ar), 129.0 (Ar), 128.2 (Ar), 128.0 (Ar), 124.5 (Ar), 120.0 (Ar);

IR (ATR): 3037, 1718, 1664 (C=O), 1518, 1498, 1435, 1318, 753, 742, 729, 701, 689, 670, 509 cm^{-1};

HRMS (ESI): calc. for $C_{19}H_{16}NO$ [M + H]$^+$: 274.1226; found: 274.1229 (–0.8 ppm).

The analytical data obtained was in accordance with the literature.[26]

2-Amino-*N*-phenylbenzamide S18

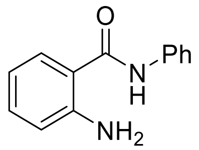

S18

Aniline (501 µL, 5.5 mmol, 1.1 eq.) and glacial acetic acid (2 drops) were added sequentially to a stirred suspension of isatoic anhydride (815 mg, 5.0 mmol, 1.0 eq.) in EtOH (30 mL) under N_2. The resulting solution was heated to 70 °C and stirred at 70 °C for 16 h. Upon cooling the mixture was concentrated under reduced pressure and the crude product was purified by flash column chromatography (SiO_2, 8:2 petrol:EtOAc), to afford the *title compound* **S18** as an off white solid (880 mg, 83%).

MP: 131–132 °C (Lit. 131–132 °C[27]);

***R*f:** (7:3 hexane:EtOAc) 0.20;

^{1}H NMR (400 MHz, CDCl$_3$): δ 7.75 (br s, 1H, NH), 7.60–7.55 (m, 2H, Ar), 7.47 (dd, *J* = 8.5, 1.5 Hz, 1H, Ar), 7.43–7.33 (m, 2H, Ar), 7.31–7.20 (m, 1H, Ar), 7.20–7.11 (m, 1H, Ar), 6.76–6.66 (m, 2H, Ar), 5.50 (br s, 2H, NH_2);

^{13}C NMR (101 MHz, CDCl$_3$): δ 167.7 (C=O), 149.1 (*ipso*-Ar), 137.9 (*ipso*-Ar), 135.9 (Ar), 132.9 (Ar), 129.2 (Ar), 124.6 (Ar), 120.6 (Ar), 117.7 (Ar), 116.3 (*ipso*-Ar);

IR (ATR): 3465, 3358, 3278, 3035, 1632 (C=O), 1598, 1530, 1499, 1486, 1439, 1315, 1250, 746, 689, 666, 585, 507cm^{-1};

HRMS (ESI): calc. for $C_{13}H_{13}N_2O$ $[M + H]^+$: 213.1022; found: 213.1022 (0.0 ppm).

The analytical data obtained was in accordance with the literature.[27]

***N*-Phenyl-2-(phenylamino)benzamide 12a**

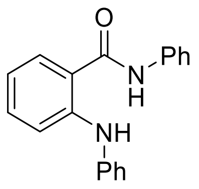

12a

Et_3N (35 µL, 0.25 mmol, 0.5 eq.) was added to a stirred suspension of aniline **S18** (106 mg, 0.5 mmol, 1.0 eq.), phenyl boronic acid (76 mg, 0.625 mmol, 1.25 eq.) and copper (I) chloride (7.5 mg, 0.075 mmol, 15 mol%) in MeOH (5 mL). The resulting suspension was stirred at rt for 16 h. Then, the mixture was concentrated under reduced pressure and the residue partitioned between CH_2Cl_2 (20 mL) and sat. $NaHCO_{3(aq)}$ (20 mL). The layers were separated, and the aqueous layer was extracted with CH_2Cl_2 (2 × 30 mL). The combined organic layers were dried ($MgSO_4$), filtered and concentrated under reduced pressure. The crude product was purified by flash column chromatography (SiO_2, 9:1 petrol:EtOAc), to afford the *title compound* **12a** as an off white solid (98 mg, 68%).

MP: 118–119 °C (Lit. 119–120 °C[28]);

***R*$_f$:** (9:1 petrol:EtOAc) 0.21;

^{1}H NMR (400 MHz, $CDCl_3$): δ 9.12 (br s, 1H, NH), 7.98 (br s, 1H, NH), 7.61–7.56 (m, 3H, Ar), 7.42–7.29 (m, 6H, Ar), 7.22–7.14 (m, 3H, Ar), 7.04 (tt, *J* = 7.5, 1.5 Hz, 1H, Ar), 6.83 (ddd, *J* = 8.0, 7.0, 1.5 Hz, 1H, Ar);

^{13}C NMR (101 MHz, $CDCl_3$): δ 167.7 (C=O), 145.6 (*ipso*-Ar), 141.3 (*ipso*-Ar), 137.5 (*ipso*-Ar), 132.6 (Ar), 129.3 (Ar), 129.0 (Ar), 127.6 (Ar), 124.7 (Ar), 122.5 (Ar), 120.7 (Ar), 118.7 (*ipso*-Ar), 118.3 (Ar), 118.1 (Ar), 115.8 (Ar);

IR (ATR): 3331, 3250, 3042, 1637 (C=O), 1588, 1512, 1444, 1315, 1286, 1265, 743, 691, 584, 529, 497, 484 cm^{-1};

HRMS (ESI): calc. for $C_{19}H_{17}N_2O$ $[M + H]^+$: 289.1335; found: 289.1339 (–1.3 ppm).

The analytical data obtained was in accordance with the literature.[29]

2,10-Diazatricyclo[10.4.0.0^{4,9}]hexadeca-1(12),4(9),5,7,13,15-hexaene-3,11-dione S19

Narrative: (prepared as an intermediate compound to potential reference compound **S3**, see overleaf).

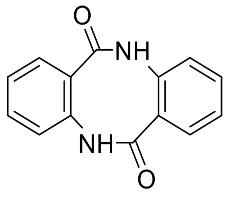

S19

A soution of methyl anthranilate (3.88 ml, 30.0 mmol, 1.0 eq.) in THF (15 mL) was added dropwise to a stirred suspension of sodium hydride (60% in mineral oil, 1.93 g, 48.3 mmol, 1.6 eq.) in THF (30 mL) ar rt under N_2. The resulting suspension was stirred at rt for 5 d, then water (10 mL) was added dropwise. 1 M $HCl_{(aq)}$ (200 mL) was added and the resulting precipitate was collected by filtration, washed with water (100 mL) and hexane (100 mL) and dried under reduced pressure to afford the *title compound* **S19** as a white solid (2.4 g, 67%).

MP: 335–337 °C (decomp.) (Lit. 344 °C[30]);

***R*f:** (9:1 petrol:EtOAc) 0.21;

^{1}H NMR (400 MHz, DMSO-*d*$_6$): δ 10.17 (br s, 2H, NH), 7.35–7.24 (m, 4H, Ar), 7.23–7.15 (m, 2H, Ar), 7.03 (br d, J = 8.0 Hz, 2H, Ar);

^{13}C NMR (101 MHz, DMSO-*d*$_6$): δ 169.4 (C=O), 134.8 (*ipso*-Ar), 133.6 (*ipso*-Ar), 130.6 (Ar), 128.2 (Ar), 127.3 (Ar), 125.8 (Ar);

IR (ATR): 3159, 3035, 2897, 1655 (C=O), 1640 (C=O), 1603, 1578, 1488, 1439, 1379, 784, 753, 691, 610, 536 cm^{-1};

HRMS (ESI): calc. for $C_{14}H_{11}N_2O_2$ [M + H]$^+$: 239.0815; found: 239.0820 (–2.0 ppm).

The analytical data obtained was in accordance with the literature.[30]

2,10-Diphenyl-2,10-diazatricyclo[10.4.0.0^{4,9}]hexadeca-1(12),4(9),5,7,13,15-hexaene-3,11-dione S3

Narrative: This compound was independently prepared to compare with a trace species observed by LC-MS *m/z* = 391. However, this species was not the same as the reference compound **S3**.

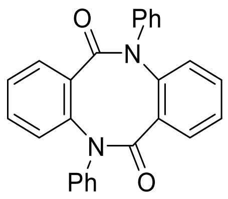

S3

A screw cap 7 mL vial was charged with bis-lactam **S19** (119 mg, 0.5 mmol, 1.0 eq.), iodobenzene (134 µL, 1.2 mmol, 2.4 eq.), K_2CO_3 (276 mg, 2.0 mmol, 4.0 eq.), CuI (10.0 mg, 0.05 mmol, 10 mol%), 1-(2-pyridyl)-3-(2-pyridyl)-1,3-propanedione (11.3 mg, 0.05 mmol, 10 mol%) and DMF (2 mL). The vial was capped with a lid with a Teflon seal and the headspace was purged with N_2. The resulting mixture was heated to 140 °C and stirred at 140 °C for 16 h. Upon cooling, EtOAc (20 mL) and sat. $NaHCO_{3(aq)}$ (20 mL) were added, and the layers separated. The aqueous layer was extracted with EtOAc (2 × 10 mL). The combined organic layers were dried ($MgSO_4$), filtered and concentrated under reduced pressure. The crude product was purified by flash column chromatography (SiO_2, 1:1 hexane:EtOAc), to afford the *title compound* **S3** as a white solid (96 mg, 49%).

MP: 277–279 °C (Lit. 345 °C[31]);

***R*f:** (1:1 petrol:EtOAc) 0.39;

^{1}H NMR (400 MHz, CDCl$_3$): δ 7.50–7.43 (m, 6H, Ar), 7.40–7.34 (m, 4H, Ar), 7.28–7.19 (m, 6H, Ar), 7.07–7.01 (m, 2H, Ar);

^{13}C NMR (101 MHz, CDCl$_3$): δ 167.8 (C=O), 141.0 (*ipso*-Ar), 139.7 (*ipso*-Ar), 136.0 (*ipso*-Ar), 130.9 (Ar), 129.4 (Ar), 128.6 (Ar), 127.5 (Ar), 127.3 (Ar), 127.1 (Ar);

IR (ATR): 3054, 1653 (C=O), 1596, 1491, 1448, 1357, 1331, 1319, 1252, 765, 755, 693, 623 cm^{-1};

HRMS (ESI): calc. for $C_{26}H_{19}N_2O_2$ $[M + H]^+$: 391.1441; found: 391.1443 (–0.4 ppm).

An X-ray diffraction crystal structure of this compound was obtained (CCDC 2081724), see below:

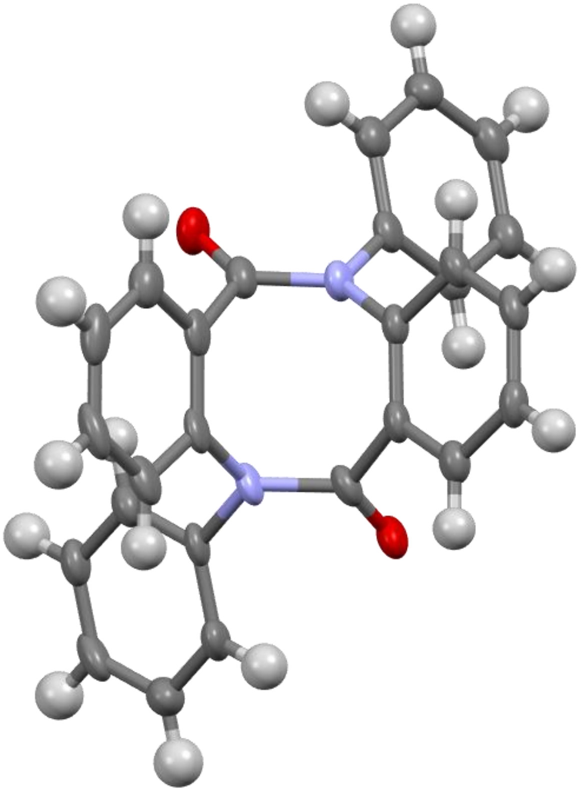

N,3-Diphenyl-[1,1'-biphenyl]-2-carboxamide S20

Narrative: This compound was independently prepared to compare with trace species detected by LC-MS. No definitive conclusion could be drawn from the results.

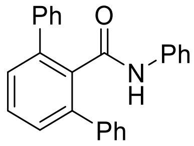

S20

A screw cap 7 mL vial was charged with benzanilide (99 mg, 0.5 mmol, 1.0 eq.), phenyl trifluoromethanesulfonate (324 μL, 2.0 mmol, 4.0 eq.), Cs_2CO_3 (651 mg, 2.0 mmol, 4.0 eq.), $Pd(OAc)_2$ (5.6 mg, 0.025 mmol, 5 mol%), PPh_3 (39 mg, 0.15 mmol, 30 mol%) and toluene (3 mL). The vial was capped with a lid with a Teflon seal and the headspace was purged with N_2. The resulting mixture was heated to 110 °C and stirred at 110 °C for 16 h. Upon cooling, EtOAc (20 mL) and sat. $NaHCO_{3(aq)}$ (20 mL) were added, and the layers separated. The aqueous layer was extracted with EtOAc (2 × 10 mL). The combined organic layers were dried ($MgSO_4$), filtered and concentrated under reduced pressure. The crude product was purified by flash column chromatography (SiO_2, 7:3 hexane:Et_2O), to afford the *title compound* **S20** as a white solid (85 mg, 48%).

MP: 268–269 °C (Lit. 270–271 °C[32]);

***R_f*:** (7:3 hexane:Et_2O) 0.19;

^{1}H NMR (400 MHz, CDCl$_3$): δ 7.56–7.49 (m, 5H, Ar), 7.44–7.30 (m, 8H, Ar), 7.19–7.13 (m, 2H, Ar), 7.05–6.98 (m, 1H, Ar), 6.94–6.89 (m, 2H, Ar), 6.86 (br s, 1H, NH);

^{13}C NMR (101 MHz, DMSO-*d$_6$*): δ 166.7 (C=O), 140.3 (*ipso*-Ar), 139.4 (*ipso*-Ar), 138.5 (*ipso*-Ar), 136.3 (*ipso*-Ar), 135.4 (*ipso*-Ar), 129.0 (Ar), 128.9 (Ar), 128.5 (Ar), 128.4 (Ar), 128.1 (Ar), 127.4 (Ar), 123.5 (Ar), 119.9 (Ar);

IR (ATR): 3216, 3057, 1642 (C=O), 1599, 1538, 1498, 1443, 1325, 757, 701, 692, 595, 554, 504 cm^{-1};

HRMS (ESI): calc. for $C_{25}H_{20}NO$ $[M + H]^+$: 350.1539; found: 350.1540 (–0.2 ppm).

The analytical data obtained was in accordance with the literature.[33]

5,7-Diphenyl-5,6-dihydrophenanthridin-6-one 12a

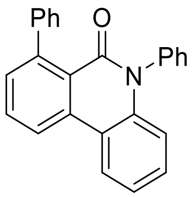

12a

A screw cap 7 mL vial was charged with amide **S20** (25 mg, 0.07 mmol, 1.0 eq.), *N*-iodo succinamide (48 mg, 0.21 mmol, 3.0 eq.), and 1,2-dichloroethane (2 mL). The vial was capped with a lid with a Teflon seal and the headspace was purged with N_2. The resulting mixture was heated to 110 °C and stirred at 110 °C for 16 h. Upon cooling, CH_2Cl_2 (10 mL) and sat. $Na_2S_2O_{3(aq)}$ (10 mL) were added, and the layers separated. The aqueous layer was extracted with CH_2Cl_2 (2 × 10 mL). The combined organic layers were dried ($MgSO_4$), filtered and concentrated under reduced pressure. The crude product was purified by flash column chromatography (SiO_2, 9:1 to 8:2 hexane:EtOAc), to afford the *title compound* **12a** as a white solid (8 mg, 33%).

MP: 205–206 °C;

***R*$_f$:** (9:1 hexane:EtOAc) 0.26;

^{1}H NMR (400 MHz, CDCl$_3$): δ 8.40 (d, *J* = 8.5 Hz, 1H, Ar), 8.36–8.31 (m, 1H, Ar), 7.77 (t, *J* = 8.0 Hz, 1H, Ar), 7.53–7.48 (m, 2H, Ar) 7.44–7.37 (m, 2H, Ar), 7.36–7.22 (m, 9H, Ar), 6.62–6.53 (m, 1H, Ar);

^{13}C NMR (101 MHz, CDCl$_3$): δ 160.8 (C=O), 145.6 (*ipso*-Ar), 143.7 (*ipso*-Ar), 139.6 (*ipso*-Ar), 138.5 (*ipso*-Ar), 135.4 (*ipso*-Ar), 132.2 (Ar), 131.5 (Ar), 130.1 (Ar), 129.3 (Ar), 129.1 (Ar), 128.5 (Ar), 128.1 (Ar), 127.5 (Ar), 126.4 (Ar), 123.4 (Ar), 123.3 (*ipso*-Ar), 122.3 (Ar), 121.5 (Ar), 118.9 (*ipso*-Ar), 116.7 (Ar);

IR (ATR): 2920, 2850, 1664 (C=O), 1602, 1587, 1471, 1309, 1298, 761, 749, 694, 670, 647, 615, 555 cm^{-1};

HRMS (ESI): calc. for $C_{25}H_{18}NO$ $[M + H]^+$: 348.1383; found: 348.1382 (+0.4 ppm).

N-{[1,1'-Biphenyl]-2-yl}-2-bromobenzamide 1c

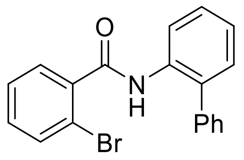

1c

Oxalyl chloride (846 µL, 10 mmol, 1.0 eq.) was added to a solution of 2-bromo benzoic acid (2.01 g, 10 mmol, 1.0 eq.) and DMF (1 drop) in CH_2Cl_2 (20 mL) at 0 °C. The solution was warmed to rt and stirred for 2 h then concentrated under reduced pressure to provide the 2-bromo benzoyl chloride intermediate. A solution of 2-bromo benzoyl chloride (max. 10 mmol) in CH_2Cl_2 (10 mL) was added dropwise to a stirred solution of 2-aminobiphenyl (1.69 g, 10 mmol, 1.0 eq.) and Et_3N (1.67 mL, 10 mmol, 1.0 eq.) in CH_2Cl_2 (10 mL) at 0 °C. The mixture was warmed to rt and stirred at rt for 2 h. Water (10 mL) was added and the layers were separated. The aqueous layer was extracted with CH_2Cl_2 (2 × 10 mL). The combined organic layers were washed with water (10 mL) and brine (10 mL), dried ($MgSO_4$), filtered and concentrated under reduced pressure. The crude product was purified by flash column chromatography (SiO_2, 9:1 hexane:EtOAc), to afford the *title compound* **1c** as a beige solid (2.24 g, 63%).

MP: 117–119 °C;

***R*f:** (9:1 hexane:EtOAc) 0.20;

^{1}H NMR (400 MHz, $CDCl_3$): δ 8.49 (d, *J* = 8.5 Hz, 1H, Ar), 7.70 (br s, 1H, NH), 7.54 (dd, *J* = 8.0, 1.5 Hz, 1H, Ar), 7.51 (dd, *J* = 7.5, 2.0 Hz, 1H, Ar), 7.48–7.22 (m, 10H);

^{13}C NMR (101 MHz, $CDCl_3$): δ 165.4 (C=O), 137.8 (*ipso*-Ar), 137.7 (*ipso*-Ar), 134.4 (*ipso*-Ar), 133.4 (Ar), 132.9 (*ipso*-Ar), 131.4 (Ar), 130.2 (Ar), 129.4 (Ar), 129.4 (Ar), 129.0 (Ar), 128.4 (Ar), 128.0 (Ar), 127.5 (Ar), 124.8 (Ar), 121.8 (Ar), 119.0 (*ipso*-Ar);

IR (ATR): 3397, 1672 (C=O), 1512, 1444, 1435, 1308, 757, 745, 706, 596, 587, 489, 458 cm^{-1};

HRMS (ESI): calc. for $C_{19}H_{15}{}^{79}BrNO$ $[M + H]^+$: 352.0332; found: 352.0334 (–0.6 ppm).

5-{[1,1'-Biphenyl]-2-yl}-5,6-dihydrophenanthridin-6-one 12a'

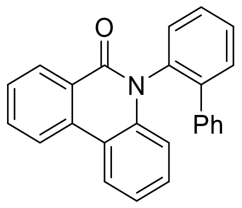

12a'

A screw cap 7 mL vial was charged with benzamide **1c** (176 mg, 0.5 mmol, 1.0 eq.), K_2CO_3 (138 mg, 1.0 mmol, 2.0 eq.), $Pd(OAc)_2$ (5.6 mg, 0.025 mmol, 5 mol%), dppe (10 mg, 0.025 mmol, 5 mol%) and DMF (2 mL). The vial was capped with a lid with a Teflon seal and the headspace was purged with N_2. The reaction mixture was heated to 130 °C and stirred at 130 °C for 2 h then allowed to cool to rt. Then, EtOAc (10 mL) was added, and the resulting mixture was filtered through Celite and washed with EtOAc. The filtrate was washed with 1M $HCl_{(aq)}$ (10 mL) and brine (10 mL), dried over $MgSO_4$, filtered and the concentrated under reduced pressure to give a crude product which was subsequently purified by flash column chromatography (SiO_2, 8:2 petrol:EtOAc), to afford the *title compound* **12a'** as a white solid (45 mg, 52%).

MP: 166–167°C;

***R*f:** (9:1 hexane:EtOAc) 0.23;

^{1}H NMR (400 MHz, $CDCl_3$): δ 8.51 (d, J = 8.0 Hz, 1H, Ar), 8.21 (d, J = 8.0 Hz, 1H, Ar), 8.15 (dd, J = 8.0, 1.5 Hz, 1H, Ar), 7.79–7.69 (m, 1H, Ar), 7.63–7.51 (m, 4H, Ar), 7.39–7.30 (m, 1H, Ar), 7.29–7.20 (m, 3H, Ar), 7.22–7.12 (m, 1H, Ar), 7.09–7.01 (m, 3H, Ar), 6.66 (d, J = 8.5 Hz, 1H, Ar);

^{13}C NMR (101 MHz, $CDCl_3$): (rotamers observed) δ 162.1 (C=O), 141.7 (*ipso*-Ar), 138.8 (*ipso*-Ar), 138.5 (*ipso*-Ar), 135.9 (*ipso*-Ar), 134.0 (*ipso*-Ar), 132.8 (Ar), 131.7 (Ar), 129.9 (Ar), 129.4 (Ar), 129.4 (Ar), 129.2 (Ar), 129.1 (Ar), 129.1 (Ar), 128.3 (Ar), 128.1 (Ar), 128.0 (Ar), 127.4 (Ar), 125.7 (*ipso*-Ar), 123.0 (Ar), 123.0 (Ar), 122.6 (Ar), 121.9 (Ar), 121.8 (Ar), 118.8 (*ipso*-Ar), 117.1 (Ar), 117.0 (Ar);

IR (ATR): 3043, 1650, 1605, 1438, 1338, 1319, 779, 761, 748, 724, 705, 691, 656 cm^{-1};

HRMS (ESI): calc. for $C_{25}H_{18}NO$ $[M + H]^+$: 348.1383; found: 348.1383 (+0.8 ppm).

2'-(Phenylcarbamoyl)-[1,1'-biphenyl]-2-carboxylic acid 13a

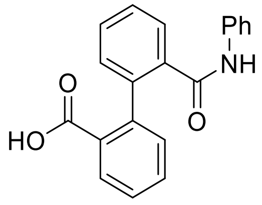

13a

DMAP (49 mg, 0.4 mmol, 0.2 eq.) was added to a stirred suspension of diphenic anhydride (448 mg, 2.0 mmol, 1.0 eq.), aniline (219 μL, 2.4 mmol, 1.2 eq.) and Et_3N (835 μL, 6.0 mmol, 3.0 eq.) in CH_2Cl_2 (20 mL). The resulting suspension was stirred at rt for 16 h. Then, CH_2Cl_2 (20 mL) and 1M $HCl_{(aq)}$ (20 mL) were added, and the layers were separated. The aqueous layer was extracted with CH_2Cl_2 (2 × 30 mL). The combined organic layers were dried ($MgSO_4$), filtered and concentrated under reduced pressure. The crude product was purified by flash column chromatography (SiO_2, 96:3:1 CH_2Cl_2:MeOH:AcOH), to afford the *title compound* **13a** as a white solid (465 mg, 73%).

MP: 181–183°C (Lit. 182.5–184 °C[34]);

***R*f:** (96:3:1 CH_2Cl_2:MeOH:AcOH) 0.23;

^{1}H NMR (400 MHz, acetone-*d*6): δ 9.36 (s, 1H, COOH or NH), 7.89–7.85 (m, 1H, Ar), 7.75–7.69 (m, 1H, Ar), 7.54–7.41 (m, 6H, Ar), 7.25–7.18 (m, 4H, Ar), 7.05–6.99 (m, 1H, Ar), COOH or NH not observed;

^{13}C NMR (101 MHz, acetone-*d*6): δ 169.8 (C=O), 167.7 (C=O), 140.7 (*ipso*-Ar), 139.6 (*ipso*-Ar), 138.9 (*ipso*-Ar), 132.0 (*ipso*-Ar), 130.4 (*ipso*-Ar), 129.9 (Ar), 129.7 (Ar), 129.0 (Ar), 128.7 (Ar), 127.9 (Ar), 127.8 (Ar), 127.7 (Ar), 123.9 (Ar), 119.3 (Ar);

IR (ATR): 2967, 1681 (C=O), 1620, 1608, 1595, 1551, 1490, 1340, 1304, 1233, 756, 747, 690, 649, 592, 512 cm^{-1};

HRMS (ESI): calc. for $C_{20}H_{16}NO_3$ $[M + H]^+$: 318.1125; found: 318.1125 (0.0 ppm).

The analytical data obtained was in accordance with the literature.[35]

***N*2-{[1,1'-Biphenyl]-2-yl}-*N*2'-phenyl-[1,1'-biphenyl]-2,2'-dicarboxamide S4**

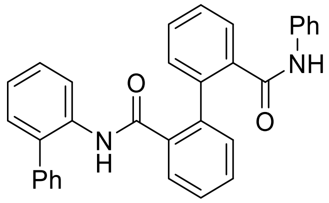

S4

A screw cap 7 mL vial was charged with pinacol boronate **S17** (32 mg, 0.1 mmol, 1.0 eq.), aryl bromide **1c** (35 mg, 0.1 mmol, 1.0 eq.), Pd_2dba_3 (4.6 mg, 0.005 mmol, 5 mol%), Sphos (4.1 mg, 0.01 mmol, 10 mol%), K_2CO_3 (41 mg, 0.3 mmol, 3.0 eq.), water (0.4 mL) and toluene (2 mL). The vial was capped with a lid with a Teflon seal and the mixture was degassed with N_2 for 10 min. Then, the resulting mixture was heated to 100 °C and stirred at 100 °C for 16 h. Upon cooling, EtOAc (20 mL) and water (20 mL) were added, and the layers separated. The aqueous layer was extracted with EtOAc (2 × 10 mL). The combined organic layers were dried ($MgSO_4$), filtered and concentrated under reduced pressure. The crude product was purified by flash column chromatography (SiO_2, 7:3 hexane:EtOAc), to afford the *title compound* **S4** as an off white solid (22 mg, 47%).

MP: 200–202 °C;

***R*f:** (7:3 hexane:EtOAc) 0.25;

^{1}H NMR (400 MHz, $CDCl_3$): δ 9.78 (br s, 1H, NH), 8.01 (dd, *J* = 8.5, 1.0 Hz, 1H, Ar), 7.85 (br s, 1H, NH), 7.74 (dd, *J* = 7.5, 1.5 Hz, 1H, Ar), 7.56–7.50 (m, 2H, Ar), 7.47–7.20 (m, 14H, Ar), 7.19–7.14 (m, 2H, Ar), 7.03 (dd, *J* = 7.5, 1.5 Hz, 1H, Ar), 7.00–6.94 (m, 1H, Ar);

^{13}C NMR (101 MHz, $CDCl_3$): δ 168.8 (C=O), 167.6 (C=O), 140.2 (*ipso*-Ar), 138.5 (*ipso*-Ar), 137.9 (*ipso*-Ar), 137.9 (*ipso*-Ar), 137.2 (*ipso*-Ar), 134.9 (*ipso*-Ar), 133.8 (*ipso*-Ar), 133.7 (*ipso*-Ar), 130.7 (Ar), 130.6 (Ar), 130.3 (Ar), 129.4 (Ar), 129.2 (Ar), 129.1 (Ar), 128.7 (Ar), 128.6 (Ar), 128.4 (Ar), 128.3 (Ar), 128.2 (Ar), 128.0 (Ar), 125.6 (Ar), 125.6 (Ar), 123.7 (Ar), 122.6 (Ar), 119.6 (Ar), one Ar peak not resolved;

IR (ATR): 3397, 3040, 1664 (C=O), 1584, 1514, 1491, 1440, 1309, 750, 702, 595, 543, 512, 489 cm^{-1};

HRMS (ESI): calc. for $C_{32}H_{25}N_2O_2$ $[M + H]^+$: 469.1911; found: 469.1908 (+0.5 ppm).

***N*-Phenyl-3-[2-(phenylcarbamoyl)phenyl]-[1,1'-biphenyl]-2-carboxamide S5**

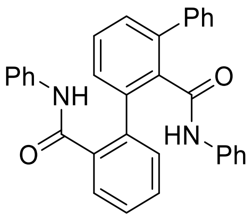

S5

A screw cap 7 mL vial was charged with pinacol boronate **S17** (32 mg, 0.1 mmol, 1.0 eq.), aryl triflate **S15** (42 mg, 0.1 mmol, 1.0 eq.), Pd_2dba_3 (4.6 mg, 0.005 mmol, 5 mol%), Sphos (4.1 mg, 0.01 mmol, 10 mol%), K_2CO_3 (41 mg, 0.3 mmol, 3.0 eq.), water (0.4 mL) and toluene (2 mL). The vial was capped with a lid with a Teflon seal and the mixture was degassed with N_2 for 10 min. Then, the resulting mixture was heated to 100 °C and stirred at 100 °C for 16 h. Upon cooling, EtOAc (20 mL) and water (20 mL) were added, and the layers separated. The aqueous layer was extracted with EtOAc (2 × 10 mL). The combined organic layers were dried ($MgSO_4$), filtered and concentrated under reduced pressure. The crude product was purified by flash column chromatography (SiO_2, 75:25 hexane:EtOAc), to afford the *title compound* **S5** as an off white solid (32 mg, 68%).

MP: 146–148 °C;

***R*f:** (7:3 hexane:EtOAc) 0.21;

^{1}H NMR (400 MHz, $CDCl_3$): δ 9.83 (br s, 1H, NH), 8.03 (br s, 1H, NH), 7.65 (dd, *J* = 7.5, 1.5 Hz, 1H, Ar), 7.52–7.44 (m, 4H, Ar), 7.43–7.28 (m, 7H, Ar), 7.24–7.11 (m, 7H, Ar), 7.06–6.98 (m, 2H, Ar), 6.93–6.88 (m, 2H, Ar);

^{13}C NMR (101 MHz, $CDCl_3$): δ 168.6 (C=O), 168.0 (C=O), 139.6 (*ipso*-Ar), 139.6 (*ipso*-Ar), 139.2 (*ipso*-Ar), 138.4 (*ipso*-Ar), 138.0 (*ipso*-Ar), 137.0 (*ipso*-Ar), 136.6 (*ipso*-Ar), 134.5 (*ipso*-Ar), 129.7 (Ar), 129.6 (Ar), 129.5 (Ar), 129.0 (Ar), 128.7 (br, Ar), 128.4 (Ar), 128.2 (Ar), 128.0 (Ar), 127.9 (Ar), 125.1 (Ar), 124.0 (Ar), 121.1 (Ar), 119.7 (Ar), three Ar signals not resolved;

IR (ATR): 3238, 3058, 2923, 2852, 1718, 1644 (C=O), 1597, 1532, 1494, 1442, 1323, 1261, 753, 690, 618, 597, 508 cm^{-1};

HRMS (ESI): calc. for $C_{32}H_{25}NO_4$ $[M + H]^+$: 469.1911; found: 469.1919 (–1.9 ppm).

2-Bromo-*N*, *N*-diphenylbenzamide 1d

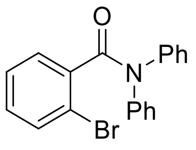

1d

Oxalyl chloride (423 µL, 5.0 mmol, 1.0 eq.) was added to a solution of 2-bromo benzoic acid (1.0 g, 5.0 mmol, 1.0 eq.) and DMF (1 drop) in CH_2Cl_2 (10 mL) at 0 °C. The solution was warmed to rt and stirred for 2 h then concentrated under reduced pressure to provide the 2-bromo benzoyl chloride intermediate. A solution of 2-bromo benzoyl chloride (max. 5 mmol) in CH_2Cl_2 (5 mL) was added dropwise to a stirred solution of diphenylamine (841 mg, 5 mmol, 1.0 eq.) and Et_3N (836 µL, 6 mmol, 1.2 eq.) in CH_2Cl_2 (5 mL) at 0 °C. The mixture was warmed to rt and stirred at rt for 2 h. Water (10 mL) was added, and the layers were separated. The aqueous layer was extracted with CH_2Cl_2 (2 × 10 mL). The combined organic layers were washed with water (10 mL) and brine (10 mL), dried ($MgSO_4$), filtered and concentrated under reduced pressure. The crude product was purified by flash column chromatography (SiO_2, 9:1 to 85:15 hexane:EtOAc), to afford the *title compound* **1d** as a beige solid (0.99 g, 56%).

MP: 111–113 °C;

***R*f:** (9:1 hexane:EtOAc) 0.10;

^{1}H NMR (400 MHz, DMSO-*d*6): δ 7.56 (dd, *J* = 7.5, 1.5 Hz, 1H), 7.51–7.04 (m, 13H);

^{13}C NMR (101 MHz, DMSO-*d*6): δ 167.6 (C=O), 143.4 (*ipso*-Ar), 142.4 (br, *ipso*-Ar), 142.0 (br, *ipso*-Ar), 138.3 (*ipso*-Ar), 132.3 (Ar), 130.5 (Ar), 129.5 (Ar), 129.2 (Ar), 129.1 (br, Ar), 128.5 (br, Ar), 127.5 (br, Ar), 127.1 (Ar), 126.8 (br, Ar), 119.6 (Ar), 118.7 (*ipso*-Ar), 116.7 (Ar);

IR (ATR): 3318, 3055, 1649 (C=O), 1589, 1489, 1347, 1310, 766, 756, 743, 698, 690, 674, 617 cm^{-1};

HRMS (ESI): calc. for $C_{19}H_{15}{}^{79}BrNO$ $[M + H]^+$: 352.0332; found: 352.0329 (+0.7 ppm).

***N2,N2,N2'*-Triphenyl-[1,1'-biphenyl]-2,2'-dicarboxamide S6**

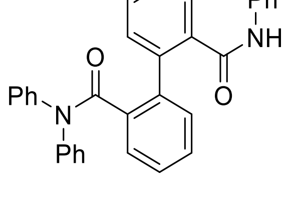

S6

A screw cap 7 mL vial was charged with pinacol boronate **S17** (53 mg, 0.15 mmol, 1.0 eq.), aryl bromide **1d** (48 mg, 0.15 mmol, 1.0 eq.), Pd_2dba_3 (6.8 mg, 0.075 mmol, 5 mol%), Sphos (6.2 mg, 0.015 mmol, 10 mol%), K_2CO_3 (62 mg, 0.45 mmol, 3.0 eq.), water (0.4 mL) and toluene (2 mL). The vial was capped with a lid with a Teflon seal and the mixture was degassed with N_2 for 10 min. Then, the resulting mixture was heated to 100 °C and stirred at 100 °C for 16 h. Upon cooling, EtOAc (20 mL) and water (20 mL) were added, and the layers separated. The aqueous

layer was extracted with EtOAc (2 × 10 mL). The combined organic layers were dried ($MgSO_4$), filtered and concentrated under reduced pressure. The crude product was purified by flash column chromatography (SiO_2, 8:2 hexane:EtOAc), to afford the *title compound* **S6** as an off white solid (40 mg, 67%).

MP: 190–192 °C;

***R*f:** (8:2 hexane:EtOAc) 0.11;

^{1}H NMR (400 MHz, $CDCl_3$): δ 9.80 (br s, 1H, NH), 7.77 (dd, *J* = 7.5, 1.5 Hz, 1H, Ar), 7.52 (td, *J* = 7.5, 1.5 Hz, 1H, Ar), 7.47 (td, *J* = 7.5, 1.5 Hz, 1H, Ar), 7.41–7.13 (m, 18H, Ar), 7.07 (dt, *J* = 8.0, 4.5 Hz, 1H, Ar), 7.00–6.93 (m, 1H, Ar);

^{13}C NMR (101 MHz, $CDCl_3$): δ 171.2 (C=O), 167.6 (C=O), 143.0 (br, *ipso*-Ar), 142.6 (br, *ipso*-Ar), 140.2 (*ipso*-Ar), 138.6 (*ipso*-Ar), 137.9 (*ipso*-Ar), 134.2 (*ipso*-Ar), 133.3 (*ipso*-Ar), 130.9 (Ar), 129.9 (Ar), 129.5 (Ar), 129.4 (br, Ar), 129.3 (Ar), 128.6 (Ar), 128.6 (Ar), 128.4 (Ar), 128.1 (Ar), 127.5 (br, Ar), 127.2 (Ar), 126.8 (br, Ar), 123.8 (Ar), 120.0 (Ar);

IR (ATR): 3245, 3031, 2924, 1660 (C=O), 1627 (C=O), 1591, 1548, 1490, 1442, 1364, 1322, 751, 705, 690, 622, 513 cm^{-1};

HRMS (ESI): calc. for $C_{32}H_{25}N_2O_2$ $[M + H]^+$: 469.1911; found: 469.1925 (–3.1 ppm).

2.7. Mechanistic Studies by ^{31}P NMR and Mass Spectrometry Experiments

This section of the study involves the partial characterization of intermediates, mixtures and/or unstable species. Therefore, selected data (given below) is provided.

2.7.1 Identification of the active Pd^0 catalytic Species Pd(dppe) I

Stoichiometric ^{31}P NMR studies were performed to identify the active palladium species entering the catalytic cycle. Supplementary Figure 14 shows the ^{31}P NMR spectra from the three following reactions (1-3):

1. 1:1 $Pd(OAc)_2$ and dppe in DMF

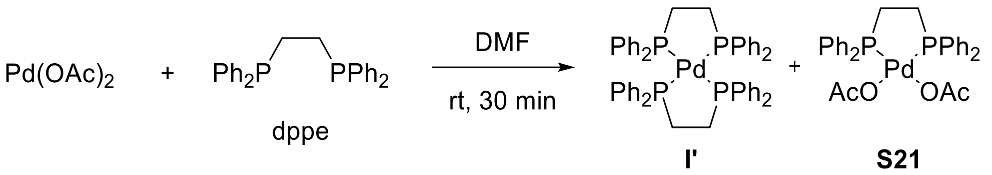

Dry, degassed DMF (1 mL) was added to $Pd(OAc)_2$ (10 mg, 0.04 mmol, 1.0 eq.) and dppe (18 mg, 0.04 mmol, 1.0 eq.) under N_2 and stirred at rt for 30 minutes. A 0.5 mL aliquot was removed from the mixture and placed in a dry Youngs NMR tube under N_2. Then, dry acetone-d_6 (0.2 mL) was added and the mixture analysed by ^{31}P NMR (Supplementary Figure 14).

^{31}P NMR (162 MHz, $(CD_3)_2CO$): δ 59.1 (s), 30.1 (s), 30.0 (s).

2. 1:2 Pd_2dba_3 and dppe in DMF

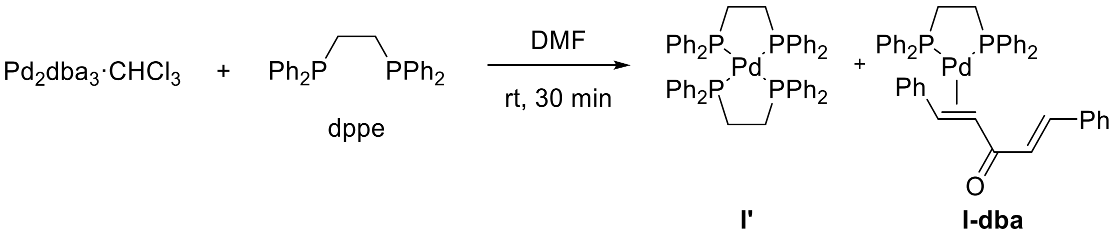

Dry, degassed DMF (1 mL) was added to $Pd_2dba_3 \cdot CHCl_3$ (10 mg, 0.01 mmol, 1.0 eq.) and dppe (8.7 mg, 0.02 mmol, 2.0 eq.) under N_2 and stirred at rt for 30 minutes. A 0.5 mL aliquot was removed from the mixture and placed in a dry Youngs NMR tube under N_2. Then, dry acetone-d_6 (0.2 mL) was added and the mixture analysed by ^{31}P NMR (Supplementary Figure 14).

^{31}P NMR (162 MHz, $(CD_3)_2CO$): δ 65.9 (s), 56.7 (s), 37.0 (br s), 35.5 (br s), 30.0 (s).

3. 1:1:1 $Pd(OAc)_2$, dppe and dba in DMF

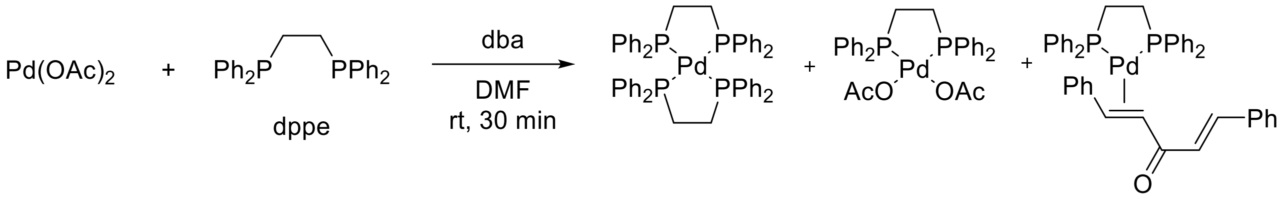

Dry, degassed DMF (1 mL) was added to $Pd(OAc)_2$ (10 mg, 0.04 mmol, 1.0 eq.) and dppe (18 mg, 0.04 mmol, 1.0 eq.) and dba (10 mg, 0.04 mmol, 1.0 eq.) under N_2 and stirred at rt for 30 minutes. A 0.5 mL aliquot was removed from the mixture and placed in a dry Youngs NMR tube under N_2. Then, dry acetone-d_6 (0.2 mL) was added and the mixture analysed by ^{31}P NMR (Supplementary Figure 14).

^{31}P NMR (162 MHz, $(CD_3)_2CO$): δ 59.1 (s), 37.1 (br s), 35.6 (br s), 30.1 (s), 29.9 (s), 29.8 (s), 29.6 (s), 29.5 (s).

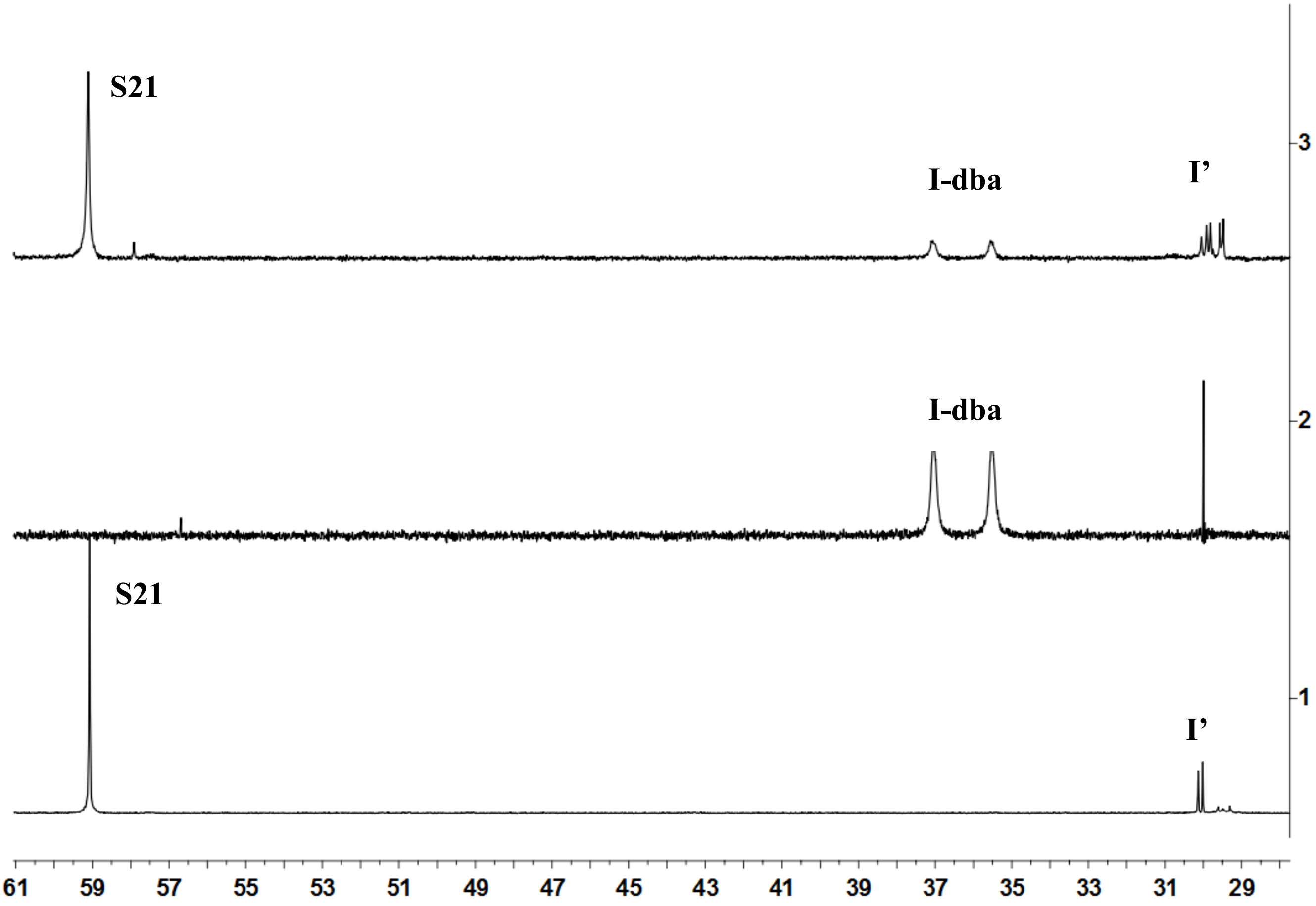

Supplementary Figure 14. ^{31}P NMR (162 MHz, $(CD_3)_2CO$) spectra of catalytic species formed from of precatalysts and ligands. (1) Spectrum of reaction 1 between $Pd(OAc)_2$ and dppe; (2) Spectrum of reaction 2 between $Pd_2dba_3 \cdot CHCl_3$ and dppe; (3) Spectrum of reaction 3 between $Pd(OAc)_2$, dppe and dba.

Supplementary Figure 14-1 shows two singlet peaks at 30.1 and 30.0 ppm. As these are both singlets, the phosphorus atoms must be equivalent. One of these peaks can be assigned to $Pd(dppe)_2$ **I’** which is reported in the literature as a singlet peak at 30.46 ppm in THF.[36] The other peak could possibly be oxidised phosphine ligand (dppeO) which in the literature is reported at 33.2 ppm in $CDCl_3$.[37] The peak at 59.07 ppm is assigned to $Pd(dppe)(OAc)_2$ **S21**. This compound has been synthesised, showing a peak at 58.9 ppm in CH_2Cl_2.[38]

Supplementary Figure 14-2 shows two broad singlets (most likely two unresolved doublets) in the ^{31}P NMR spectrum at 35.5 (FWHM = 28.6 Hz) and 37.0 (FWHM = 28.3 Hz) ppm. These signals correspond to compound **I-dba**. This compound is well documented in the literature with ^{31}P NMR peaks reported at 37.8 and 36.4 ppm in DMSO-d_6[39] and 34.41 and 36.63 ppm in THF.[36]

Reaction 3 aimed to bring the previous two reactions together by the addition of dba to $Pd(OAc)_2$ and dppe. The aim was to trap any catalytically active Pd species formed from $Pd(OAc)_2$ and dppe with a dba ligand. Supplementary Figure 14-3 indicates that this was successful with the two broad singlets seen at 35.5 (FWHM = 26.4 Hz) and 37.0 (FWHM = 27.9 Hz) ppm. A peak at 59.11 ppm was also seen for compound **V**. From these studies it be deduced that the likely active catalyst is the Pd^0 complex Pd(dppe) **I** (shown in Figure 9 in the manuscript). Pathways for pre-catalyst activation are shown below in Supplementary Figure 15.

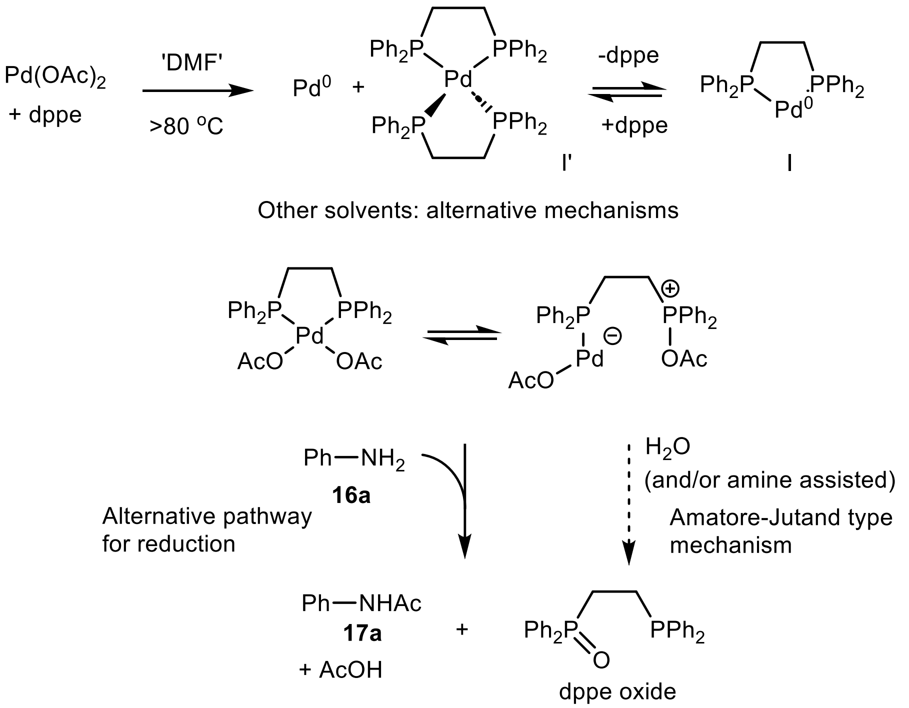

* other solvents may have different modes of pre-catalyst activation, including the generation of dppeO (mono-oxide) ligand.

Supplementary Figure 15. Pathways for $Pd(OAc)_2$ activation by dppe in various solvents.

2.7.2 Identification of Oxidative Addition products by ^{31}P NMR and HRMS

The reaction of 2-bromobenzamide **1a** with stoichiometric Pd in the absence of base was performed and followed by ^{31}P NMR experiment to identify intermediates. This reaction was conducted in DMF-d_7 with $Pd_2dba_3{\cdot}CHCl_3$ and dppe as the precatalyst (Supplementary Figure 16).

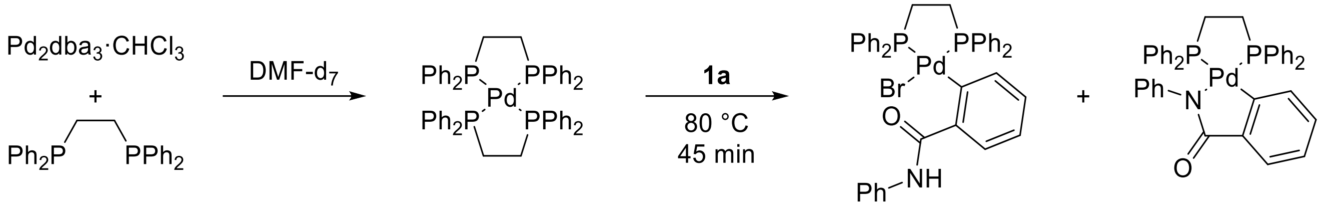

I' **II** **S22**

Supplementary Figure 16. Generation of $Pd(dppe)_2$ and reactivity in oxidative addition.

All manipulations were performed in a glovebox to ensure a rigorous nitrogen atmosphere was maintained. Supplementary Figure 17 shows the ^{31}P NMR spectra at several timepoints.

Degassed DMF-d_7 (0.5 mL) was added to $Pd_2dba_3{\cdot}CHCl_3$ (5.0 mg, 5.5 μmol, 1.0 eq.) and dppe (4.3 mg, 0.011 mmol, 2.0 eq.) under N_2 in a Youngs NMR tube. The sample was analysed by ^{31}P NMR. Then, 2-bromo-*N*-phenylbenzamide (3.0 mg, 0.011 mmol, 1.0 eq.) was added to the catalyst system under N_2 and a ^{31}P NMR spectrum recorded. The reaction was then heated to 80 °C for 45 minutes and a ^{31}P NMR spectrum recorded before further heating for 16 hours and analysis by ^{31}P NMR.

Spectrum 1: **^{31}P NMR (203 MHz, DMF-d_7):** δ 57.4 (s), 36.6 (d, J = 6.0 Hz), 35.1 (d, J = 6.0 Hz), 29.4 (s).

Spectrum 2: **^{31}P NMR (203 MHz, DMF-d_7):** δ 57.4 (s), 36.6 (br s), 35.1 (br s), 29.4 (s).

Spectrum 3: **^{31}P NMR (203 MHz, DMF-d_7):** δ 65.8 (s), 59.1 (d, J = 28 Hz), 56.8 (s), 41.8 (d, J = 28 Hz), 36.6 (br s), 35.1 (br s), 29.4 (s).

Spectrum 4: **^{31}P NMR (203 MHz, DMF-d_7):** δ 65.8 (s), 59.1 (d, J = 28 Hz), 56.6 (s), 41.8 (d, J = 28 Hz).

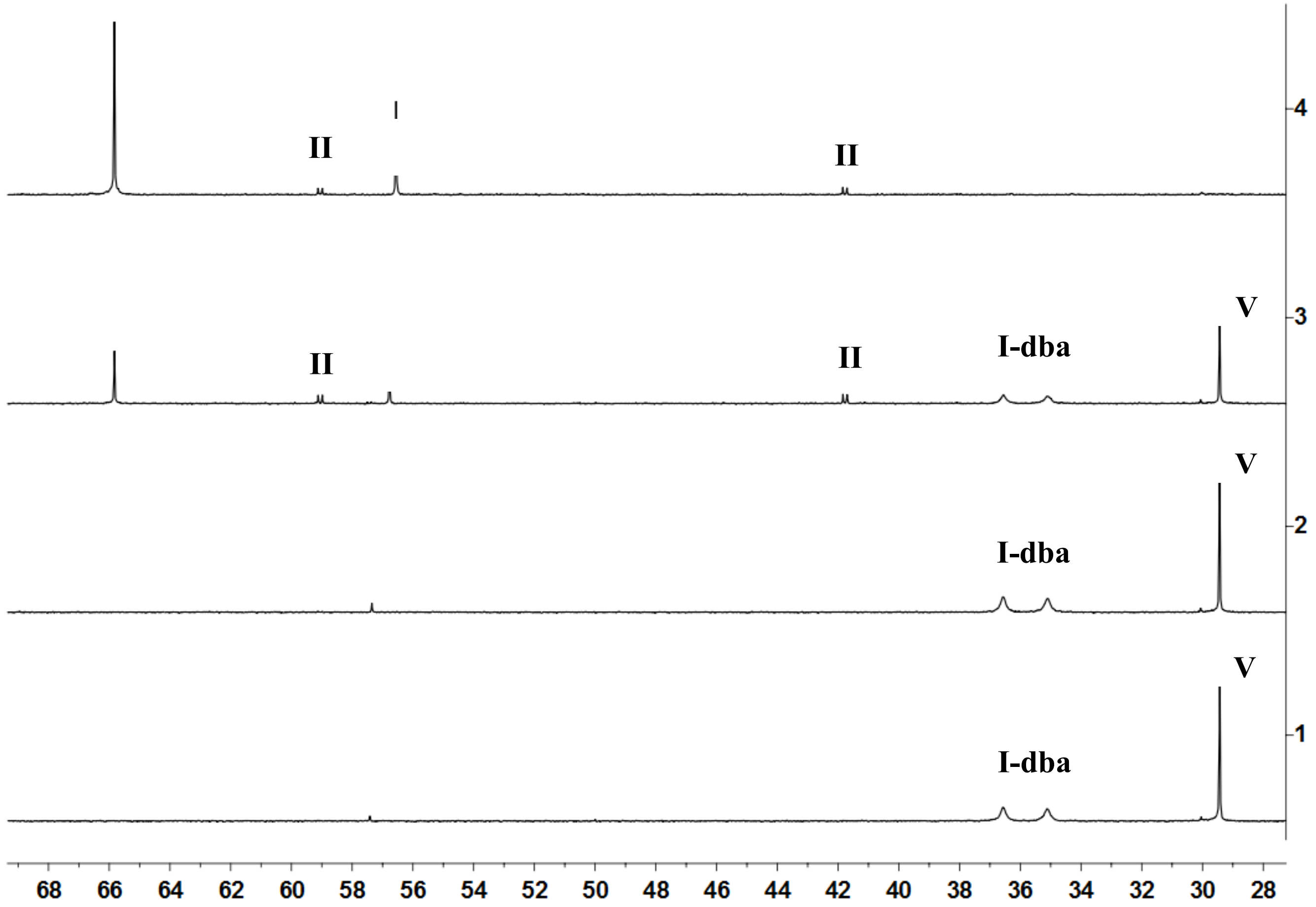

Supplementary Figure 17. ^{31}P NMR (203 MHz, DMF-*d*$_7$) spectra of the reaction of 2-bromo-*N*-phenylbenzamide **1a** with $Pd_2dba_3 \cdot CHCl_3$ and dppe recorded at (1) rt, t = 0 minutes; (2) after addition of substrate at rt, t = 5 minutes; (3) 45 mins, heating at 80 °C; (4) 16 hours heating at 80 °C.

Supplementary Figure 17-1 shows the catalyst system and. The peak at 29.43 ppm corresponds to the $Pd(dppe)_2$ complex **I'**. Two doublets with a coupling constant of 6 Hz were observed at 35.10 and 36.56 ppm, corresponding to the two phosphorus atoms in the Pd(dppe)(dba) complex **I-dba**. A small fourth peak was seen at 57.42 ppm which was an unknown phosphorus species.

After addition of 2-bromo-*N*-phenylbenzamide **1a**, the same peaks were observed immediately after addition of the substrate (Supplementary Figure 17-2). A spectrum was recorded after 45 minutes heating at 80 °C (Supplementary Figure 17-3). Two sets of doublets appeared at 41.8 and 59.1 ppm with a coupling constant of 28 Hz. These are presumed to be the two phosphorus atoms in Pd(Ar)Br(dppe) **II**. ^{31}P NMR signals for oxidative addition products of this type tend to be found around 50 and 35 ppm.[40,41] A higher chemical shift would be expected due to the electron-withdrawing nature of the amide carbonyl. The previously reported catalyst peaks were observed along with a new singlet peak at 65.8 ppm. The

peak observed previously at 57.4 ppm had either shifted to 56.8 ppm and increased to 10% of total phosphorus or had disappeared and a new species formed.

After 16 hours heating (Supplementary Figure 17-4), the original complex peaks at 29.4, 35.1 and 36.6 ppm had disappeared, leaving only the two doublets of **II** at 41.8 and 59.1 ppm, and two singlets at 56.6 and 65.8 ppm. The doublet peaks were small, only 4% of the total phosphorus observed. It was concluded from this final ^{31}P NMR spectrum that the singlet peaks at 56.6 and 65.8 ppm, which grew significantly over the course of the reaction were degradation products. The NMR tube contained black precipitate (Pd black) and so the peaks likely correspond to free phosphorus species in solution. The reaction was repeated with a ^{31}P NMR spectrum taken after 45 minutes heating. The reaction was then opened to air and one drop of the solution was removed, diluted in MeCN and analysed by ESI-MS. Ten peaks showing Pd isotope patterns were detected (Supplementary Figure 18), three (**I'**, **S22** and **VI**) of which have been identified:

HRMS (ESI): **I'** calc. for $C_{52}H_{48}P_4Pd$ $[M]^{2+}$: 451.0865; found 451.0852 (–2.9 ppm); **S22** calc. for $C_{39}H_{34}NOP_2Pd$ $[M + H]^+$: 700.1159; found 700.1130 (+4.6 ppm); **VI** calc. for $C_{52}H_{42}N_2NaO_2P_2Pd$ $[M + Na]^+$: 917.1667; found 917.1606 (+6.3 ppm);

Complex **I'** is the palladium species seen in the NMR studies formed when the Pd precatalyst reacts with dppe. Complex **S22** is formed when the substrate oxidatively adds to the active catalyst, followed by formation of a palladacycle and elimination of HBr. It is possible that complex **S22** is formed via loss of HBr in the mass spectrometer from complex **II** and it is actually complex **II** in solution. Complex **VI** is the proposed structure for the peak seen at 917 m/z.

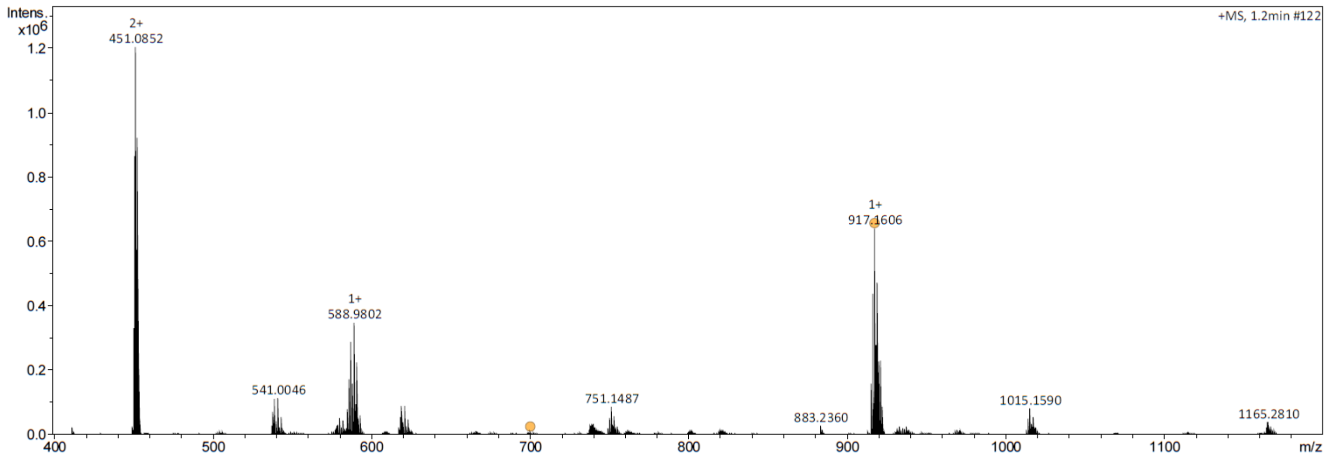

Supplementary Figure 18. ESI-Mass spectrum of the reaction of 2-bromo-*N*-phenyl-benzamide **1a** with $Pd_2dba_3 \cdot CHCl_3$ and dppe.

Supplementary Figure 19 shows the comparison between experimental and theoretical isotope distribution[42] for each of species **I´**, **S22** and **VI**. Each observed pattern matching the theoretical values satisfactorily, supporting the proposed structures for these Pd species.

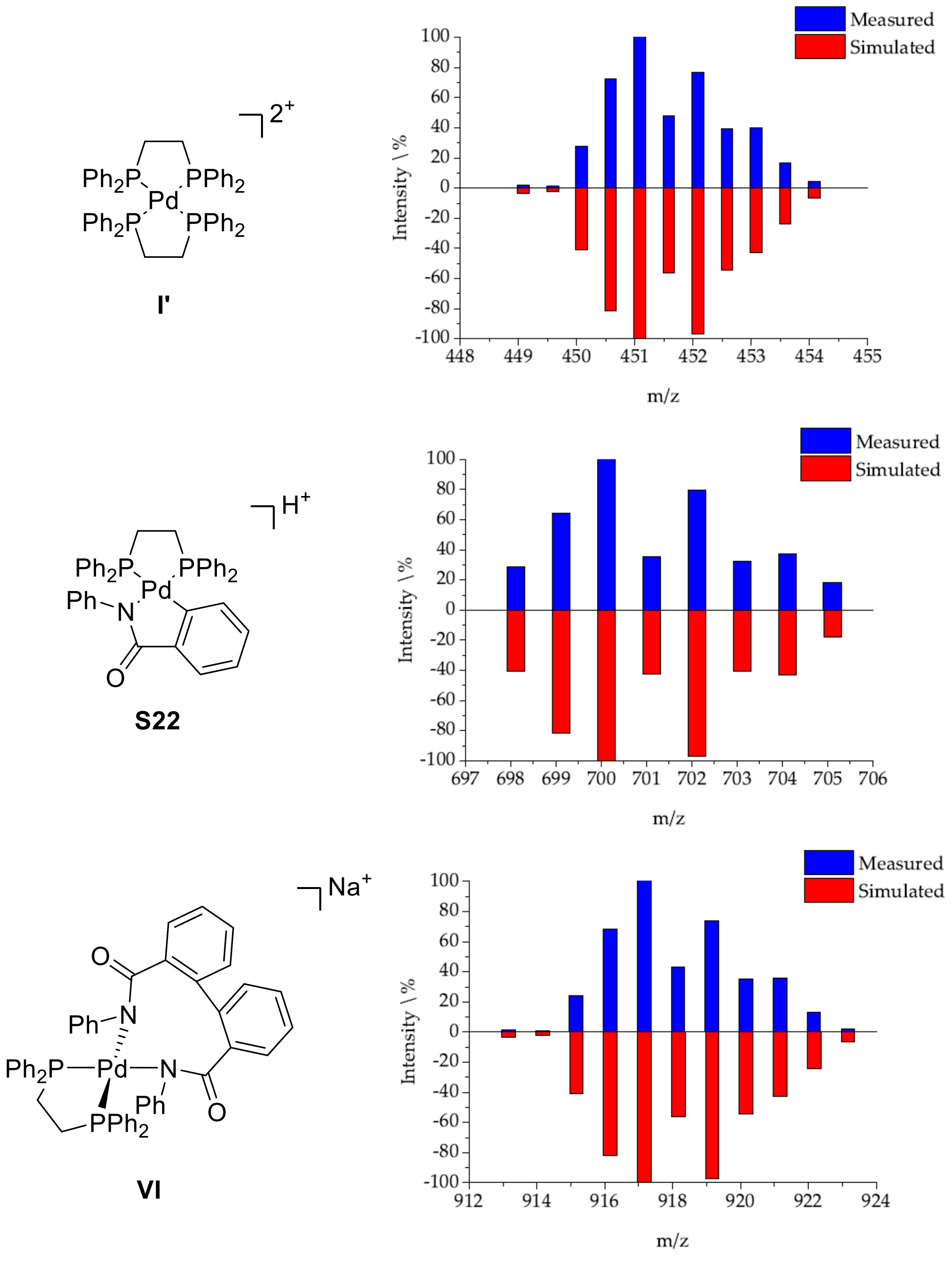

Supplementary Figure 19. Measured and theoretical isotope patterns and proposed structures for **I'**, **S22** and **VI**, observed in the reaction mixture of 2-bromo-*N*-phenyl-benzamide **1a** with $Pd_2dba_3{\cdot}CHCl_3$ and dppe in DMF-d_7 by ESI-MS after 45 minutes heating at 80 °C.

3. Supplementary Discussion

3.1 Principal component analysis of unscaled data

Supplementary Figure 20 shows scores plots for the first two principal components obtained using unscaled data. The loadings show that differences along PC1, accounting for 97.3% of the variance in the data, are related to differences between 2-bromo-benzamide **1a**, the starting material, and product **2a**, the major product. While DMF and propylene carbonate gave the greatest amount of product **2a**, regardless of reaction time or temperature, Bu_2O led to the most **1a** remaining, with a slow but steady increase in product **2a** with increasing reaction time. Other solvents, notably toluene, show a dependence on reaction time with most reactions in the centre of the plot. Although responsible for little variance in the data, PC2 shows differences in minor product quantities with MeCN and MEK resulting in most side-products, particularly symmetrical bis-amide **4a**. The dual solvent system, Bu_2O:DMF (9:1), leads to more major product **2a** than Bu_2O but less than DMF and also more side-products. Most BuCN reactions show little **1a** remaining but vary in the proportion of major product **2a** to side products.

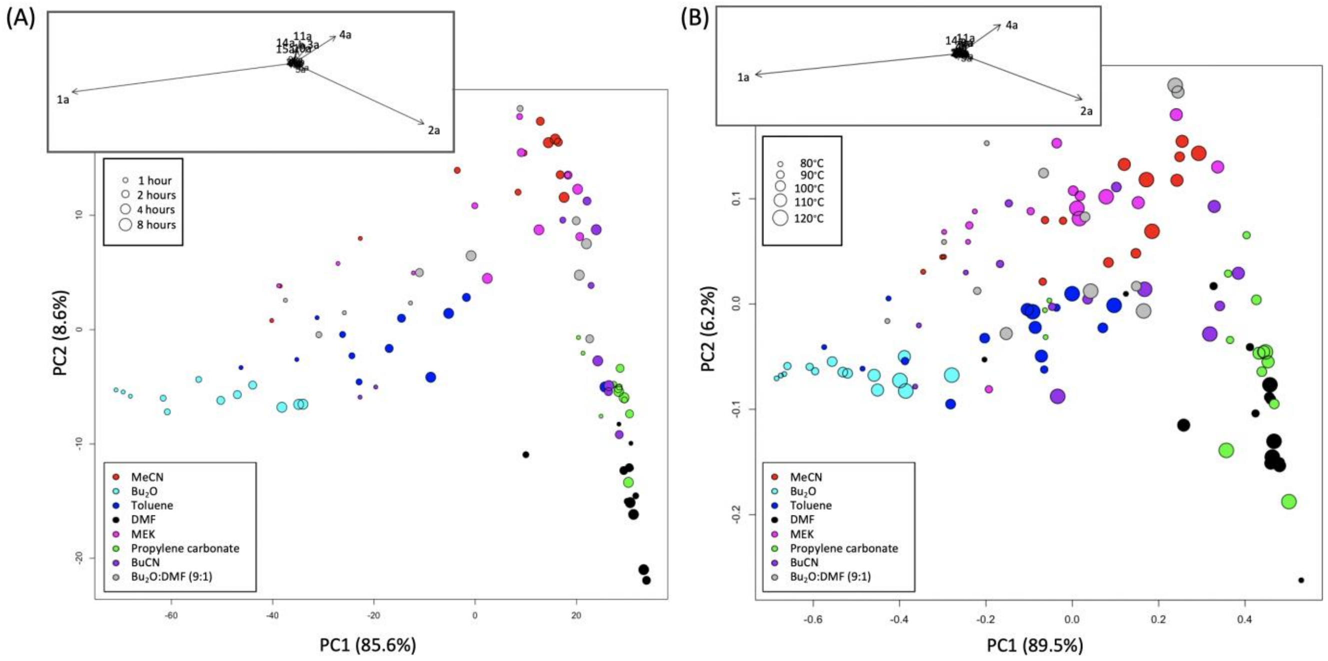

Supplementary Figure 20. Scores plots for the first two principal components obtained using unscaled data. (A) shows reactions performed with 8 different solvents at 4 different reaction times. (B) shows reactions performed with 8 different solvents at 5 different temperatures. The loadings, shown as vectors in the insets, indicate the contribution of the various products to the principal components.

3.2 Principal component analysis of all Times and Temperature

Supplementary Figure 21 shows scores plots for the first two principal components for all reactions performed with 8 different solvents, 4 different reaction times and 5 different temperatures after scaling to unit-variance

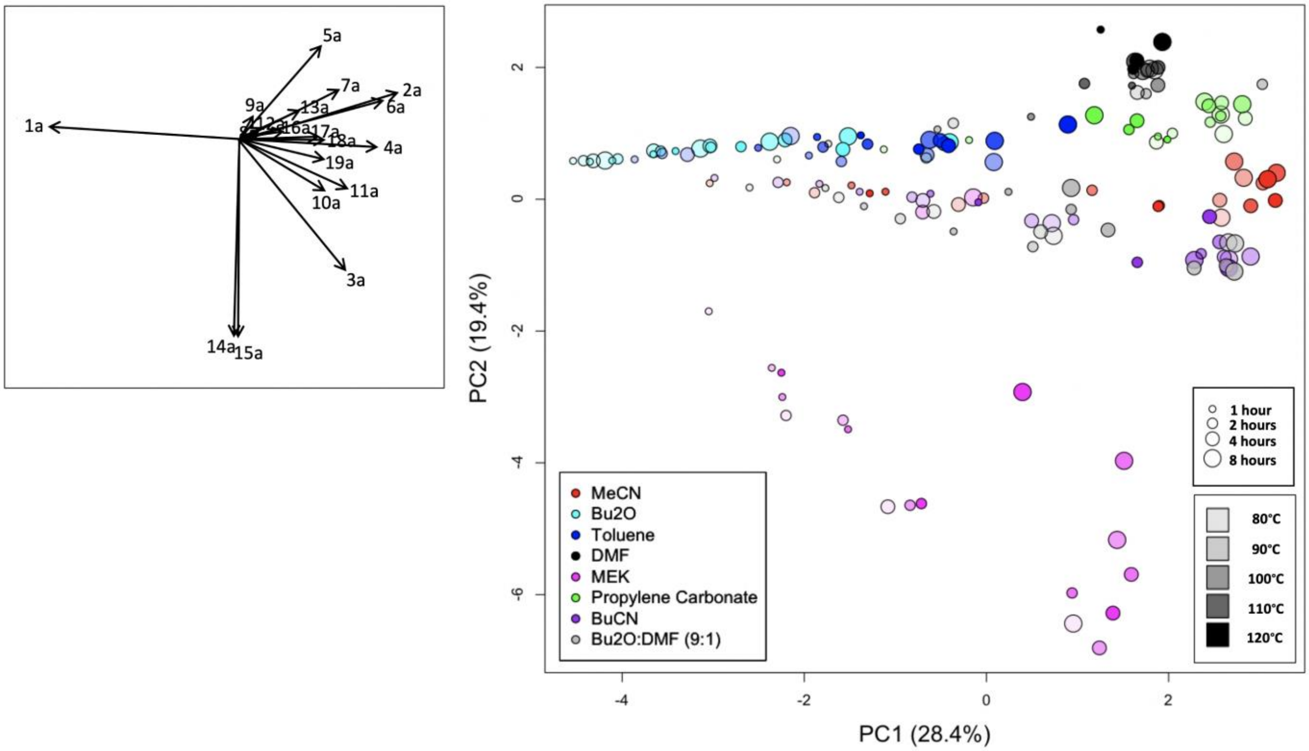

Supplementary Figure 21. Scores plots for the first two principal components for reactions performed with 8 different solvents, 4 different reaction times and 5 different temperatures after scaling to unit-variance. The loadings, shown as vectors on the inset on the left, indicate the contribution of the various products to the principal components.

3.3 X-Ray Crystallographic Data for Single Crystals

Supplementary Table 4. Summary data taken from .cif files for all single crystal X-ray diffraction structures (note: all deposited to the Cambridge Crystallographic Data Centre, CCDC; identification codes given in the table.

**Compound**	**2a**	**2b**	**4a**
CCDC number	2063167	2063164	2063166
Identification code	ijsf1415	ijsf1521	ijsf1607
Empirical formula	$C_{19}H_{13}NO$	$C_{22}H_{18}Cl_3NO$	$C_{26}H_{20}N_2O_2$
Formula weight	271.30	418.72	392.44
Temperature/K	110.05(10)	109.9(2)	110.0(3)
Crystal system	monoclinic	monoclinic	tetragonal
Space group	$P2_1/c$	$P2_1/c$	$P4_32_12$
a/Å	5.4029(3)	12.15159(18)	10.71833(9)
b/Å	23.0685(11)	11.14359(18)	10.71833(9)
c/Å	10.6735(6)	14.5618(2)	35.3505(6)
α/°	90	90	90
β/°	102.643(5)	92.0114(14)	90
γ/°	90	90	90
Volume/Å^3	1298.05(12)	1970.64(5)	4061.16(10)
Z	4	4	8
ρ_{calc}mg/mm^3	1.388	1.411	1.284
m/mm^{-1}	0.086	4.300	0.651
F(000)	568.0	864.0	1648.0
Crystal size/mm^3	0.2809 × 0.2072 × 0.1389	0.192 × 0.139 × 0.12	0.288 × 0.274 × 0.091
Radiation	MoKα (λ = 0.7107)	CuKα (λ = 1.54184)	CuKα (λ = 1.54184)
2Θ range for data collection	6.58 to 60°	9.998 to 134.156	8.62 to 142.29
Index ranges	$-4 \le h \le 7$, $-14 \le k \le 32$, $-15 \le l \le 11$	$-14 \le h \le 11$, $-11 \le k \le 13$, $-10 \le l \le 17$	$-12 \le h \le 11$, $-12 \le k \le 12$, $-43 \le l \le 35$
Reflections collected	6611	7076	15290

Independent reflections	3781 [R_{int} = 0.0212, R_{sigma} = 0.0356]	3519 [R_{int} = 0.0189, R_{sigma} = 0.0240]	3845 [R_{int} = 0.0220, R_{sigma} = 0.0205]
Data/restraints/parameters	3781/0/190	3519/0/246	3845/0/279
Goodness-of-fit on F^2	1.057	1.060	1.048
Final R indexes [I>=2σ (I)]	R_1 = 0.0479, wR_2 = 0.1222	R_1 = 0.0359, wR_2 = 0.0884	R_1 = 0.0276, wR_2 = 0.0675
Final R indexes [all data]	R_1 = 0.0594, wR_2 = 0.1322	R_1 = 0.0393, wR_2 = 0.0911	R_1 = 0.0295, wR_2 = 0.0686
Largest diff. peak/hole / e $Å^{-3}$	0.34/-0.27	0.51/-0.69	0.13/-0.17
Flack parameter			-0.04(8)

**Compound**	**7a**	**5a**	**S3**
CCDC number	2063165	2081723	2081724
Identification code	ijsf1507	ijsf21029	ijsf21030
Empirical formula	$C_{19}H_{11}NO$	$C_{26}H_{18}N_2O_2$	$C_{26}H_{18}N_2O_2$
Formula weight	269.29	390.42	390.42
Temperature/K	110.05(10)	110.00(10)	110.00(10)
Crystal system	orthorhombic	monoclinic	monoclinic
Space group	$P2_12_12_1$	$P2_1/c$	$P2_1/c$
a/Å	6.12041(14)	9.3160(2)	12.363(2)
b/Å	13.4413(3)	26.1205(5)	10.8150(17)
c/Å	15.2868(4)	8.4571(2)	15.866(2)
α/°	90	90	90
β/°	90	115.801(3)	112.054(19)
γ/°	90	90	90
Volume/$Å^3$	1257.59(5)	1852.78(8)	1966.2(6)
Z	4	4	4
ρ_{calc}mg/mm^3	1.422	1.400	1.319
m/mm^{-1}	0.697	0.713	0.672
F(000)	560.0	816.0	816.0
Crystal size/mm^3	0.2138 × 0.1642 × 0.1202	0.181 × 0.062 × 0.023	0.113 × 0.04 × 0.01
Radiation	CuKα (λ = 1.54184)	Cu Kα (λ = 1.54184)	Cu Kα (λ = 1.54184)

2Θ range for data collection	8.76 to 142.408	10.548 to 134.16	7.716 to 134.16
Index ranges	$-6 \le h \le 7$, $-16 \le k \le 14$, $-18 \le l \le 18$	$-11 \le h \le 5$, $-29 \le k \le 31$, $-10 \le l \le 10$	$-14 \le h \le 13$, $-12 \le k \le 9$, $-18 \le l \le 17$
Reflections collected	8225	6860	6700
Independent reflections	2407 [R_{int} = 0.0302, R_{sigma} = 0.0249]	3301 [R_{int} = 0.0185, R_{sigma} = 0.0278]	3499 [R_{int} = 0.0700, R_{sigma} = 0.1184]
Data/restraints/parameters	2407/3/206	3301/0/343	3499/0/271
Goodness-of-fit on F^2	1.123	1.022	0.970
Final R indexes [I>=2σ (I)]	R_1 = 0.0342, wR_2 = 0.0845	R_1 = 0.0374, wR_2 = 0.0940	R_1 = 0.0696, wR_2 = 0.1588
Final R indexes [all data]	R_1 = 0.0349, wR_2 = 0.0851	R_1 = 0.0483, wR_2 = 0.0998	R_1 = 0.1312, wR_2 = 0.1966
Largest diff. peak/hole / e $Å^{-3}$	0.12/-0.24	0.25/-0.27	0.42/-0.34
Flack parameter	-0.45(17)		

3.4 Representative NMR Spectra of Compounds

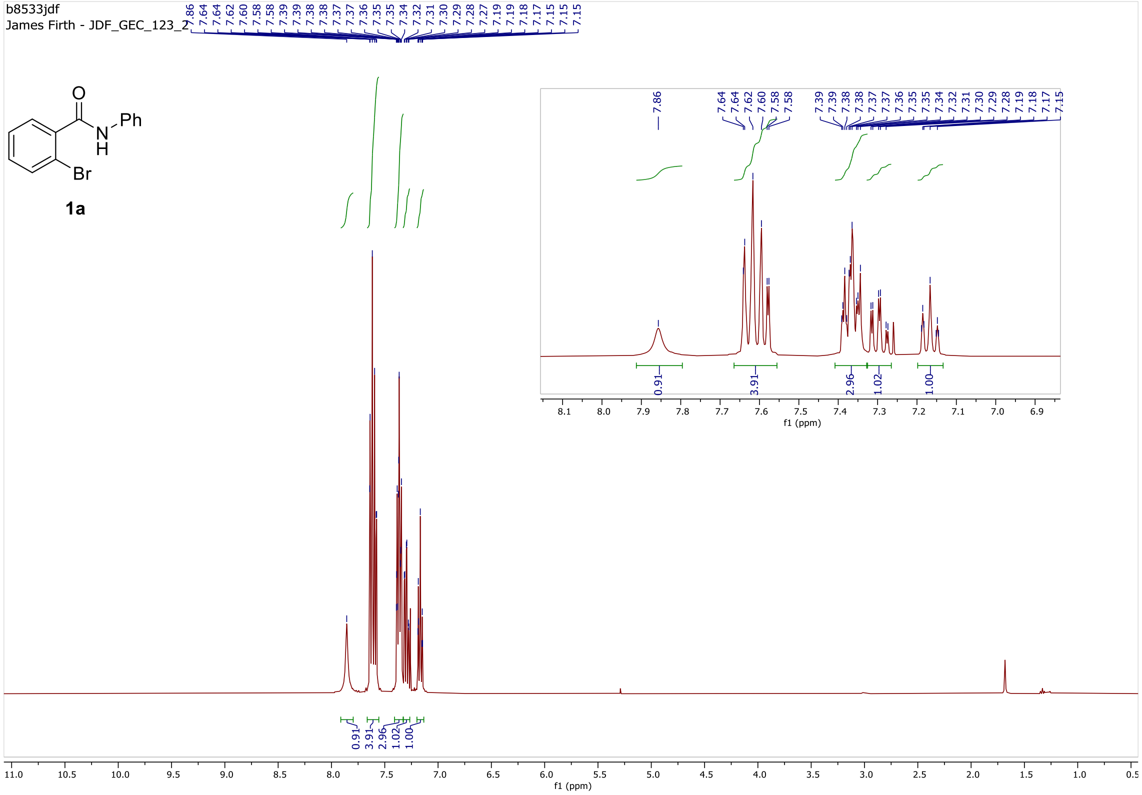

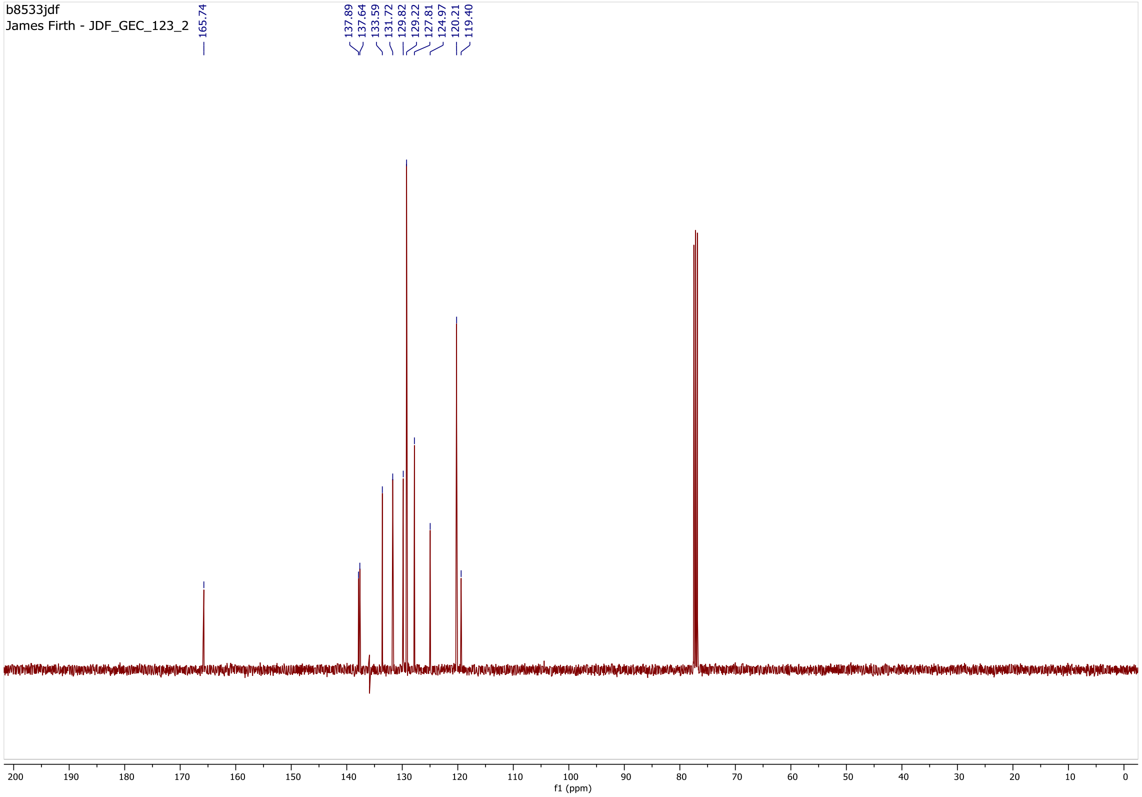

Supplementary Figure 22. Top: ^{1}H NMR spectrum of **1a** (400 MHz, 298 K, $CDCl_3$). Bottom: ^{13}C NMR spectrum of **1a** (101 MHz, 298 K, $CDCl_3$).

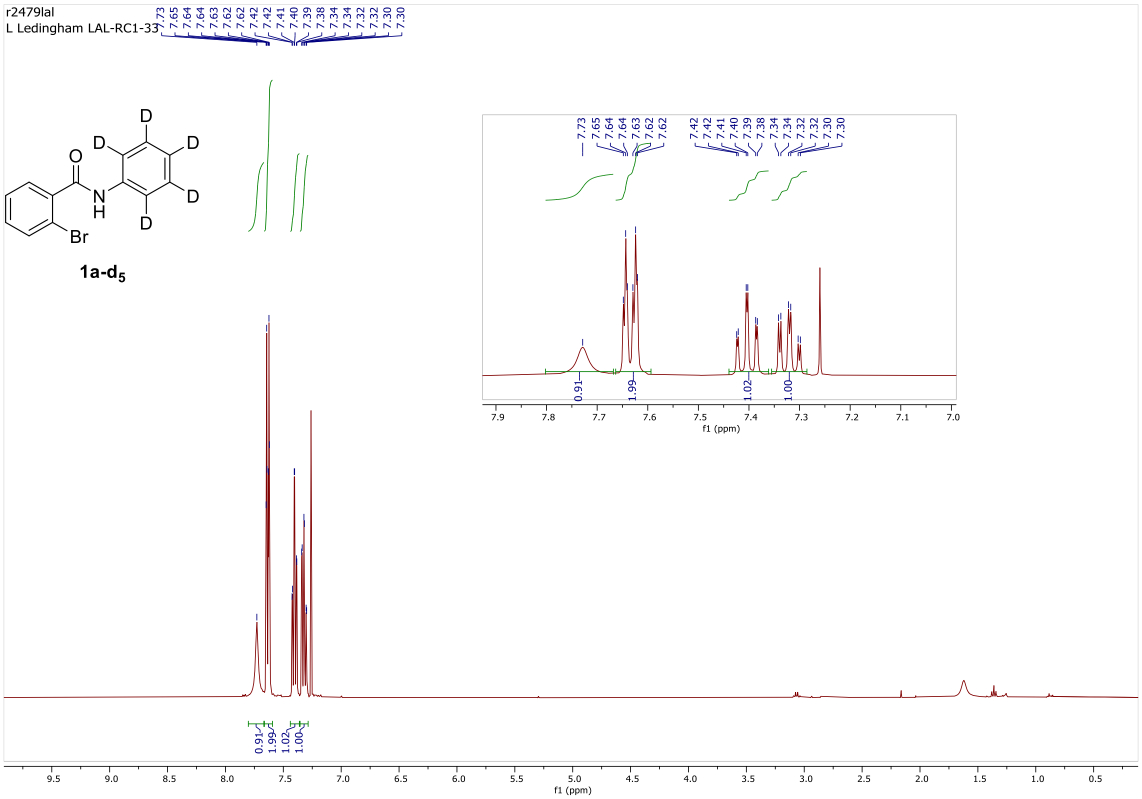

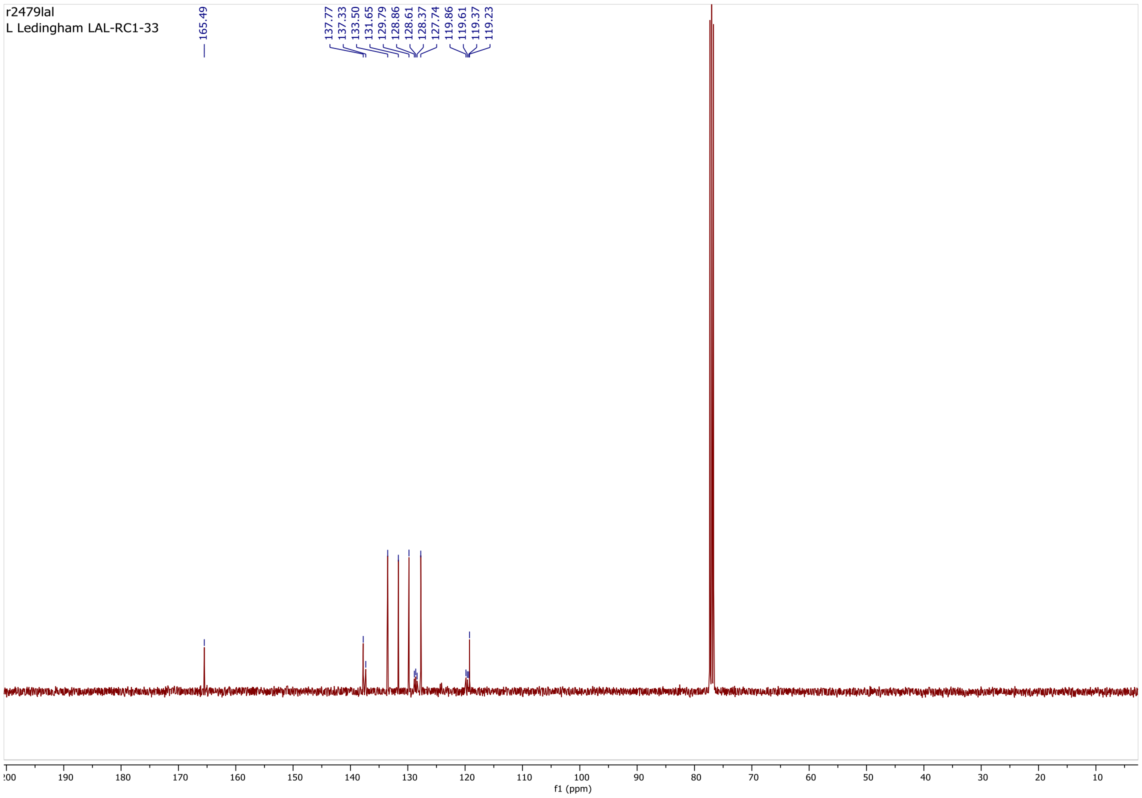

Supplementary Figure 23. Top: ^{1}H NMR spectrum of **1a-d$_5$** (400 MHz, 298 K, $CDCl_3$). Bottom: ^{13}C NMR spectrum of **1a-d$_5$** (101 MHz, 298 K, $CDCl_3$).

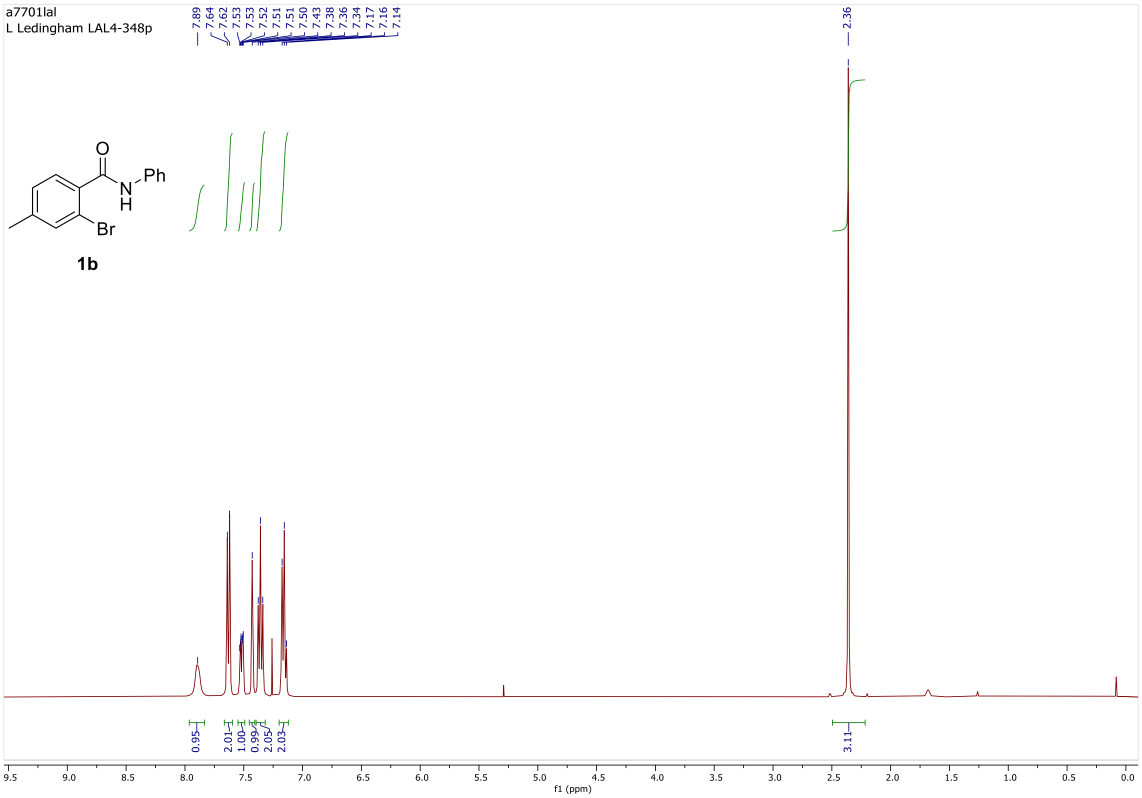

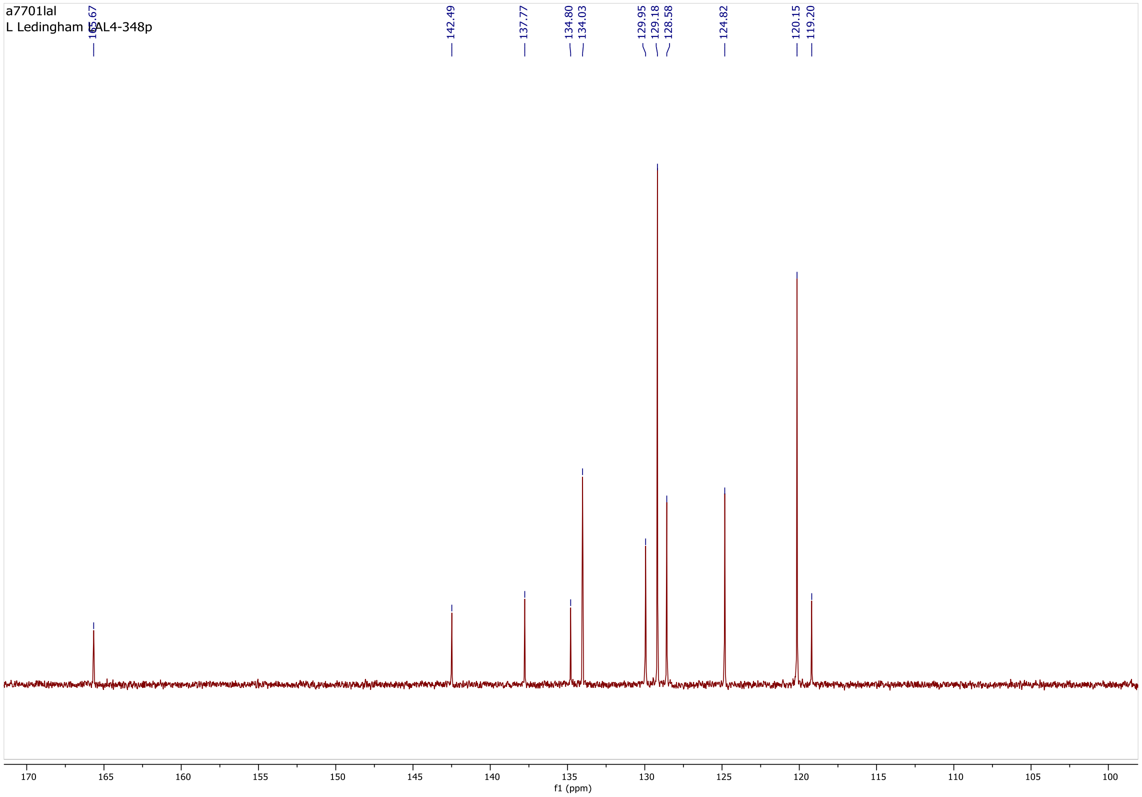

Supplementary Figure 24. Top: ^{1}H NMR spectrum of **1b** (400 MHz, 298 K, $CDCl_3$). Bottom: ^{13}C NMR spectrum of **1b** (101 MHz, 298 K, $CDCl_3$).

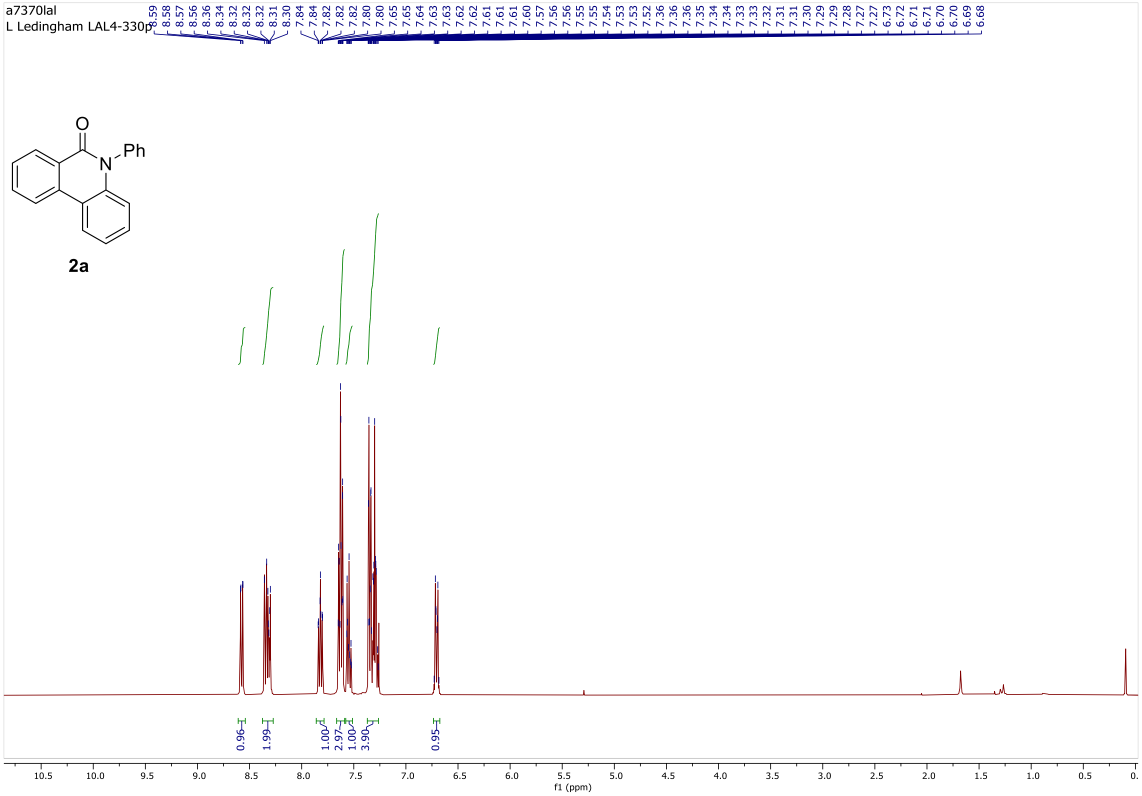

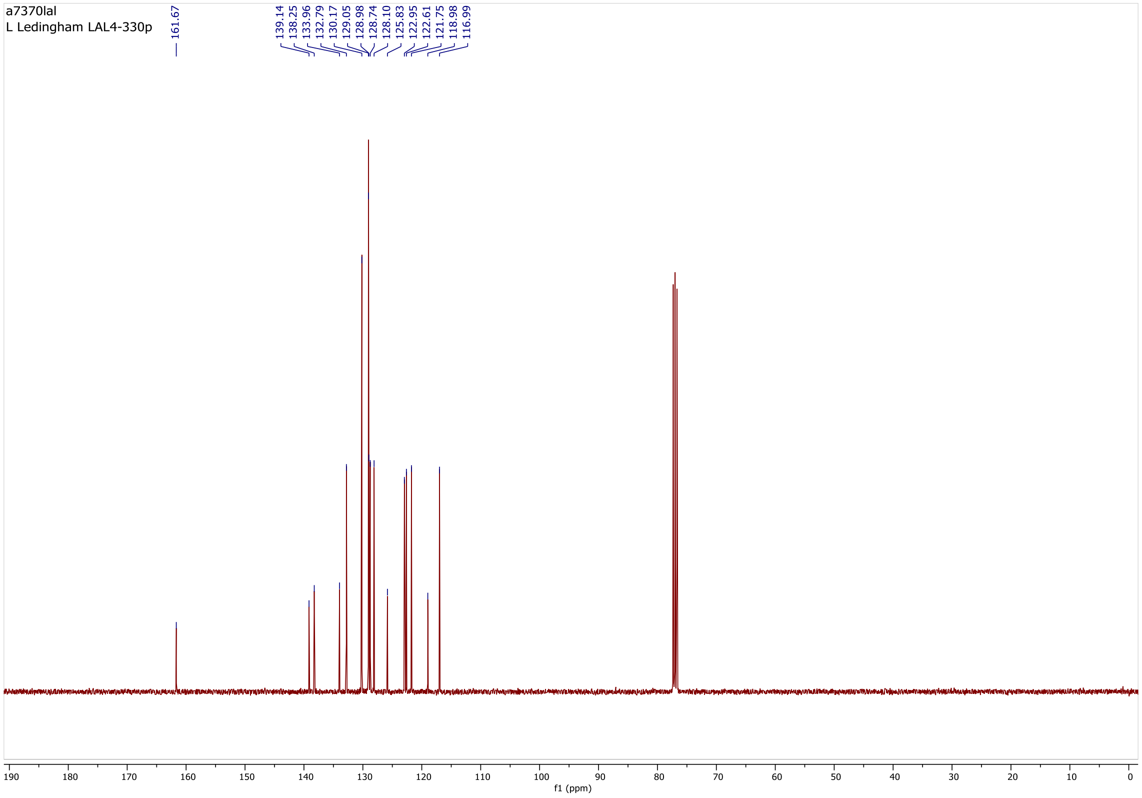

Supplementary Figure 25. Top: ^{1}H NMR spectrum of **2a** (400 MHz, 298 K, $CDCl_3$). Bottom: ^{13}C NMR spectrum of **2a** (101 MHz, 298 K, $CDCl_3$).

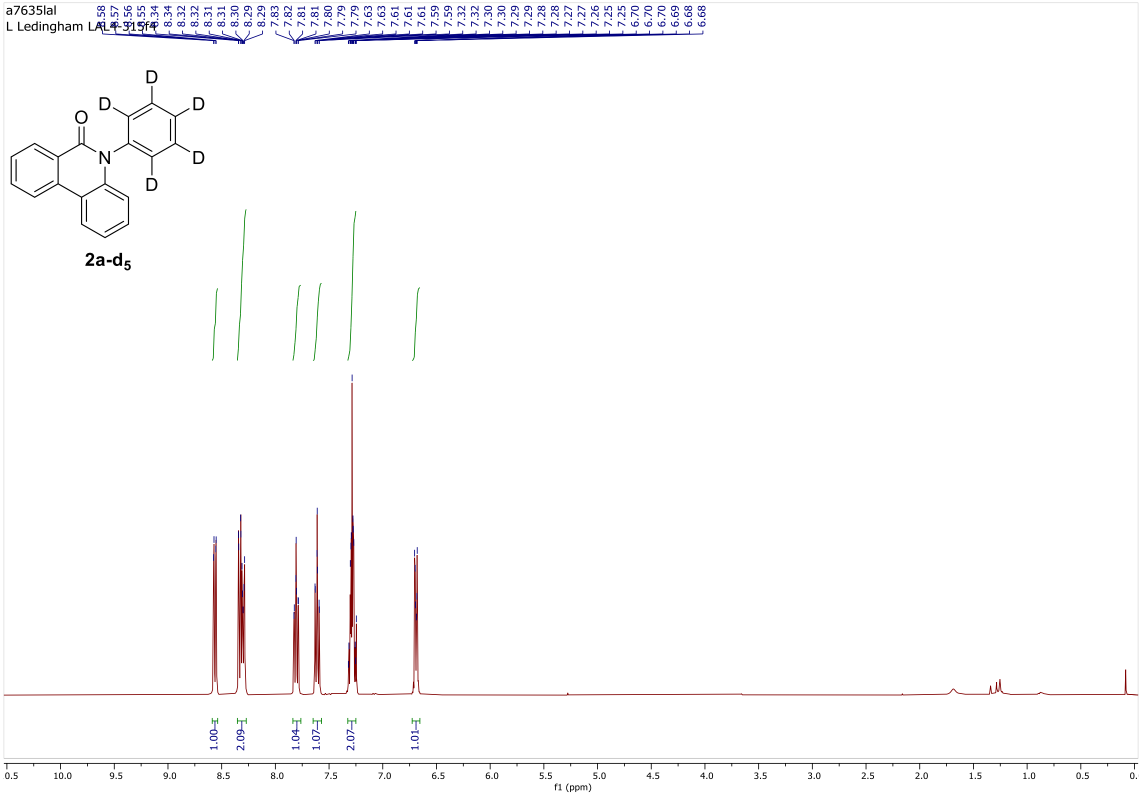

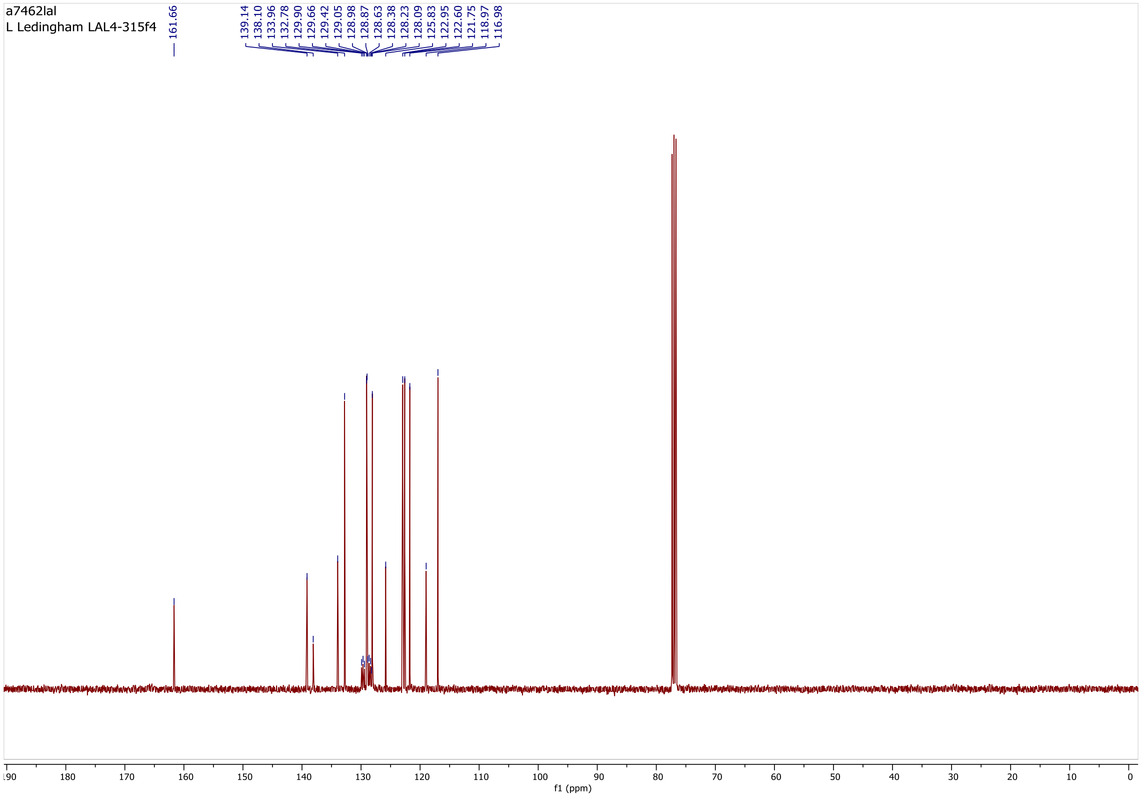

Supplementary Figure 26. Top: ^{1}H NMR spectrum of **2a-*d*$_5$** (400 MHz, 298 K, $CDCl_3$). Bottom: ^{13}C NMR spectrum of **2a-*d*$_5$** (101 MHz, 298 K, $CDCl_3$).

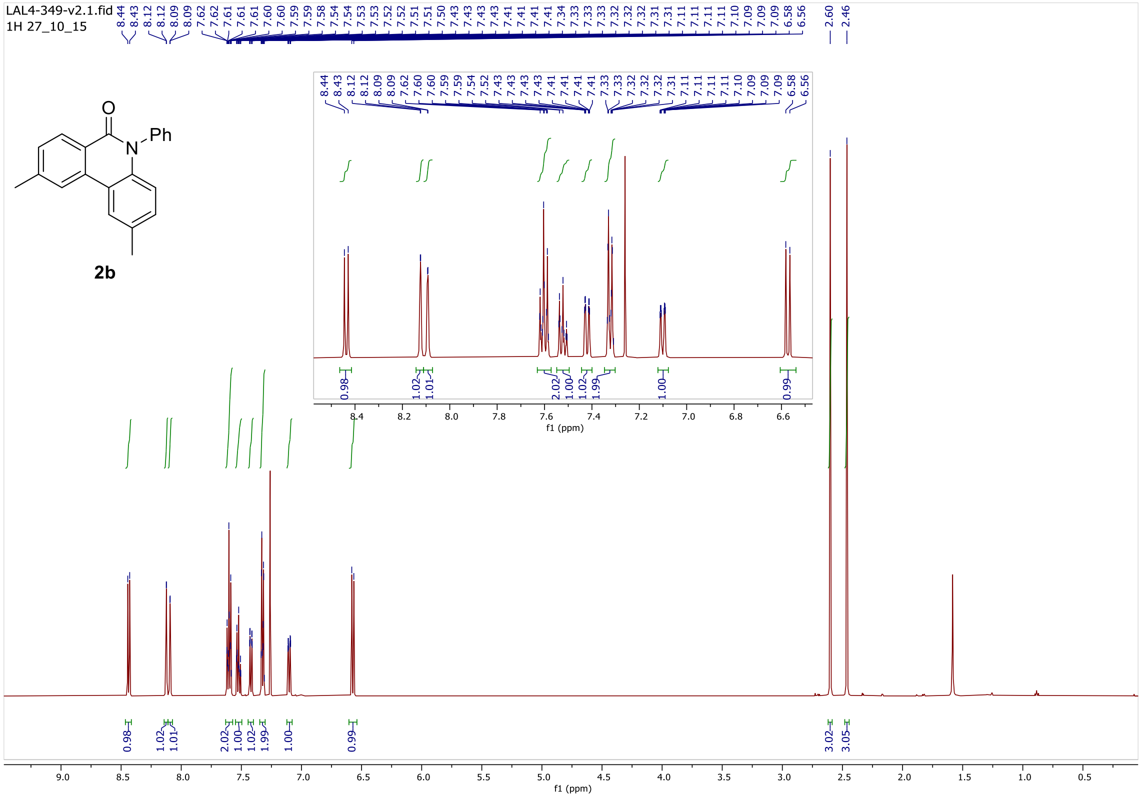

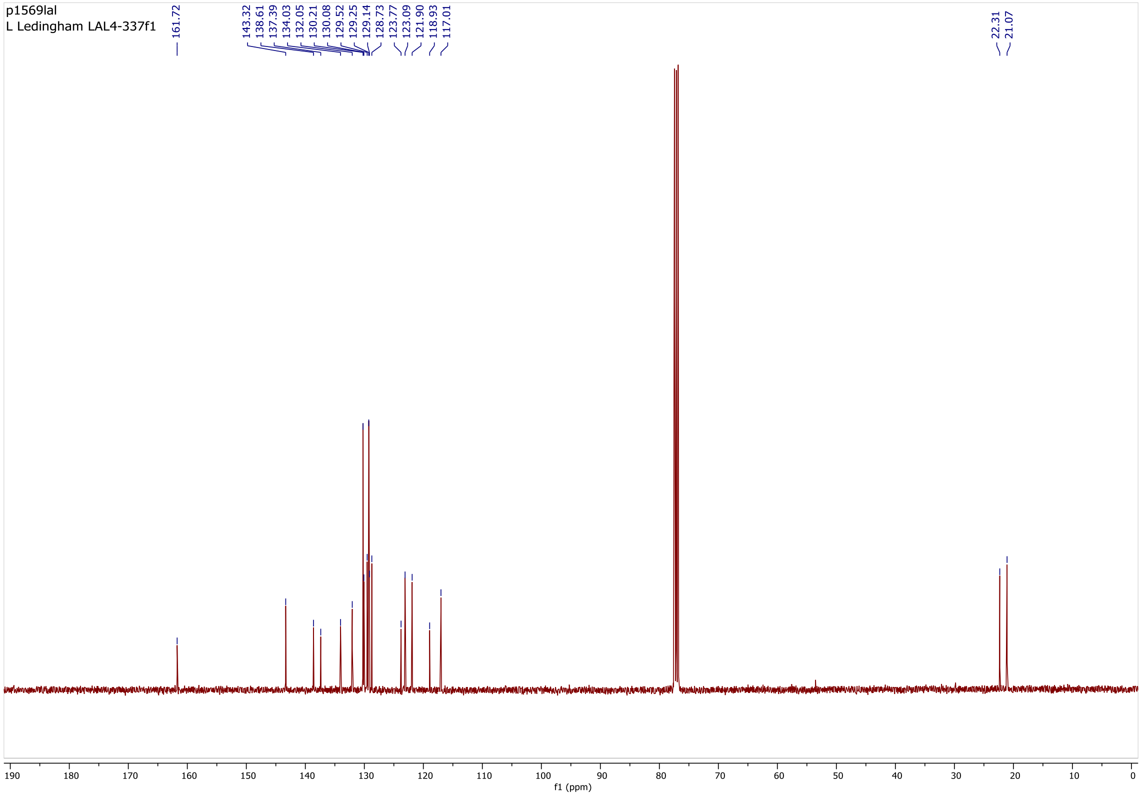

Supplementary Figure 27. Top: ^{1}H NMR spectrum of **2b** (500 MHz, 298 K, $CDCl_3$). Bottom: ^{13}C NMR spectrum of **2b** (101 MHz, 298 K, $CDCl_3$).

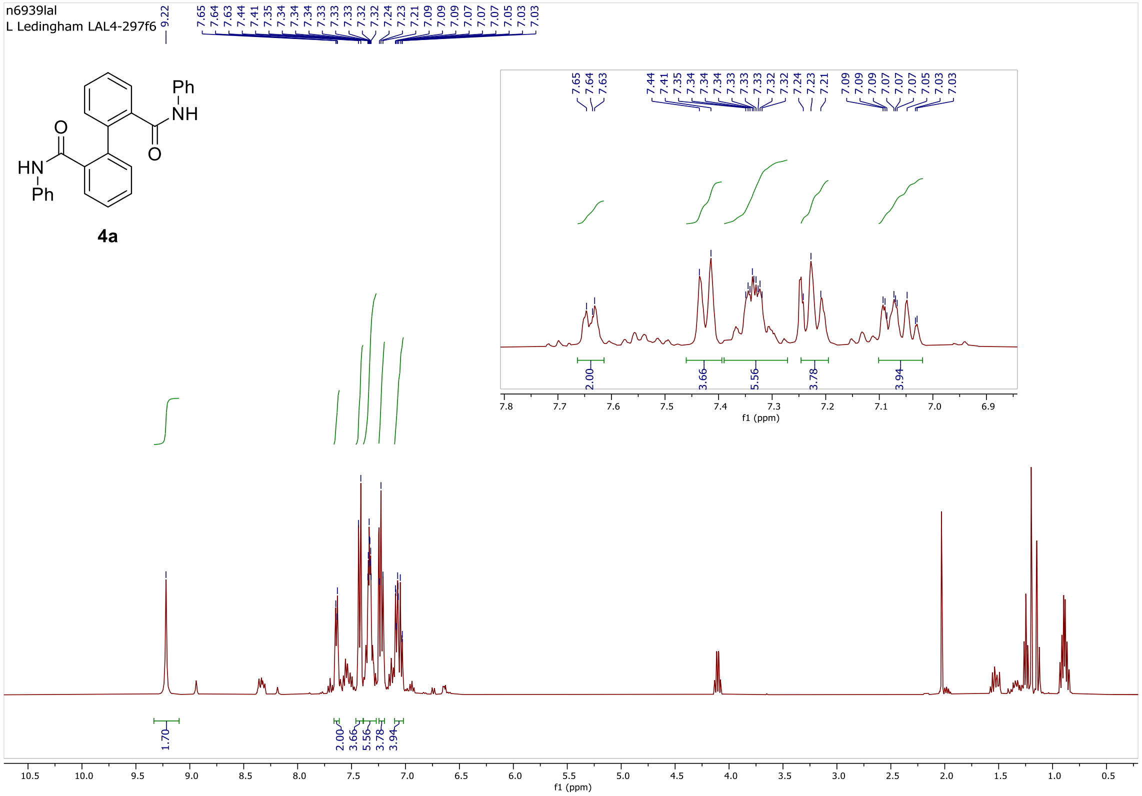

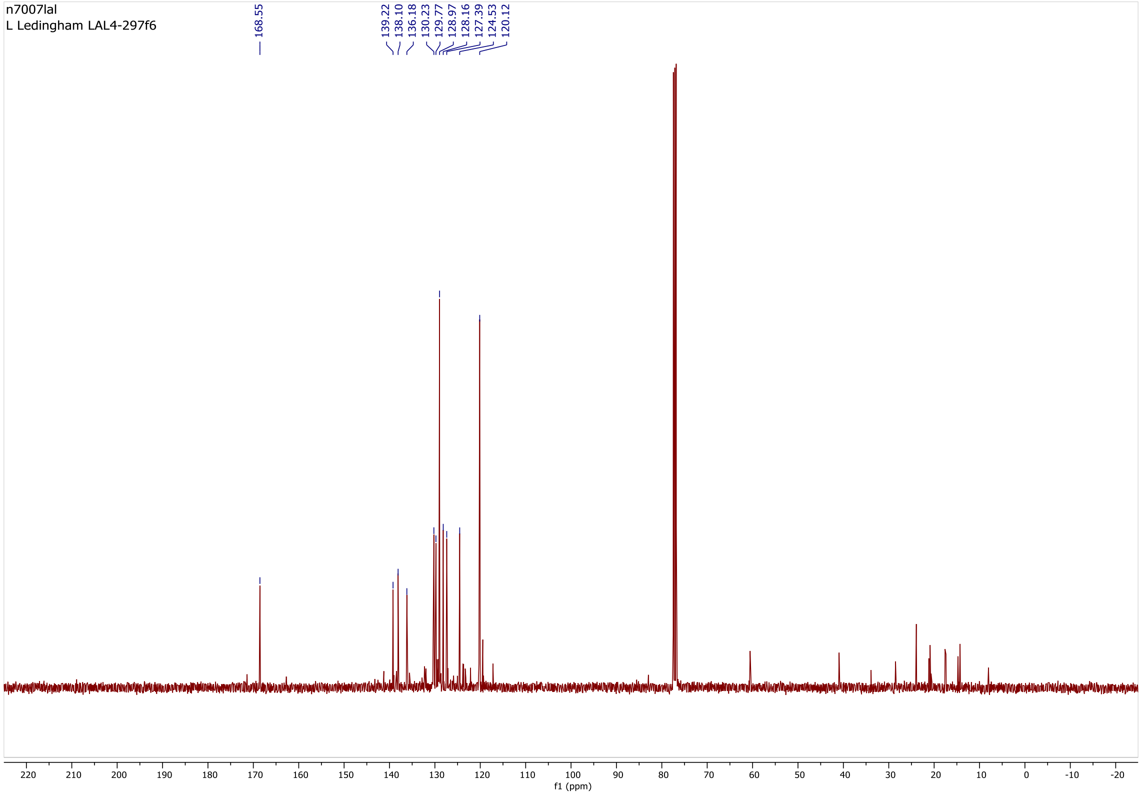

Supplementary Figure 28. Top: ^{1}H NMR spectrum of **4a** (400 MHz, 298 K, $CDCl_3$). Bottom: ^{13}C NMR spectrum of **2b** (101 MHz, 298 K, $CDCl_3$). Note that **4a** was isolated from reaction of **1a**. Both spectra show impurities, including residual ethyl acetate.

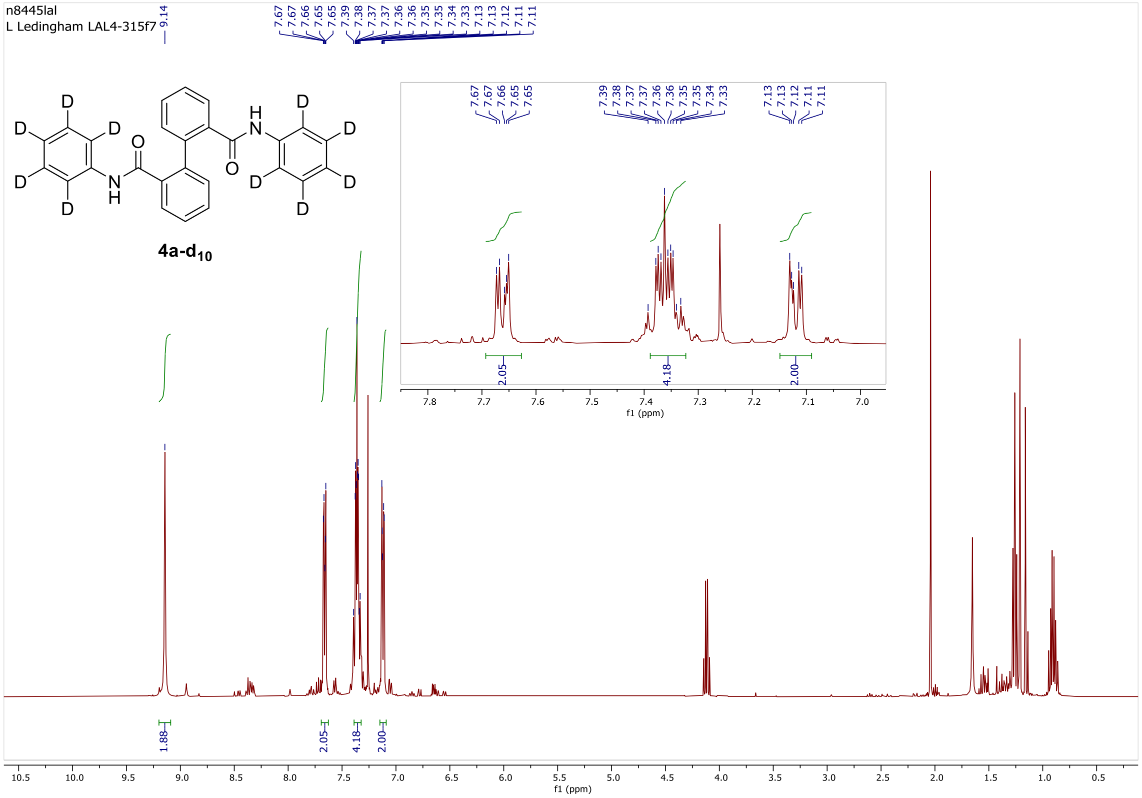

Supplementary Figure 29. ^{1}H NMR spectrum of **4a-*d*$_{10}$** (400 MHz, 298 K, $CDCl_3$). Note that **4a-*d*$_{10}$** was isolated from reaction of **1a-*d*$_5$**. The spectrum shows impurities, including residual ethyl acetate.

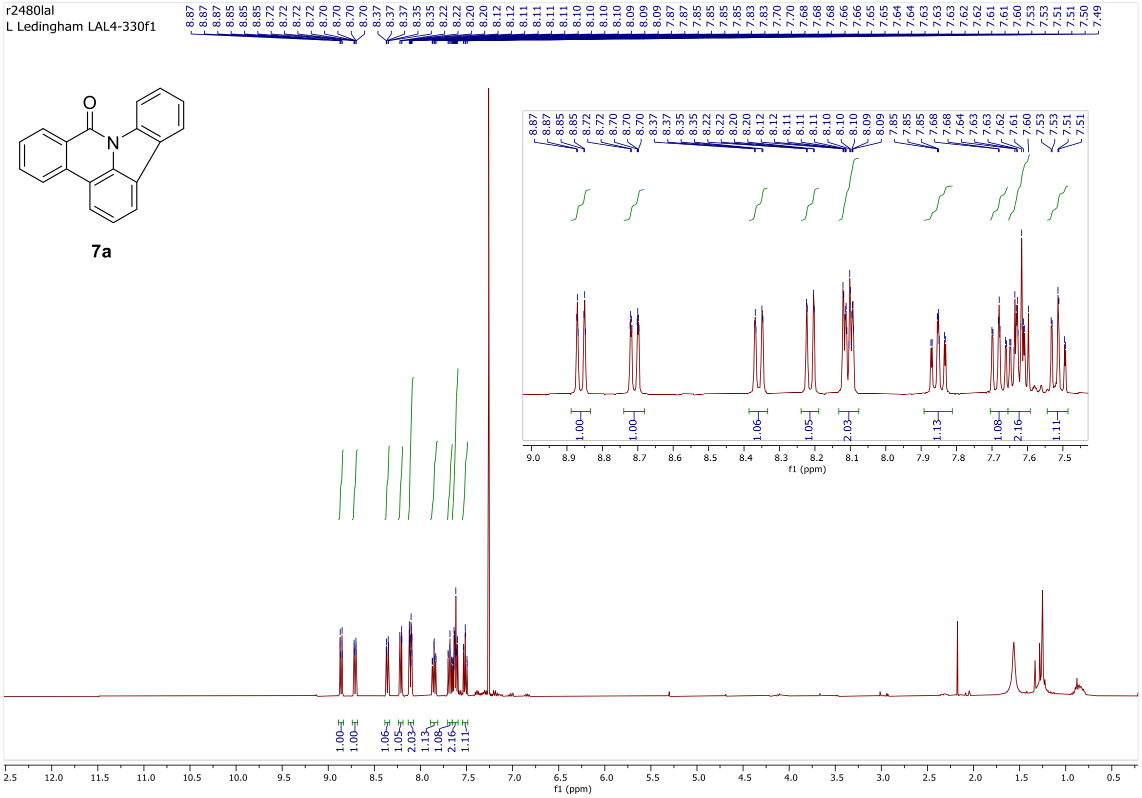

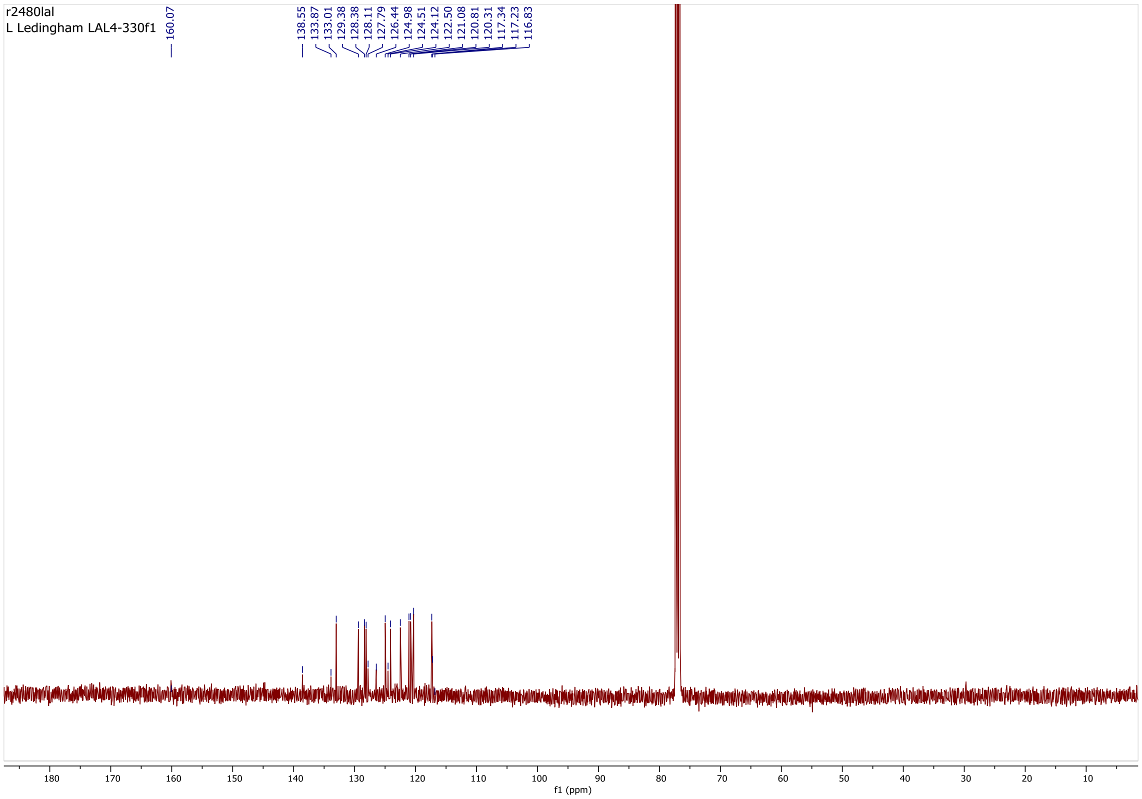

Supplementary Figure 30. Top: ^{1}H NMR spectrum of **7a** (500 MHz, 298 K, $CDCl_3$). Bottom: ^{13}C NMR spectrum of **7a** (101 MHz, 298 K, $CDCl_3$). Note that compound **7a** was isolated from a reaction of **1a**.

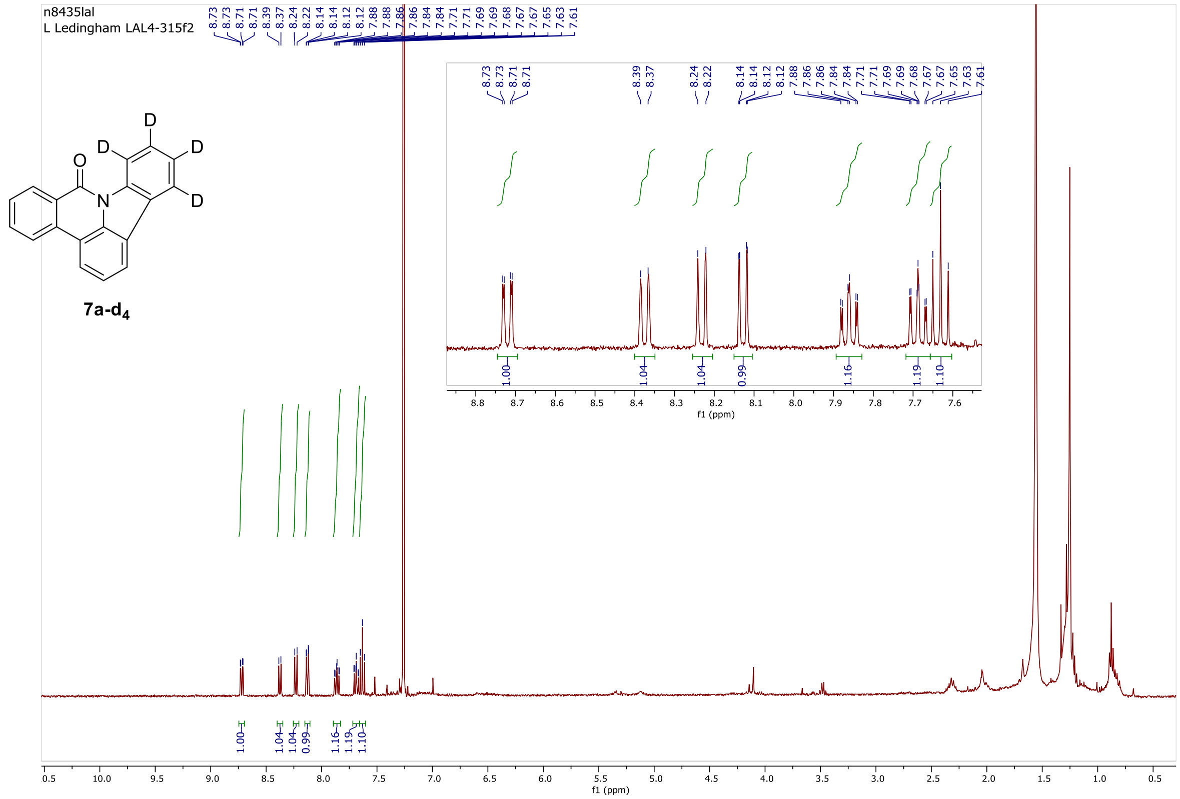

Supplementary Figure 31. Top: ^{1}H NMR spectrum of **7a-*d*$_4$** (400 MHz, 298 K, $CDCl_3$). Note that **7a-*d*$_4$** was isolated from reaction of **1a-*d*$_5$**.

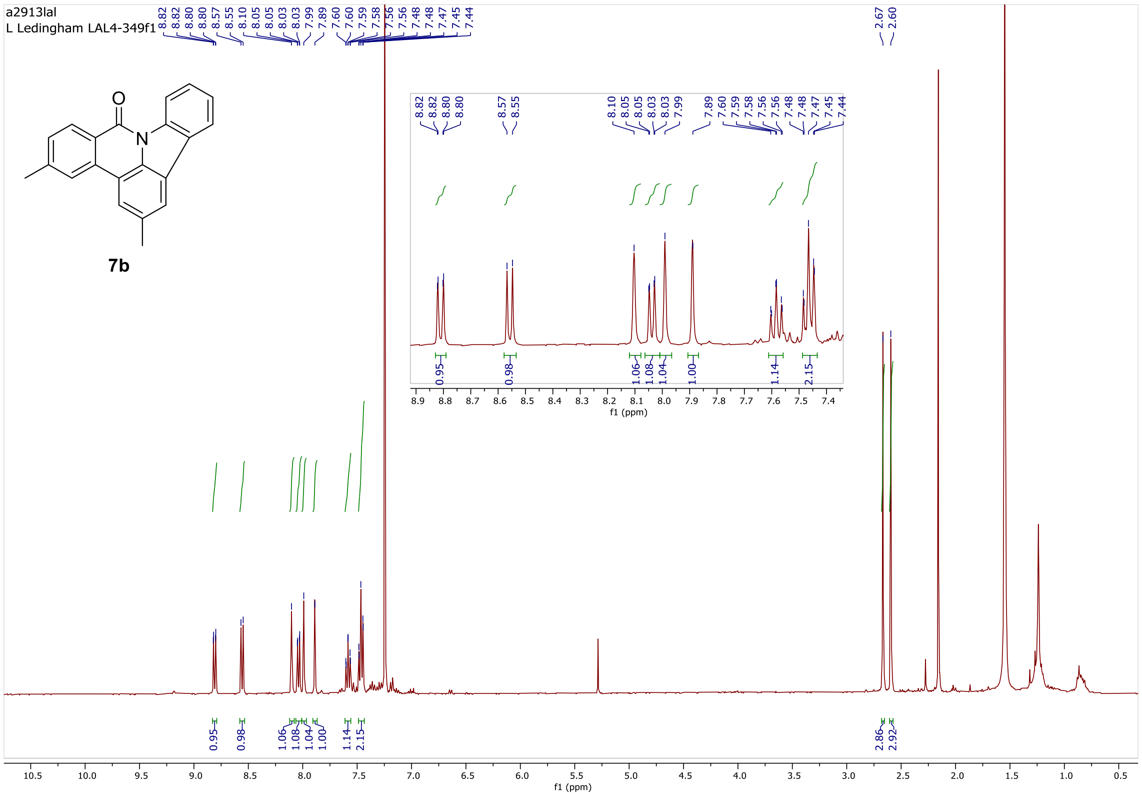

Supplementary Figure 32. ^{1}H NMR spectrum of **7b** (400 MHz, 298 K, $CDCl_3$).

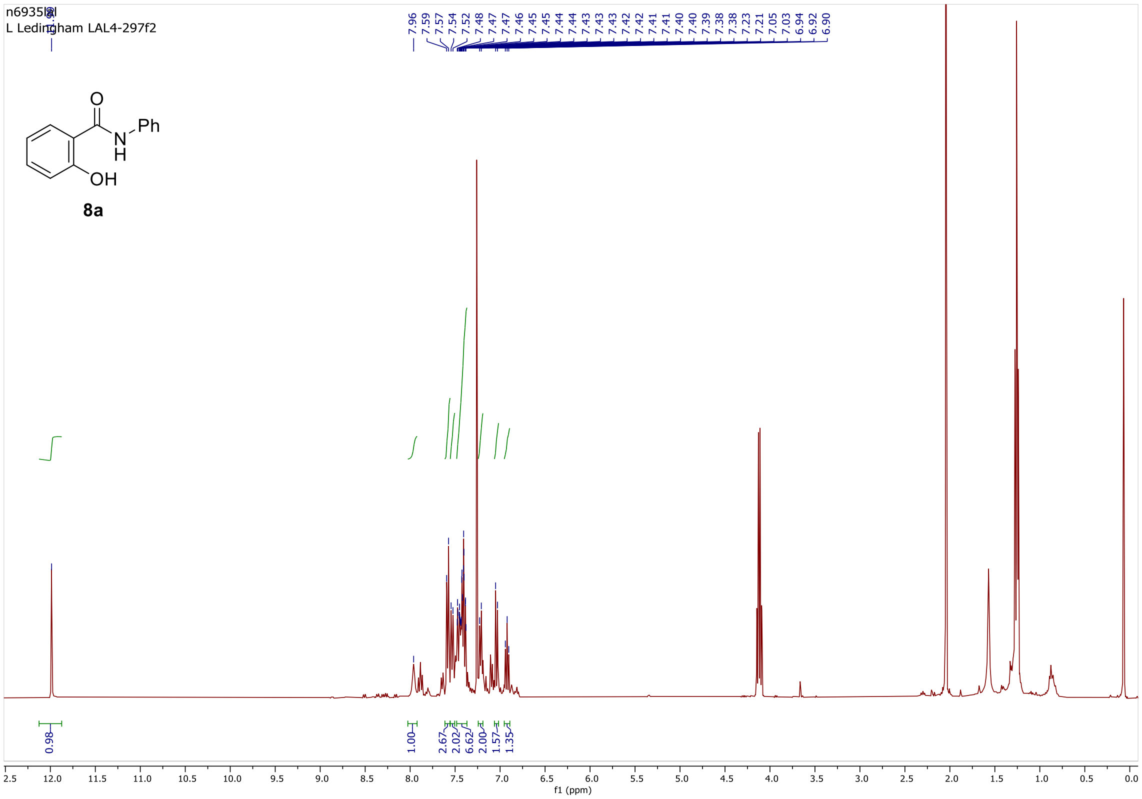

Supplementary Figure 33. ^{1}H NMR spectrum of **8a** (400 MHz, 298 K, $CDCl_3$). Note that compound **8a** was isolated from a reaction of **1a**.

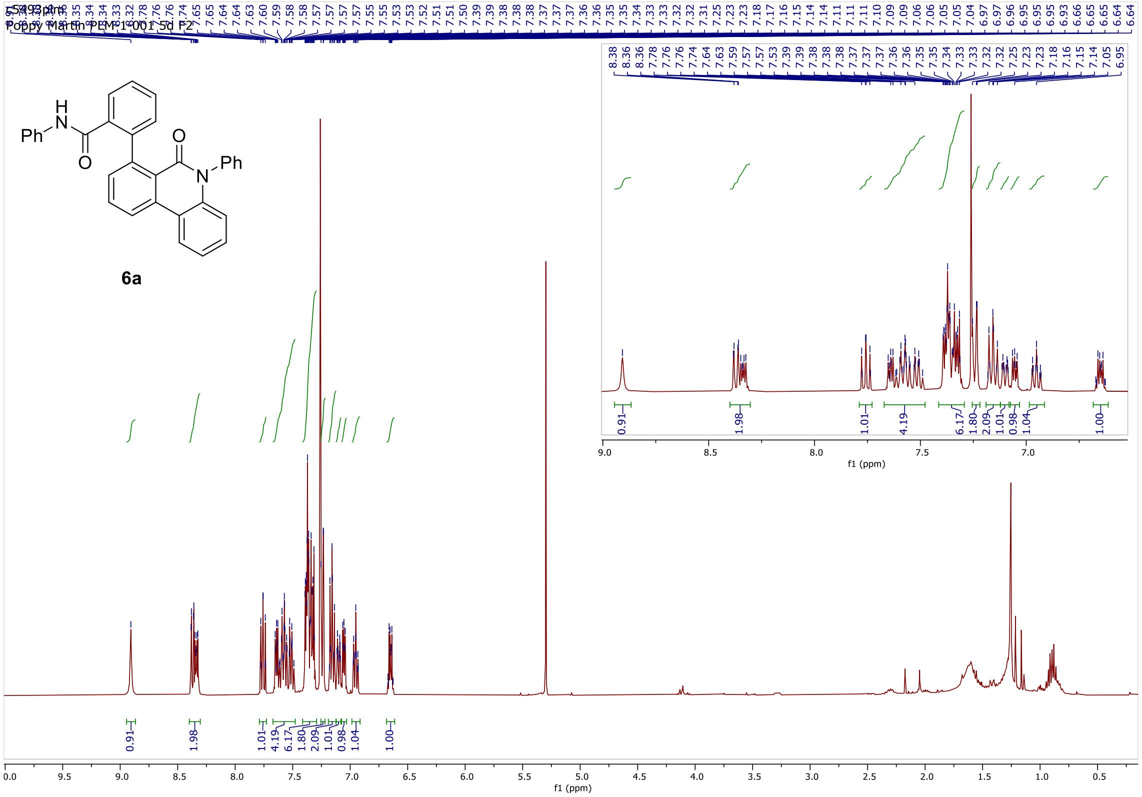

Supplementary Figure 34. ^{1}H NMR spectrum of **6a** (400 MHz, 298 K, $CDCl_3$). Note that compound **6a** was isolated from a reaction of **1a**.

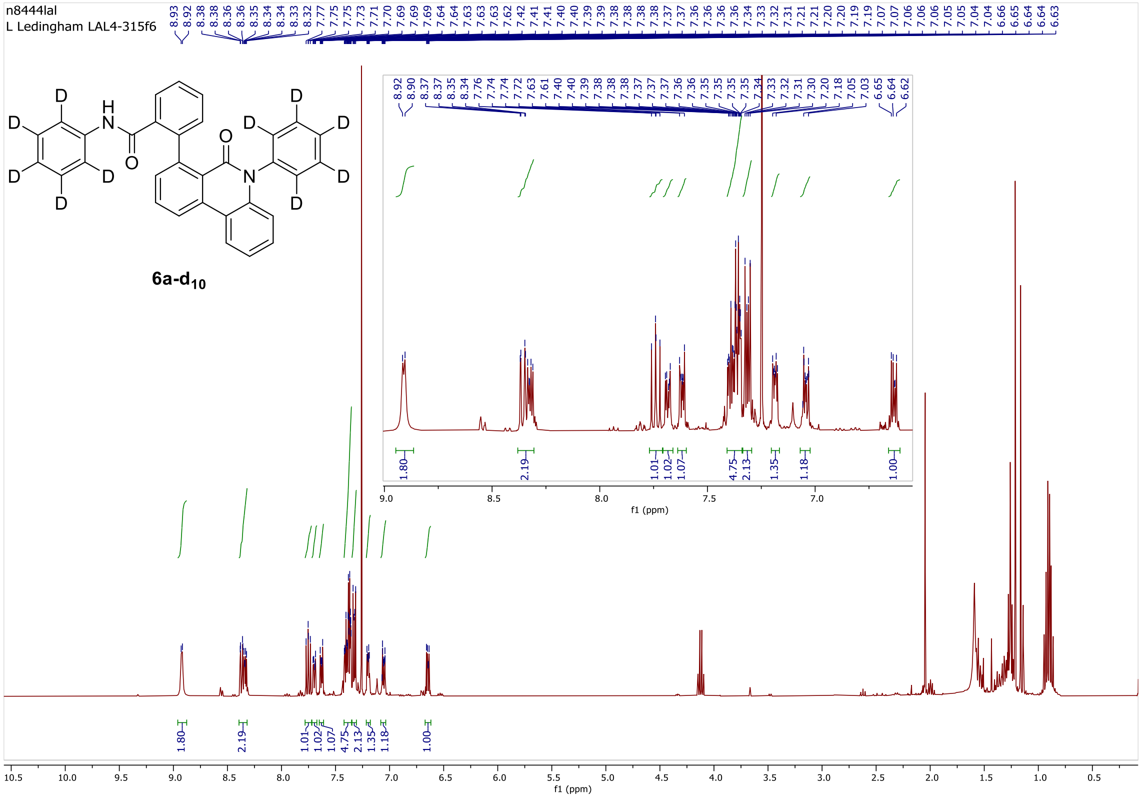

Supplementary Figure 35. ^{1}H NMR spectrum of **6a-*d*$_{10}$** (400 MHz, 298 K, $CDCl_3$). Note that compound **6a-*d*$_{10}$** was isolated from a reaction of **1a-*d*$_{10}$**.

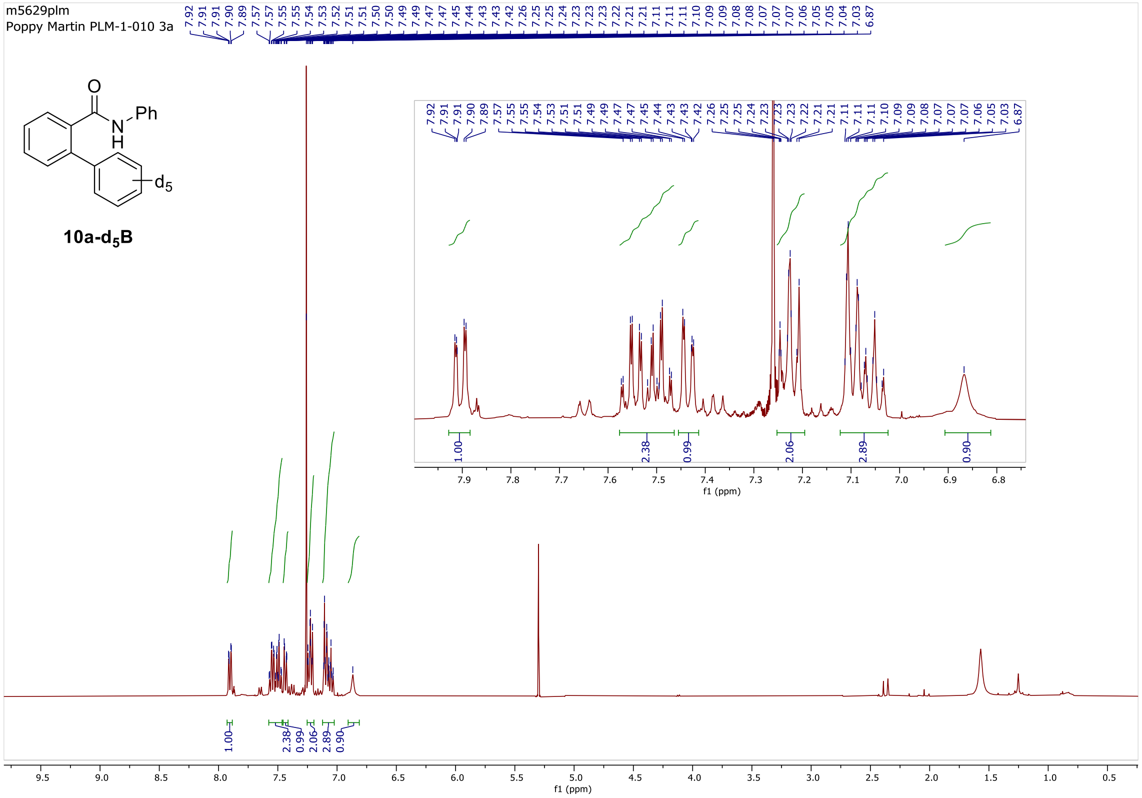

Supplementary Figure 36. ^{1}H NMR spectrum of **10a-*d*$_5$B** (400 MHz, 298 K, $CDCl_3$).

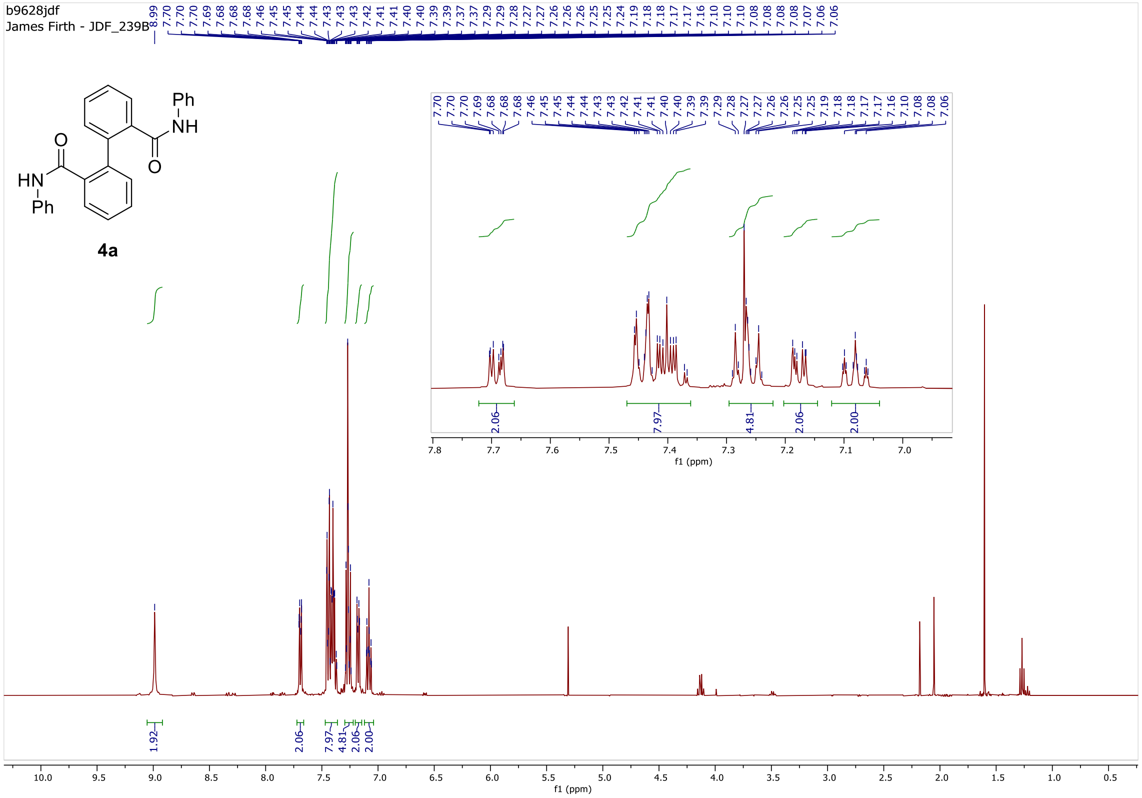

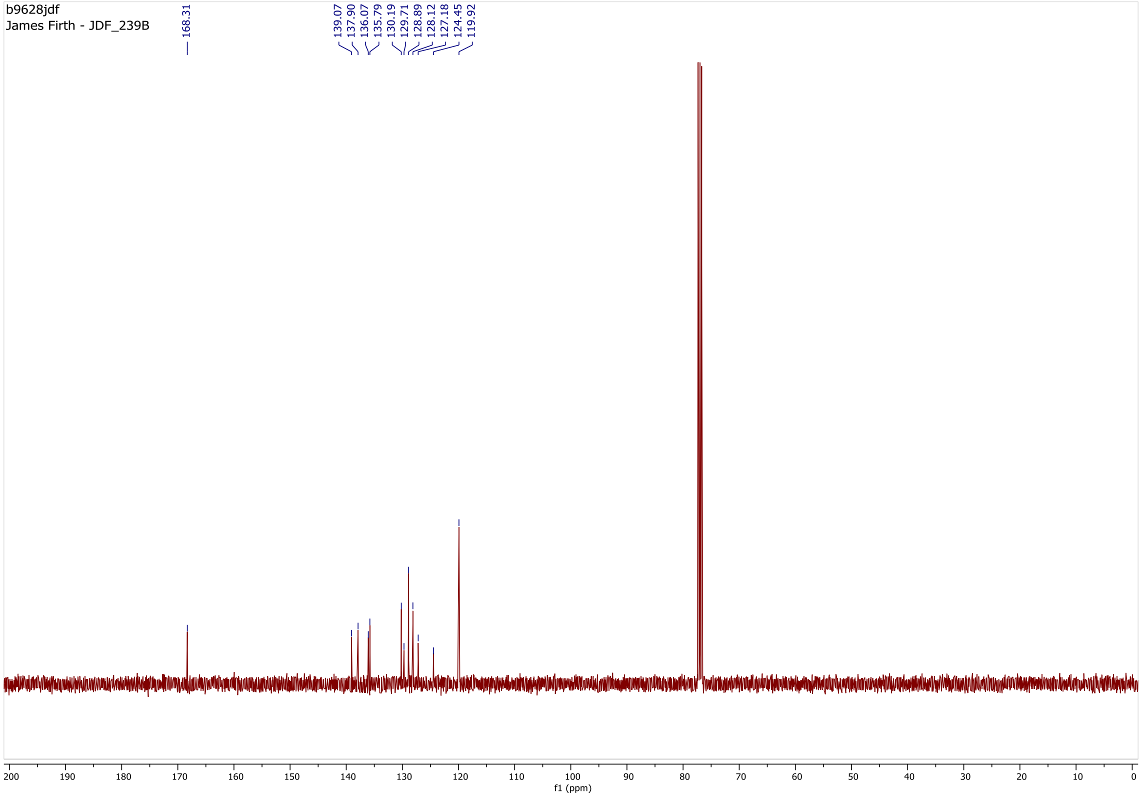

Supplementary Figure 37. Top: ^{1}H NMR spectrum of **4a** (independent sample) (400 MHz, 298 K, $CDCl_3$). Bottom: ^{13}C NMR spectrum of **4a** (independent sample) (101 MHz, 298 K, $CDCl_3$).

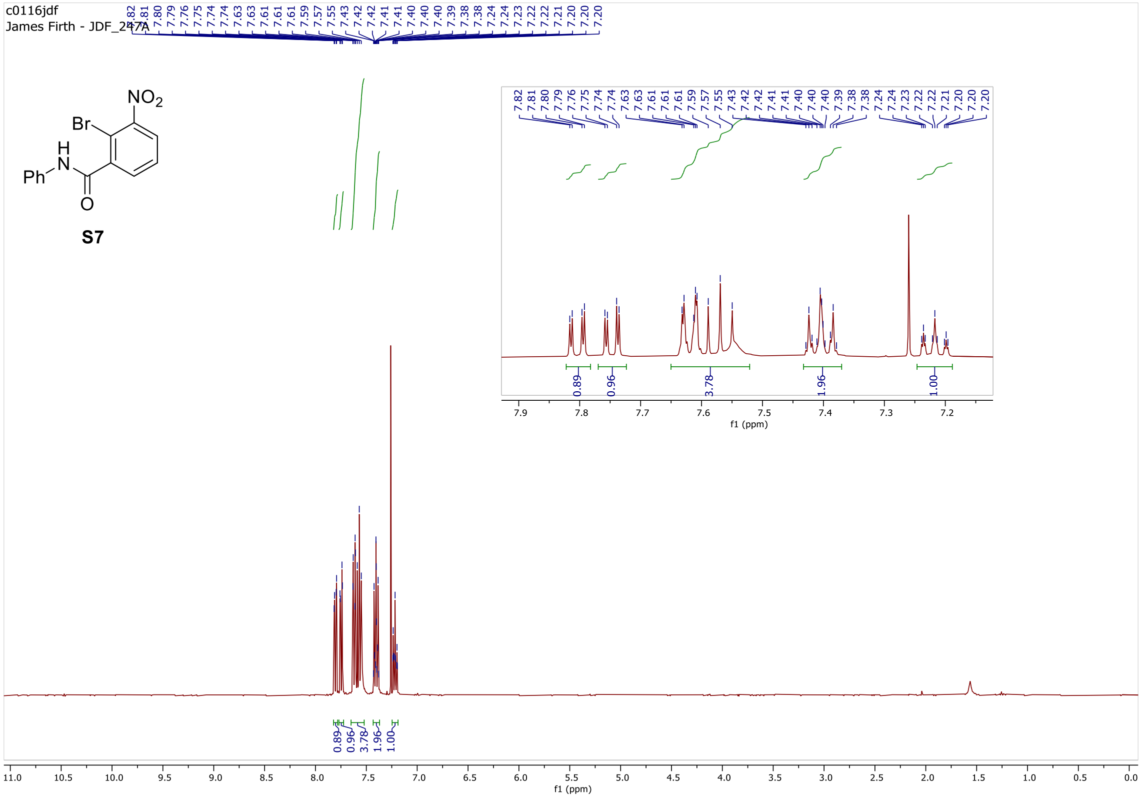

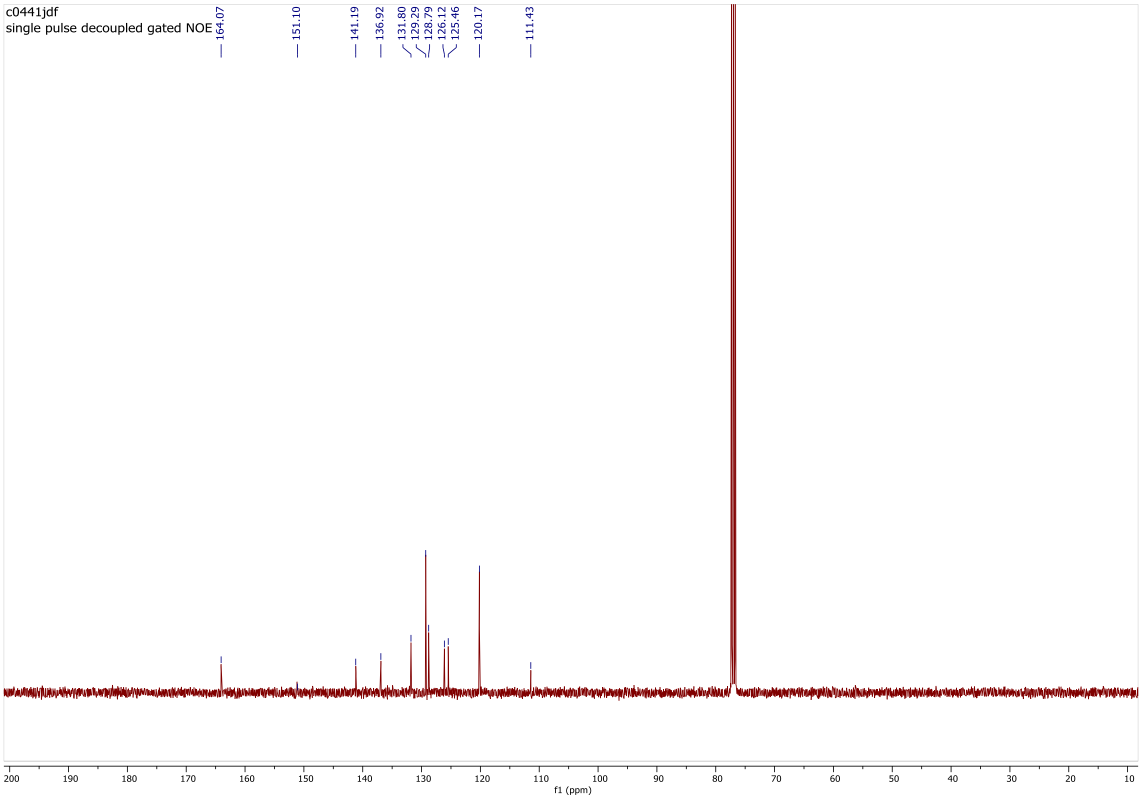

Supplementary Figure 38. Top: ^{1}H NMR spectrum of **S7** (reaction intermediate) (400 MHz, 298 K, $CDCl_3$). Bottom: ^{13}C NMR spectrum of **S7** (reaction intermediate) (101 MHz, 298 K, $CDCl_3$).

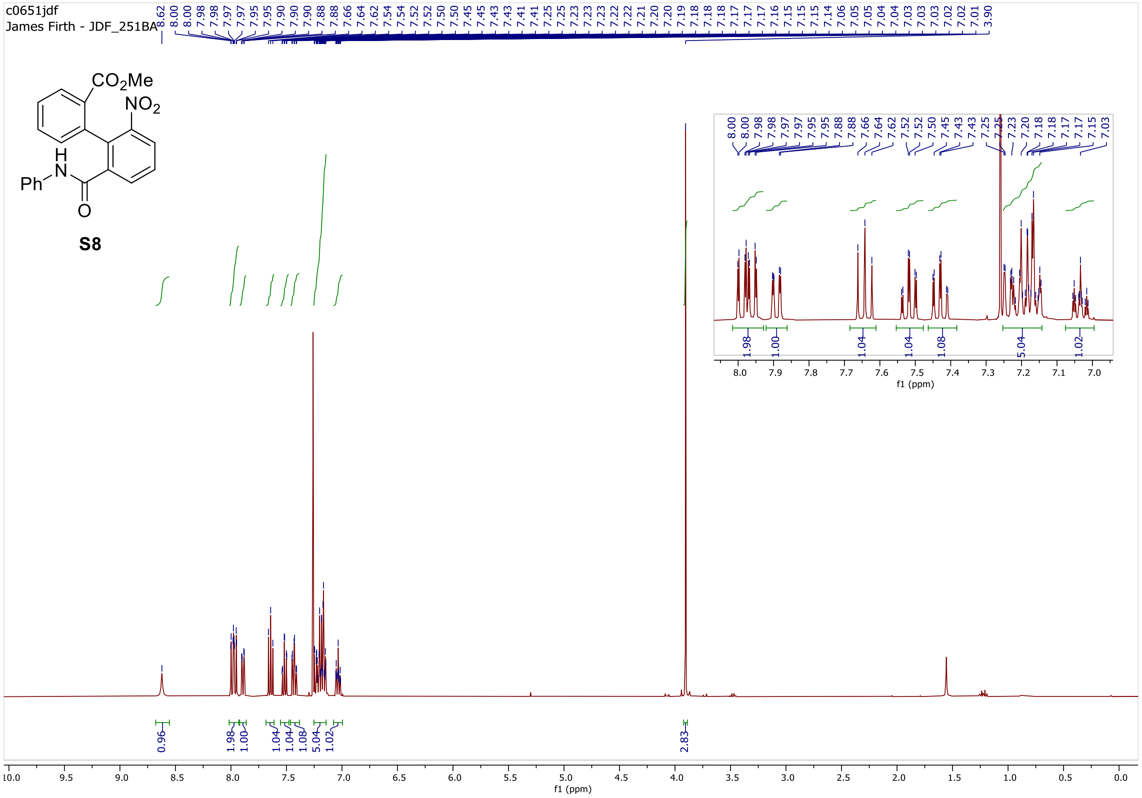

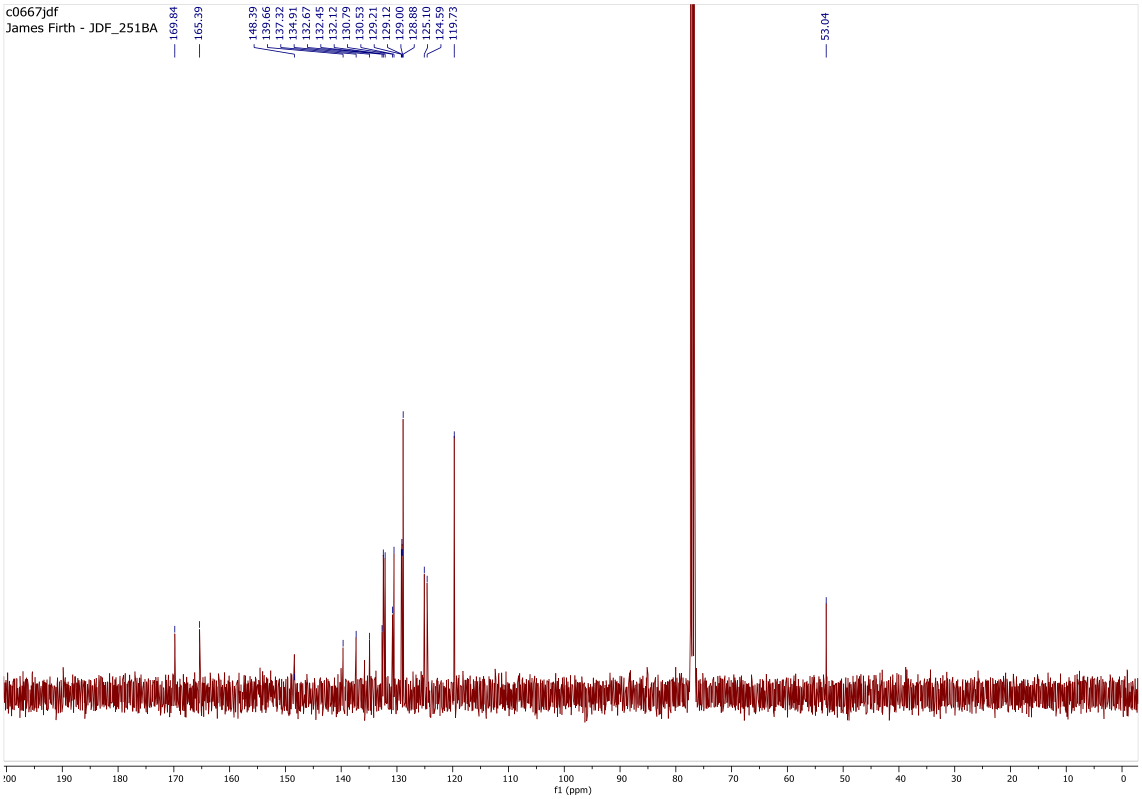

Supplementary Figure 39. Top: ^{1}H NMR spectrum of **S8** (reaction intermediate) (400 MHz, 298 K, $CDCl_3$). Bottom: ^{13}C NMR spectrum of **S8** (reaction intermediate) (101 MHz, 298 K, $CDCl_3$).

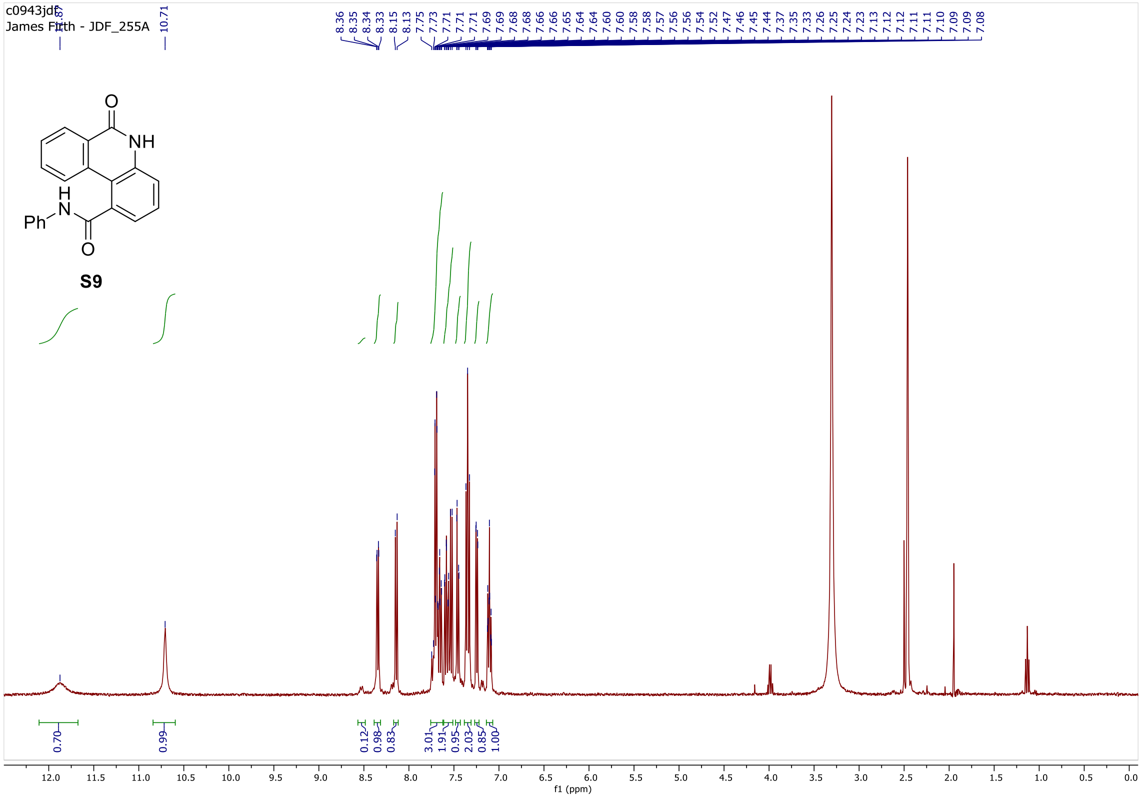

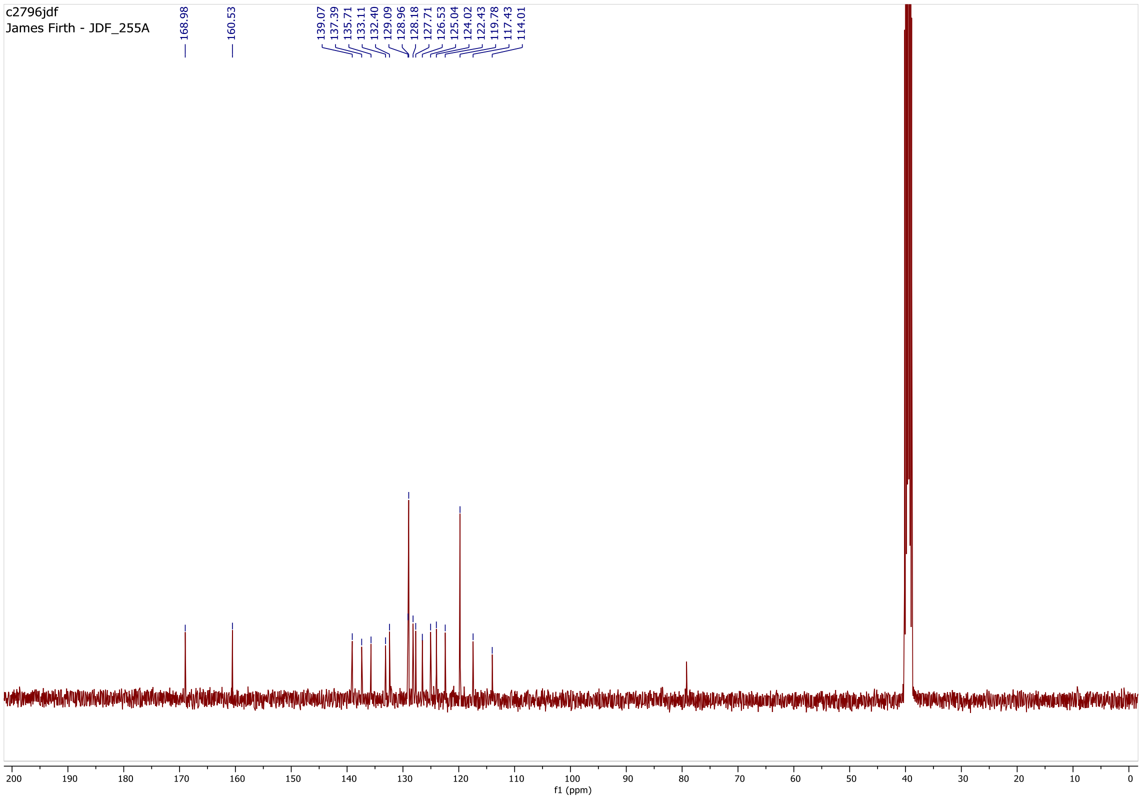

Supplementary Figure 40. Top: ^{1}H NMR spectrum of **S9** (potential product) (400 MHz, 298 K, DMSO-d_6). Bottom: ^{13}C NMR spectrum of **S9** (potential product) (101 MHz, 298 K, DMSO-d_6).

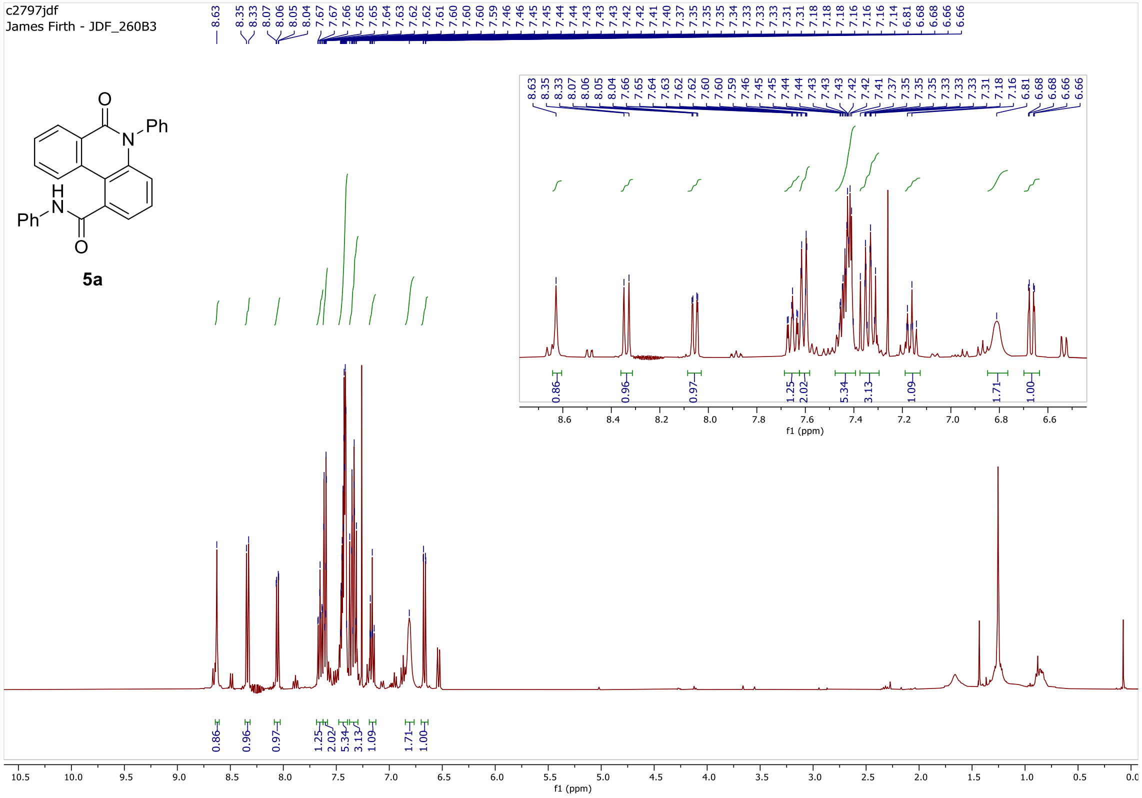

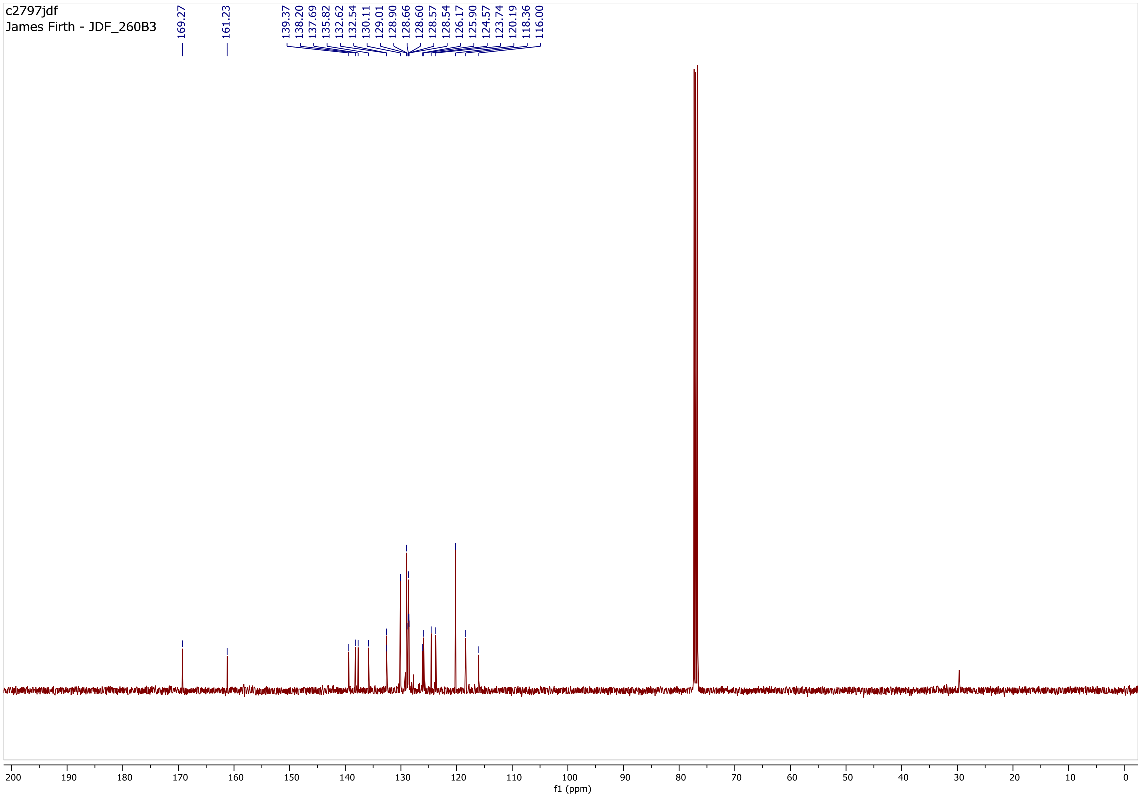

Supplementary Figure 41. Top: ^{1}H NMR spectrum of **5a** (400 MHz, 298 K, $CDCl_3$). Bottom: ^{13}C NMR spectrum of **5a** (101 MHz, 298 K, $CDCl_3$).

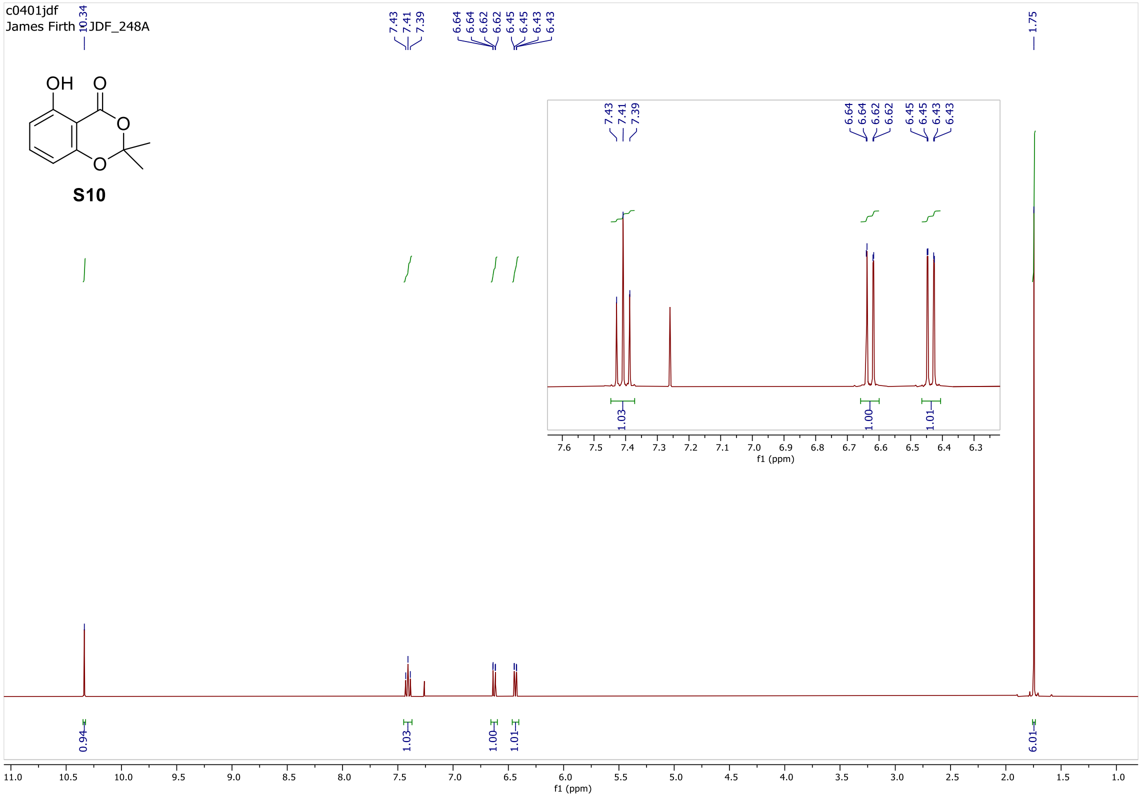

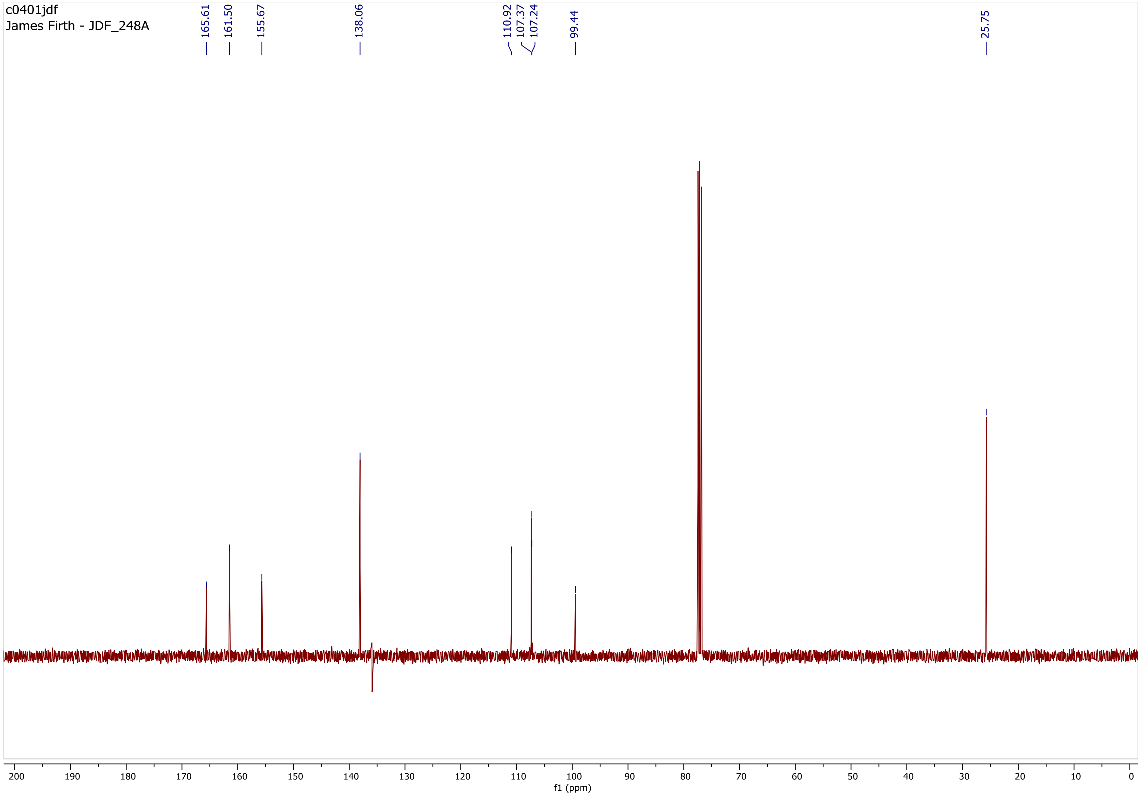

Supplementary Figure 42. Top: ^{1}H NMR spectrum of **S10** (reaction intermediate) (400 MHz, 298 K, $CDCl_3$). Bottom: ^{13}C NMR spectrum of **S10** (reaction intermediate) (101 MHz, 298 K, $CDCl_3$).

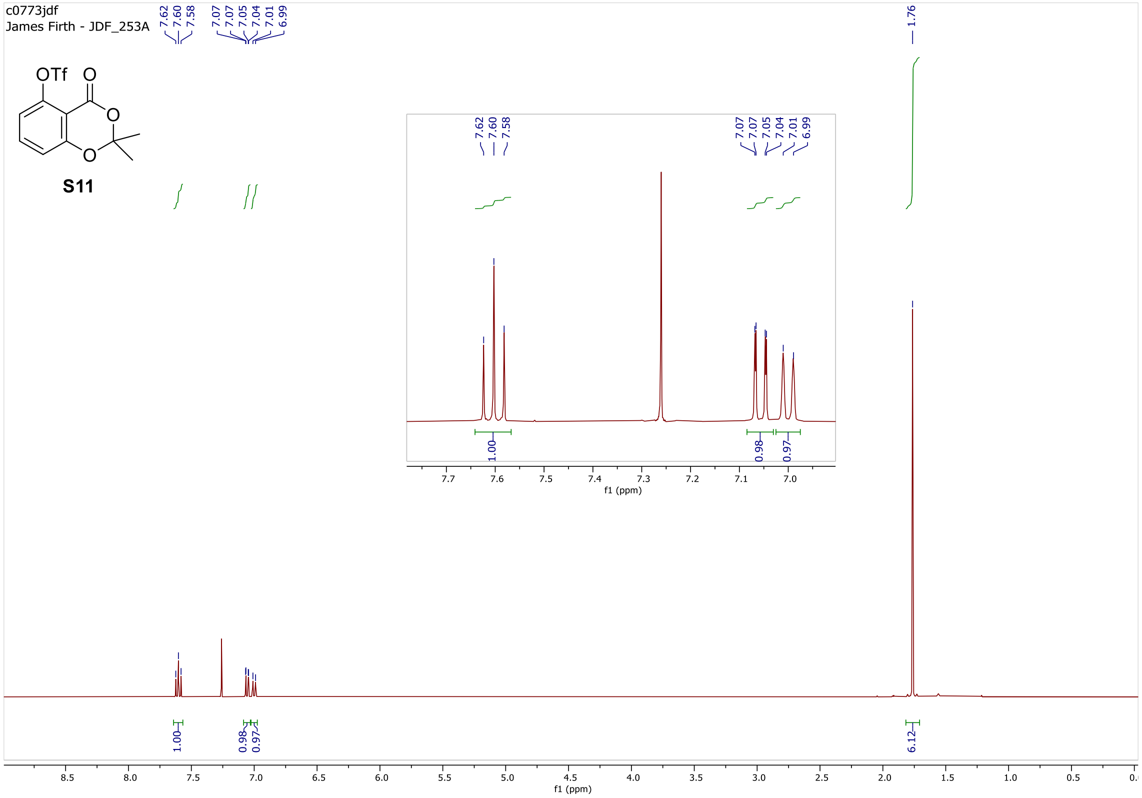

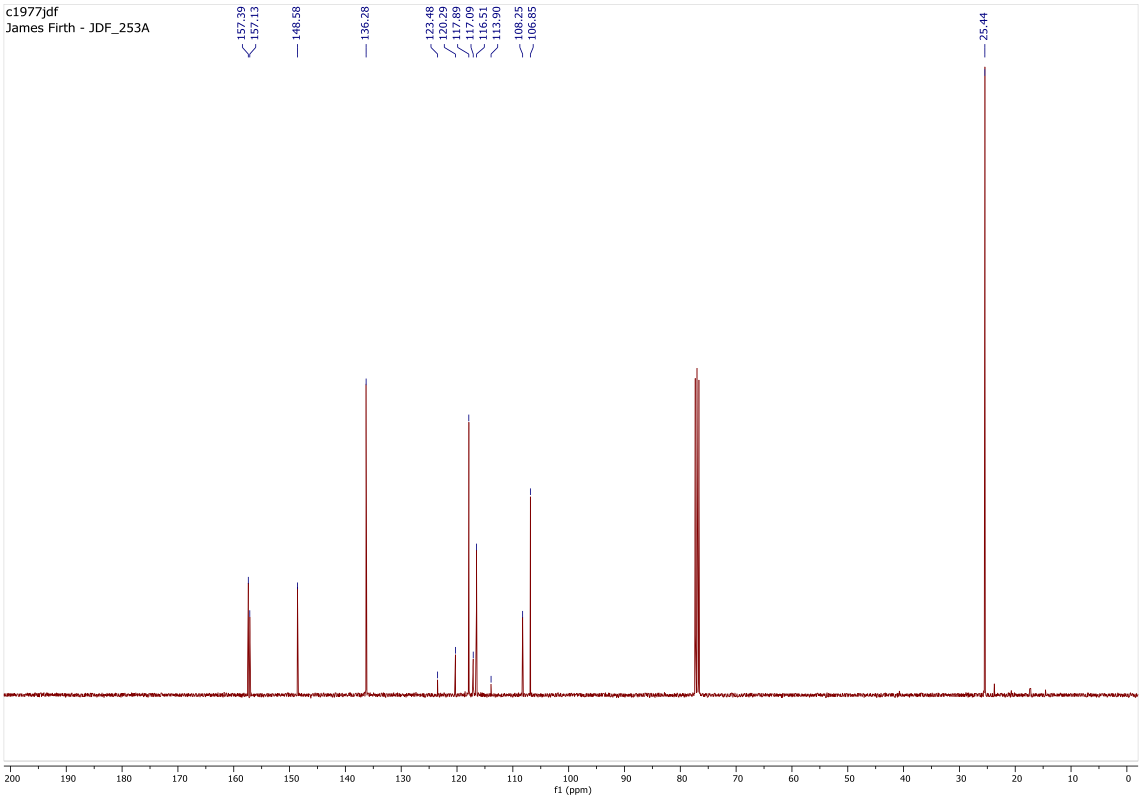

Supplementary Figure 43. Top: ^{1}H NMR spectrum of **S11** (reaction intermediate) (400 MHz, 298 K, $CDCl_3$). Bottom: ^{13}C NMR spectrum of **S11** (reaction intermediate) (101 MHz, 298 K, $CDCl_3$).

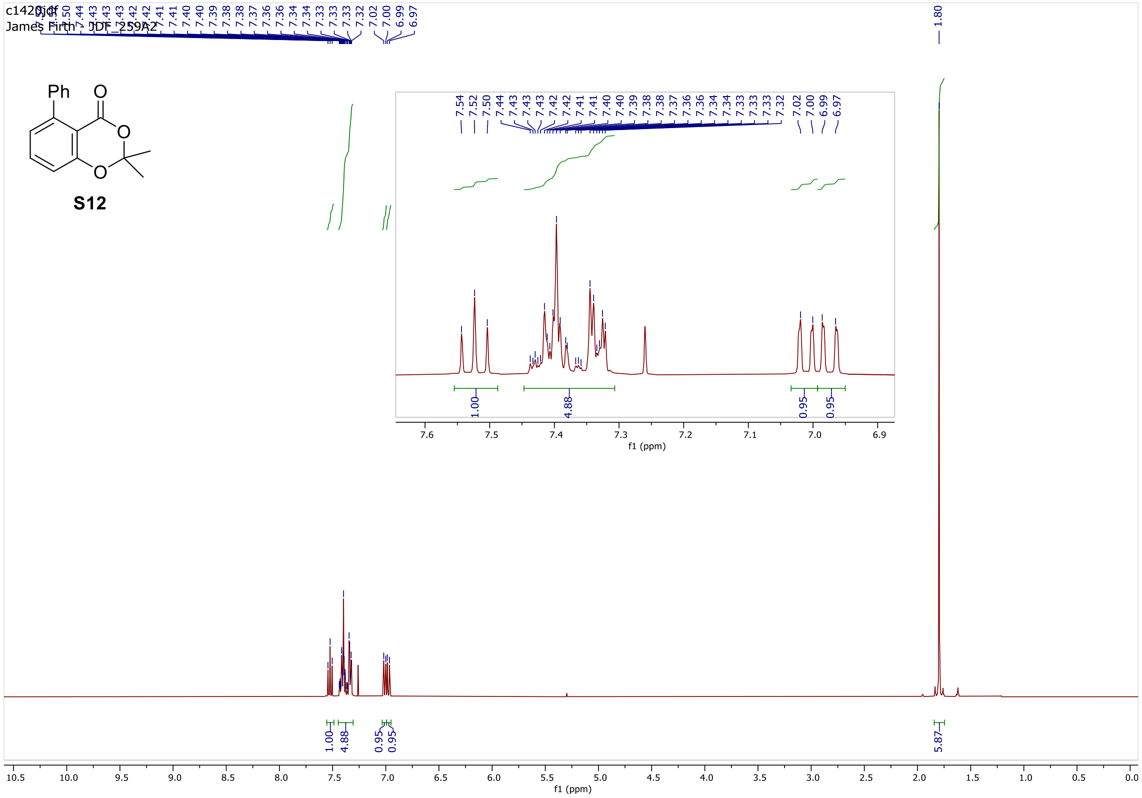

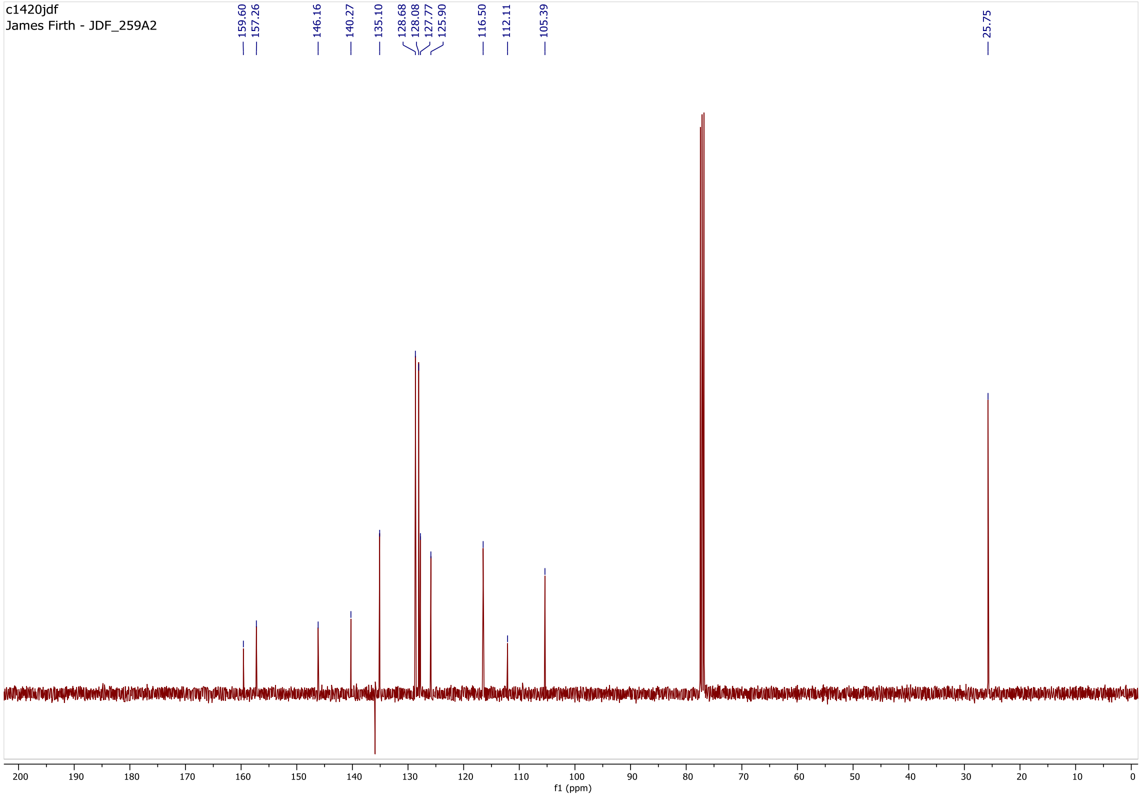

Supplementary Figure 44. Top: ^{1}H NMR spectrum of **S12** (reaction intermediate) (400 MHz, 298 K, $CDCl_3$). Bottom: ^{13}C NMR spectrum of **S12** (reaction intermediate) (101 MHz, 298 K, $CDCl_3$).

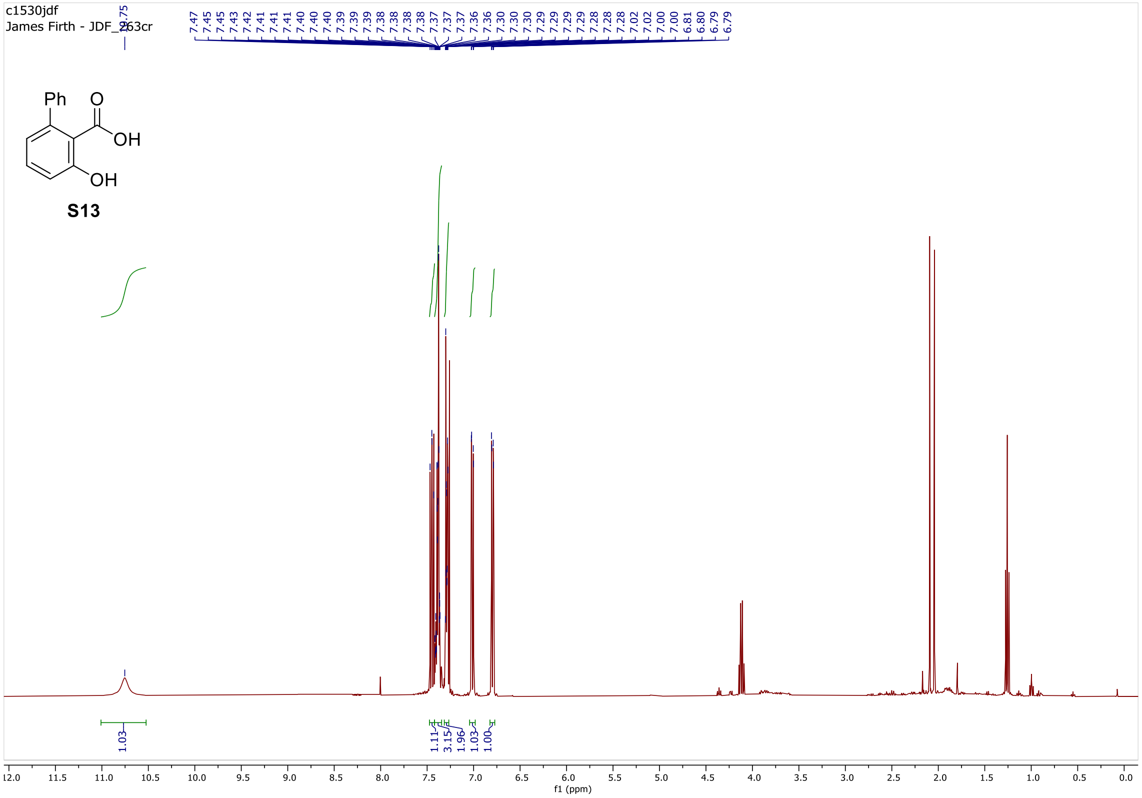

Supplementary Figure 45. ^{1}H NMR spectrum of **S13** (reaction intermediate) (400 MHz, 298 K, $CDCl_3$).

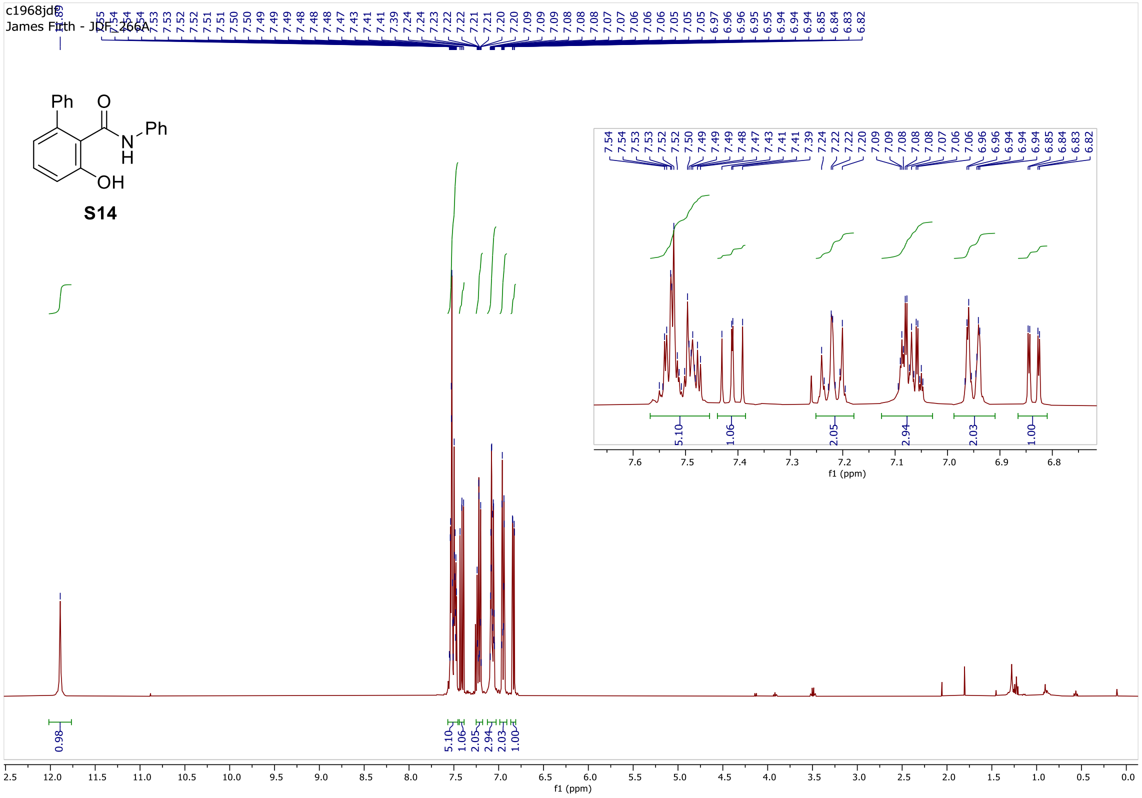

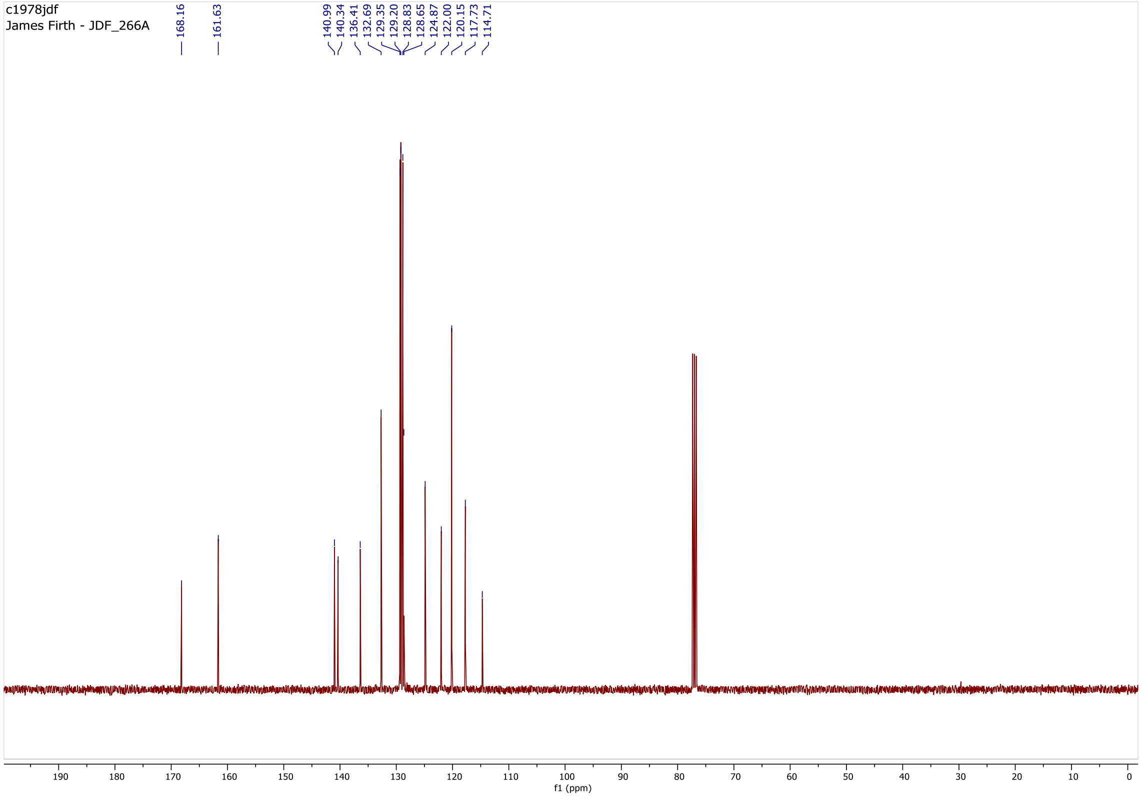

Supplementary Figure 46. Top: ^{1}H NMR spectrum of **S14** (reaction intermediate) (400 MHz, 298 K, $CDCl_3$). Bottom: ^{13}C NMR spectrum of **S14** (reaction intermediate) (101 MHz, 298 K, $CDCl_3$).

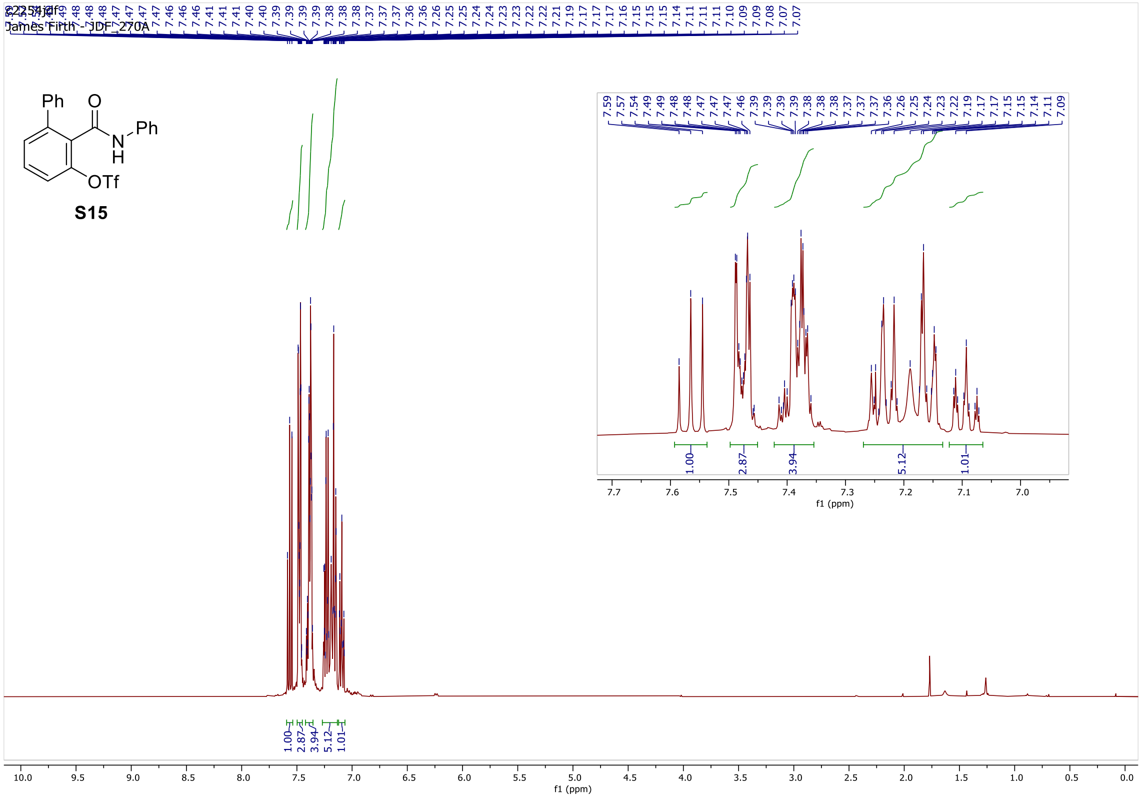

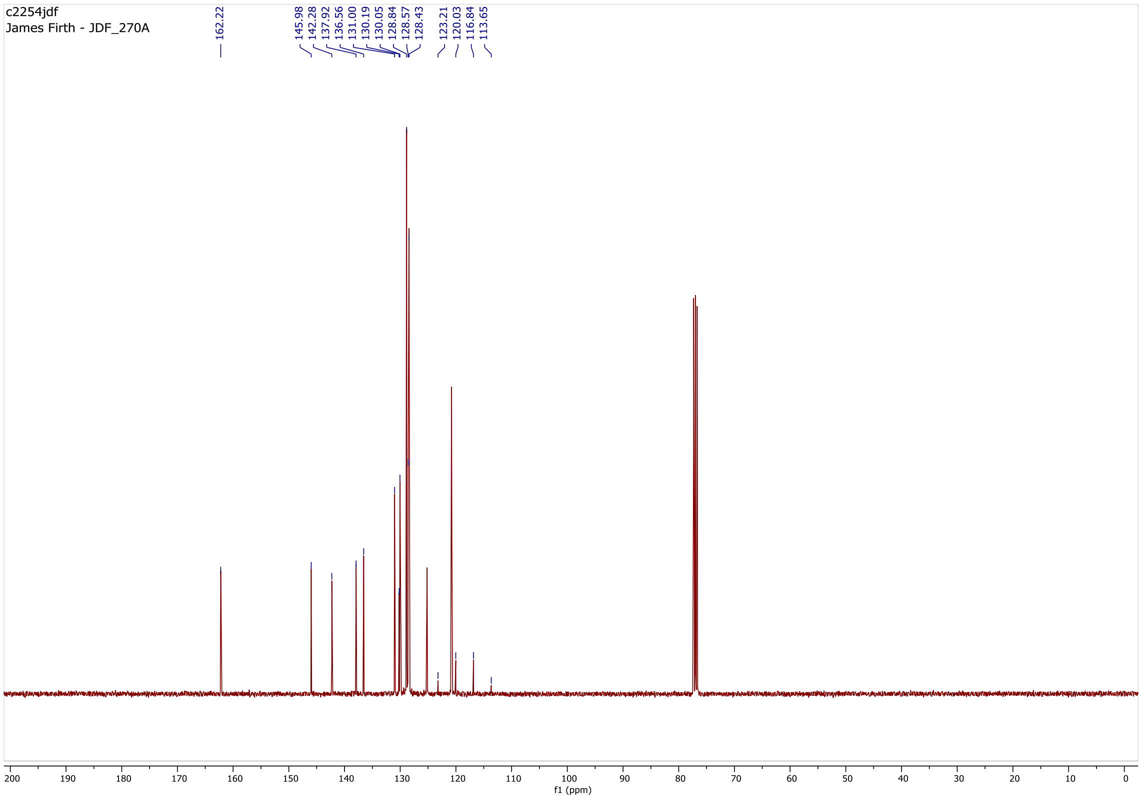

Supplementary Figure 47. Top: ^{1}H NMR spectrum of **S15** (reaction intermediate) (400 MHz, 298 K, $CDCl_3$). Bottom: ^{13}C NMR spectrum of **S15** (reaction intermediate) (101 MHz, 298 K, $CDCl_3$).

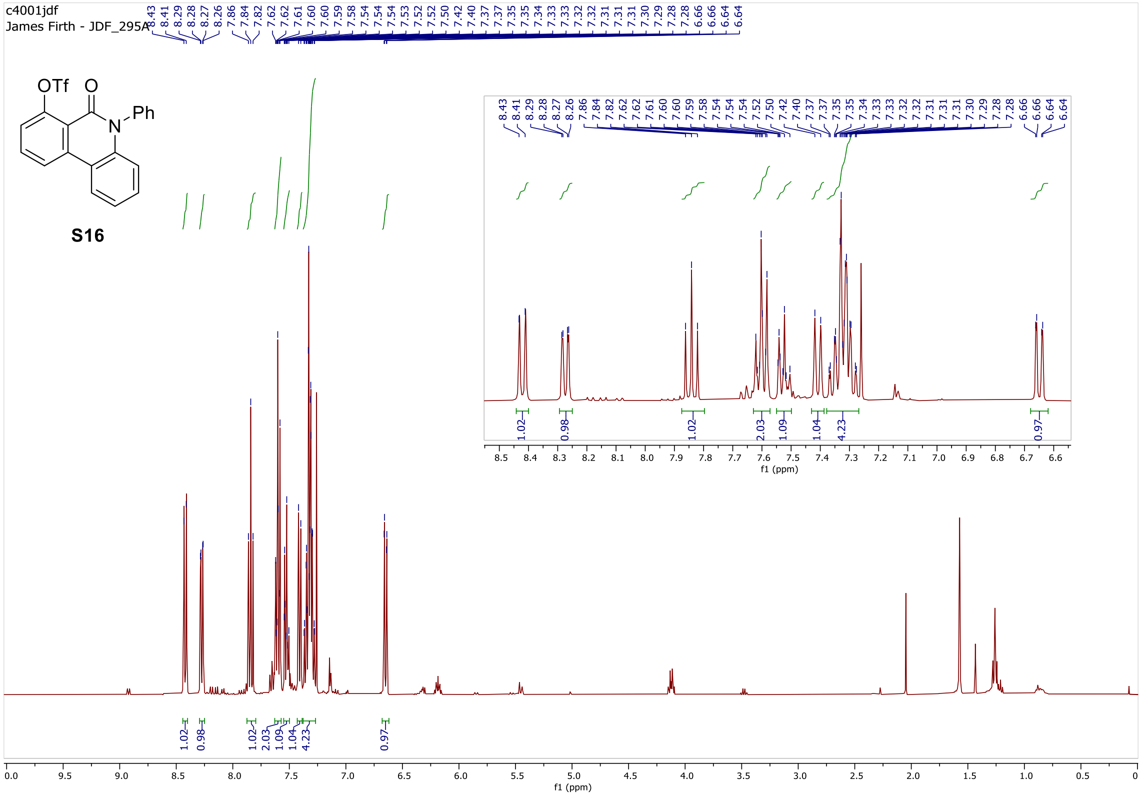

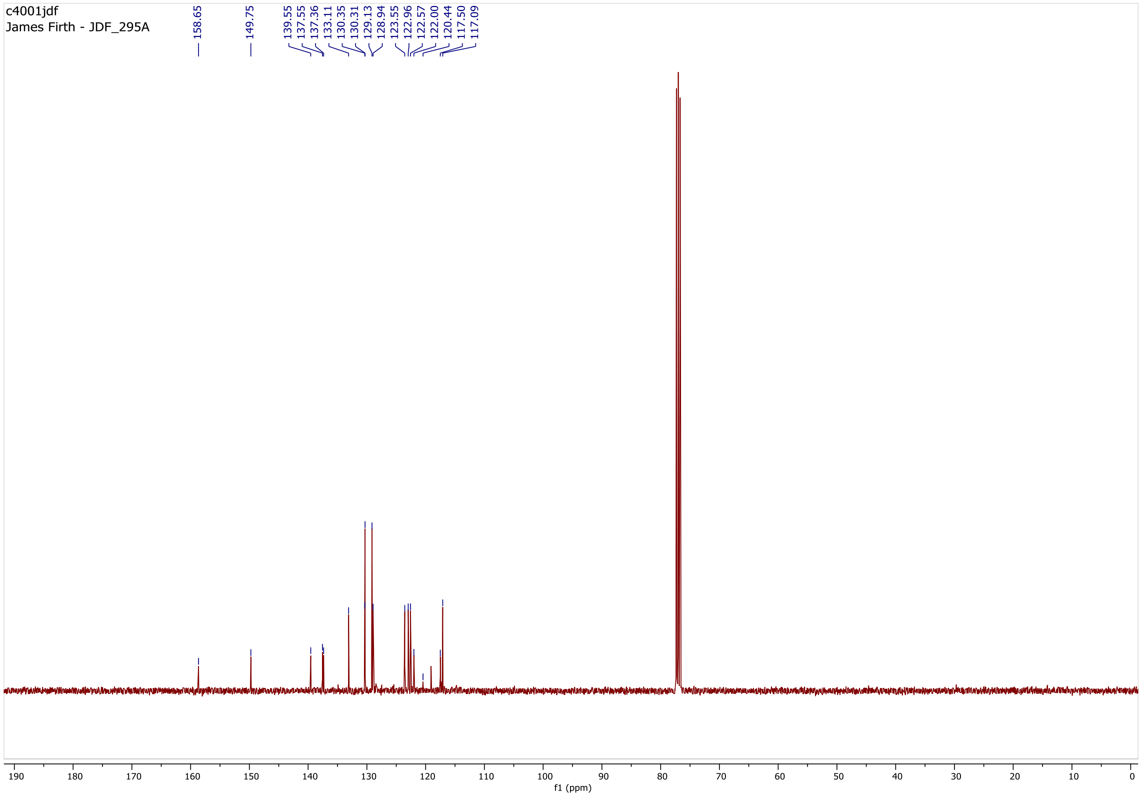

Supplementary Figure 48. Top: ^{1}H NMR spectrum of **S16** (reaction intermediate) (400 MHz, 298 K, $CDCl_3$). Bottom: ^{13}C NMR spectrum of **S16** (reaction intermediate) (101 MHz, 298 K, $CDCl_3$).

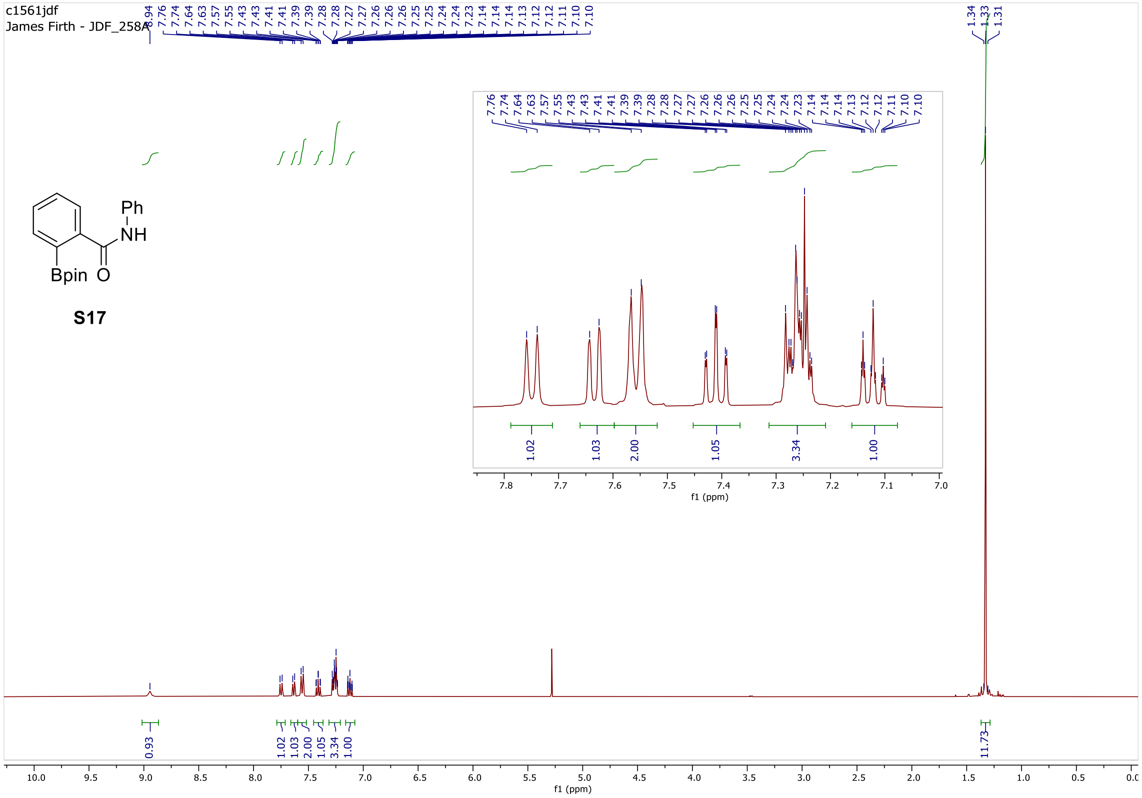

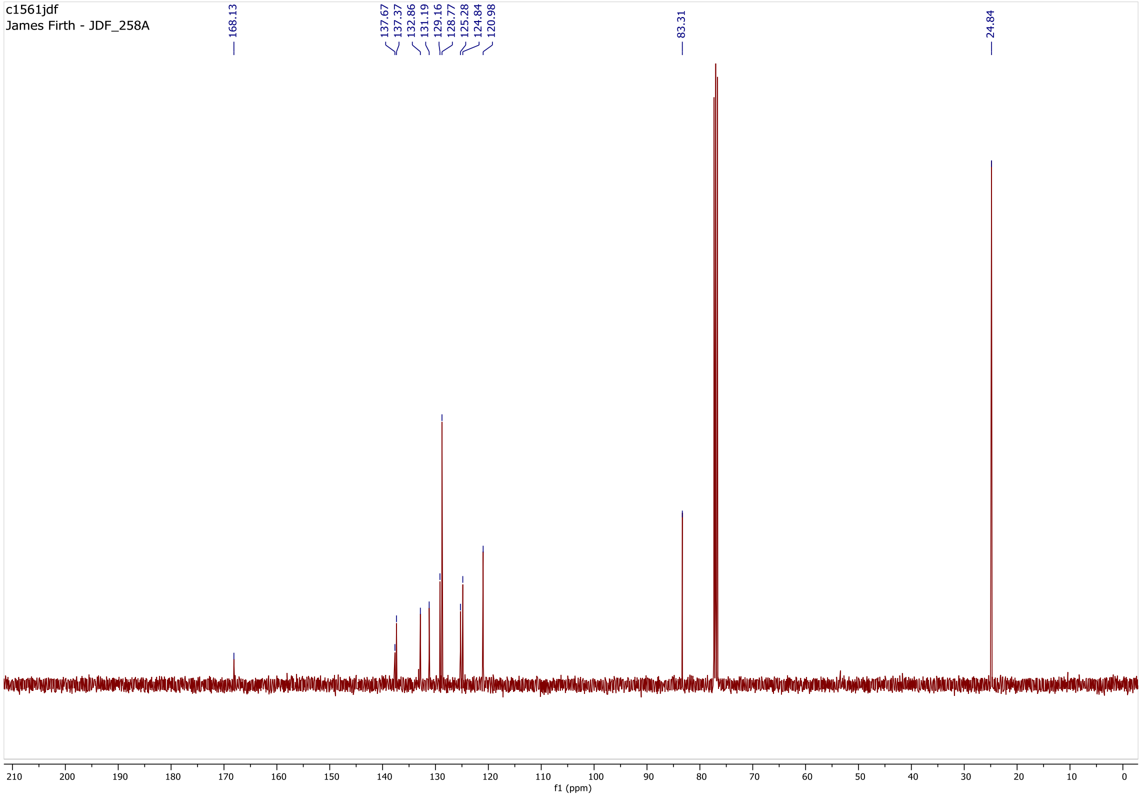

Supplementary Figure 49. Top: ^{1}H NMR spectrum of **S17** (reaction intermediate) (400 MHz, 298 K, $CDCl_3$). Bottom: ^{13}C NMR spectrum of **S17** (reaction intermediate) (101 MHz, 298 K, $CDCl_3$).

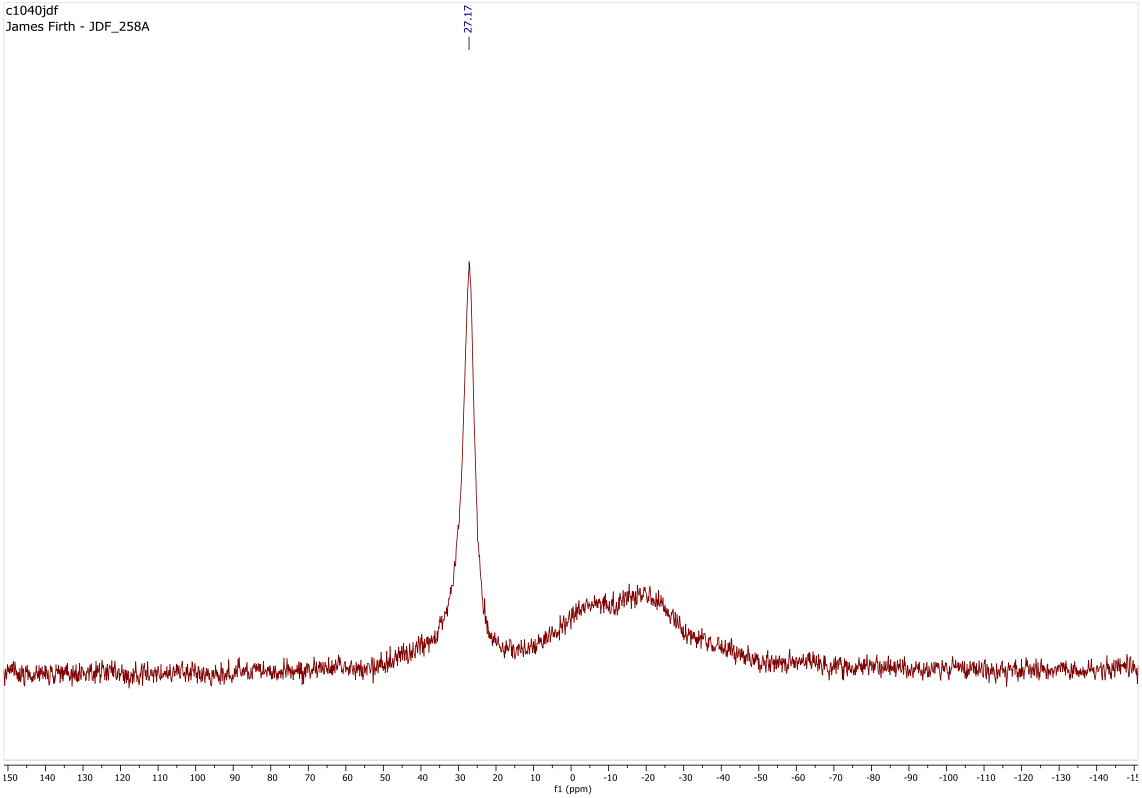

Supplementary Figure 50. ^{11}B NMR spectrum of **S17** (reaction intermediate) (128 MHz, 298 K, $CDCl_3$).

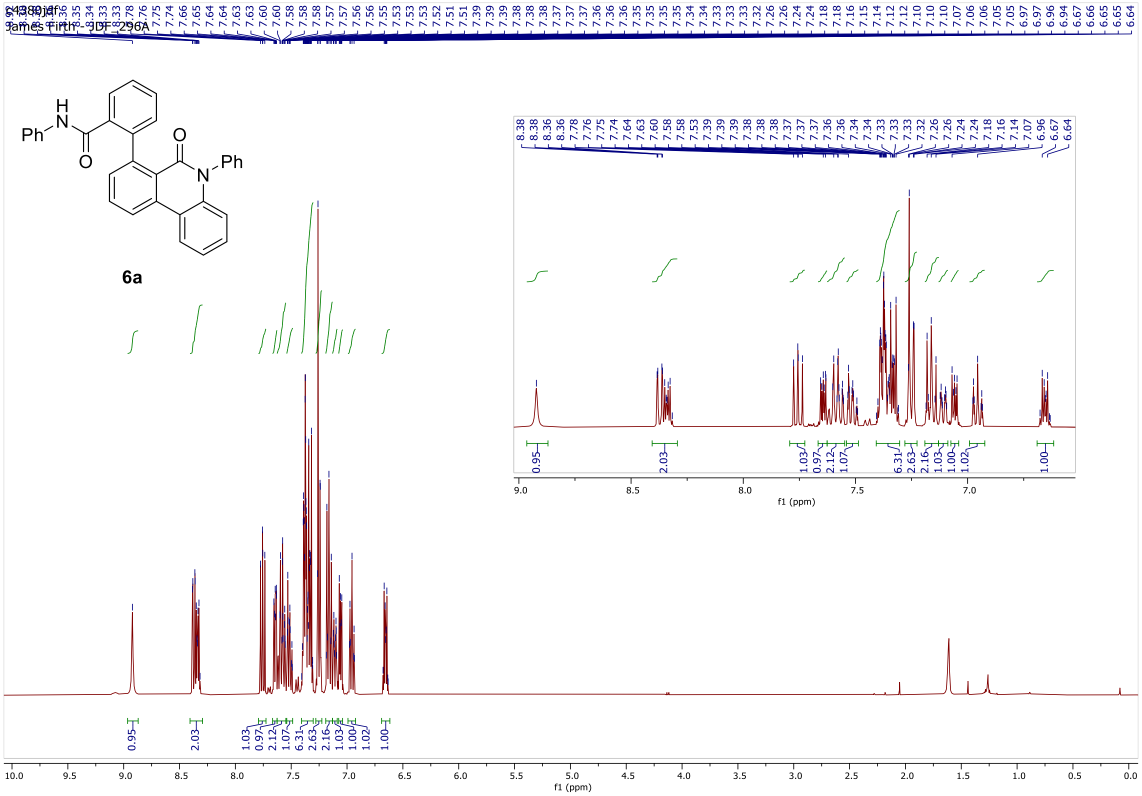

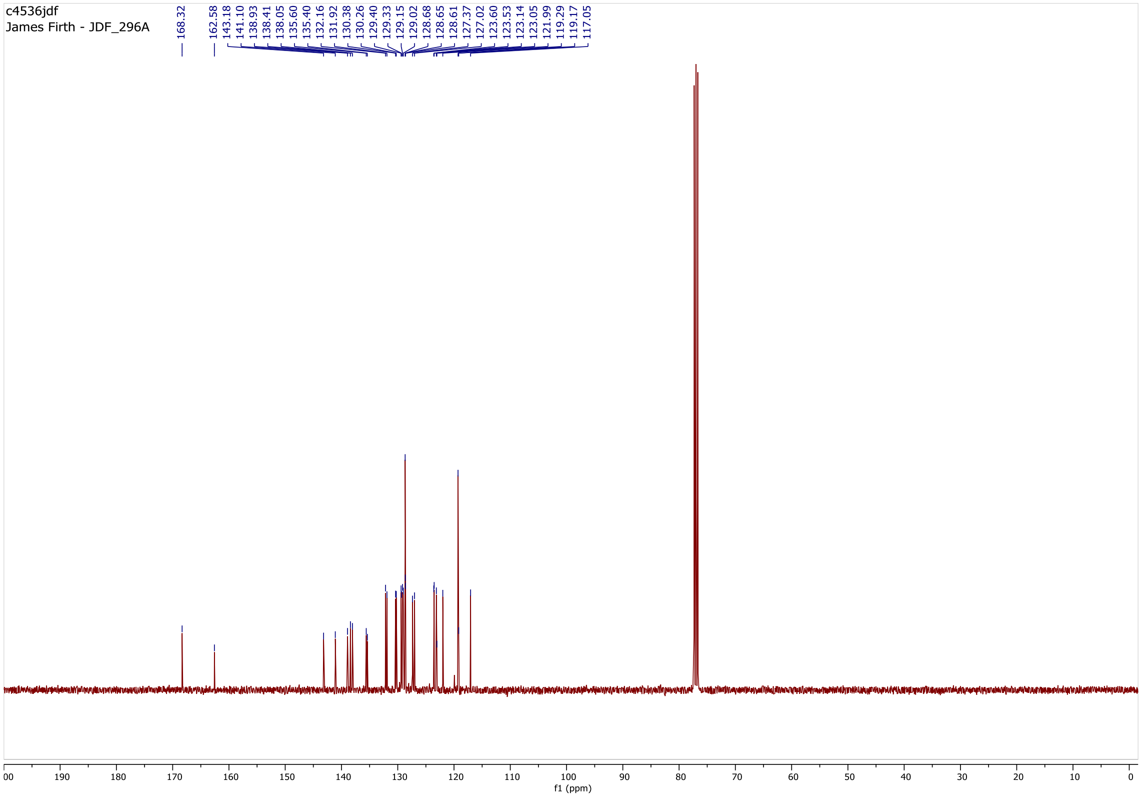

Supplementary Figure 51. Top: ^{1}H NMR spectrum of **6a** (independent sample) (400 MHz, 298 K, $CDCl_3$). Bottom: ^{13}C NMR spectrum of **6a** (independent sample) (101 MHz, 298 K, $CDCl_3$).

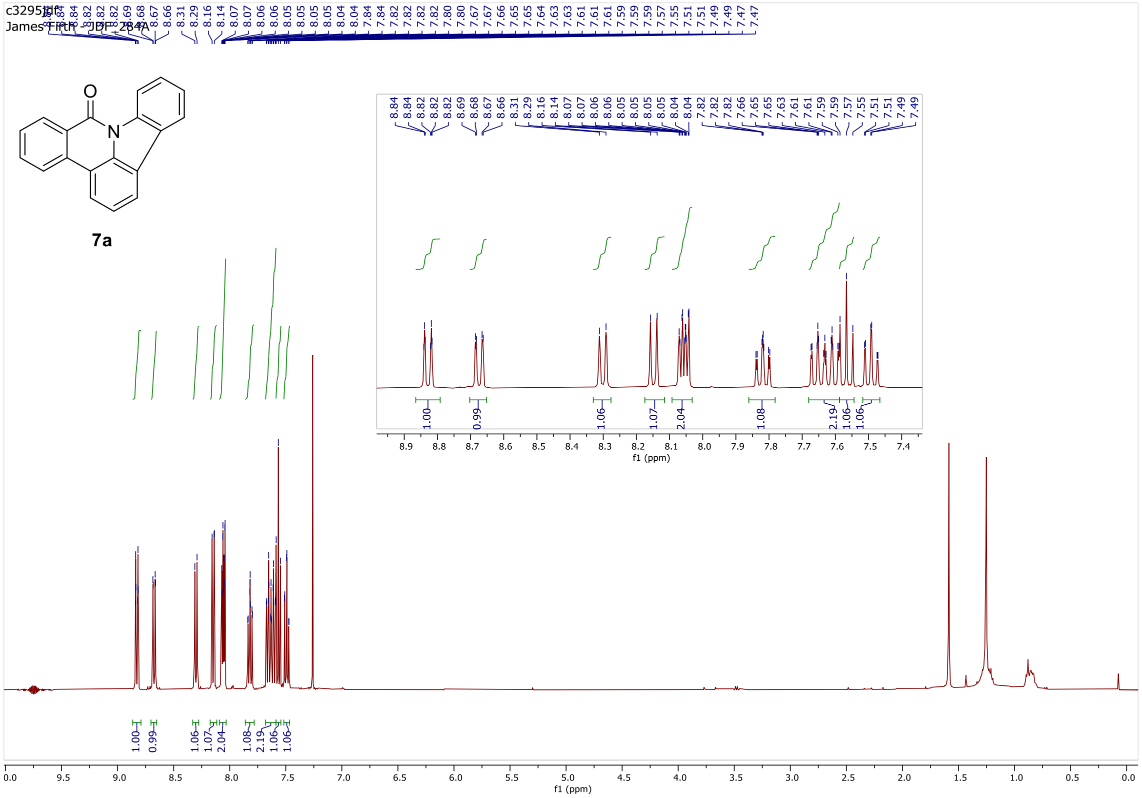

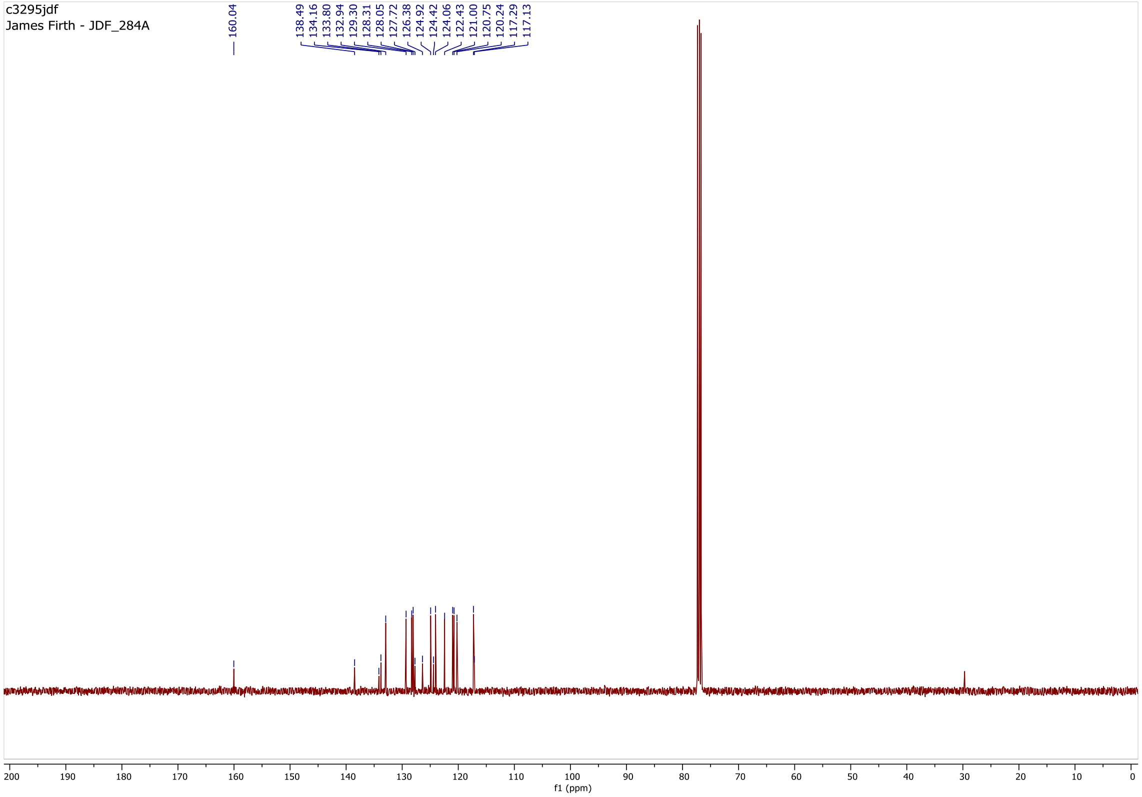

Supplementary Figure 52. Top: ^{1}H NMR spectrum of **7a** (independent sample) (400 MHz, 298 K, $CDCl_3$). Bottom: ^{13}C NMR spectrum of **7a** (independent sample) (101 MHz, 298 K, $CDCl_3$).

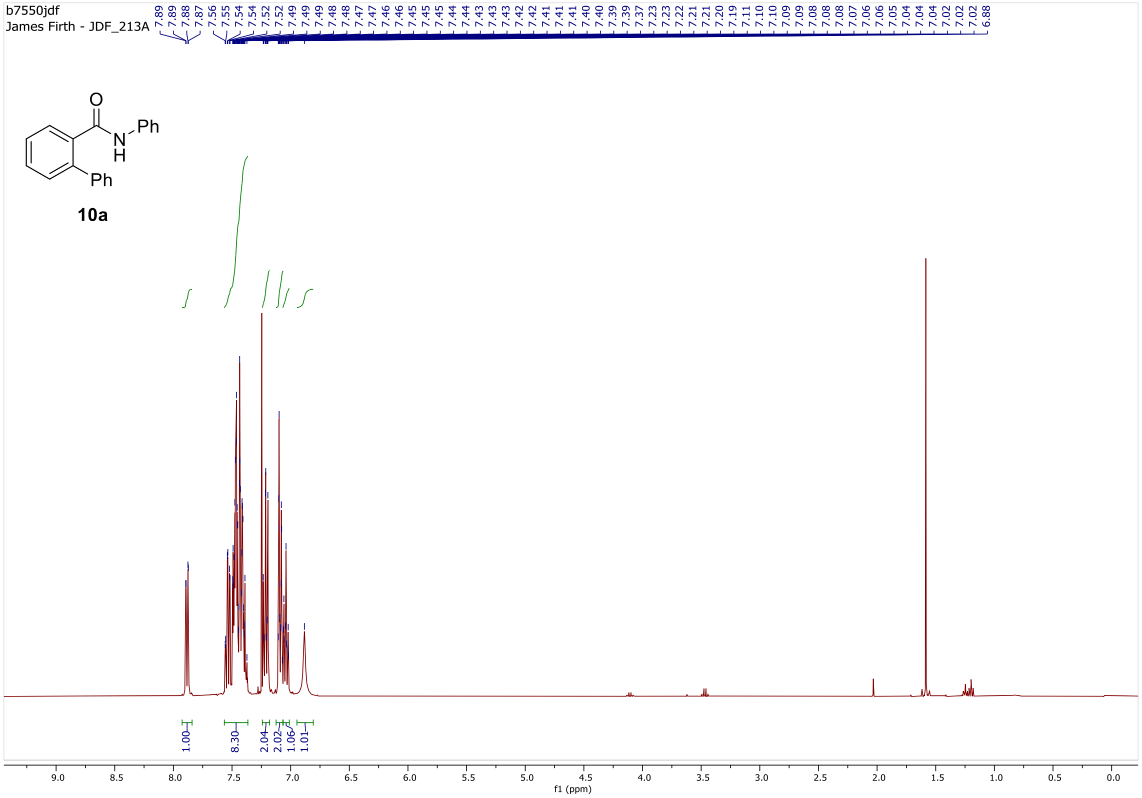

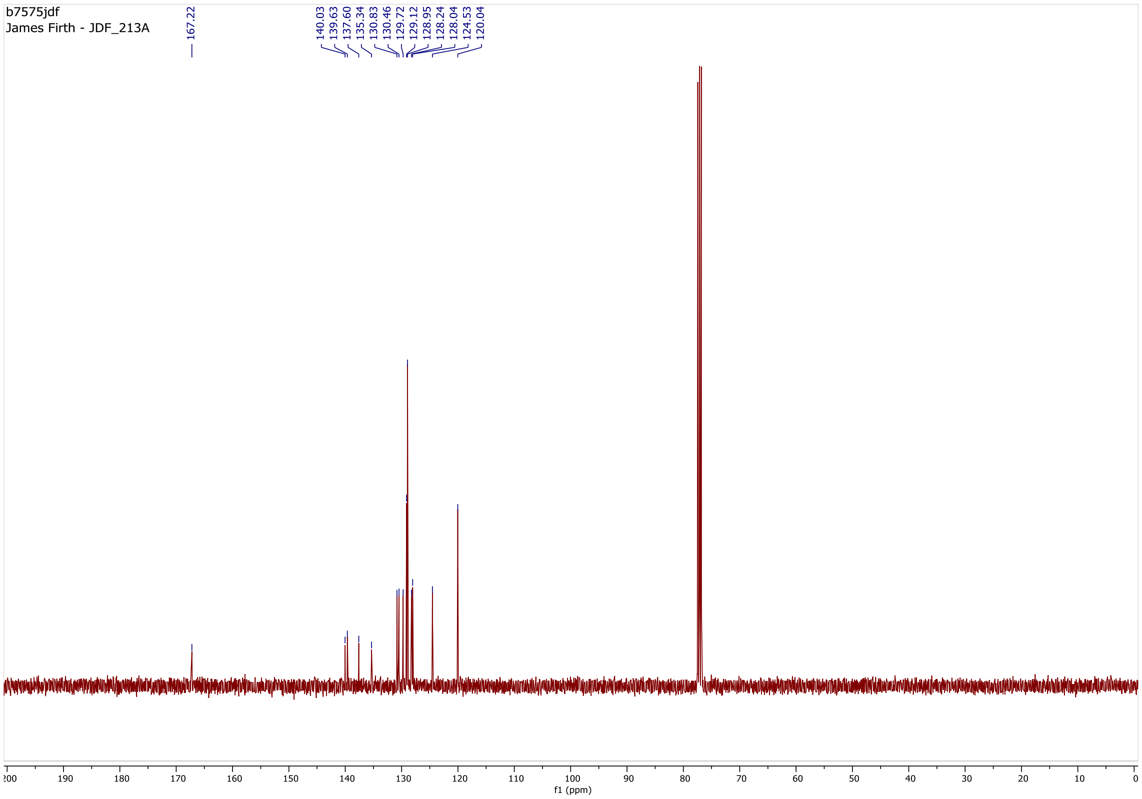

Supplementary Figure 53. Top: ^{1}H NMR spectrum of **10a** (400 MHz, 298 K, $CDCl_3$). Bottom: ^{13}C NMR spectrum of **10a** (101 MHz, 298 K, $CDCl_3$).

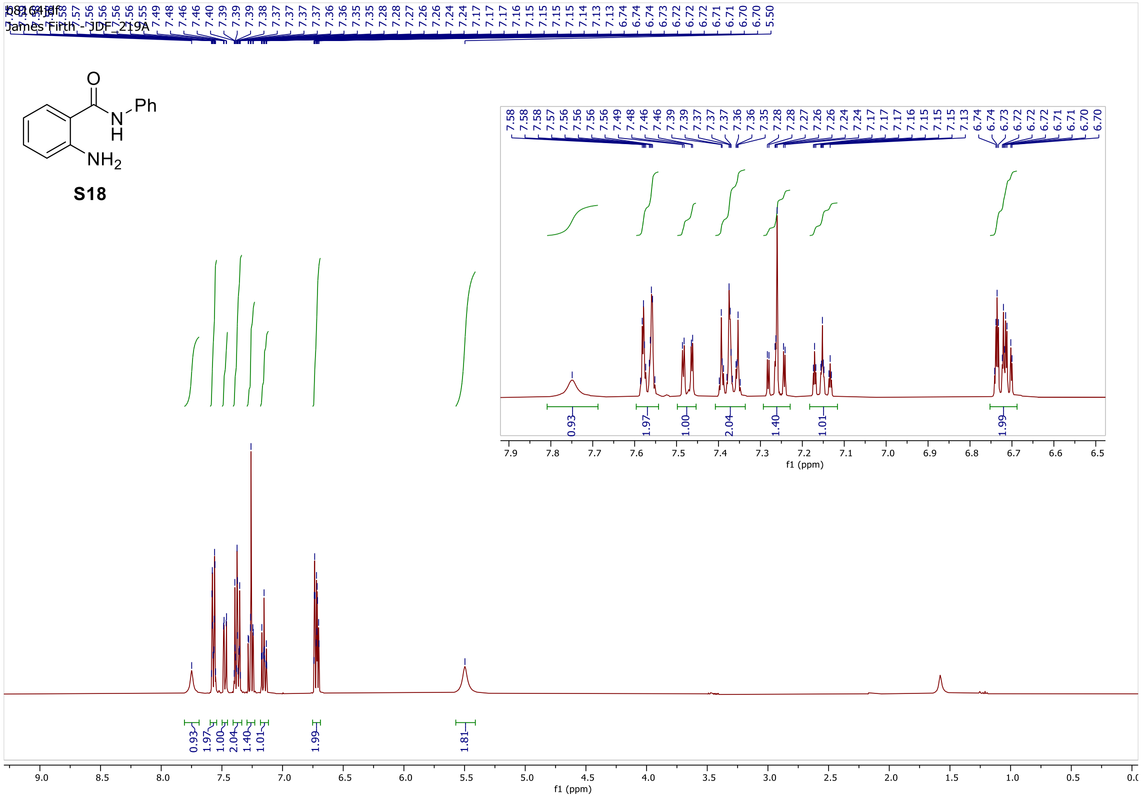

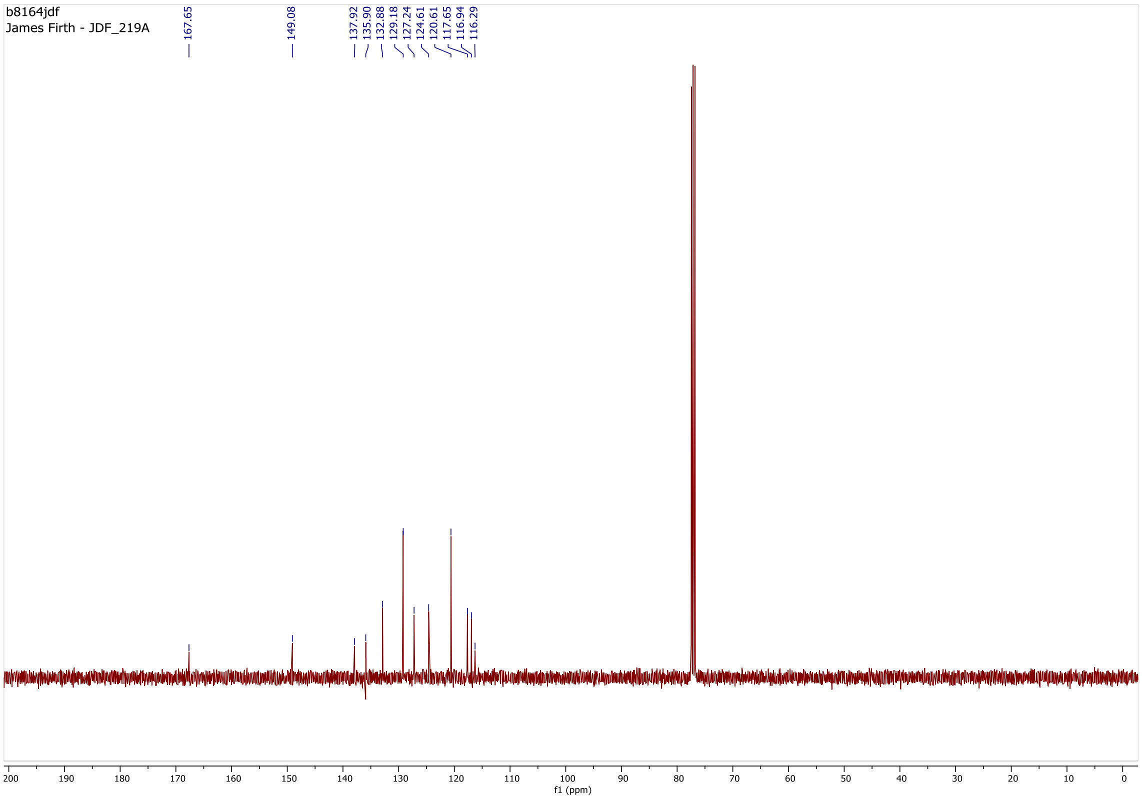

Supplementary Figure 54. Top: ^{1}H NMR spectrum of **S18** (400 MHz, 298 K, $CDCl_3$). Bottom: ^{13}C NMR spectrum of **S18** (101 MHz, 298 K, $CDCl_3$).

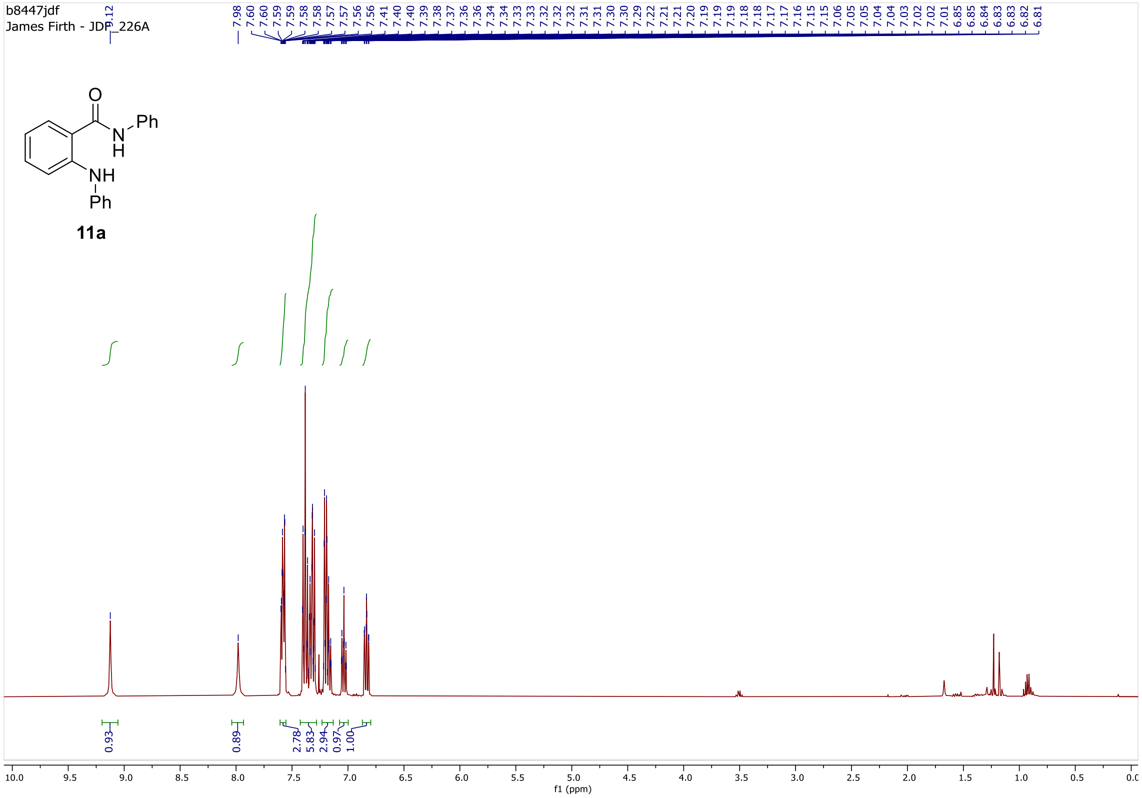

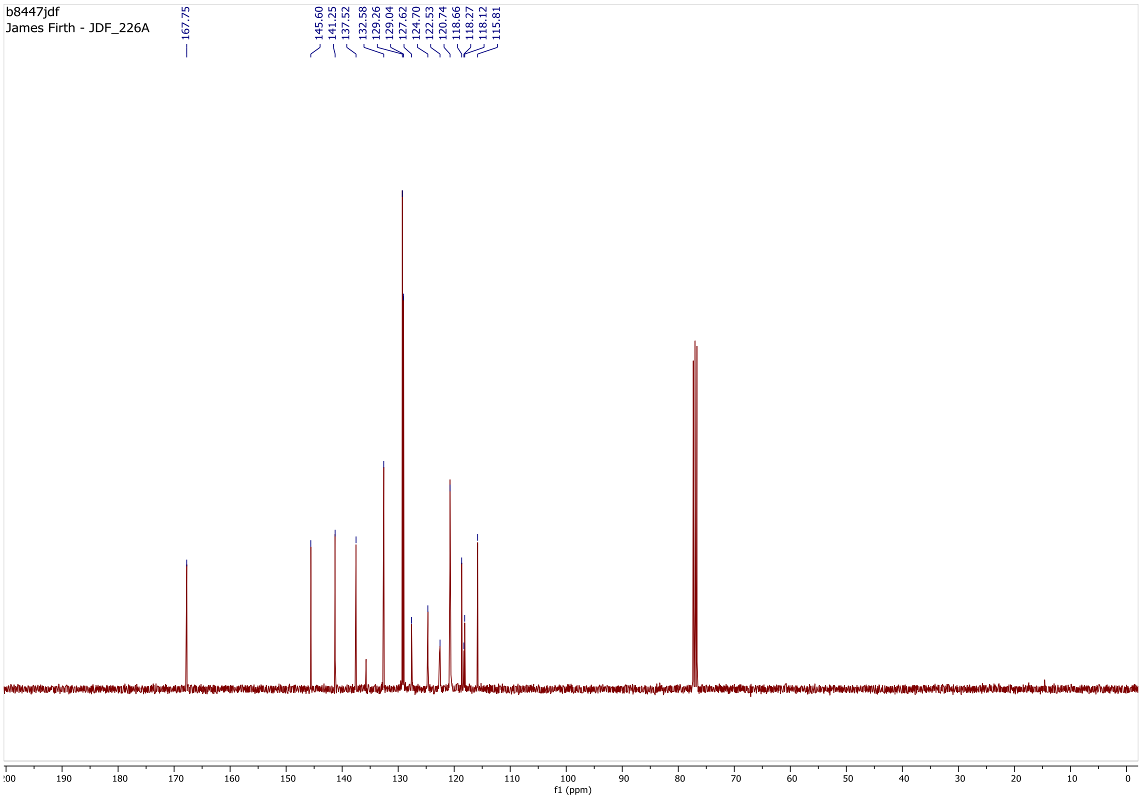

Supplementary Figure 55. Top: ^{1}H NMR spectrum of **11a** (400 MHz, 298 K, $CDCl_3$). Bottom: ^{13}C NMR spectrum of **11a** (101 MHz, 298 K, $CDCl_3$).

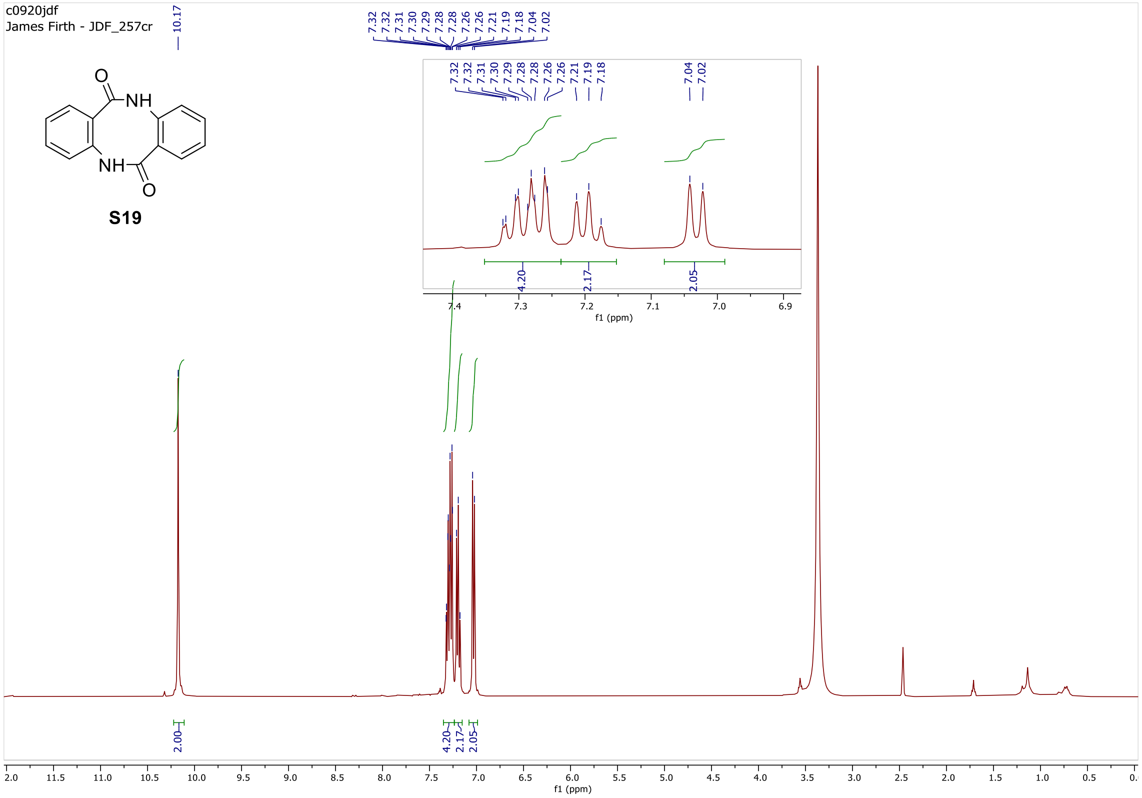

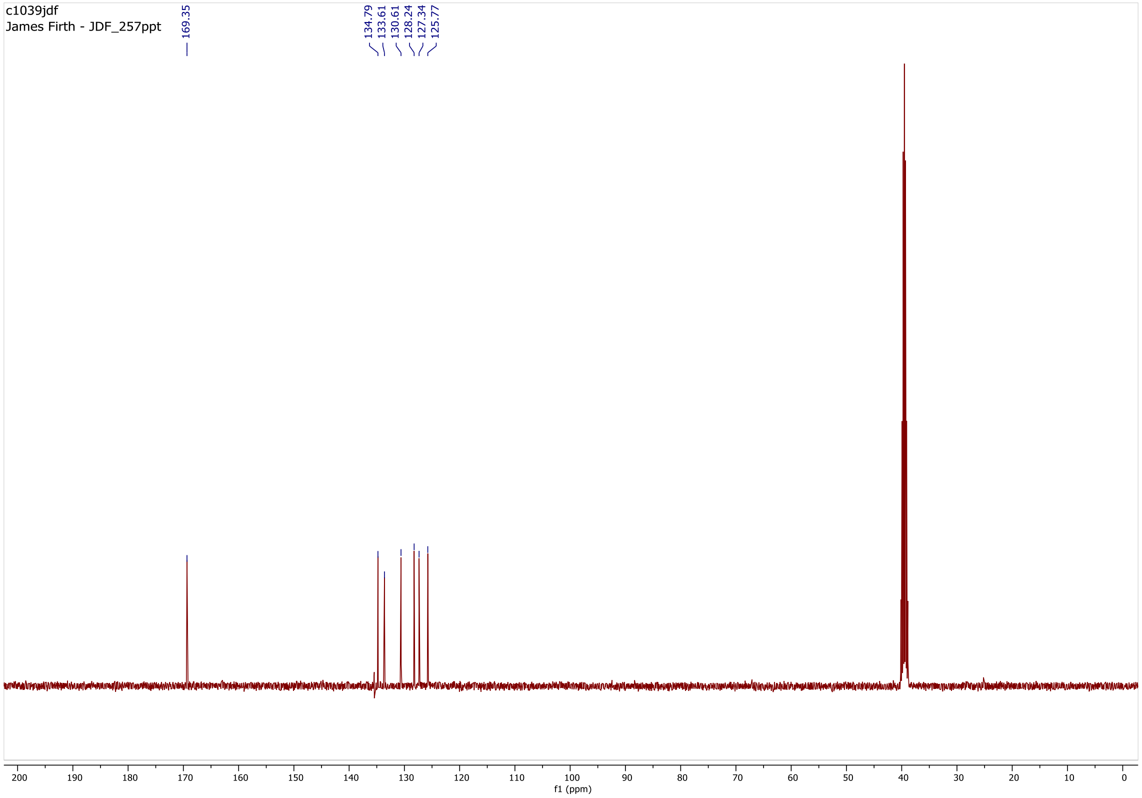

Supplementary Figure 56. Top: ^{1}H NMR spectrum of **S19** (400 MHz, 298 K, DMSO-d_6). Bottom: ^{13}C NMR spectrum of **S19** (101 MHz, 298 K, DMSO-d_6).

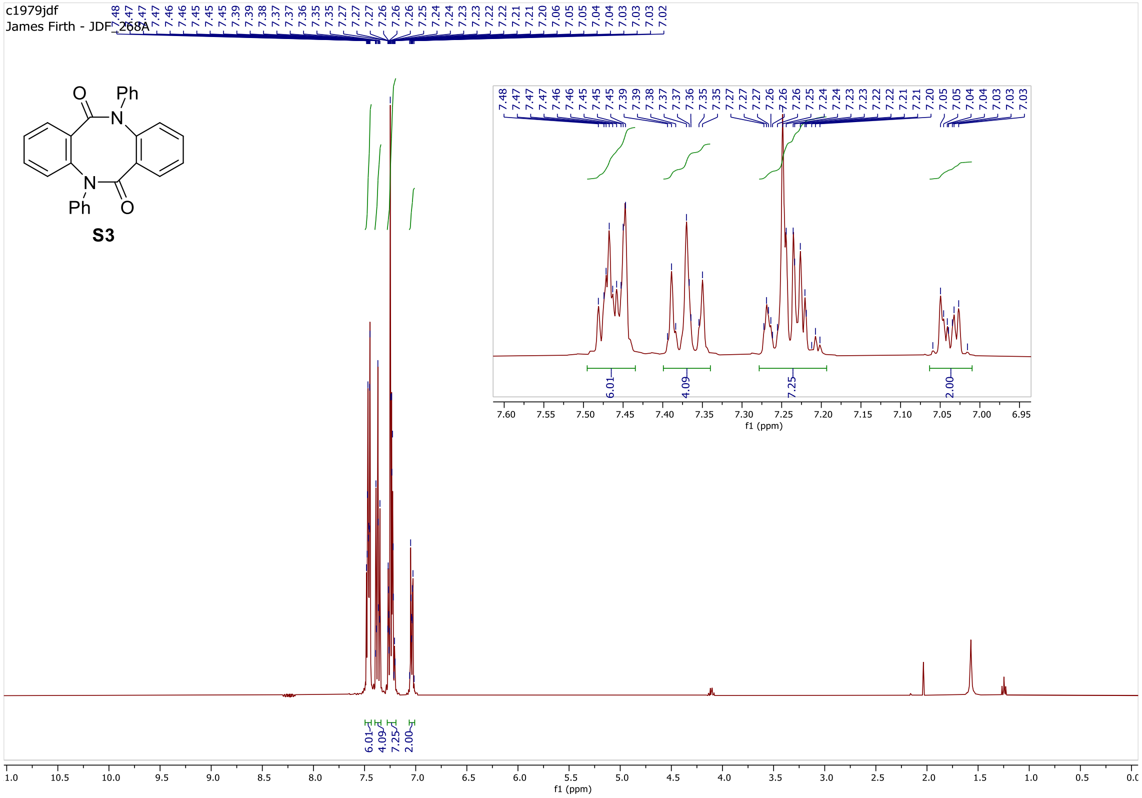

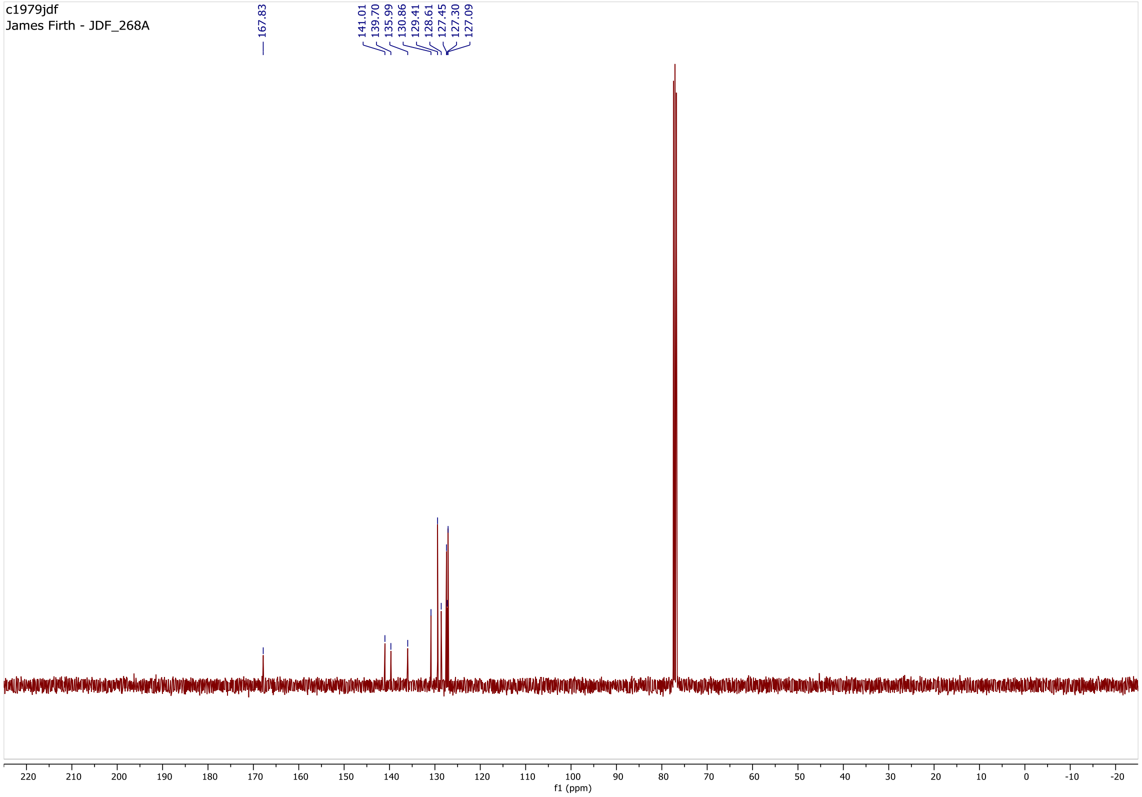

Supplementary Figure 57. Top: ^{1}H NMR spectrum of **S3** (400 MHz, 298 K, $CDCl_3$). Bottom: ^{13}C NMR spectrum of **S3** (101 MHz, 298 K, $CDCl_3$).

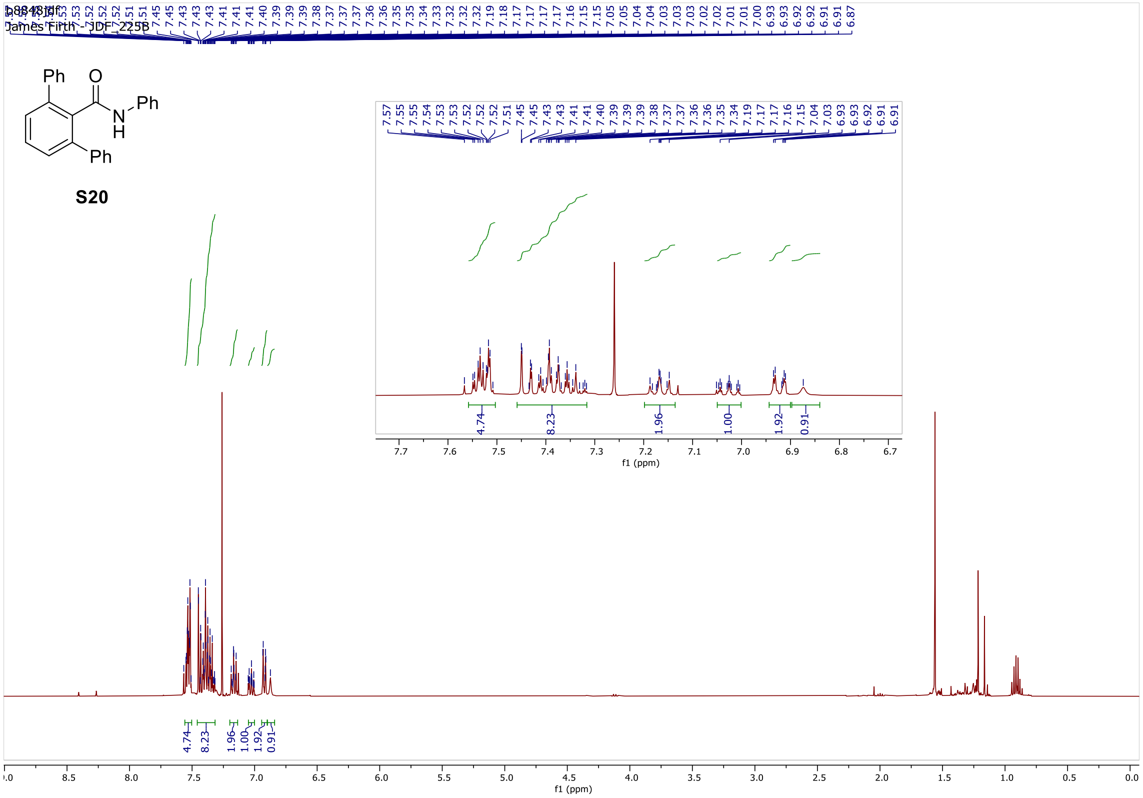

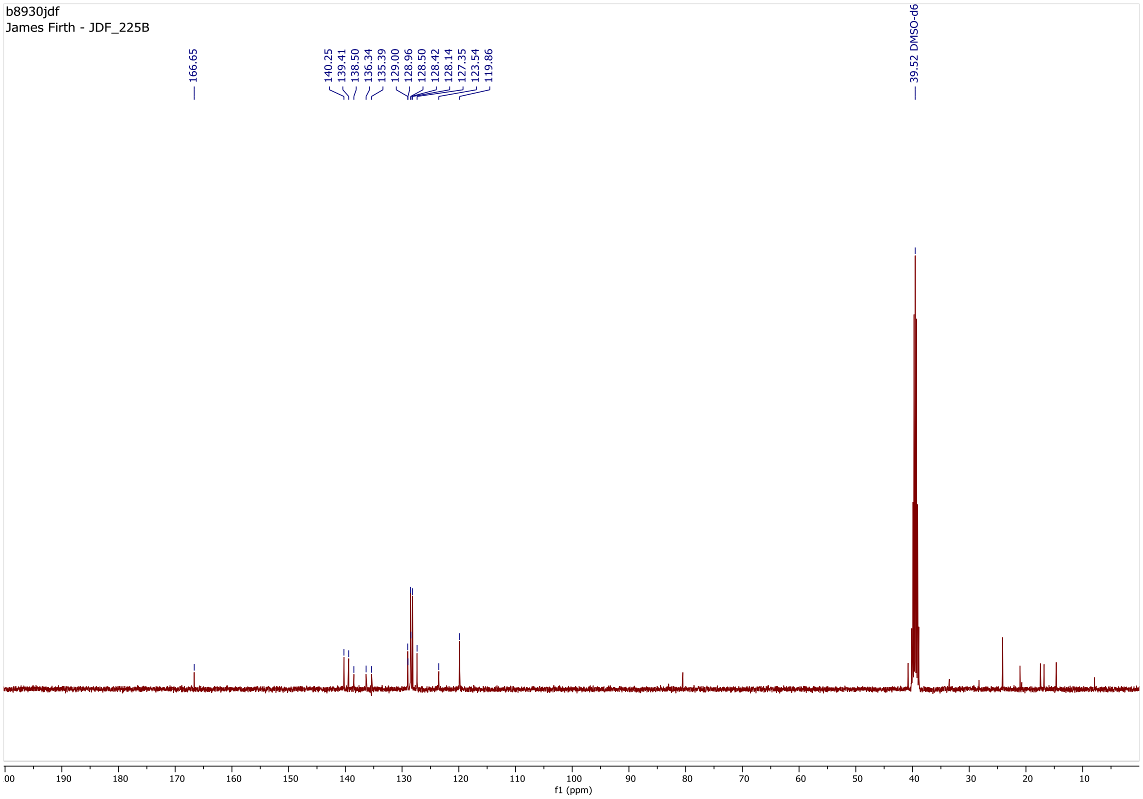

Supplementary Figure 58. Top: ^{1}H NMR spectrum of **S20** (400 MHz, 298 K, $CDCl_3$). Bottom: ^{13}C NMR spectrum of **S20** (101 MHz, 298 K, $CDCl_3$).

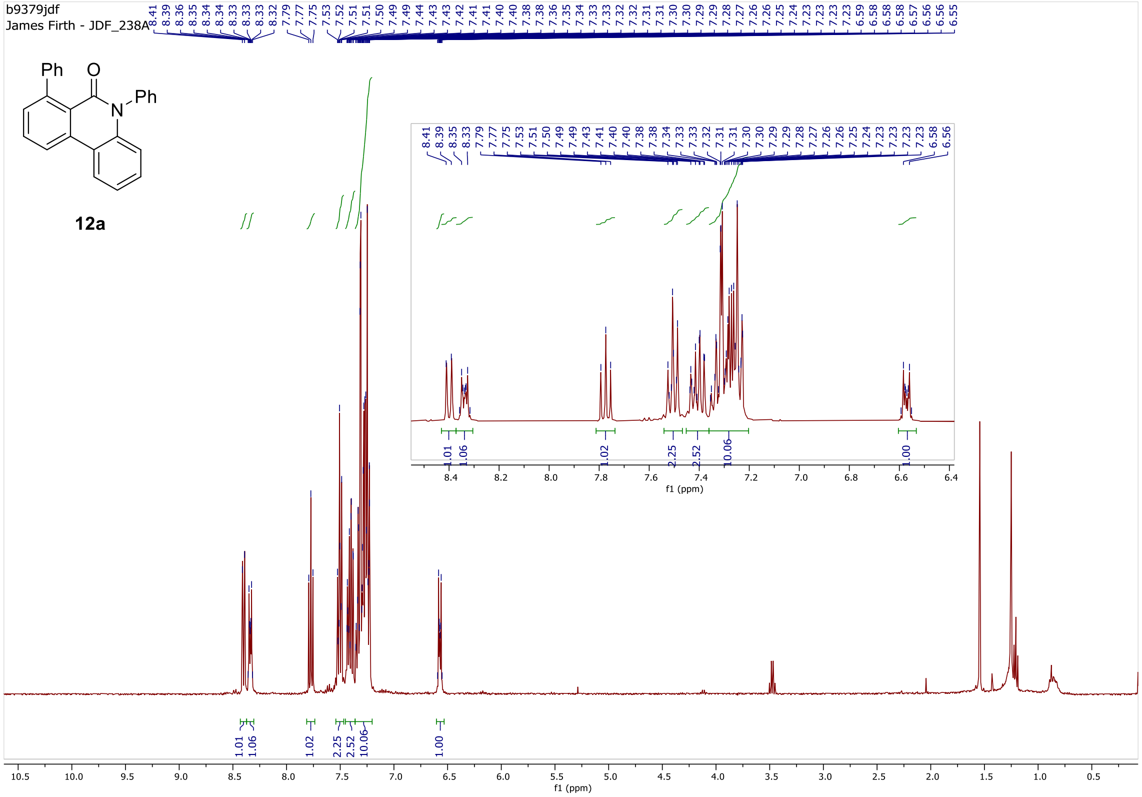

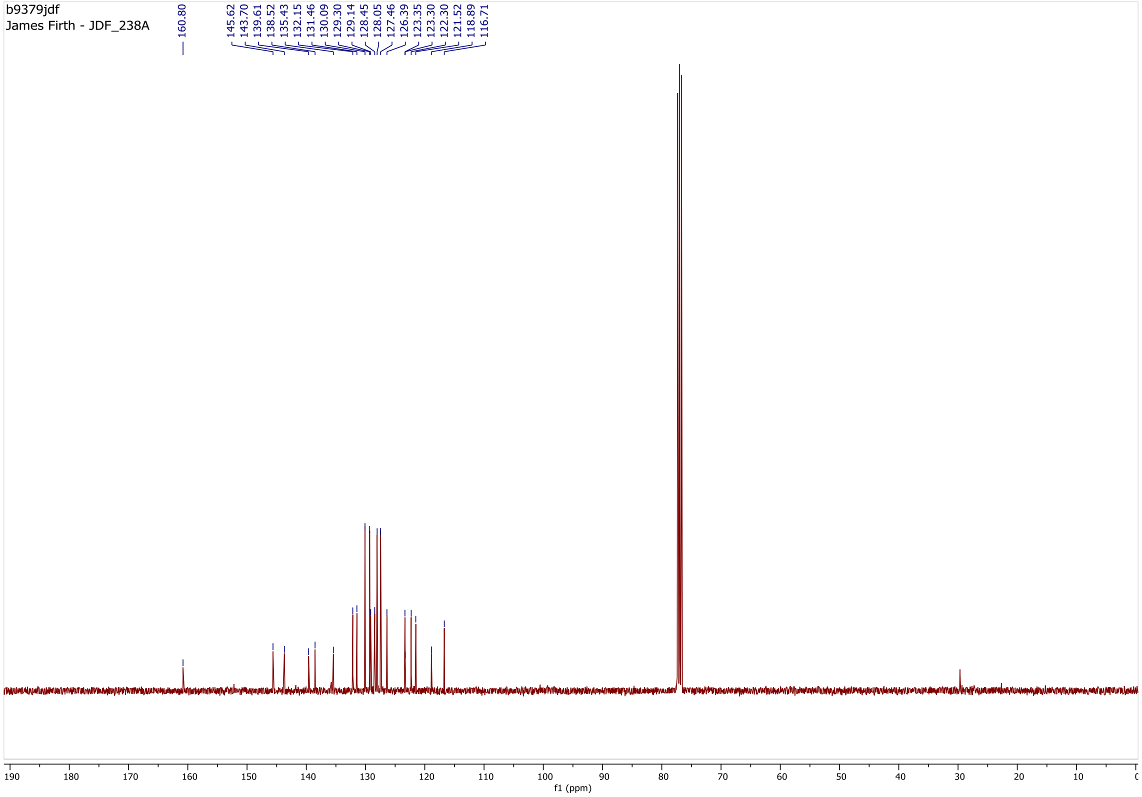

Supplementary Figure 59. Top: ^{1}H NMR spectrum of **12a** (400 MHz, 298 K, $CDCl_3$). Bottom: ^{13}C NMR spectrum of **12a** (101 MHz, 298 K, $CDCl_3$).

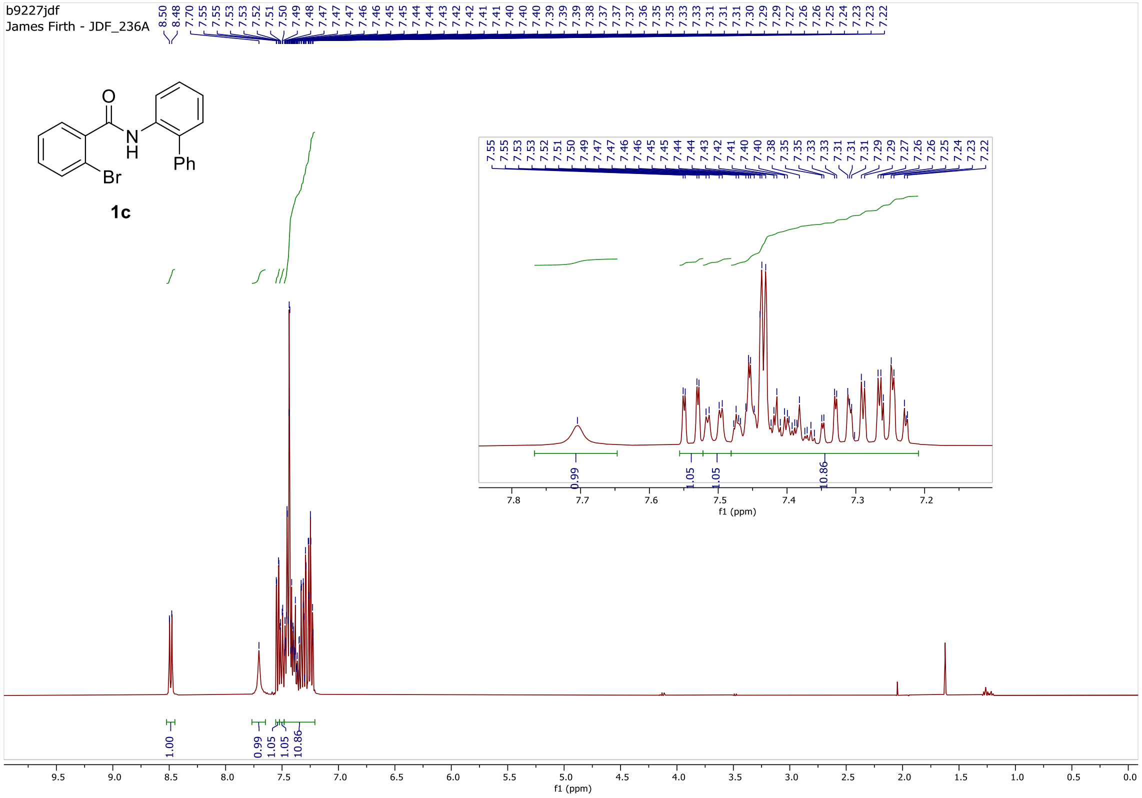

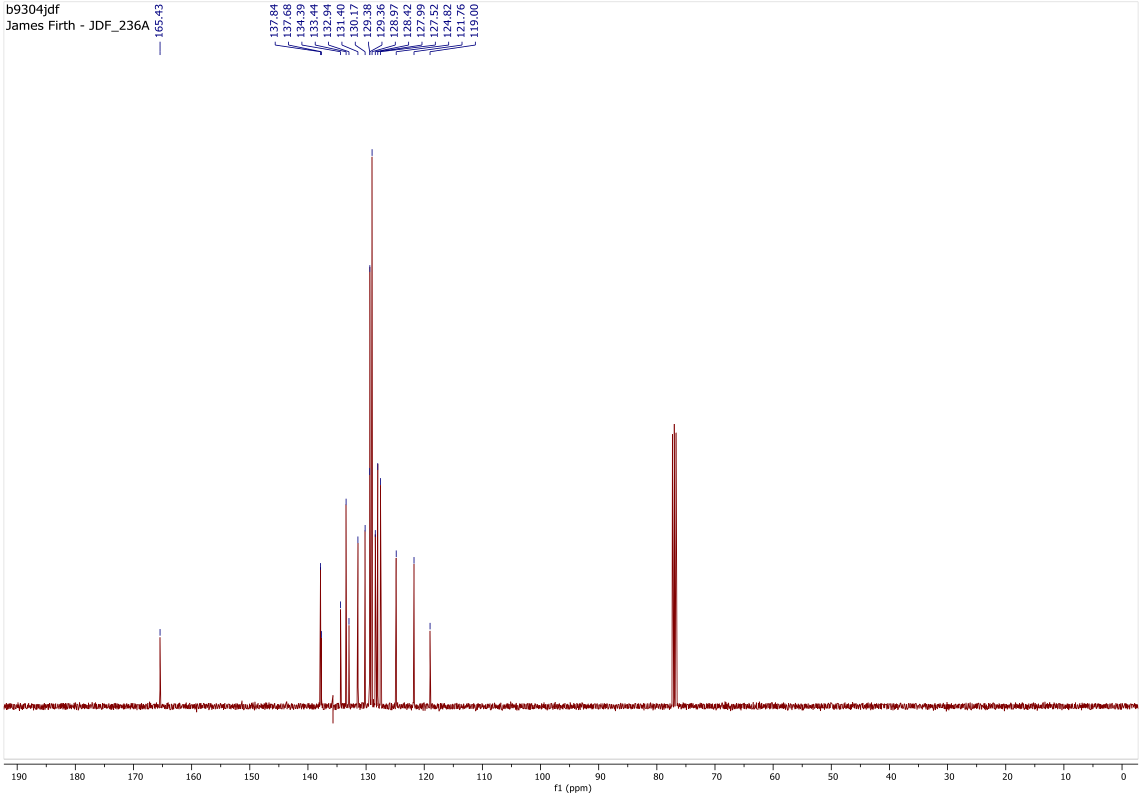

Supplementary Figure 60. Top: ^{1}H NMR spectrum of **1c** (400 MHz, 298 K, $CDCl_3$). Bottom: ^{13}C NMR spectrum of **1c** (101 MHz, 298 K, $CDCl_3$).

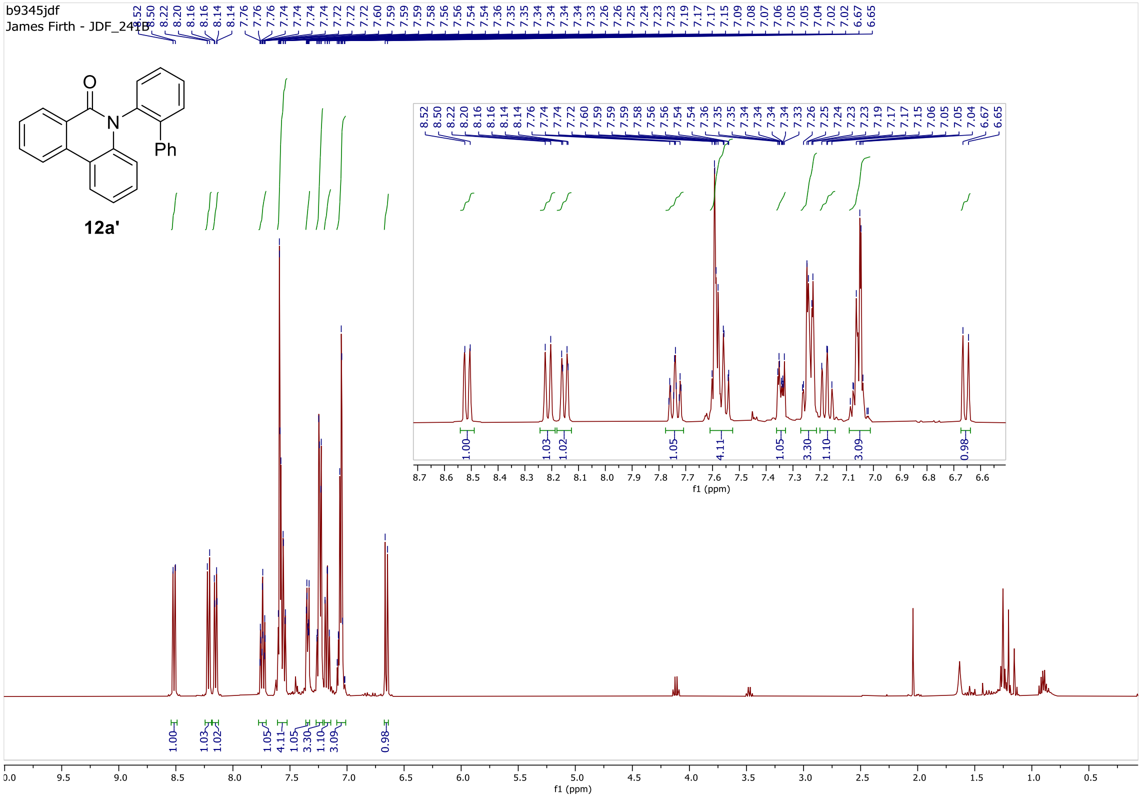

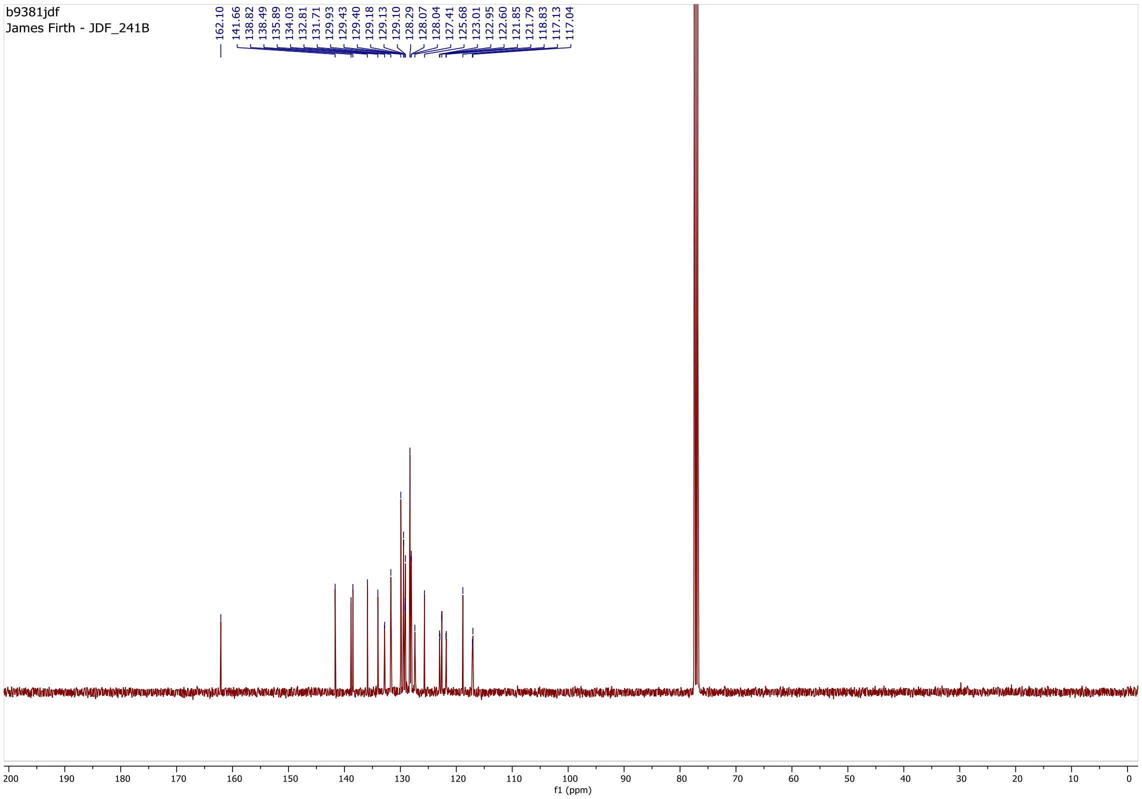

Supplementary Figure 61. Top: ^{1}H NMR spectrum of **12a’** (400 MHz, 298 K, $CDCl_3$). Bottom: ^{13}C NMR spectrum of **12a’** (101 MHz, 298 K, $CDCl_3$).

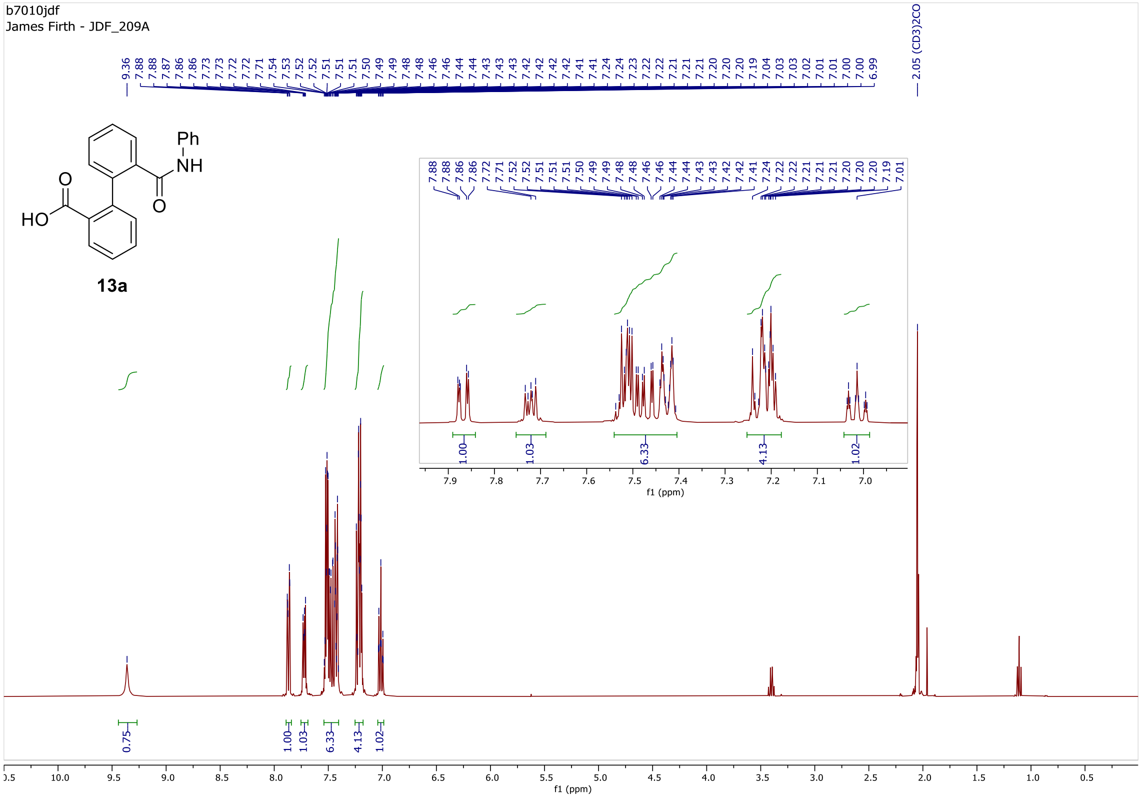

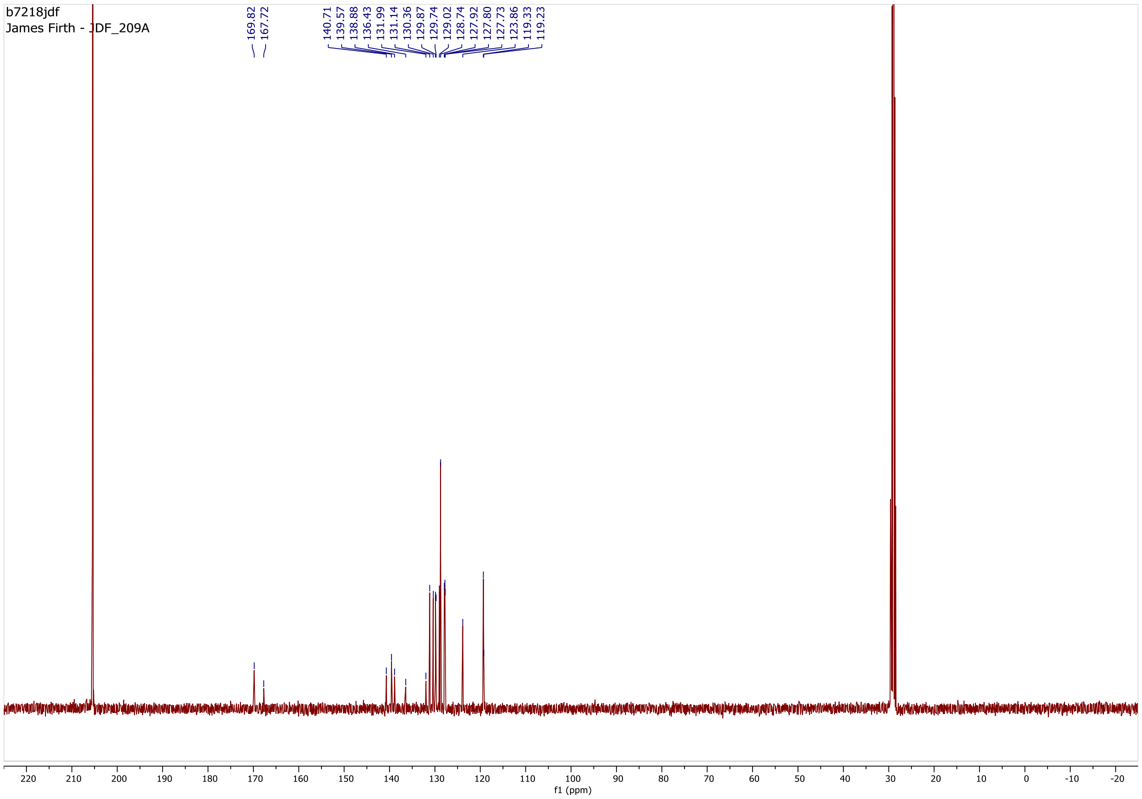

Supplementary Figure 62. Top: ^{1}H NMR spectrum of **13a** (400 MHz, 298 K, acetone-d_6). Bottom: ^{13}C NMR spectrum of **13a** (101 MHz, 298 K, acetone-d_6).

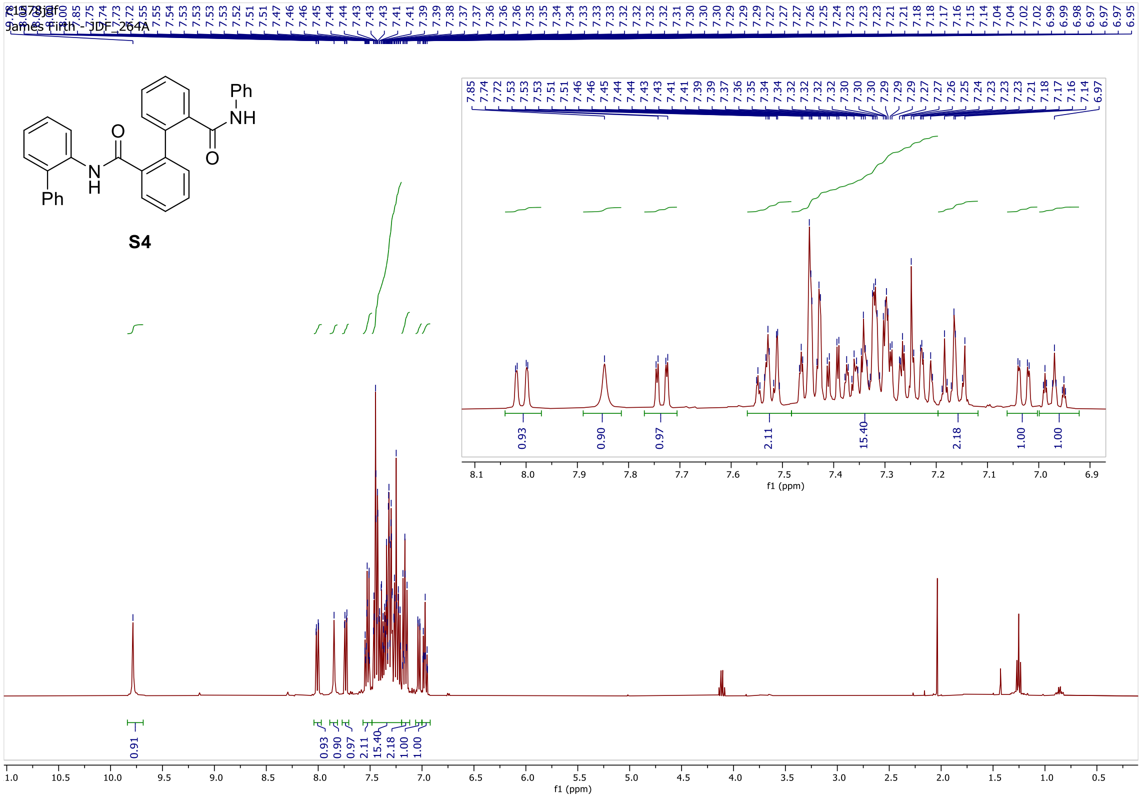

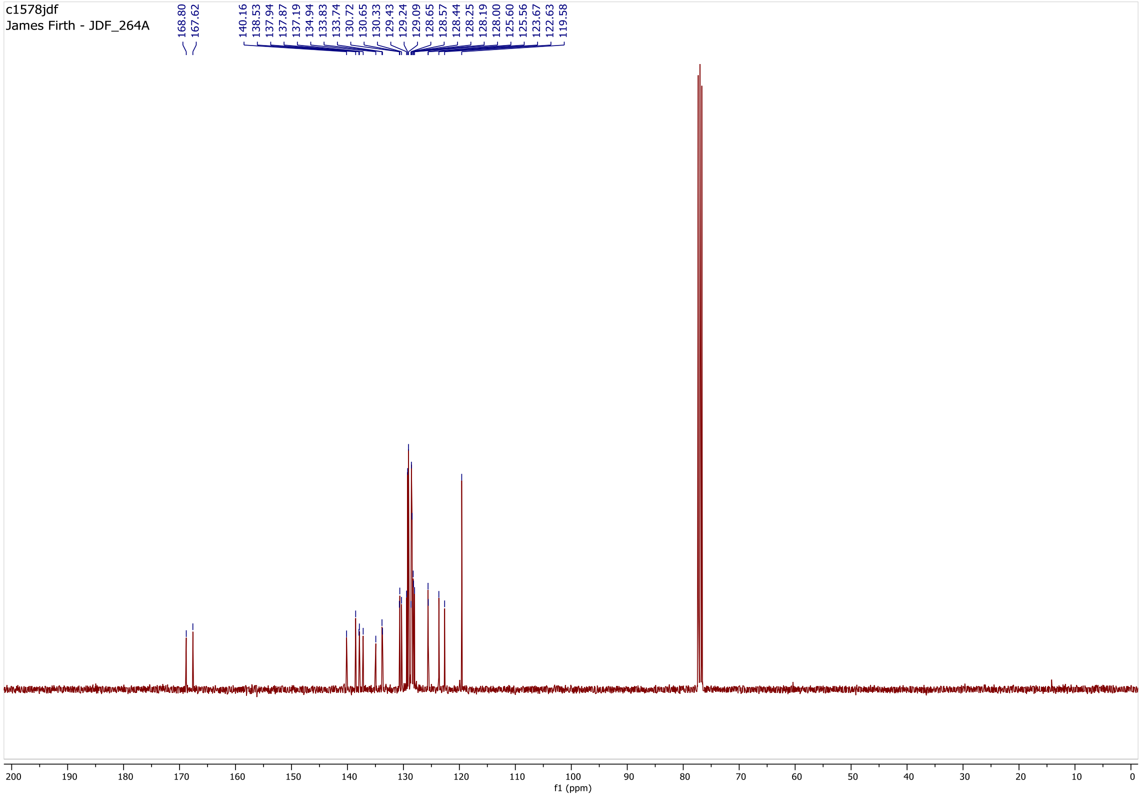

Supplementary Figure 63. Top: ^{1}H NMR spectrum of **S4** (400 MHz, 298 K, $CDCl_3$). Bottom: ^{13}C NMR spectrum of **S4** (101 MHz, 298 K, $CDCl_3$).

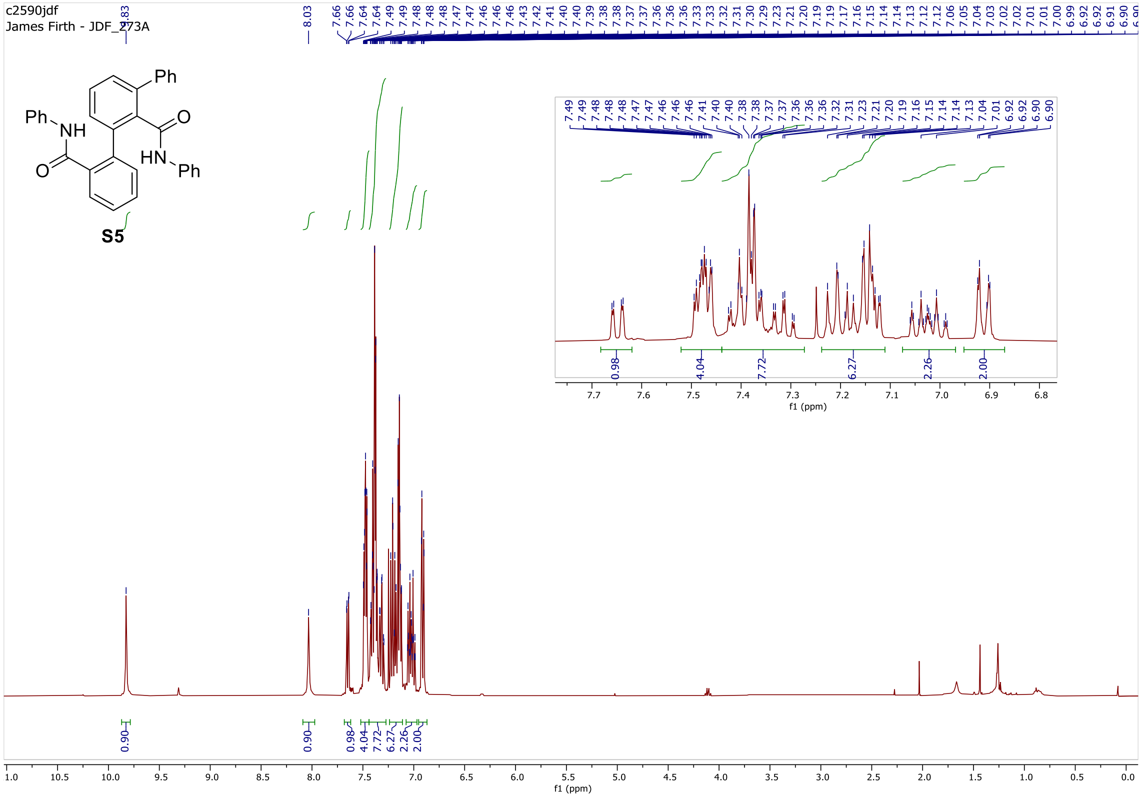

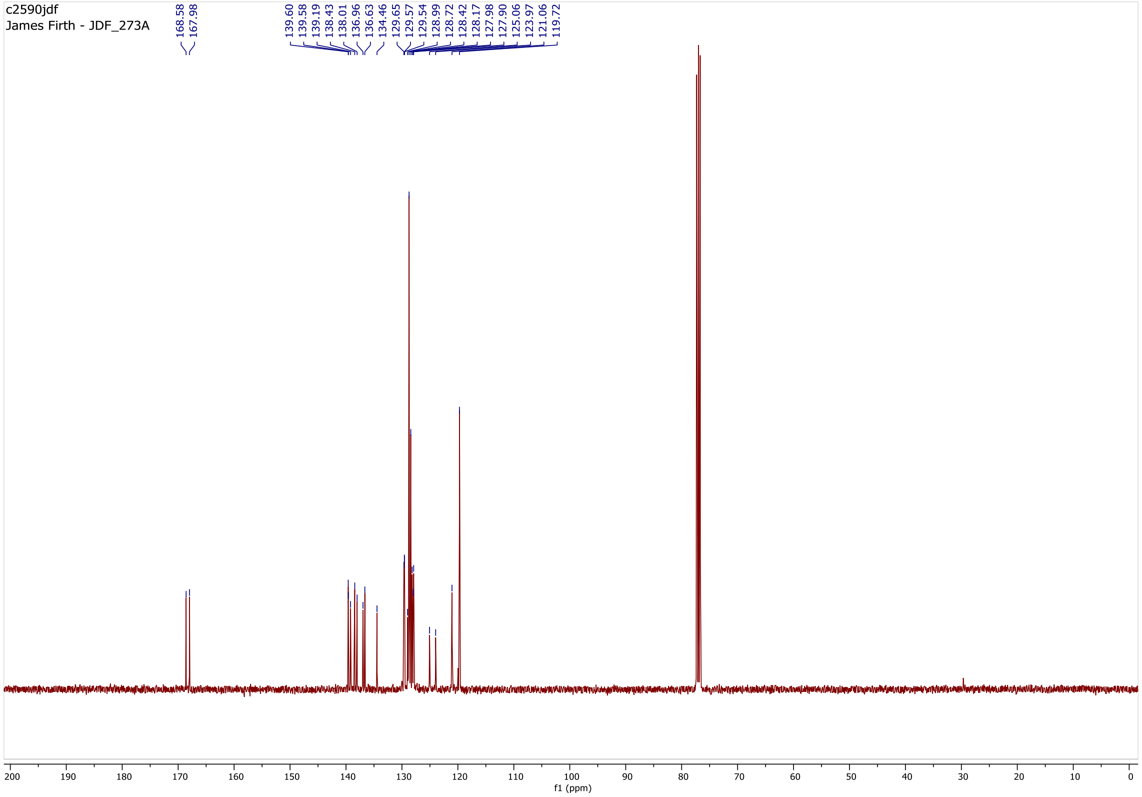

Supplementary Figure 64. Top: ^{1}H NMR spectrum of **S5** (400 MHz, 298 K, $CDCl_3$). Bottom: ^{13}C NMR spectrum of **S5** (101 MHz, 298 K, $CDCl_3$).

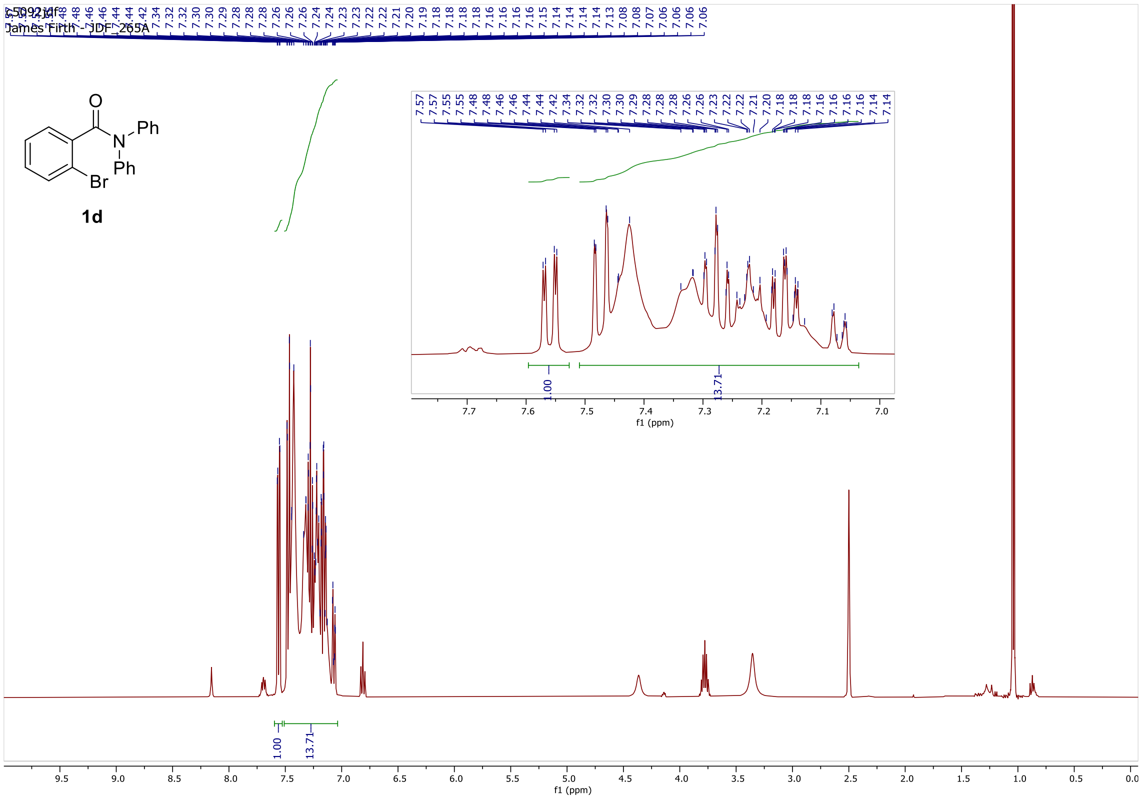

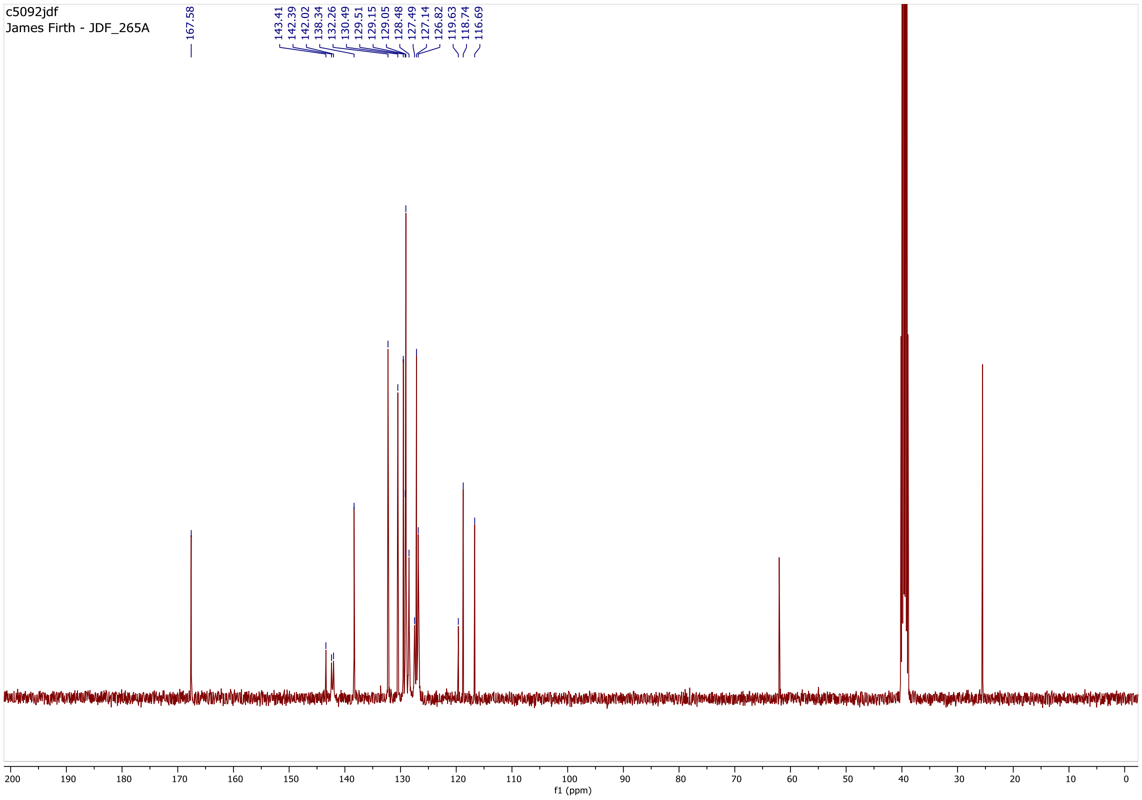

Supplementary Figure 65. Top: ^{1}H NMR spectrum of **1d** (400 MHz, 298 K, DMSO-d_6). Bottom: ^{13}C NMR spectrum of **1d** (101 MHz, 298 K, DMSO-d_6).

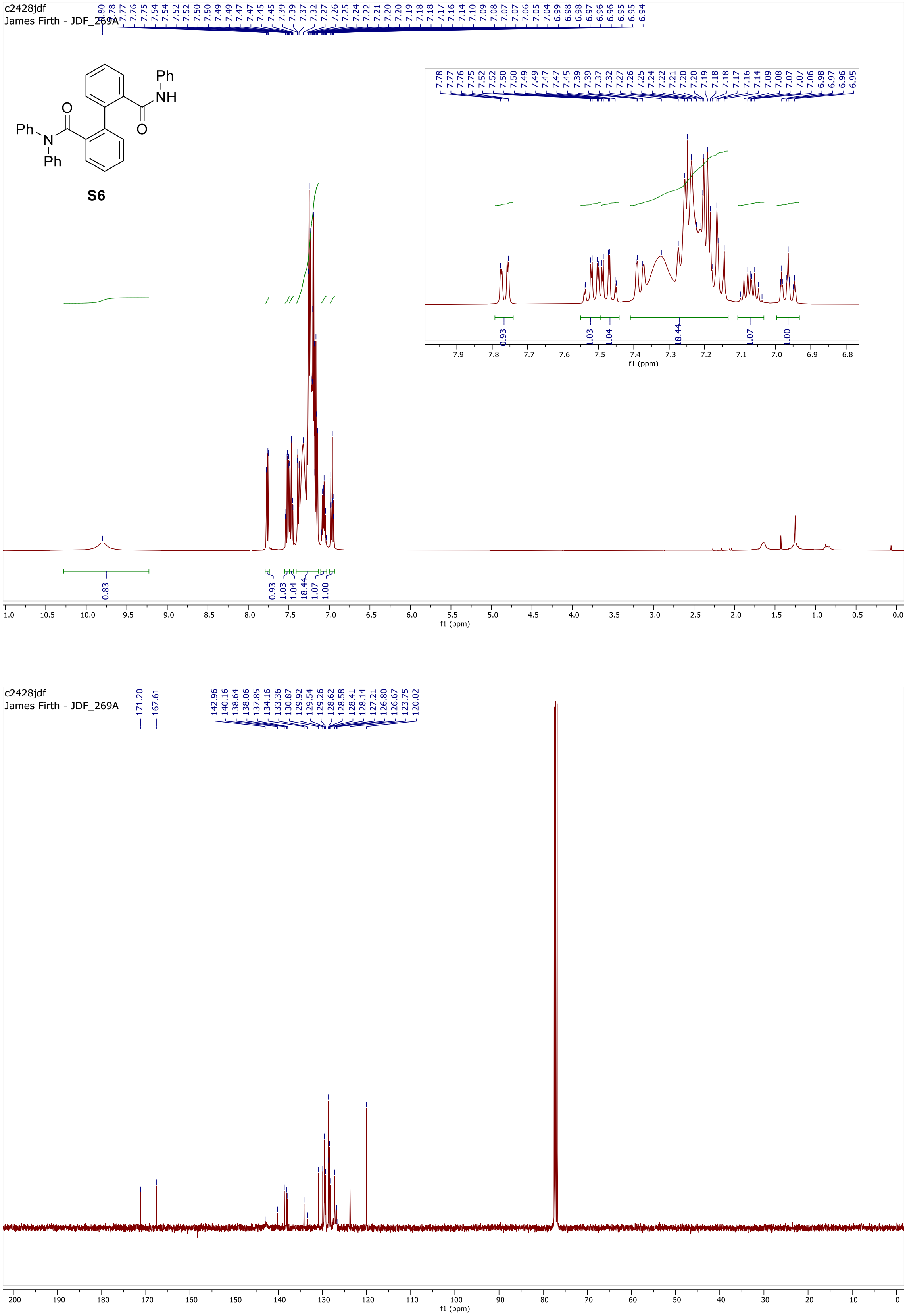

Supplementary Figure 66. Top: ^{1}H NMR spectrum of **S6** (400 MHz, 298 K, $CDCl_3$). Bottom: ^{13}C NMR spectrum of **S6** (101 MHz, 298 K, $CDCl_3$).

6. References

(1) *CrysAlis Pro, Rigaku Oxford Diffraction. Version 1.171.40.84a*.

(2) Empirical absorption correction using spherical harmonics, implemented in SCALE3 ABSPACK scaling algorithm within CrysAlisPro software, Oxford Diffraction Ltd. Version 1.171.34.40.

(3) Dolomanov, O. V; Bourhis, L. J.; Gildea, R. J.; Howard, J. A. K.; Puschmann, H. *J. Appl. Crystallogr.* **2009**, *42*, 339–341.

(4) Palatinus, L.; Chapuis, G. *J. Appl. Crystallogr.* **2007**, *40*, 786–790.

(5) Sheldrick, G. M. *Acta Crystallogr. Sect. A* **2015**, *71*, 3–8.

(6) Sheldrick, G. M. *Acta Crystallogr. Sect. C* **2015**, *71*, 3–8.

(7) Nonlinear Dynaminc, www.nonlinear.com (accessed Feb 15, 2021).

(8) R Core Team (2020). R Foundation for Statistical Computing, Vienna, Austria. https://www.R-project.org/ (accessed Feb 15, 2021).

(9) Jarvis, A. G.; Sehnal, P. E.; Bajwa, S. E.; Whitwood, A. C.; Zhang, X.; Cheung, M. S.; Lin, Z.; Fairlamb, I. J. S. *Chem. Eur. J.* **2013**, *19*, 6034–6043.

(10) Wang, Y.; Zhu, D.; Tang, L.; Wang, S.; Wang, Z. *Angew. Chem. Int. Ed.* **2011**, *50*, 8917–8921.

(11) Liu, H.; Han, W.; Li, C.; Ma, Z.; Li, R.; Zheng, X.; Fu, H.; Chen, H. *Eur. J. Org. Chem.* **2016**, 389–393.

(12) Glover, S. A.; Goosen, A. *J. Chem. Soc. Perkin Trans. 1* **1974**, 2353–2356.

(13) Goswami, S.; Adak, A. K.; Mukherjee, R.; Jana, S.; Dey, S.; Gallagher, J. F. *Tetrahedron* **2005**, *61*, 4289–4295.

(14) Ferraccioli, R.; Carenzi, D.; Motti, E.; Catellani, M. *J. Am. Chem. Soc.* **2006**, *128*, 722–723.

(15) Gehring, A. P.; Tremmel, T.; Bracher, F. *Synthesis* **2014**, *46*, 893–898.

(16) Nimnual, P.; Tummatorn, J.; Thongsornkleeb, C.; Ruchirawat, S. *J. Org. Chem.* **2015**, *80*, 8657–8667.

(17) Crawford, S. M.; Alsabeh, P. G.; Stradiotto, M. *Eur. J. Org. Chem.* **2012**, 6042–6050.

(18) Navarro, O.; Marion, N.; Oonishi, Y.; Kelly, R. A.; Nolan, S. P. *J. Org. Chem.* **2006**, *71*, 685–692.

(19) Tan, Y.; Hartwig, J. F. *J. Am. Chem. Soc.* **2010**, *132*, 3676–3677.

(20) Shen, G.; Wang, Y.; Zhao, X.; Huangfu, X.; Tian, Y.; Zhang, T.; Yang, B. *Synlett* **2017**, *28*, 2030–2035.

(21) Burger, A.; Schmalz, A. C. *J. Org. Chem.* **1954**, *19*, 1841–1846.

(22) Singh, V. P.; Singh, H. B.; Butcher, R. J. *Eur. J. Org. Chem.* **2011**, 5485–5497.

(23) Uchiyama, M.; Ozawa, H.; Takuma, K.; Matsumoto, Y.; Yonehara, M.; Hiroya, K.; Sakamoto, T. *Org. Lett.* **2006**, *8*, 5517–5520.

(24) Liu, Y.-C.; Huang, Z.-Y.; Chen, Q.; Yang, G.-F. *Tetrahedron* **2013**, *69*, 9025–9032.

(25) Li, J.; Ackermann, L. *Chem. Eur. J.* **2015**, *21*, 5718–5722.

(26) Wu, L.; Hao, Y.; Liu, Y.; Wang, Q. *Org. Biomol. Chem.* **2019**, *17*, 6762–6770.

(27) Zhang, S.; Tan, Z.; Xiong, B.; Jiang, H. F.; Zhang, M. *Org. Biomol. Chem.* **2018**, *16*, 531–535.

(28) Sorenson, R. J. *J. Org. Chem.* **2000**, *65*, 7747–7749.

(29) Novanna, M.; Kannadasan, S.; Shanmugam, P. *Tetrahedron Lett.* **2019**, *60*, 151163.

(30) Pettersson, B.; Bergman, J.; Svensson, P. H. *Tetrahedron* **2013**, *69*, 2647–2654.

(31) Corral, C.; Madroñcro, I.; Vrga, S. *J. Heterocycl. Chem.* **1977**, *14*, 99–102.

(32) Du, C. J. F.; Hart, H.; Ng, K. K. D. *J. Org. Chem.* **1986**, *51*, 3162–3165.

(33) Kametani, Y.; Satoh, T.; Miura, M.; Nomura, M. *Tetrahedron Lett.* **2000**, *41*, 2655–2658.

(34) Cole, C. A.; Pan, H.-L.; Namkung, M. J.; Fletcher, T. L. *J. Med. Chem.* **1970**, *13*, 565–567.

(35) Nobuta, T.; Kawabata, T. *Chem. Commun.* **2017**, *53*, 9320–9323.

(36) Amatore, C.; Broeker, G.; Jutand, A.; Khalil, F. *J. Am. Chem. Soc.* **1997**, *119*, 5176–5185.

(37) Saga, Y.; Han, D.; Kawaguchi, S.; Ogawa, A.; Han, L.-B. *Tetrahedron Lett.* **2015**, *56*, 5303–5305.

(38) Bianchini, C.; Lee, H. M.; Meli, A.; Oberhauser, W.; Peruzzini, M.; Vizza, F. *Organometallics* **2002**, *21*, 16–33.

(39) Herrmann, W. A.; Thiel, W. R.; Broißmer, C.; Öfele, K.; Priermeier, T.; Scherer, W. *J. Organomet. Chem.* **1993**, *461*, 51–60.

(40) Ludwig, M.; Strömberg, S.; Svensson, M.; Åkermark, B. *Organometallics* **1999**, *18*, 970–975.

(41) Mann, G.; Baranano, D.; Hartwig, J. F.; Rheingold, A. L.; Guzei, I. A. *J. Am. Chem. Soc.* **1998**, *120*, 9205–9219.

(42) Patiny, L.; Borel, A. *J. Chem. Inf. Model.* **2013**, *53*, 1223–1228.
